# Degradation of G-quadruplex-binding proteins in chromatin using G4-ligand-based proteolysis-targeting chimeras

**DOI:** 10.1038/s41557-026-02111-y

**Published:** 2026-03-19

**Authors:** Zixuan Wang, Xuan He, Xiaoyun Zhang, Jochen Spiegel, Sean M. Flynn, Shankar Balasubramanian

**Affiliations:** 1 Yusuf Hamied Department of Chemistry, University of Cambridge, Cambridge, UK; 2 Cancer Research UK Cambridge Institute, Li Ka Shing Centre, University of Cambridge, Cambridge, UK; 3 School of Clinical Medicine, University of Cambridge, Cambridge, UK

**Keywords:** Proteins, Small molecules, Small molecules, Nucleic acids, Transcription factors

## Abstract

Targeted protein degradation can intervene with the function of disease-related proteins, but most current approaches rely on direct ligand engagement of a protein target, limiting their applicability to proteins that are difficult to bind selectively. Here we present a conceptually unique approach to degrade proteins associated with DNA G-quadruplex (G4) secondary structures in a chromatin context. G4s are non-canonical nucleic acid structures that form at regulatory regions of transcriptionally active genes in open chromatin, and are abundant in cancer states. Although many proteins recognize or regulate G4 structures, selectively targeting G4-binding proteins in their native chromatin environment is challenging. Our bifunctional molecules are proteolysis-targeting chimeras that bind naturally occurring G4s, recruit E3 ubiquitin ligases and degrade G4-specific transcription factors and chromatin remodellers such as FUS, SMARCA4 and ATRX. These proteins are important therapeutic targets that play crucial roles in transcription regulation and DNA repair. Our approach has the potential to be exploited in a therapeutic strategy to target diseases characterized by aberrant G4 activity, such as cancers.

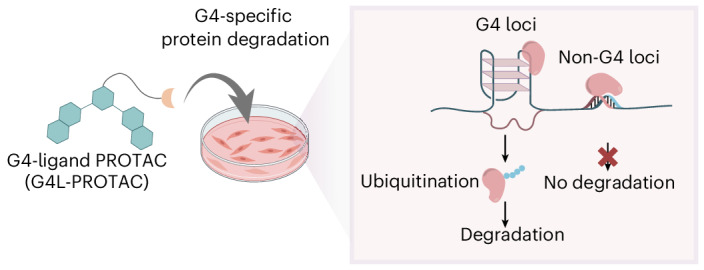

## Abstract (web summary)

DNA G-quadruplex (G4) structures can form at regulatory regions of transcriptionally active genes in open chromatin and are abundant in cancer states, but targeting G4-binding proteins in their native chromatin environment is challenging. Now bifunctional molecules that bind naturally occurring G4 sites and recruit ubiquitination machinery facilitate the degradation of G4-specific transcription factors and chromatin remodellers.

## Main

DNA–protein interactions are fundamental to essential cellular processes that include transcription, replication and genome maintenance^1^. Interfering with DNA–protein interactions has therapeutic potential in diseases such as cancer, where dysregulation of these interactions contributes to disease progression^2–4^. Pharmacological modulation of DNA–protein interactions within native chromatin is challenging due to the complexity of chromatin architecture, the dynamic and transient nature of these interactions, and the frequent absence of well-defined binding pockets on DNA-binding proteins^5,6^. Proteolysis-targeting chimeras (PROTACs) typically bind to the target and recruit the ubiquitin–proteasome system to degrade the target protein, although a binding pocket is still required on the target protein^7–9^. Here, we describe a differentiated approach whereby a PROTAC first binds to a nucleic acid feature in chromatin, then orchestrates degradation of proximal, associated proteins. This approach has several advantages. First, multiple proteins and/or protein complexes associated with chromatin can be degraded due to the targeting of a common DNA feature in their proximity. Second, the target proteins are not required to have a small-molecule binding site^10^. Third, this approach enables degradation in a chromatin-specific context, allowing investigation of protein function in its native chromatin environment.

**Fig. 1:**
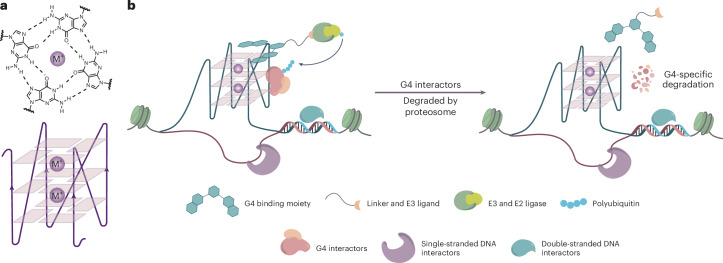
Schematic for G4L-PROTACs. **a**, A G-tetrad, formed by four Hoogsteen base-paired guanines stabilized by a monovalent cation M^+^ (top), and a parallel G4 structure composed of stacked G-tetrads (bottom). **b**, A schematic representation of the G4L-PROTAC strategy. The G4L-PROTAC consists of a small-molecule G4-binding ligand coupled to an E3 ligase recruiter via a linker. Upon treatment, the G4L-PROTAC binds to G4 structures in chromatin to bring the E3 ligase proximal to nearby proteins. This facilitates the ubiquitination of G4-associated protein, without requiring direct protein interaction by the PROTAC warhead, and their subsequent degradation by the proteasome.

Here, we describe the targeting of DNA secondary structures called G-quadruplexes (G4s), as anchoring points in chromatin for PROTACs. G4s are non-canonical four-stranded structures that can form in certain G-rich nucleic acid sequences. Their general structure comprise stacked arrays of hydrogen-bonded guanines arranged in a planar G-tetrad, stabilized by cations^11^ (Fig. 1a). DNA G4s have been detected in open chromatin^12,13^, enriched in the promoters of transcriptionally active genes that include several oncogenes, suggestive of their active involvement in cancer^13,14^. Notably, G4s are particularly abundant in cancer states^15,16^. Individual proteins and protein assemblies involved in the control of gene expression, including transcription factors and chromatin modellers, can bind to G4s and are enriched at G4 sites in cells^17–19^. A notable example is the SWI/SNF chromatin remodeller complex, a multisubunit assembly that regulates gene expression by repositioning nucleosomes to facilitate transcription, which plays critical regulatory roles in disease states, including cancers^3,20^. We have previously developed a range of small molecules that bind G4s, such as pyridostatin (PDS), and have recently demonstrated that a diazirine-modified variant of PDS can be used to photo-crosslink proximal G4-binding transcription factors and chromatin modellers^21^. These observations inspired us to explore bifunctional PROTACs that bind G4s (G4L-PROTACs) and degrade proteins that either bind directly to G4s or are part of protein assemblies proximal to G4s (Fig. 1b). Because G4 formation is linked with active transcriptional and cancer states, a G4L-PROTAC approach represents a unique method to degrade key regulatory proteins involved in transcription and cancer mechanisms^15,16^.

Recent studies have reported oligonucleotide-based G4-PROTACs that tether synthetic oligonucleotides containing G4 motifs to E3 ligase ligands to degrade model proteins such as DHX36^22,23^. Such studies deploy exogenous nucleic acids to bind proteins, rather than targeting natural G4s in chromatin and the natural protein context of such sites. Here, we report a small-molecule system—G4L-PROTACs—that engages natural, genomic G4 structures to drive targeted degradation of chromatin-bound G4-binding proteins in living cells. We demonstrate the degradation of known G4-associated transcription factors and chromatin regulators such as FUS^24,25^ and ATRX^26,27^, and we also identify distinct G4-binding targets, including the transcription factor SOX2 and the splicing component SNRNP70. This small-molecule-based strategy enables efficient and selective degradation of multiple G4-binding proteins at the G4 sites within chromatin, but not other regions in chromatin, which may present an alternative class of therapeutics.

## Results

### Design of G4L-PROTACs

**Fig. 2:**
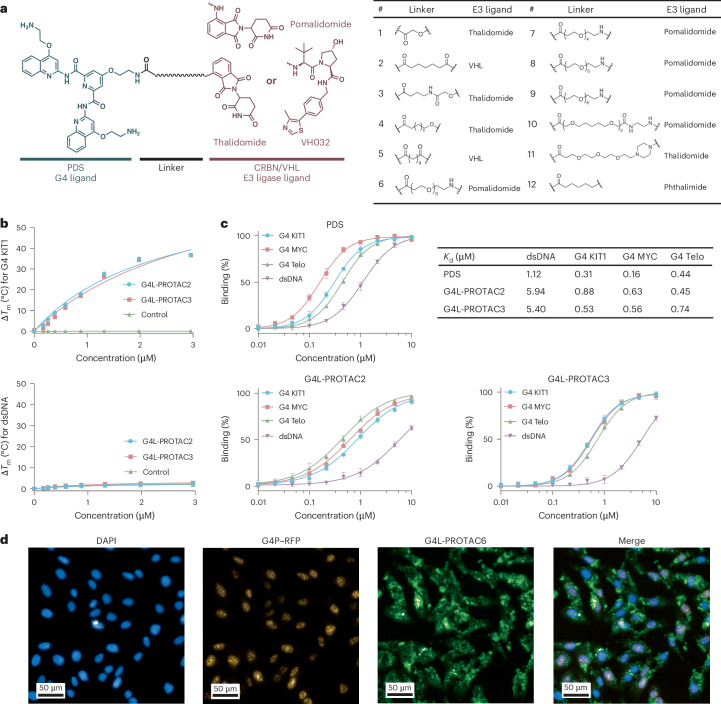
G4L-PROTACs bind to G4s selectively. **a**, Chemical structures of G4-ligand PROTACs (**1**–**12**). **b**, Thermal melting shift (Δ*T*_m_) titrations for G4 KIT1 (top) and dsDNA (bottom) upon increasing concentrations of **G4L-PROTAC2** and **G4L-PROTAC3**. Δ*T*_m_ was measured by a FRET melting assay. Data are presented from two independent experiments (*n* = 2). **c**, Fluorescence quenching assay confirming G4L-PROTAC binding to G4 structures with nanomolar dissociation constants. Mean and error (± standard deviation (s.d.)) are from four independent experiments (*n* = 4). **d**, Subcellular localization of **G4L-PROTAC6** in U2OS cells imaged by confocal microscopy. Representative confocal images of U2OS cells transfected with a nuclear G4-binding reporter plasmid (G4P–RFP) and treated with a fluorescent **G4L-PROTAC6**. The 4′,6-diamidino-2-phenylindole (DAPI, blue) marks nuclear DNA; G4P–RFP (yellow) marks intranuclear G4 structures; **G4L-PROTAC6** (green) shows the intracellular distribution of this compound; the merged image shows partial nuclear colocalization of **G4L-PROTAC6** with G4P–RFP-labelled foci and DAPI-stained nuclei. Quantification of nuclear versus cytoplasmic compound fluorescence was performed on *n* = 48 individual measurements using Revvity Harmony 5.3; values are reported as the percentage of nuclear signal (54%). Representative images are shown from six independent experiments repeated with similar results (*n* = 6). Source data

We designed and synthesized a series of G4L-PROTACs based on the small molecule PDS, which binds G4s in cells^28,29^ (Fig. 2a). Linker length is critical for efficient target engagement and degradation^30^. We therefore generated constructs with E3 ligase ligands, thalidomide^31^, pomalidomide^32^ (for CRBN) and VH032 (for VHL)^33^, coupled to PDS, with flexible linkers ranging from 2 to 27 atoms to enable degradation of proteins at proximities 10–80 Å from the G4 site (Fig. 2a and Supplementary Table 6). We also synthesized a Control-PROTAC probe (**12**) lacking the E3 ligase binding moiety (Fig. 2a).

**Extended Data Fig. 1:**
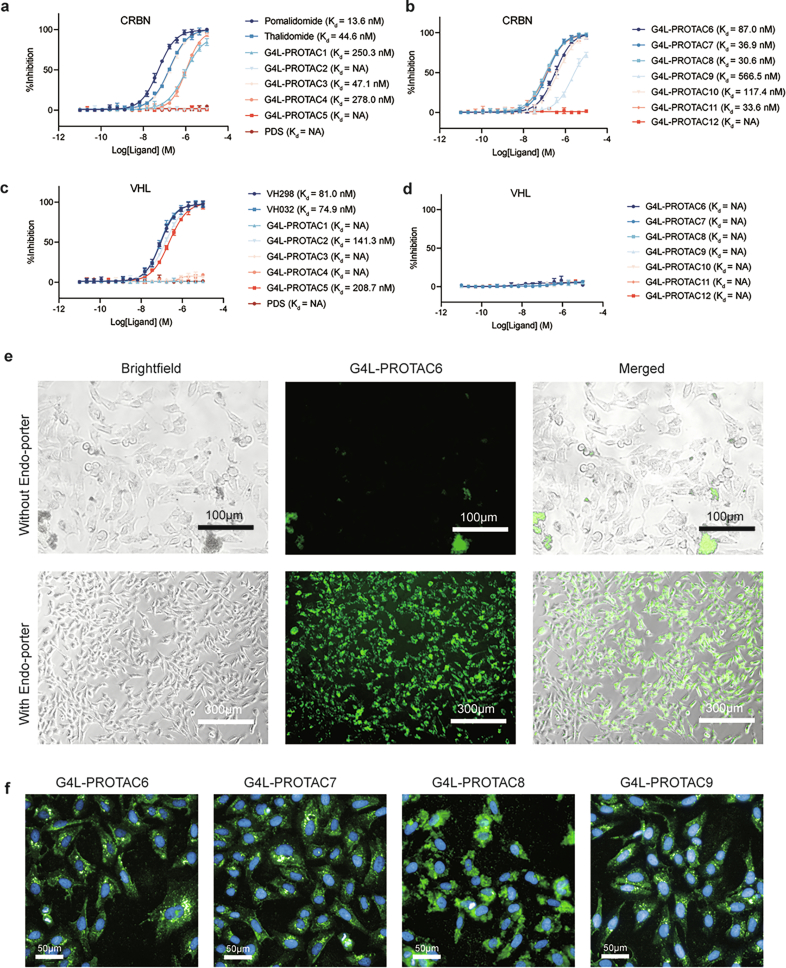
Binding affinity, cellular uptake, and subcellular localization of G4L-PROTACs. **a**, **b**, CRBN-binding G4L-PROTACs tested using a BODIPY FL–thalidomide probe in competition with thalidomide, pomalidomide, and G4L-PROTACs **1**–**12**. Data are presented as mean ± s.d. with individual data points overlaid; n = 3 independent experiments. **c**, **d**, VHL-binding G4L-PROTACs tested using a BODIPY FL–VH032 probe in competition with VH032, VH298, and G4L-PROTACs **1**–**12**. Data are presented as mean ± s.d. with individual data points overlaid; n = 3 independent experiments. **e**, Fluorescence microscopy imaging showing that **G4L-PROTAC6** (green) does not efficiently enter live cells in the absence of Endo-Porter, whereas co-treatment with Endo-Porter facilitates robust intracellular delivery. Brightfield, GFP channel, and merged images are shown. Representative images are shown from six independent experiments repeated with similar results (n = 6). **f**, Confocal microscopy images of U2OS cells treated with fluorescent **G4L-PROTAC6**, **G4L-PROTAC7**, **G4L-PROTAC8**, or **G4L-PROTAC9** in the presence of Endo-Porter. Green indicates G4L-PROTAC signal; nuclei were counterstained with DAPI (blue). G4L-PROTACs distributed to both the cytoplasm and nucleus, with ~54% of the signal localized to the nucleus, indicating efficient nuclear penetration. Scale bars, 100 µm (a) and 20 µm (b). Representative images are shown from six independent experiments repeated with similar results (n = 6). Source data

We performed fluorescence polarization assays for both CRBN- and VHL-recruiting compounds to determine dissociation constant (*K*_d_) values. G4L-PROTACs **1**–**11** retained strong binding to their respective E3 ligases, confirming that the linker and ligase-recruiting moieties do not impair E3 engagement (Extended Data Fig. 1a–d). To assess the G4 binding affinity and selectivity of some G4L-PROTACs, we used a fluorescence resonance energy transfer (FRET) thermal melting assay to determine ligand-stabilized G4 structures, as indicated by the shift in thermal melting transition (Δ*T*_m_)^34^. We evaluated two G4L-PROTACs with thalidomide (**2**) and VH032 (**3**) as E3 ligase ligand, and both showed strong G4 stabilization, comparable to the unmodified parent compound thalidomide, across a panel of well-characterized DNA G4 oligonucleotides, including G4 KIT1, G4 MYC and G4 Telo (Fig. 2b and Supplementary Table 1). These G4L-PROTACs also exhibited relatively less stabilization of double-stranded DNA (dsDNA), indicating selectivity for G4 structures (Fig. 2b). Using the fluorescence quenching-based ligand binding assay^35^, we showed G4 binding of G4L-PROTACs, with apparent dissociation constants (*K*_d_) comparable to those of the parent ligand (Fig. 2c and Supplementary Table 2). For example, **G4L-PROTAC3** exhibited a *K*_d_ of 532 ± 35 nM for G4 KIT1 as compared with PDS (*K*_d_ = 305 ± 18 nM for G4 KIT1) (Fig. 2c). Thus, the G4-binding capability of PDS is substantially preserved in the G4L-PROTACs.

**Extended Data Fig. 2:**
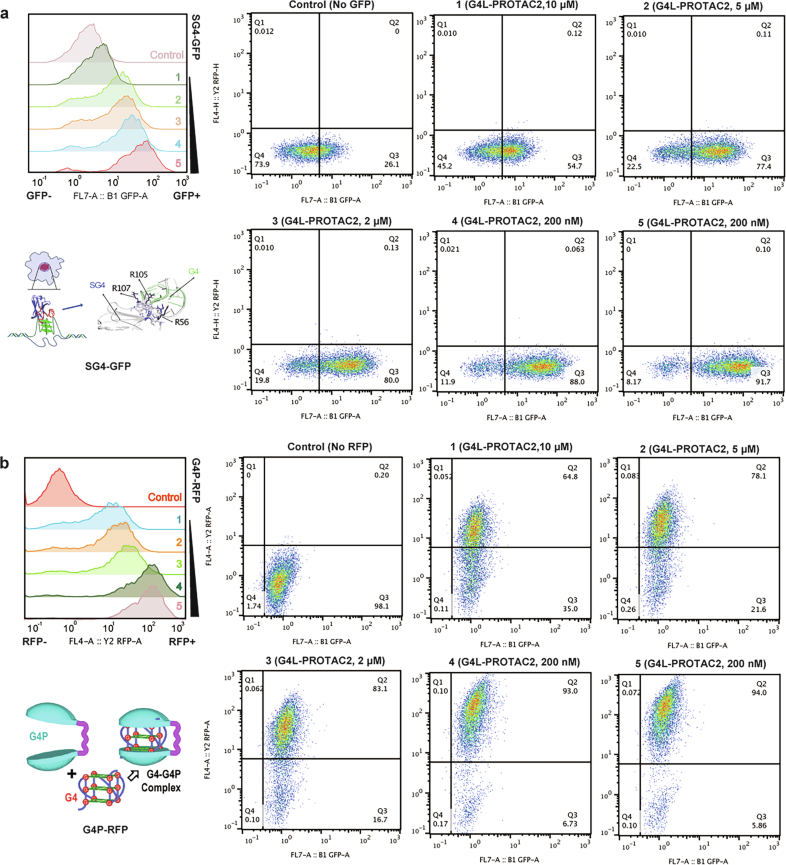
G4L-PROTACs induce degradation of exogenous G-quadruplex binding proteins. **a**, Flow cytometry analysis of U2OS cells expressing SG4-GFP, a G4-specific nanobody fused to GFP, following treatment with different concentration of **G4L-PROTAC2** (1–5: 10 μM, 5 μM, 2 μM, 200 nM, 20 nM treatments respectively). Histograms (top left) and dot plots (right) show a dose-dependent reduction in GFP fluorescence, indicating degradation of the SG4-GFP reporter. A schematic of the SG4-GFP construct and its G4 binding is shown bottom left. **b**, Flow cytometry analysis of U2OS cells expressing G4P-RFP, a G4-binding protein fused to RFP, after treatment with different concentration of **G4L-PROTAC2** (1–5: 10 μM, 5 μM, 2 μM, 200 nM, 20 nM treatments respectively). Reduced RFP signal (top left histogram) and corresponding dot plots (right) confirm target degradation. Schematic illustration of G4P-RFP and its G4 interaction is shown bottom left. In both panels, control represents doxycycline untreated cells.

To assess the performance of G4L-PROTACs, we investigated their effects on known G4-interacting proteins. U2OS cells (human bone osteosarcoma epithelial cells) were treated with varying concentrations of G4L-PROTACs for 12 h. Using the natural fluorescence of pomalidomide/thalidomide-based G4L-PROTACs, we observed that they did not permeate live cells very well^36^ (Extended Data Fig. 1e). We overcame this issue using Endo-Porter, a peptide known to promote endocytosis^37^. G4L-PROTACs were delivered into live cells using Endo-Porter and monitored by fluorescence microscopy (Fig. 2d and Extended Data Fig. 1e), with over 50% of the intracellular fluorescence localized to the nucleus (Extended Data Fig. 1f). As initial proof of concept, we demonstrated G4L-PROTAC-induced degradation of an engineered G4-specific antibody, SG4–GFP and of a nuclear G4-binding reporter (G4P–RFP), using flow cytometry^12,38^ (Extended Data Fig. 2a,b).

### G4L-PROTACs induce degradation of G4-binding protein

**Fig. 3:**
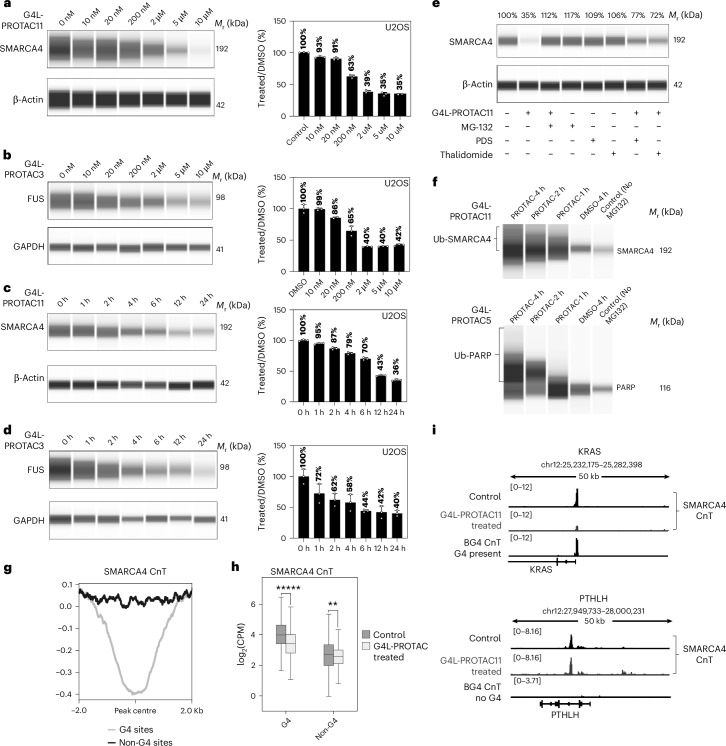
G4L-PROTACs promotes the proteasomal degradation of G4-binding proteins. **a**, Western blot analysis showing dose-dependent degradation of SMARCA4 protein in U2OS cells treated with **G4L-PROTAC11** for 12 h. A marked reduction (>60%) is observed at concentrations of 200 nM and above. Representative blots from one of three biological replicates. Data in the bar plot are presented as mean ± s.d. from three independent biological replicates (*n* = 3). *M*_r_, relative molecular mass. **b**, Western blot analysis showing dose-dependent degradation of FUS protein in U2OS cells treated with **G4L-PROTAC3** for 12 h (200 nM induces substantial degradation). Representative blots from one of three biological replicates are shown. Data in bar plot are presented as mean ± s.d. from three independent biological replicates (*n* = 3). **c**, Time course showing a decline in SMARCA4 protein levels. U2OS cells were treated with 2 µM **G4L-PROTAC11**. Protein levels decrease as early as 2 h post-treatment and remain consistently low for 24 h. Data in bar plot are presented as mean ± s.d. from three independent biological replicates (*n* = 3). **d**, Time-course analysis showing the degradation of FUS in U2OS cells treated with 2 µM **G4L-PROTAC3**, with a significant reduction observed within 1 h and sustained for 24 h. Data in the bar plot are presented as mean ± s.d. from three independent biological replicates (*n* = 3). **e**, Competitive inhibition assays. Incubation with excess PDS or thalidomide shows reduced SMARCA4 and FUS degradation by PROTAC11. Proteasomal inhibition rescues SMARCA4 degradation in U2OS cells treated with 10 μM **G4L-PROTAC11** followed by 10 μM MG-132. Representative blots are shown from independent replicates with similar results (*n* = 3). **f**, Ubiquitination assays demonstrating increased ubiquitination of SMARCA4 and PARP1 upon **G4L-PROTAC11** and **G4L-PROTAC5** treatments. Representative results from three independent experiments (*n* = 3). **g**, Metaplot of SMARCA4 CUT&Tag signal in U2OS cells treated with **G4L‑PROTAC11** versus untreated control, averaged across BG4‑defined G4 sites and matched non‑G4 sites. Lines show the mean across four independent biological replicates (*n* = 4). The number of sites included: G4 sites *n* = 8,568, non‑G4 sites *n* = 6286. **h**, Box plots comparing SMARCA4 CUT&Tag signal (log_2_CPM) at BG4‑defined G4 sites versus matched non‑G4 sites for control and G4L‑PROTAC‑treated conditions. Box plots show the median (centre line), interquartile range (box, 25th–75th percentile) and whiskers (1.5× interquartile range). Two‑sided *t*‑test comparing SMARCA4 CUT&Tag signal (log_2_CPM) between PDS control and G4L‑PROTAC‑treated samples at BG4‑defined G4 sites and matched non‑G4 sites. G4 sites: *t* = 28.296, *P* = 1.90 × 10^−173^. Non‑G4 sites: *t* = −4.842, *P* = 1.30 × 10^−6^ (positive *t* indicates higher signal in PDS than G4L-PROTAC). Data represent four independent biological replicates (*n* = 4). CPM, counts per million mapped reads. **i**, Genome browser views for SMARCA4 binding identified by CUT&Tag, following G4L-PROTAC or control treatments, compared with G4 sites mapped by G4-CUT&Tag (using BG4 antibody) at the KRAS (G4-positive) and PTHLH (G4-negative) loci. Source data

**Extended Data Fig. 3:**
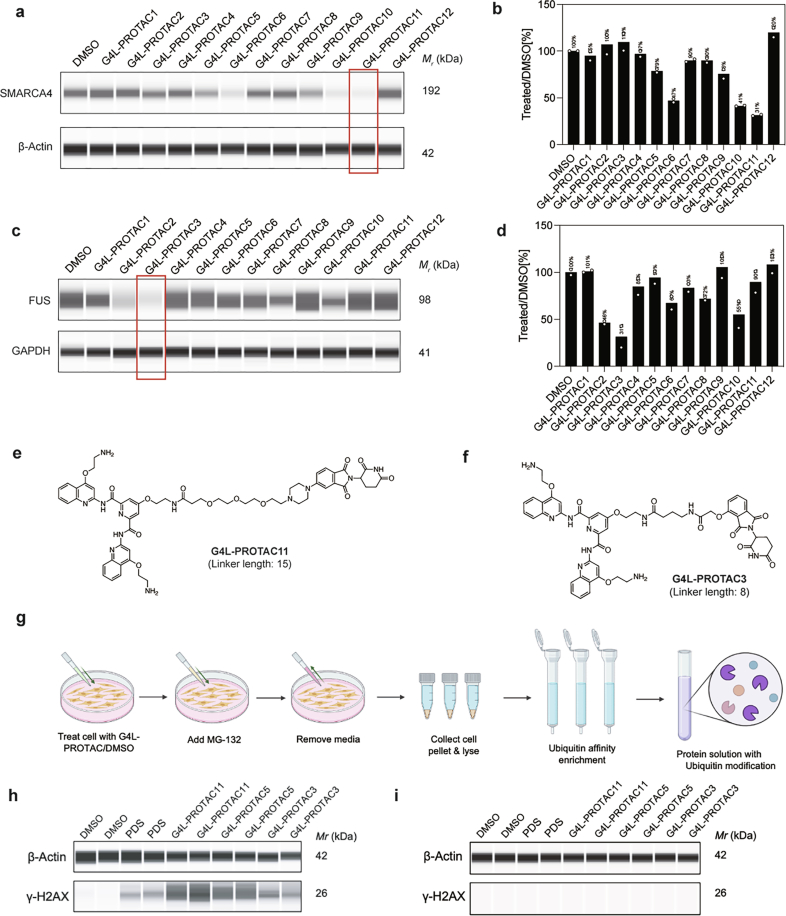
G4L-PROTACs degrade G4-binding proteins via the proteasome system. **a**–**d**, Western blot analysis of SMARCA4 (a-b) and FUS (c-d) in U2OS cells treated with different G4L-PROTACs (2 μM) for 12 h in the presence of Endo-Porter. β-Actin and GAPDH were used as loading controls. Selected compounds (red-boxed) induced most reduction in target protein levels. **e**, **f**, Chemical structures of **G4L-PROTAC11** (linker length: 15 atoms) and **G4L-PROTAC3** (linker length: 8 atoms), respectively. Where quantification is shown, dots showing individual biological replicates; n = 2 independent biological replicates (independently treated cultures). **g**, Schematic overview of the tandem ubiquitin-binding entity (TUBE)-based ubiquitination enrichment assay. Cells were treated with G4L-PROTAC (2 μM) and MG-132 (10 μM) for 6 h, lysed, and subjected to polyubiquitin affinity enrichment. Schematic created with BioRender.com. **h**, Western blot analysis of γH2AX levels in U2OS cells treated with DMSO, PDS, or indicated G4L-PROTACs (10 μM, 72 h). G4L-PROTAC treatment induces DNA damage responses comparable to or exceeding those of PDS, as indicated by increased γH2AX levels, while β-actin serves as loading control. Representative blots are shown from two independent biological replicates. **i**, Short-term exposure (12 h) to 2 μM of the same compounds in U2OS cells does not induce γH2AX signal, indicating that G4L-PROTAC–mediated DNA damage requires longer treatment duration and/or higher concentrations to manifest detectable effects. Representative blots are shown from two independent biological experiments. Illustrations in **g** created in BioRender; Wang, Z. https://biorender.com/4h068d6 (2026). Source data

**Extended Data Fig. 4:**
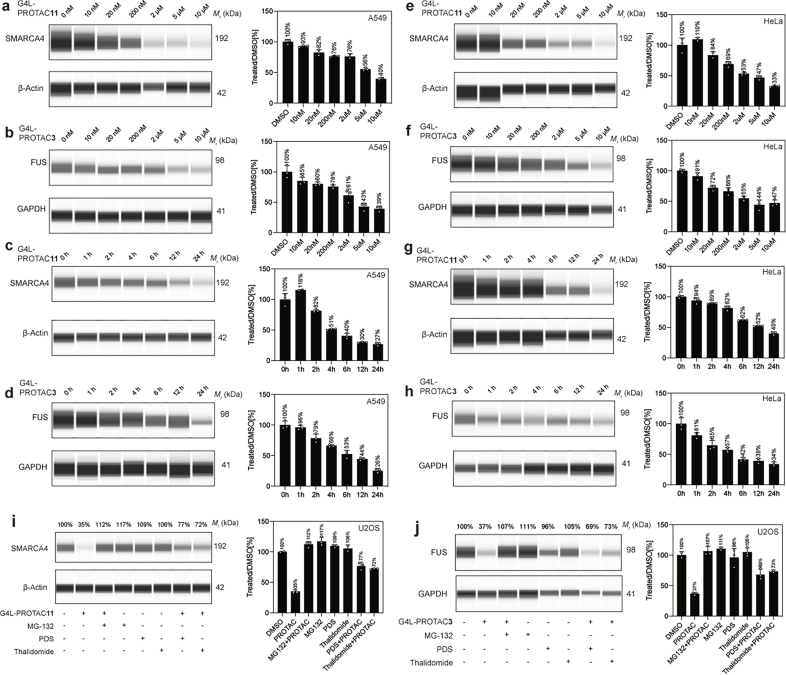
G4L-PROTAC–induced degradation of target proteins in multiple cancer cell lines and proceeds via a proteasome-dependent mechanism. **a**–**h**, Western blot analysis and quantification of target protein degradation in A549 (left) and HeLa (right) cells treated with **G4L-PROTAC11** (targeting SMARCA4, panels **a**, **b**, **e**, **f**) and **G4L-PROTAC3** (targeting FUS, panels **c**, **d**, **g**, **h**) for 0 – 24 h across a concentration range (0 –10 μM). β-Actin and GAPDH were used as loading controls. Time and dose-dependent degradation of SMARCA4 and FUS was observed in both cell lines. Quantification is shown as mean ± s.d. with dots representing individual biological replicates (n = 3). **i**, **j**, Competition assays in A549 cells treated with **G4L-PROTAC11** (**i**) or **G4L-PROTAC3** (**j**) in the presence or absence of excess thalidomide or PDS. Degradation of SMARCA4 (**i**) and FUS (**j**) was attenuated by co-treatment with either competitor, consistent with a quatenary complex–dependent and proteasome-mediated degradation mechanism. Quantification is shown as mean ± s.d. with dots representing individual biological replicates (n = 3). Source data

We tested the G4L-PROTACs for degradation of G4-binding proteins SMARCA4 and FUS as representative targets, because both proteins can bind G4s, occupy sites where G4s form in gene promoters^39^ and were previously identified via photo-crosslinking to a PDS-based probe in cells^21^. U2OS cells were treated with varying concentrations of G4L-PROTACs in combination with Endo-Porter for 12 h, followed by protein analysis via western blotting (Extended Data Fig. 3a–d). **G4L-PROTAC3** caused degradation of FUS in a dose-dependent manner, with 2 µM being sufficient to reduce FUS protein levels to below 50% (Fig. 3b and Extended Data Fig. 3c,d). Similarly, 2 µM **G4L-PROTAC11** led to 60% reduction in SMARCA4 levels (Fig. 3a and Extended Data Fig. 3a,b). By contrast, the negative control G4L-PROTAC12* does not cause degradation of either SMARCA4 or FUS, even at 10 µM concentration (Extended Data Fig. 3a–d). Time-course experiments using 2 µM of **G4L-PROTAC11** revealed 60% decline in SMARCA4 after 12 h, with these levels remaining consistently low up to 48 h (Fig. 3c and Extended Data Fig. 3a). Treatment with 2 µM of **G4L-PROTAC3** resulted in a 30% reduction of FUS levels within just 1 h, which further decreased to 60% after 12 h and remained low for up to 48 h (Fig. 3d and Extended Data Fig. 3b). These findings highlight robust and sustained degradation of endogenous SMARCA4 and FUS by G4L-PROTACs with different linkers and different E3 ligase ligands. We further validated G4L-PROTAC activity in A549 and HeLa cells, confirming target degradation across a number of cancer cell types (Extended Data Fig. 4a–h).

To confirm the degradation mechanism, we performed proteasome inhibition and ligand competition experiments^40^. First, we treated cells with the proteasome inhibitor MG-132 in combination with G4L-PROTACs. Western blot analysis showed MG-132 inhibits degradation of SMARCA4, suggesting that G4L-PROTAC-induced protein degradation is dependent on the proteasome pathway (Fig. 3e). Moreover, increased ubiquitination of SMARCA4 and PARP1 was observed upon G4L-PROTAC and MG-132 treatment, as determined by a tandem ubiquitin-binding entity-based enrichment assay^41^, confirming degradation via the ubiquitin–proteasome pathway (Fig. 3f and Extended Data Fig. 3g). Introduction of PDS or thalidomide, as competitive inhibitors for G4 binding and E3 ligase recruitment, respectively, causes reduction in the degradation effect of G4L-PROTACs, supporting the dependence on both G4 binding and ubiquitin ligase recruitment in the mechanism (Fig. 3e and Extended Data Fig. 4i,j).

### G4L-PROTACs selectively deplete proteins from G4 regions

Given that our approach targets the degradation of proteins in the context of G4 formation, a G4L-PROTAC might cause greater and more selective loss of protein at the key chromatin sites, rather than simply depleting the protein pool. This would be particularly relevant for chromatin-interacting proteins such as SMARCA4 and FUS, which are known to associate with both G4 and non-G4 genomic regions. We evaluated this by profiling SMARCA4 and FUS chromatin-binding sites in U2OS cells treated with **G4L-PROTAC11** or **G4L-PROTAC3** relative to an untreated control, using CUT&Tag with primary antibodies specific for each protein^42,43^ (Extended Data Fig. 6a and Supplementary Fig. 1). In cells untreated with G4L-PROTACs, some SMARCA4 and FUS binding sites overlap with regions where G4s are folded, as mapped by the G4-antibody BG4^44^, and both proteins also show binding sites at non-G4 regions (Extended Data Fig. 6b). Upon G4L-PROTAC treatment, we observed an overall reduction in SMARCA4 and FUS binding to chromatin (Extended Data Fig. 6c, d and Supplementary Figs. 12 and 13). Importantly, there was a marked reduction in SMARCA4 or FUS protein binding signals at G4 sites, with relatively little change in protein binding at non-G4 sites (see Fig. 3g,h for SMARCA4 and Extended Data Fig. 6e,f for FUS; Supplementary Figs. 4 and 6–9). These results are exemplified by the mapping of SMARCA4 binding at specific genomic loci and visualized as peaks in a genome browser view. For example, at the G4-positive KRAS locus, SMARCA4 shows distinct binding peaks that are lost following G4L-PROTAC treatment, whereas SMARCA4 binding is unaltered at the non-G4 site within the PTHLH genes (Fig. 3i and Supplementary Fig. 5). This is consistent with G4L-PROTACs acting at G4s in the chromatin context and suggests that the kinetics of locally induced degradation at G4s is quicker than replenishment at these chromatin sites from the protein pool, for SMARCA4 and FUS in U2OS cells.

To evaluate whether G4L-PROTAC-mediated degradation of G4-binding proteins alters G4 structure formation or stability, we performed BG4 CUT&Tag (Extended Data Fig. 7a), under conditions that allowed protein degradation without detectable DNA damage (Extended Data Fig. 3i). PDS treatment alone resulted in 195 significantly reduced G4 peaks, while **G4L-PROTAC3** and **G4L-PROTAC11** led to a reduction in G4 signal for 3,116 and 1,460 G4 peaks, respectively, out of a total of ~20,000 G4 peaks (false discovery rate (FDR) <0.05). Therefore, G4L-PROTACs do cause a reduction for a small subset (7–15%) of the global G4s detected (Extended Data Fig. 7b–d). This group of reduced G4 sites was enriched in promoters and introns, and the associated G4s may have some dependency on the degraded G4-binding proteins for stability or detection.

**Fig. 4:**
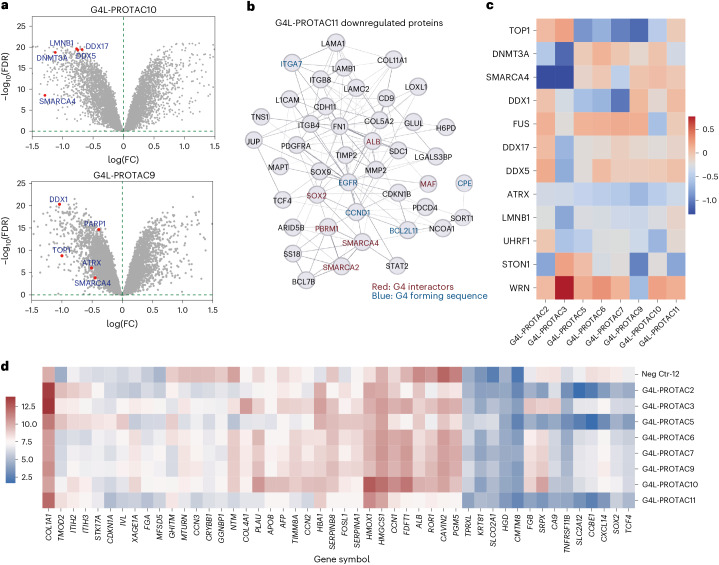
The degradation profiles of G4L-PROTACs. **a**, Volcano plot of quantitative proteomics from U2OS cells treated with **G4L-PROTAC10** (top) or **G4L-PROTAC9** (bottom) relative to control. Each point represents a protein (*x* axis, log_2_ fold change (FC); *y* axis, −log_10_ *P* value). Significantly downregulated proteins, include G4-binding proteins (for example, SMARCA4, TOP1, DNMT3A, LMNB1 and ATRX) are highlighted in red. Representative data from three biological replicates are shown. Significance threshold: log_2_ fold change <–0.585; FDR <0.05. **b**, PPI network (STRING/cytoscape) for significantly downregulated proteins after **G4L-PROTAC11** treatment. Red nodes indicate known G4-binding proteins (G4 interactors); blue nodes indicate proteins encoded by genes with G4-forming sequences. **c**, Heatmap of differential proteomic profiles showing unique G4-binding protein degradation patterns for eight G4L-PROTACs, with variations attributed to linker length and selectivity. Values shown are mean log_2_ fold change across three independent biological replicates per compound (*n* = 3). **d**, Heatmap of differential proteomic profiles showing unique total protein degradation patterns for eight G4L-PROTACs, with variations attributed to linker length and selectivity. Values shown are mean log_2_ fold change across three independent biological replicates per compound (*n* = 3). Neg Ctr-12, negative control compound G4L-PROTAC12* lacking the E3 ligase recruitment moiety. Source data

For an unbiased, global analysis of protein degradation by G4L-PROTACs, we performed mass-spectrometry-based proteomic profiling in U2OS cells for all eight G4L-PROTACs (Fig. 4a, Extended Data Fig. 8a and Supplementary Figs. 18 and 19). In the proteomics analysis, proteins were considered significantly degraded if they met both a fold change and statistical significance threshold (log_2_ fold change <–0.585; FDR <0.05). This dual criterion balances biological relevance with statistical confidence, ensuring that only proteins showing reproducible and meaningful decreases in abundance were classified as degraded. For example, **G4L-PROTAC10** significantly downregulated a great number of known G4-binding proteins, including SMARCA4, TOP1, DNMT3A, LMNB1, ATRX and PARP1 (Fig. 4a and Supplementary Figs. 18 and 19), as determined by comparison with a curated list of previously reported G4-interacting proteins^21,45,46^. Notably, multiple known G4-interacting proteins were recurrently degraded by different G4L-PROTACs. For example, TOP1 showed the most frequent degradation across multiple G4L-PROTACs (**3**, **5**, **6**, **7** and **9**), while SMARCA4 exhibited the largest fold change in degradation, particularly with G4L-PROTACs **6**, **10** and **11**. (Fig. 4a,b and Extended Data Fig. 8a).

In addition to targeting chromatin-associated G4-binding proteins, G4L-PROTACs also induced degradation of several RNA G4-binding proteins, including HNRNPA1^47^ and SRSF1^48^. Quantitative analysis revealed up to ~7% overlap with a curated RNA G4-binding protein dataset across multiple compounds, indicating that G4L-PROTACs may engage both DNA- and RNA-associated G4 protein networks (Extended Data Fig. 9e and Supplementary Table 9). Although the main focus of our study was chromatin-associated G4 proteins, these findings suggest a broader scope of G4L-PROTACs in modulating G4-related proteomes across nuclear and cytoplasmic compartments.

To investigate how G4L-PROTACs perturb cellular protein networks, we performed protein–protein interaction (PPI) analysis focusing on significantly downregulated proteins (FDR <0.05, fold change ≥1.5) from quantitative proteomics (Fig. 4b, Extended Data Fig. 8b and Supplementary Fig. 26). Many of these proteins are highly interconnected, and their coordinated downregulation by G4L-PROTACs suggests that G4 structures may serve as anchoring hubs for assembling functional protein networks in chromatin. Notably, several downregulated proteins are known G4-binding proteins (Fig. 4b), including SMARCA4 and PBRM1, both members of the SWI/SNF chromatin remodelling complex, supporting the on-target mechanism of G4L-PROTACs. We also found that each G4L-PROTAC exhibited a distinct degradation signature. Functional annotation of the resulting clusters revealed pathway-specific enrichment patterns: for example, **G4L-PROTAC2** affected proteins involved in cell migration and transcription regulation, while **G4L-PROTAC3** targeted ubiquitination and cell cycle regulators (Extended Data Fig. 8b). Other compounds were associated with pathways including chromatin remodelling, DNA repair, Wnt signalling and β-catenin binding, depending on their E3 ligase recruiter and linker architecture. These findings support the modular design of G4L-PROTACs and their ability to engage functionally distinct protein networks through tunable degradation. Further Gene Ontology (GO) enrichment analysis indicated that the upregulated proteins were involved in DNA damage response pathways, suggesting that G4L-PROTAC treatment also induces DNA damage within the cells, consistent with the known mode of action of PDS^28,43^ (Extended Data Fig. 3h and Supplementary Figs. 21–23).

Proteomic profiles revealed distinct degradation profiles for the different G4L-PROTACs, owing to the influence of linker length (Extended Data Fig. 9c,f and Supplementary Fig. 20). Comparative heatmap analysis showed that G4L-PROTACs with linkers lengths of at least 7 atoms showed overall degradation of G4-interacting proteins, with the most pronounced overall degradation of G4-interacting proteins effects observed for a G4L-PROTAC bearing a 23-atom linker (**10**), highlighting the importance of spatial orientation and flexibility in target selectivity (Fig. 4c,d and Extended Data Fig. 9c).

### G4L-PROTACs inhibit cell proliferation

**Fig. 5:**
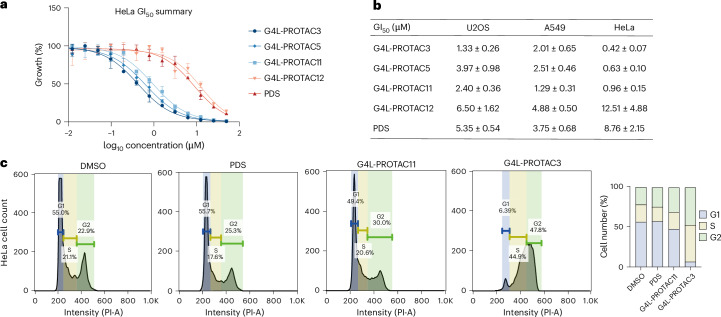
G4L-PROTACs exhibit enhanced antiproliferative and cell cycle arrest effects. **a**, Growth inhibition curves in HeLa cells treated with PDS, **G4L-PROTAC3**, **G4L-PROTAC5**, **G4L-PROTAC11** and the negative control G4L-PROTAC12*. Cells were treated with serial dilutions of each compound for 72 h, and viability was measured using CellTiter-Glo. Data are presented as mean ± s.d. from three independent biological replicates. Curve-fitting model: four‑parameter logistic. **b**, Summary table of GI_50_ values for each compound in U2OS, A549 and HeLa cell lines. **G4L-PROTAC3** and **G4L-PROTAC11** show markedly lower GI₅₀ values in HeLa cells compared with PDS and G4L-PROTAC12*. Values are reported as mean ± s.d. across *n* = 3 independent biological replicates. **c**, Flow cytometry cell cycle analysis of distribution in HeLa cells treated with DMSO, PDS, **G4L-PROTAC11** or **G4L-PROTAC3** (10 μM, 72 h). Left: DNA content was measured using propidium iodide (PI) staining. G4L-PROTAC treatment induced pronounced G2/M arrest relative to controls. Right: quantification of cell populations in G1, S and G2 phases. Source data

**Extended Data Fig. 5:**
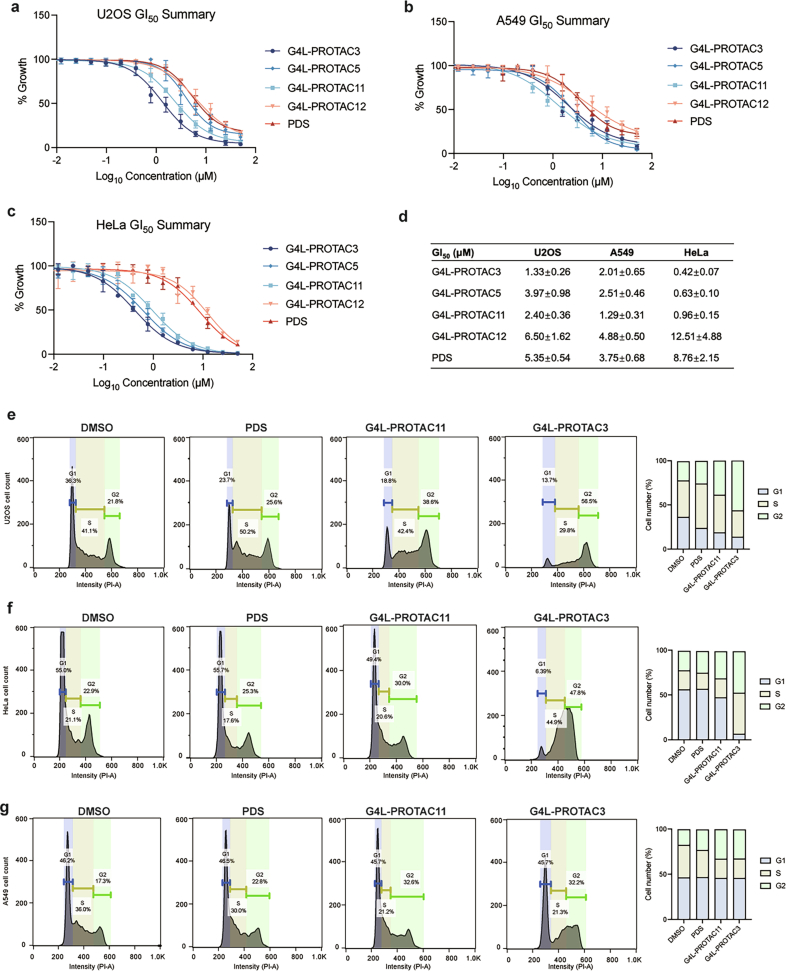
G4L-PROTACs suppress long-term cancer cell proliferation and induce G2/M cell cycle arrest. **a**–**c**, Dose–response curves measuring cell proliferation in U2OS (**a**), A549 (**b**), and HeLa (**c**) cells treated with different concentrations of G4L-PROTAC**3**, **5**, **11**, **12**, and the G4 ligand PDS for 72 h. Cell viability was assessed using CellTiter-Glo assays and normalized to DMSO-treated controls. Data represent mean ± s.d. from three biological replicates. **d**, Summary of GI_50_ values (μM) for each compound in the indicated cell lines. **G4L-PROTAC3** consistently demonstrated the lowest GI_50_ across all cell types tested, while PDS and G4L-PROTAC12* served as less potent controls. GI_50_ values are reported as mean ± s.d. across n = 3 biological replicates. **e**–**g**, Flow cytometry–based cell cycle analysis in HeLa (**e**), U2OS (**f**), and A549 (**g**) cells treated for 72 h with DMSO, PDS, **G4L-PROTAC11**, or **G4L-PROTAC3** (10 μM each). PI staining was used to determine DNA content, and quantification of G1, S, and G2/M phase populations is shown (right). Source data

To evaluate the antiproliferative effects of G4L-PROTACs, we conducted a GI_50_ (the concentration required to inhibit cell growth by 50%) analysis in three cancer cell lines: U2OS, A549, and HeLa (Fig. 5a, Extended Data Fig. 5a–c). Cells were treated with a panel of G4L-PROTACs (**3**, **5**, **11** and **12**) or PDS across a dose range (50 μM to 0.006 μM) for 96 h, and viability was measured using CellTiter-Glo assays. The most potent compound was **G4L-PROTAC3**, with GI_50_ values of 1.33 ± 0.26 μM in U2OS, 2.01 ± 0.65 μM in A549 and 0.42 ± 0.07 μM in HeLa cells (Fig. 5b). In all three cell lines, **G4L-PROTAC3** and **G4L-PROTAC11** demonstrated greater antiproliferative activity than the G4 ligand PDS alone. These findings support the conclusion that dual G4 binding and targeted protein degradation confer enhanced suppression of cancer cell growth. Notably, PDS and the control G4L-PROTAC12*, which contains a phthalimide moiety that is incapable of recruiting E3 ligase and therefore lacks degradation capability, were consistently less potent across all cell models tested.

To investigate the mechanistic basis of growth suppression, we performed cell cycle analysis in U2OS, HeLa and A549 cells treated with dimethyl sulfoxide (DMSO), PDS, **G4L-PROTAC3** or **G4L-PROTAC11** (10 μM, 72 h) (Fig. 5c and Extended Data Fig. 5e–g). PDS treatment led to a modest increase in G2/M accumulation (25.3%) relative to DMSO (22.9%) in HeLa cells, consistent with previous reports that PDS induces replication stress and G2 arrest through stabilization of G4 structures and activation of the DNA damage response^43^. By contrast, **G4L-PROTAC11** caused a stronger G2/M arrest (30.9%), and **G4L-PROTAC3** produced the most pronounced effect, increasing the G2/M population to 47.7% (Fig. 5c). These shifts were accompanied by corresponding reductions in the G1 population, suggesting checkpoint activation. Notably, both G4L-PROTACs induced more substantial G2/M accumulation than PDS or the negative control G4L-PROTAC12*, highlighting the added impact of G4-proximal protein degradation. Consistent trends were observed in U2OS and A549 cells, where G4L-PROTACs also led to increased G2/M arrest compared with controls (Extended Data Fig. 5e–g).

To assess whether G4L-PROTACs induce DNA damage similarly to the G4 ligand PDS, we performed western blotting for γH2AX, a marker of double-strand breaks. U2OS cells treated with representative **G4L-PROTAC3**, **G4L-PROTAC5** and **G4L-PROTAC11** (10 μM, 72 h) showed γH2AX signal, generally exceeding that of PDS (Extended Data Fig. 3h). These observations are consistent with previous reports showing that PDS induces DNA damage and checkpoint activation, including γH2AX phosphorylation and accumulation of cells in G2/M phase^43^. While PDS stabilizes G4s, G4L-PROTACs stabilize G4s and degrade G4-binding proteins. We identified that **G4L-PROTAC3** target FUS, TOP1 and UHRF1, while **G4L-PROTAC5** and **G4L-PROTAC11** deplete chromatin remodellers, including SMARCA4 and PARP1—factors whose loss can impair replication fork stability or G2/M progression. These observations are consistent with the elevated DNA damage signal, as assessed by γH2AX measurement (Extended Data Fig. 3h).

Together, these findings demonstrate that G4L-PROTACs disrupt cell cycle progression more effectively than G4 ligand alone. This is consistent with a dual mechanism of action involving both G4 stabilization and degradation of chromatin-associated factors.

### Discovery of additional G4-binding proteins

**Fig. 6:**
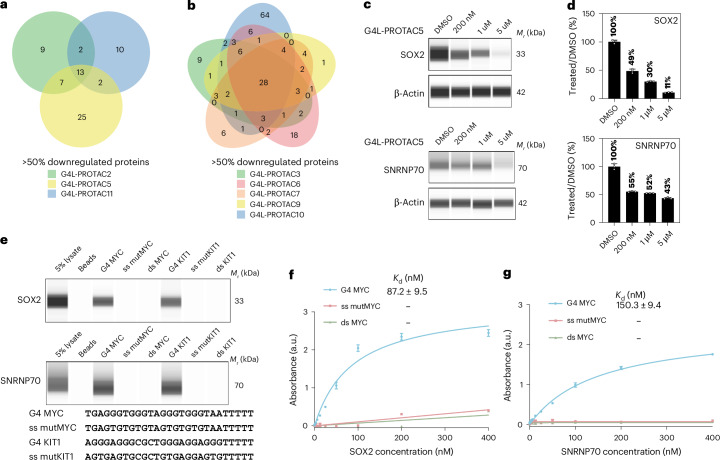
Identification and validation of additional G4-binding proteins. **a**,**b**, Venn diagram showing subsets of proteins downregulated by >50% following different G4L-PROTACs treatments in quantitative proteomics. Panel **a** shows proteins downregulated across **G4L-PROTAC2**, **5** and **11**, whereas panel **b** shows proteins downregulated across **G4L-PROTAC3**, **6**, **7**, **9** and **10**. Data represent the mean from three independent experiments. **c**, Western blot analysis validating SOX2 and SNRNP70 protein degradation after **G4L-PROTAC5** treatment. Representative blots from three independent experiments. **d**, Quantification of SOX2 and SNRNP70 degradation upon G4L-PROTACs treatments. Data represent mean ± s.d. from three biological replicates. **e**, G4 binding of SOX2 and SNRNP70 proteins, as determined by affinity capture and western blotting using the indicated oligonucleotides from U2OS nuclear extracts. Selective interaction of SOX2 and SNRNP70 with MYC or KIT1 G4 structures was observed relative to control oligonucleotides either mutated to abrogate G4 formation or corresponding duplex DNA (G-runs are highlighted in bold). Representative blots shown; *n* = 3 independent biological replicates. ds, double-stranded DNA; ss, single-stranded DNA. **f**,**g**, Binding curves as determined by ELISA for the human recombinant full-length SOX2 (**f**) and SNRNP70 (**g**) protein to G4 MYC, the single-stranded mutant (ss mutMYC) and double-stranded MYC (ds MYC). Apparent dissociation constants (*K*_d_) are indicated. Data are presented as mean ± s.d. from three independent biological replicates. Source data

To investigate whether protein degradation patterns of G4L-PROTACs can identify G4-binding proteins, we performed quantitative proteomics across multiple treatments and identified proteins consistently downregulated by more than 30% or 50% (Fig. 6a,b and Extended Data Fig. 10a–c). SOX2 and SNRNP70 were predicted to form stable complex with G4 MYC and were selected for further validation (Extended Data Fig. 10f,g and Supplementary Fig. 24). SOX2 was consistently downregulated across all eight G4L-PROTAC treatments, while SNRNP70 was degraded by **G4L-PROTAC5** and had previously been identified as a G4-interacting protein by two independent methods, CMPP^21^ and G4-LIMCAP^46^. Western blotting confirmed their degradation upon **G4L-PROTAC5** treatment for 12 h (Fig. 6c,d). To assess their G4-binding properties, we performed affinity pull-down assays using 3′-biotinylated oligonucleotides forming defined G4 structures (for example, MYC and KIT1), alongside single-stranded mutants (ss-mutMYC and ss-mutKIT1) where G4s had been mutated to abrogate G4 formation, and double-stranded controls^49,50^ (Supplementary Table 3). Both SOX2 and SNRNP70 showed selective enrichment with G4 oligonucleotides over controls (Fig. 6e). As pull-down assays may capture indirect interactors, we next performed enzyme-linked immunosorbent assays (ELISAs) using pure, recombinant proteins to measure direct binding affinities (Supplementary Table 5). Both SOX2 and SNRNP70 bound G4 MYC with *K*_d_ values of 87.2 ± 9.5 nM for SOX2 and 150.3 ± 9.4 nM for SNRNP70, while exhibiting negligible binding to control sequences that are either single-stranded or double-stranded (Fig. 6f,g). To further confirm that these G4-binding proteins are G4L‑PROTAC substrates, ubiquitination assays were performed following G4L‑PROTAC treatment. Both SOX2 and SNRNP70 displayed clear ubiquitin conjugation, consistent with their targeted degradation via the ubiquitin–proteasome pathway (Extended Data Fig. 10d,e). These results validate SOX2 and SNRNP70 as previously unrecognized direct G4-binding proteins and are consistent with their selective degradation by G4L-PROTACs. These findings also demonstrate the value of G4L-PROTACs as discovery tool molecules for identifying additional G4-binding proteins.

## Discussion

Here, we describe a chemical degradation approach that exploits naturally occurring G4 structures as anchor points to direct the degradation of selected chromatin-associated proteins in live cells. Unlike standard PROTACs, which directly bind target proteins, G4L-PROTACs recruit E3 ligases to folded G4s at specific sites in chromatin, leading to the local degradation of associated proteins. Importantly, the proteins subjected to degradation are contextually dependent on the presence or absence of the anchoring G4 feature in chromatin. This method offers a distinct conceptual route for targeted protein degradation. Previous attempts to harness G4s for PROTACs have necessitated the introduction of synthetic G4-forming oligonucleotides (DNA or RNA) into cells to recruit proteins for degradation. By contrast, we have deployed bifunctional small molecules that degrade proteins in a natural G4-chromatin context. Our approach differs fundamentally from previous G4-PROTAC strategies, which deployed transfection of exogenous G4 oligonucleotides^22,23^. The G4L-PROTACs we have presented are bifunctional small molecules that directly bind to native DNA G4 structures, enabling the degradation of endogenous G4-associated proteins within their chromatin context. A fundamental feature of our study is that we have used genomic mapping approaches to prove that our G4L-PROTAC degrades G4-associated protein selectively at G4 sites in cellular chromatin. This is key to proving the mechanism of action. This has not been addressed in a recently published study showing degradation of DHX36 in cells^51^.

Using a series of PDS-based G4L-PROTACs, we demonstrated the degradation of natural G4-binding proteins, including transcriptional regulators and chromatin remodellers such as FUS and SMARCA4. The mechanism of their degradation was confirmed to be proteasome dependent and required both G4 binding and E3 ligase recruitment, as demonstrated by chemical competition and inhibition assays. We also found that the degradation of SMARCA4 or FUS occurs preferentially at chromatin regions rich in G4 structures rather than G4-negative regions, where protein occupancy remained largely unchanged. This supports the idea that G4L-PROTACs act on proteins at G4-specific genomic loci rather than by depleting the total cellular pool of target proteins. The results are consistent with previous observations that G4-binding ligands such as PDS localize to open chromatin and gene promoters^23^. Importantly, this approach seems to degrade proteins that function specifically at sites of folded G4s within transcriptionally active chromatin.

Quantitative proteomics showed that many known G4-binding proteins were selectively degraded relative to randomly chosen proteins. Several hundred proteins showed notable changes across eight distinct G4L-PROTACs, with the degradation pattern influenced by linker length and composition. Notably, proteomic analysis revealed that longer linkers tended to increase the efficiency and breadth of target degradation, highlighting the importance of spatial reach in proximity-based degrader design.

G4L-PROTAC-induced degradation may affect proteins that are either directly bound to or localized near G4 structures, or that are part of nearby protein assemblies. Comparative analysis with published G4P chromatin immunoprecipitation and BioID datasets confirmed substantial overlap with known G4-binding proteins, validating our approach. However, we also observed depletion of proteins not previously annotated as G4 binders, such as SMARCA2, WDR5, EP400 and BCLAF1, which we interpret as components of G4-associated chromatin environments (Extended Data Fig. 8b). This suggests the potential of G4L-PROTACs to probe not only direct G4 interactions but also broader G4-proximal protein networks.

Beyond validating known targets, we identified and confirmed additional G4-binding proteins. As examples, we studied two previously unrecognized G4 interactors—SOX2 and SNRNP70—that were found to be consistently degraded by multiple G4L-PROTACs. In validation studies, both proteins were shown biophysically to bind G4 structures with nanomolar affinity in vitro, confirming direct interaction. These results show that G4L-PROTACs can also reveal previously unknown components of the endogenous G4-protein network, thus functioning as tools for biological discovery.

The mechanism of G4L-PROTAC-mediated degradation may involve complex spatial dynamics between the PROTAC, G4 structures and G4-binding proteins. While competition between the G4 ligand (for example, PDS) and proteins for the same G4 is possible, prior studies have shown that ligands (PDS) and proteins can co-occupy G4 sites or bind in proximity within G4-rich loci^21^. In addition, some genomic regions may harbour multiple adjacent G4s, potentially allowing both the PROTAC and its target protein to engage nearby structures^13,52^. However, we cannot rule out the possibility of some loci with only a single G4, where competitive binding could influence degradation efficiency, which would contribute to the observed selectivity.

Although it is not clear whether G4L-PROTACs act catalytically or stoichiometrically in cells, our prior observations on the dynamics of PDS binding to G4s in cells suggest the potential for multiple binding events that might lead to a catalytic mode of action^53^.

While our approach was effective in live cells, the G4L-PROTACs required Endo-Porter to achieve intracellular uptake. Future improvements in uptake and delivery—such as tailoring the G4L-PROTAC scaffold to engage endogenous transporters such as CD36—will be required to enable application in animal models or therapeutic settings.

Overall, this study demonstrates a modality for degrading chromatin-associated proteins. By localizing degradation to specific genomic regions defined by structured DNA elements, the method provides a way to perturb nuclear protein function with spatial precision. We anticipate that the general principle will be applicable to other nucleic acid structures, and that further refinement of cell-permeable, structure-guided PROTACs will enhance both their utility as research tools and their potential as therapeutic agents.

## Methods

### Chemical synthesis and characterizations

Detailed synthetic procedures, molecular characterizations, cell viability assays, western blot analyses and general experimental conditions are provided in the Supplementary Information. PDS was synthesized according to previously published protocols^21^.

### Cell culture

Human osteosarcoma U2OS cells (ATCC, HTB-96), cervical carcinoma HeLa (ATCC, CCL-2) and lung adenocarcinoma A549 (ATCC, CCL-185) cell lines were cultured in phenol red-free Dulbecco’s modified Eagle medium (DMEM) (high glucose and l-glutamine plus, Gibco, cat. no. 21063045) supplemented with 10% (v/v) heat-inactivated foetal bovine serum (Gibco, cat. no. 10082147) and 1 mM sodium pyruvate (hereafter referred to as full-growth DMEM). Cells were cultured at 37 °C in a humidified atmosphere containing 5% CO_2_. Regular mycoplasma testing confirmed the absence of contamination.

### SMARCA4 and FUS CUT&Tag

The CUT&Tag protocol was adapted from previously described procedures^54^. In brief, U2OS cells were cultured to 70–80% confluence and treated with 2 μM of G4L-PROTAC for 6 h before collection. To facilitate cellular uptake of the compound, 6 μl of Endo-Porter (Endo-Porter PEG: 1 mM Endo-Porter in 10% polyethylene glycol, 1,500 molecular weight, aqueous) was added per 1 ml of culture medium together with the G4L-PROTAC. Cells were then detached using Accutase (Gibco, cat. no. A1110501), quenched with full-growth DMEM and centrifuged at 200*g* for 5 min. The resulting cell pellets were fixed in 0.1% (w/v) formaldehyde in phosphate-buffered saline (PBS) for 2 min at room temperature and quenched with 110 mM glycine in PBS (pH 7.0). Cells were then centrifuged at 300*g* for 5 min at 4 °C, washed and resuspended in cold wash buffer (20 mM HEPES, pH 7.5, 150 mM KCl, 0.5 mM spermidine, and cOmplete EDTA-free protease inhibitor cocktail; Roche, cat. no. 11836170001). Cells were either used immediately or cryopreserved in wash buffer supplemented with 10% DMSO at −80 °C. A final cell concentration of 6 million cells per millilitre was used for subsequent steps.

Concanavalin A beads (15 μl per sample; Bangs Laboratories, cat. no. BP531) were washed twice with binding buffer (20 mM HEPES, pH 7.5, 10 mM KCl, 1 mM CaCl_2_ and 1 mM MnCl_2_) and resuspended in 10 μl of the same buffer. A 100-μl suspension of cells was combined with 10 μl of prewashed beads in 0.2-ml PCR tubes and mixed on an Intelli Mixer RM-2M (ELMI) using mode C3 (cycle: 6 s at 108°, 6 s at 252°, 12 s pause) at 20 rpm for 10 min at room temperature. Bead-bound cells were gently washed twice with 100 μl wash buffer using a magnetic stand and resuspended in 100 μl of a 1:100 diluted primary antibody solution. Control samples without primary antibody were included. Incubations were carried out on the same mixer at 4 °C overnight.

Unbound primary antibodies were removed by washing three times with 200 μl of Dig-wash buffer (wash buffer with 0.05% digitonin), followed by incubation with 100 μl of guinea pig anti-rabbit IgG secondary antibody (antibodies-online, cat. no. ABIN101961) diluted 1:100 in Dig-wash buffer for 1 h at room temperature with mixing.

The pA–Tn5 adapter complex (2 μM) was prepared as previously described and diluted 1:200 in Dig-300 buffer (20 mM HEPES, pH 7.5, 300 mM KCl, 0.5 mM spermidine, 0.01% digitonin, and protease inhibitors)^42^. Cells were washed three times with 200 μl Dig-wash buffer to remove excess antibody before incubation with 100 μl of the pA–Tn5 complex for 1 h with mixing at room temperature. Subsequently, cells were washed three times in 200 μl Dig-300 buffer to eliminate unbound pA-Tn5 and resuspended in 200 μl of tagmentation buffer (Dig-300 with 10 mM MgCl_2_) for 1 h at 37 °C on the mixers.

After tagmentation, cells were washed twice in 200 μl TAPS buffer (10 mM TAPS and 0.2 mM EDTA), then resuspended in 100 μl extraction buffer (10 mM Tris–HCl, pH 8.0, 0.5 mg ml^−1^ proteinase K (Thermo Scientific, cat. no. EO0491) and 0.5% SDS). Samples were incubated at 55 °C for 1.5 h at 800 rpm. DNA was purified using the DNA Clean & Concentrator-5 kit (Zymo Research, cat. no. D4013) and eluted in 25 μl elution buffer following a 10-min incubation at room temperature.

For library amplification, 21 μl of tagmented DNA was combined with 2 μl each of uniquely barcoded v2 Ad1.x and Ad2.x primers (10 μM each)^55^, and 25 μl of NEBNext Ultra II Q5 2× PCR Master Mix (New England Biolabs, cat. no. M0544) and subjected to PCR amplification with the following program: 72 °C for 5 min; 98 °C for 30 s; followed by 10 cycles of 98 °C for 10 s and 63 °C for 10 s; and a final extension at 72 °C for 1 min. Libraries were purified with 1.3× volume (65 μl) of Ampure XP beads (Beckman Coulter, cat. no. A63882), incubated at room temperature for 10 min, and washed twice with 80% ethanol. DNA was eluted in 25 μl of 10 mM Tris–HCl (pH 8.0).

Library size distribution was verified using a TapeStation automated electrophoresis system (Agilent) with a High Sensitivity D1000 ScreenTape (Agilent, cat. no. 5067-5584), and library concentration quantified using a NEBNext Library Quant Kit (New England Biolabs, cat. no. E7630L). Pooled libraries were size-selected by adding 0.4× volume Ampure XP beads, transferring the supernatant to a new tube and then adding 1.3× volume Ampure XP beads for final clean-up. Beads were washed twice with 80% ethanol, and DNA was eluted in 30 μl of 10 mM Tris–HCl, pH 8.0. Sequencing was performed on an Illumina NextSeq 2000 using the NextSeq 1000/2000 P2 reagent kit (100 cycles, cat. no. 20046811). At least two biological replicates with two technical replicates were performed for each experiment.

### Sample dissolution, TMT labelling and reverse-phase fractionation

Cell pellets were homogenized by pipetting in lysis buffer (1% sodium deoxycholate (Sigma, cat. no. D6750), 100 mM triethylammonium bicarbonate (Sigma, cat. no. T7408), 10% isopropanol, 50 mM NaCl, 1× cocktail protease and phosphatase inhibitors (Thermo Fisher Scientific, cat. no. 78441) + nuclease (Thermo Fisher Scientific, cat. no. 88700) (0.5 μl per 1 ml buffer). Lysates were briefly sonicated to improve sample dissolution. Protein quantification was performed using the Bradford assay (BIO-RAD-Quick start) according to the the manufacturer’s protocol. Seventy micrograms of protein was reduced and alkylated in 5 mM tris-2-carboxymethyl phosphine (Thermo Fisher Scientific, cat. no. 77720) and 10 mM iodoacetamide at room temperature for 1 h in the dark. Then, 100 mM triethylammonium bicarbonate was added to reach pH 8.0, and protein was digested overnight at room temperature in 3-μg trypsin solution (Pierce, cat. no. 90058). Tandem mass tag (TMT) labelling was performed by incubating each sample with 0.5 mg TMTpro-18plex reagents (Thermo Fisher Scientific) for 1 h at room temperature. The reaction was quenched in hydroxylamine (Thermo Scientific, cat. no. 90115) to a final concentration of 0.3% for 15 min. TMT-labelled peptides were combined, and formic acid was added to precipitate out sodium deoxycholate. Peptides were dried in a SpeedVac (Thermo Fisher Scientific). Peptides were reconstituted in 80 μl 20 mM ammonia solution with 0.1% formic acid and fractionated into 42 samples across a 90-min gradient by offline high-pH reverse-phase chromatography (Thermo Fisher Scientific). Samples were pooled orthogonally into 20 (PR1795) or 22 (PR1860) fractions and dried in the SpeedVac. Each fraction was reconstituted in 0.1% formic acid for liquid chromatography–tandem mass spectrometry (LC–MS/MS) analysis below.

### LC–MS/MS

Samples were analysed on an Vanquish Neo UHPLC system (Thermo Fisher Scientific) coupled with an Orbitrap Ascend Mass Spectrometer (Thermo Fisher Scientific). Peptides were trapped on a 300-μm internal diameter (ID) × 5 mm C18 trap cartridge (5 µm, 100 Å) followed by a 100-min elution using 75-μm ID × 50 cm C18 RP column (2 µm, 100 Å) at a 300 nl min^−1^ flow rate. In each data collection cycle, one full MS scan (400–1,600 *m*/*z*) was acquired in the Orbitrap (120k resolution, automatic gain control (AGC) setting of 4 × 10^5^ and maximum injection time (MIT) of 251 ms). A subsequent MS2 analysis was conducted using a top-speed approach with a 3-s cycle time, during which the most abundant ions were fragmented by collision-induced dissociation and analysed in the ion trap. MS2 was performed with a collision energy of 30%, AGC setting of 1 × 10^4^, an isolation window of 0.7 *m*/*z* and a MIT of 35 ms). Previously analysed precursor ions were dynamically excluded for 45 s. MS3 analyses was performed for TMT quantification, whereby precursor ion selection was based on the previous MS2 scan and isolated using a 2.0 Da *m*/*z* window. MS3 was conducted using sequential precursor selection methodology with the top10 settings. Higher-energy collisional dissociation (HCD) was used for MS3, performed with 55% collision energy, and reporter ions were detected using the Orbitrap (45k resolution, AGC setting of 1 × 10^5^ and MIT of 200 ms).

### Data processing

The Proteome Discoverer 3.0. (Thermo Scientific) was used to process tandem mass spectra. The SequestHT search engine was used, and spectra were searched against the UniProt *Homo sapiens* FASTA database (taxon ID 9606, version 2024). All searches were performed using a static modification of TMTpro (+304.207 Da) at any N terminus and lysines, and carbamidomethyl at cysteines (+57.021 Da). Methionine oxidation (+15.9949 Da) and deamidation on asparagine and glutamine (+0.984) were included as dynamic modifications. Mass spectra were searched using precursor ion tolerance 20 ppm and fragment ion tolerance 0.5 Da. For peptide confidence, 1% FDR was applied and peptides uniquely matched to a protein were used for quantification.

### Crystal violet staining and cell density quantification

The protocol was adapted from that previously described^56^. In brief, U2OS cells were seeded at a density of 1,000 cells per well in full-growth DMEM medium in a 6-well plate and grown for 3 weeks. Cells were cultured in the presence of 200 nM compound compared with vehicle DMSO in 1.5 ml full-growth DMEM medium. To facilitate cellular uptake of the compound, 6 μl of Endo-Porter (Endo-Porter PEG: 1 mM Endo-Porter in 10% polyethylene glycol, 1,500 molecular weight, aqueous) was added per 1 ml of culture medium together with compound. Three weeks later, cells were washed with 2 ml PBS and stained with 1 ml crystal violet solution (0.5% crystal violet in 20% methanol) at room temperature for 10 min. Unbound crystal violet was removed by rinsing in distilled water (2 ml 4×), and cells were subsequently air-dried. Cells were visualized on a Bio-Rad ChemiDoc MP system using the Coomassie Blue Gel channel (590/110, white trans) with 0.5 s manual exposure. Image processing and cell density quantification were performed using the open-source software Fiji (version 2.14.0/1.54f)^57^.

### CUT&Tag sequencing data processing

The paired-end raw sequencing reads (PE61) produced by Illumina (Nextseq2000) were demultiplexed using demuxFQ (version 3.1.0, supported by CRUK-CI Genomics) with the following options: -c -d -i -e -t 1 -r 0.01 -R -l 9. The quality of the demultiplexed FASTQ files was assessed using FastQC (version 0.12.1)^58^ and visualized with MultiQC (version 1.18)^59^. Reads from each sample were then trimmed using fastp (version 0.23.4)^60^ with a quality threshold of 25 (-q 25). The trimmed reads were aligned to the reference genome hg38 using Bowtie2 (version 2.5.2)^61^ with the options --end-to-end --very-sensitive --no-mixed --no-discordant --phred33 -I 10 -X 1500. Duplicates were marked using MarkDuplicates from Picard (version 3.1.0)^62^ and subsequently removed with SAMtools (version 1.18)^63^ using the -F 1804 option. Reads mapped to mitochondrial DNA were also removed. After alignment, coordinates of the aligned fragments were generated using the bamtobed option of bedtools (version 2.31.0)^64^. These coordinates were used to generate coverage files in bedGraph format using the genomecov option. The bedGraph files were then used to call peaks with SEACR (version 1.3)^65^ under the options 0.01 non stringent. To visualize the aligned profiles, bamCoverage from deepTools (version 3.5.5)^66^ was used to generate bigWig files from the alignments with options: -bs 10 --normalizeUsing CPM --effectiveGenomeSize 2913022398 --extendReads --minFragmentLength 10 --maxFragmentLength 1000. The compared bigwig files were generated by bamCompare from deepTools with default options.

### Differential binding analysis for CUT&Tag peaks

Peaks called by SEACR together with their corresponding alignment bam files were used to construct peak sets for DiffBind (R package, version 3.12.0)^67^ to identify significantly differential binding sites between G4L-PROTAC treatment and control groups for G4-binding proteins. The plotMA function in DiffBind was used to generate MA plots to visualize the differential binding sites. Significant differential binding sites were defined as peaks with FDR lower than 0.05. Differential analysis were performed using R (version 4.3.2) script.

### Peak overlaps

Reproducible peaks were identified by extracting regions that appeared in at least two out of three technical replicates using the multiinter option in bedtools, followed by overlapping peak regions between two biological replicates to ensure robustness. Any overlap between reliable peaks from G4L-PROTAC-treated and control samples was visualized using the Intervene Python package (version 0.6.5)^68^.

### G4 affinity enrichment and western analysis

U2OS cells were cultured until they reached approximately 80% confluence. Cell pellets were resuspended at a density of 10 million cells per 300 µl in a low-salt buffer (20 mM HEPES, pH 7.4, 10 mM NaCl, 3 mM MgCl_2_, 0.2 mM EDTA and 1 mM dithiothreitol (DTT)) containing protease inhibitor cocktail (PIC) and incubated on ice for 15 min. Subsequently, 15 µl of 10% NP-40 was added, followed by 1 min of vortexing. The samples were centrifuged at 900*g* for 10 min at 4 °C to isolate nuclear pellets, which were then washed with low-salt buffer.

Nuclear pellets were lysed at a density of 30 million cells per 250 µl in a high-salt buffer (20 mM HEPES, pH 7.4, 500 mM NaCl, 3 mM MgCl_2_, 0.2 mM EDTA, 0.5% NP-40 and 1 mM DTT) supplemented with PIC. Lysis was performed using a Diagenode Bioruptor Plus sonicator (ten cycles of 30 s on/30 s off, high setting, 4 °C). Lysates were centrifuged at 16,000*g* for 10 min at 4 °C to collect nuclear proteins, and protein concentration was measured using a bicinchoninic acid assay (Pierce BCA, Thermo Fisher Scientific) assay.

A 50-µl slurry of streptavidin MagneSphere paramagnetic beads (Promega, cat. no. Z5481) was washed three times with 2 ml of pull-down buffer (25 mM HEPES, 10.5 mM NaCl, 110 mM KCl, 1 mM MgCl_2_, 0.01 mM ZnCl_2_, 20% glycerol, 0.1% Igepal C-630, 1 mM DTT and PIC) containing 3% bovine serum albumin (BSA) and 0.2 g l^−1^ salmon sperm DNA (Invitrogen, cat. no. 15632011). For preclearing, 75 µg of nuclear protein was added to 500 µl of pull-down buffer (with 3% BSA and 0.2 g l^−1^ salmon sperm DNA) and incubated with the prewashed beads at 4 °C for 2 h. Meanwhile, a second set of 50 µl beads was washed using the same procedure.

To immobilize oligonucleotides, 50 µl of 10 µM annealed biotinylated oligonucleotides (Sigma-Aldrich) were added to 500 µl of pull-down buffer and incubated with the washed beads at room temperature for 30 min with rotation. Oligonucleotide-coupled beads were then washed three times with 2 ml of pull-down buffer and incubated with 500 µl of precleared lysate at 4 °C overnight with rotation.

Following incubation, beads were washed five times with 500 µl of cold pull-down buffer. Bound proteins were eluted by heating the beads at 70 °C for 10 min in 25 µl of lithium dodecyl sulfate sample buffer containing 50 mM freshly prepared DTT. A 3-µl aliquot of the eluate was analysed via capillary electrophoresis using a ProteinSimple Wes automated western blotting system following the manufacturer’s protocol. Remaining samples were stored at –20 °C. Detection of target bands was performed using primary antibodies (Supplementary Table 4) and anti-rabbit/anti-mouse secondary antibodies, with signal analysis conducted via Compass for SW software (ProteinSimple).

### ELISA

ELISAs for binding affinity and specificity were performed as described previously with minor modifications^16^. In brief, biotinylated oligonucleotides were bound to Pierce streptavidin-coated high-capacity plates (ThermoFisher) followed by blocking with 3% BSA and incubation with full-length recombinant human GST-tagged SOX2 (Abnova, cat. no. H00006657-Q01) and SNRNP70 (Abnova, cat. no. H00006625-P01), in ELISA buffer (100 mM KCl and 50 mM KH_2_PO4, pH 7.4). After three washes with the ELISA buffer, detection was achieved with an anti-GST horseradish-peroxidase-conjugated antibody (Abcam, cat. no. ab3416) diluted to 1:2,000 in an ELISA buffer that contained 3% BSA and 3,3′,5,5′-tetramethylbenzidine ELISA substrate (slow kinetic rate) (Abcam, ab171525). Signal intensity was measured at 450 nm on a SPECTROstar nano microplate reader (BMG Labtech). *K*_d_ values were calculated from binding curves assuming a one-site binding model using GraphPad Prism software, and standard errors of the mean were calculated from three replicates.

### TMT labelled proteomics data analysis

We conducted two TMT labelling experiments, each comprising 18 samples. The first experiment generated proteomic data for treatments with G4L-PROTACs **2**, **5** and **11**, alongside DMSO as negative controls. The second experiment provided proteomic data for treatments with G4L-PROTACs **3**, **6**, **7**, **9** and **10**, as well as DMSO controls. Peptide intensities were analysed using the qPLEXanalyzer R package^69^. Peptide intensities were normalized through median scaling, followed by aggregation into protein intensities and independent rescaling within each group. Differential protein intensities between the G4L-PROTAC treatment and control groups were assessed using the computeDiffStats function. This process estimated log_2_ fold changes and applied multiple testing correction to *P* values using the Benjamini–Hochberg method to control the FDR. Degraded proteins were defined using a combined criterion of log_2_ fold change <–0.585 (equivalent to a ≥1.5-fold decrease) and FDR <0.05. FDR values were estimated using the qPLEX-RIME framework, which integrates variance modelling from the limma approach to enhance robustness, particularly for low-abundance proteins.

### PPI network analysis

PPI networks were constructed using STRING (Search Tool for the Retrieval of Interacting Genes/Proteins) based on proteins that showed at least a one-third decrease in abundance and were significantly downregulated compared with the control group (FDR < 0.05). Subnetworks were identified using the Markov cluster algorithm. Functional annotation of the subnetworks was performed using DAVID (Database for Annotation, Visualization, and Integrated Discovery) to assess biological relevance. The analysis revealed that various biological processes were notably affected, including the extracellular matrix–receptor interaction, signal transduction and regulation of GTPase activity. Pathway and GO enrichment analyses for the filtered protein sets demonstrated consistent enrichments in multiple biological categories.

### Ubiquitinated protein enrichment

U2OS cells were treated with 10 μM of G4L-PROTAC in the presence of 10 μM MG132 (a proteasome inhibitor) for 1 h, 2 h or 4 h before collection. DMSO + MG132 and G4L-PROTAC without MG132 were used as controls to assess both basal and proteasome-inhibited ubiquitination levels. Ubiquitinated proteins were enriched using the Signal-Seeker Ubiquitination Detection Kit (Cytoskeleton, cat. no. BK161), following the manufacturer’s protocol. In brief, after treatment, cells were lysed in BlastR lysis buffer supplemented with protease and deubiquitinase inhibitors, and lysates were clarified and incubated for 2 h at 4 °C with Ubiquitination Affinity Beads provided in the kit. These beads (cat. no. UBA01B) contain covalently crosslinked ubiquitin-binding domains, including tandem ubiquitin-binding entities, designed to capture both monoubiquitinated and polyubiquitinated proteins with high affinity. This broad capture range enables comprehensive profiling of ubiquitination states, which may confer distinct functional consequences on target proteins. After extensive washing to reduce non-specific binding, bound proteins were eluted, denatured and analysed by western blot. Rather than probing with anti-ubiquitin antibodies, membranes were probed with antibodies against specific proteins of interest (for example, SMARCA4, PARP1, SOX2 and SNRNP70) to assess their ubiquitination status under each treatment condition.

### Cell cycle analysis by flow cytometry

U2OS cells were seeded and treated with 10 μM of DMSO, PDS, **G4L-PROTAC11** or **G4L-PROTAC3** for 72 h in the presence of 6 μl ml^−1^ Endo-Porter to facilitate cellular uptake. Following treatment, cells were collected and processed using the Tali Cell Cycle Kit (Invitrogen, cat. no. A10798) according to the manufacturer’s protocol. In brief, cells were washed twice with Dulbecco’s PBS, fixed in ice-cold 70% ethanol dropwise while vortexing gently to prevent clumping, and stored at –20 °C overnight. For staining, fixed cells were washed with Dulbecco’s PBS, pelleted by centrifugation and resuspended in 200 μl of Tali Cell Cycle Solution (containing propidium iodide, RNase A and Triton X-100). Samples were incubated at room temperature for 30 min in the dark and gently vortexed before acquisition. Flow cytometry was performed on a MACSQuant VYB cytometer (Miltenyi Biotec), and data were analysed to determine the distribution of cells in G1, S and G2/M phases based on DNA content.

### Reporting summary

Further information on research design is available in the Nature Portfolio Reporting Summary linked to this article.

## Online content

Any methods, additional references, Nature Portfolio reporting summaries, source data, extended data, supplementary information, acknowledgements, peer review information; details of author contributions and competing interests; and statements of data and code availability are available at 10.1038/s41557-026-02111-y.

## Supplementary information

Supplementary Information Supplementary Figs. 1–29, synthetic procedures, nuclear magnetic resonance spectra and Supplementary Tables 1–9.

Reporting Summary

## Source data

Source Data Fig. 2 Statistical source data.

Source Data Fig. 3 Unprocessed western blots.

Source Data Fig. 4 Statistical source data.

Source Data Fig. 4 Unprocessed image.

Source Data Fig. 5 Statistical source data.

Source Data Fig. 6 Statistical source data.

Source Data Fig. 6 Unprocessed western blots.

Source Data Extended Data Fig. 1 Statistical source data.

Source Data Extended Data Fig. 3 Statistical source data.

Source Data Extended Data Fig. 3 Unprocessed western blots.

Source Data Extended Data Fig. 4 Statistical source data.

Source Data Extended Data Fig. 4 Unprocessed western blots.

Source Data Extended Data Fig. 5 Statistical source data.

**Extended Data Fig. 6:**
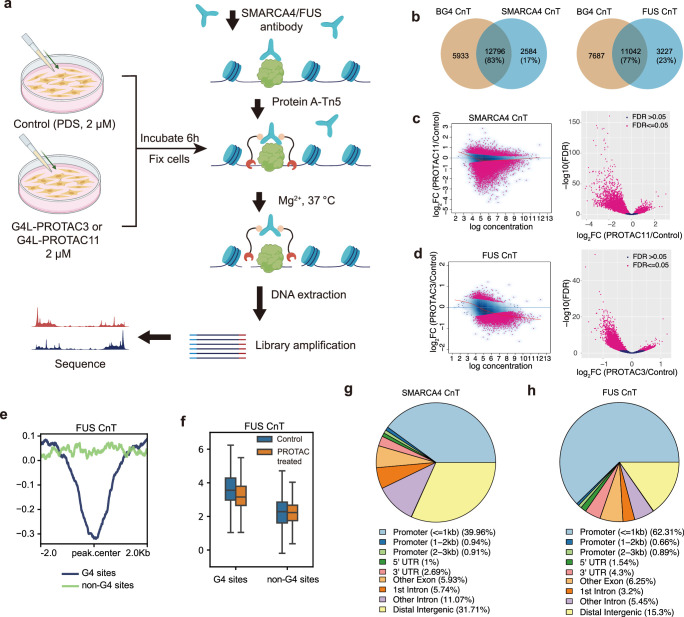
G4L-PROTACs selectively deplete SMARCA4 and FUS from G4 chromatin regions. **a**, Schematic of CUT&Tag to measure chromatin occupancy of SMARCA4 and FUS protein after treatment of U2OS cell with **G4L-PROTAC11** or **G4L-PROTAC3**, respectively (2 µM, 6 h; Endo-proter). **b**, Overlap of SMARCA4 (left) or FUS (right) CUT&Tag peaks with BG4-defined G4 sites in untreated cells. The number of peaks used (SMARCA4 peaks n = 15380, FUS peaks n = 14269, BG4 peaks n = 18729). Data are from three independent biological replicates contributing to peak sets (n = 3). Schematic (including iconography) created with BioRender.com. **c**, **d**, CUT&Tag signal comparisons for SMARCA4 (c) or FUS (d) between G4L-PROTAC-treated and control cells. Scatter plots show log-transformed fold change (log_2_FC) versus signal intensity (red indicates significant changes, FDR < 0.05). Volcano plots (right) showing statistically significant changes in chromatin binding upon G4L-PROTAC treatment (FDR < 0.05). **e**, Average genome-wide binding profile of FUS protein occupancy at G4 versus non-G4 sites, showing preferential signal loss at G4 regions following **G4L-PROTAC3** treatment. Lines show mean across four independent replicates. **f**, Box plots of FUS CUT&Tag signal changes at G4 and non-G4 regions, indicating selective depletion at G4 sites (two-tailed t-test) following **G4L-PROTAC3** (orange) relative to control (blue) treatments. Data are from three independent biological replicates contributing to peak sets (n = 3). Box plots show median (centre line), interquartile range (box, 25^th^–75^th^ percentile), and whiskers (1.5× IQR). Two-sided t-test comparing FUS CUT&Tag signal (log2 CPM) between PDS control and G4L-PROTAC-treated samples at BG4-defined G4 sites and matched non-BG4 sites. BG4 sites: t = 32.133, P = 5.78 × 10^-222. non-BG4 sites: t = 0.285, P = 0.7756. (Positive t indicates higher signal in PDS than G4L-PROTAC.) **g**, **h**, Genomic distribution of down-regulated SMARCA4 (g) or FUS (h) CUT&Tag peaks following G4L-PROTAC treatment. Illustrations in **a** created in BioRender; Wang, Z. https://biorender.com/4h068d6 (2026). Source data

**Extended Data Fig. 7:**
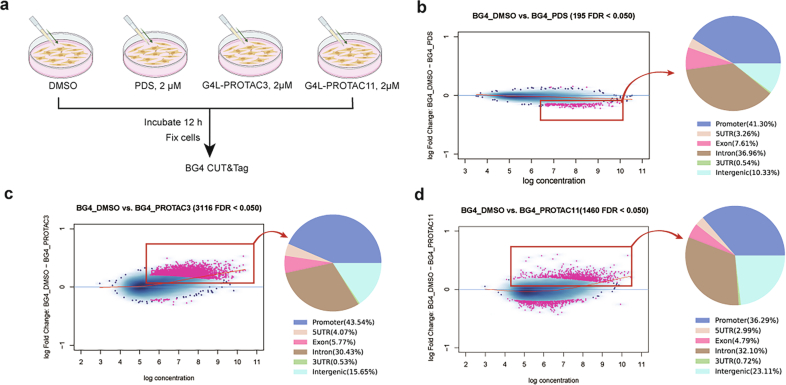
Effects of G4L-PROTAC–induced protein degradation on DNA and chromatin G4 landscapes. **a**, Schematic diagram of BG4 CUT&Tag in U2OS cells treated with DMSO, PDS, or indicated G4L-PROTACs. Schematic (including iconography) created with BioRender.com. **b**–**d**, Genome-wide profiling of G4 structures using BG4 CUT&Tag following 12 h treatment with 2 μM of PDS (**b**), **G4L-PROTAC3** (**c**), or **G4L-PROTAC11** (**d**) compared to DMSO controls. Volcano plots show log2 fold change (x-axis) versus −log10 P value (y-axis) for individual G4 peaks, with significantly reduced peaks (FDR < 0.05) highlighted in pink. The corresponding pie charts display genomic annotations of significant peaks. Illustrations in **a** created in BioRender; Wang, Z. https://biorender.com/4h068d6 (2026).

Source Data Extended Data Fig. 6 Statistical source data.

Source Data Extended Data Fig. 6 Unprocessed image.

**Extended Data Fig. 8:**
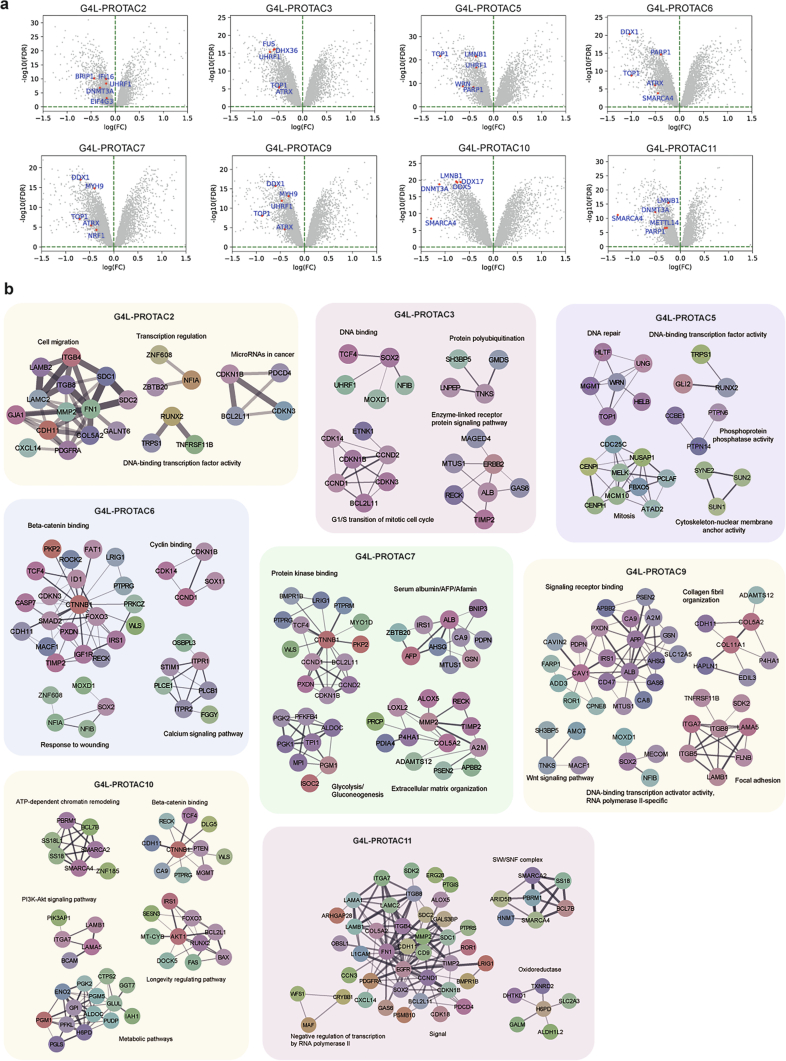
The degradation profiles of G4L-PROTACs in U2OS cells. **a**, Volcano plots showing proteomic changes following treatment of U2Os cells with G4L-PROTACs compared to a negative control based on label-free quantification. Each point represents a protein (x-axis, log_2_ fold change; y-axis, −log_10_ P value), with significantly down-regulated G4-binding proteins (G4BPs) highlighted (FDR < 0.05). **b**, STRING-derived protein–protein interaction networks for significantly down-regulated proteins across selected G4L-PROTAC treatments, showing clustered functional modules (for example, chromatin regulators and transcription factors). Source data

Source Data Extended Data Fig. 8 Statistical source data.

**Extended Data Fig. 9:**
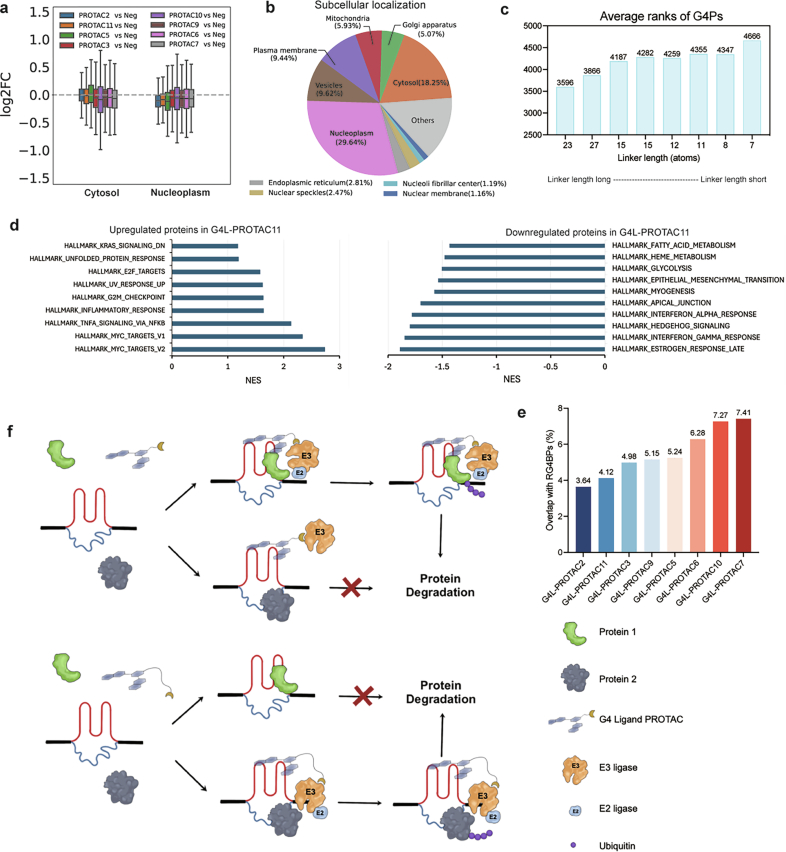
Impact of linker length on the degradation activity and subcellular targeting of G4L-PROTACs. **a**, Box plots of log_2_ fold change in abundance for significantly (FDR < 0.05) downregulated proteins localized to cytosol versus nucleoplasm across eight G4L-PROTACs (relative to a negative control). Box plots show median, IQR, and whiskers (1.5× IQR); n = 3 independent biological replicates per compound. **b**, Pie chart summarizing the sub-cellular localization of proteins significantly down-regulated across 8 G4L-PROTACs (FDR < 0.05). Overall enrichment observed for nucleoplasmic (29.64%) and cytosolic (18.25%) proteins, followed by vesicles, plasma membrane, and other compartments. **c**, Proteomic rank summary for known G4-binding proteins (FDR < 0.05) across G4L-PROTACs with linkers of 7-23 atoms. Bars represent individual biological replicates (n = 3 per compound). **d**, Gene set enrichment analysis (GSEA) of proteomic changes in **G4L-PROTAC2**-treated cells. Up-regulated proteins (left) are enriched for pathways related to DNA damage response and KRAS signalling, while down-regulated proteins (right) include factors involved in metabolism, mesenchymal transition, and interferon response. Normalized enrichment scores (NES) are shown for selected Hallmark gene sets. **e**, Percentage overlap between degraded proteins and a curated RNA G4-binding protein list. **f**, Schematic illustrating how linker length may influence productive proximity between the recruited E3 ligase and proximal G4-associated proteins. Source data

**Extended Data Fig. 10:**
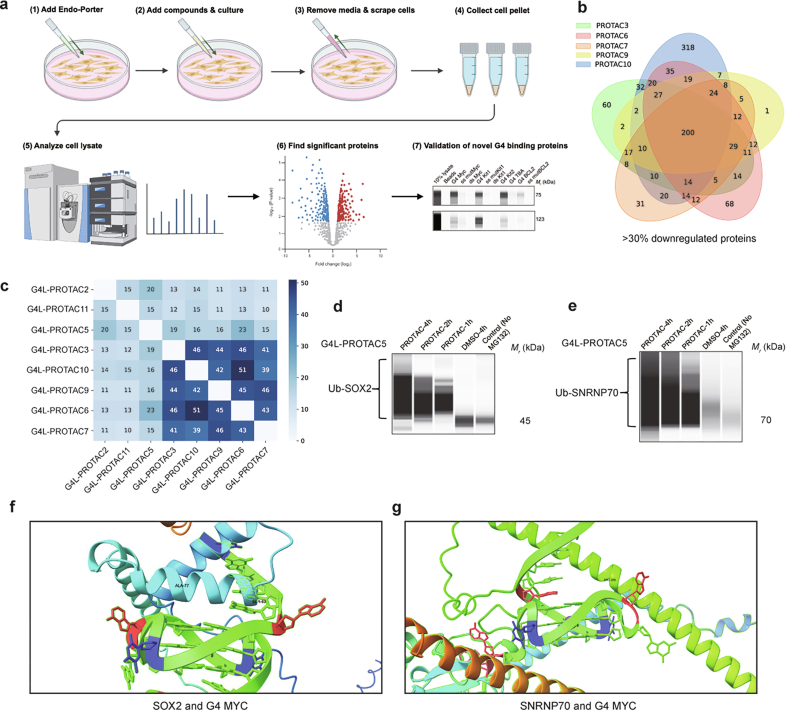
G4L-PROTACs enable identification of additional G4-binding proteins. **a**, Workflow for identifying candidate G4-binding proteins using G4L-PROTACs, quantitative proteomics, and downstream validation by immunoblotting/biochemical assays. Workflow schematic created with BioRender.com. **b**, Venn diagrams showing overlap of proteins down-regulated by >30% across five G4L-PROTACs. Significance threshold: log2 fold-change < –0.585; FDR < 0.05. **c**, Heatmap of pairwise overlap (%) in degraded protein targets across eight G4L-PROTACs. Significance threshold: log2 fold-change < –0.585; FDR < 0.05. **d**, **e**, Ubiquitination assays of SOX2 (**d**) and SNRNP70 (**e**) following G4L-PROTAC treatment, showing enriched ubiquitinated species by immunoblot after affinity enrichment under denaturing conditions. Representative blots are shown from two independent biological experiments. **f**, **g**, Predicted structural models (AlphaFold3) of SOX2–G4 MYC (**f**) and SNRNP70–G4 MYC (**g**) illustrating candidate interaction interfaces. Schematic in **a** created in BioRender; Wang, Z. https://biorender.com/4h068d6 (2026).

Source Data Extended Data Fig. 9 Statistical source data.

## Data Availability

TMT-labelled quantitative proteomics data are provided in the supplementary data file Supplementary_Data_G4L_PROTAC, which contains peptide intensities, metadata and enriched proteins from the G4L-PROTACs versus negative control statistical comparisons. SMARCA4 and FUS CUT&Tag data are available at NCBI GEO (accession number GSE296701). BG4 CUT&Tag data were generated previously and are available under accession number GSE181373. The MS proteomics data have been deposited to the ProteomeXchange Consortium via the PRIDE partner repository with the dataset identifier PXD073248. Source data are provided with this paper.

## Code availability

Details about the bioinformatics data analysis are available via GitHub at https://github.com/sblab-informatics/G4L-PROTACs.
