# Supplementary material for: Degradation of G-quadruplex-binding proteins in chromatin using G4-ligand-based proteolysis-targeting chimeras
Source: Nat Chem. 2026 Mar 19;18(6):1092–101. doi: 10.1038/s41557-026-02111-y (PMC13236602; doi:10.1038/s41557-026-02111-y)
Supplement: Supplementary file 1 — Supplementary Figs. 1–29, synthetic procedures, nuclear magnetic resonance spectra and Supplementary Tables 1–9. [file 41557_2026_2111_MOESM1_ESM.pdf]

nature chemistry

Supplementary information https://doi.org/10.1038/s41557-026-02111-y

Degradation of G-quadruplex-binding proteins in chromatin using G4-ligand-based proteolysis-targeting chimeras

In the format provided by the authors and unedited

Table of contents

General information 3

Experimental methods 3

Supplementary Table 1. 7

Supplementary Table 2. 8

Supplementary Table 3. 9

Supplementary Table 4. 10

Supplementary Table 5. 11

Supplementary Table 6. 12

Supplementary Table 7. 13

Supplementary Table 8.. 14

Supplementary Table 9. 15

Synthetic procedures 16

NMR spectra 31

Supplementary Figure 1 56

Supplementary Figure 2 57

Supplementary Figure 3 58

Supplementary Figure 4 59

Supplementary Figure 5 60

Supplementary Figure 6 61

Supplementary Figure 7 62

Supplementary Figure 8 63

Supplementary Figure 9 64

Supplementary Figure 10 65

Supplementary Figure 11 66

Supplementary Figure 12 67

Supplementary Figure 13 68

Supplementary Figure 14 69
Supplementary Figure 15 70
Supplementary Figure 16 71
Supplementary Figure 17 72
Supplementary Figure 18 73
Supplementary Figure 19 75
Supplementary Figure 20 77
Supplementary Figure 21 79
Supplementary Figure 22 80
Supplementary Figure 23 81
Supplementary Figure 24 83
Supplementary Figure 25 85
Supplementary Figure 26 87
Supplementary Figure 27 91
Supplementary Figure 28 92
Supplementary Figure 29 93
Source Data: Supplementary Figure 29 95
References 96

General information

All chemical reagents were used as received from commercial suppliers unless otherwise stated. Organic solvents were either purchased as anhydrous or purified by standard distillation methods prior to use. Reactions were carried out in oven-dried glassware under an argon atmosphere unless otherwise specified. NMR spectra were recorded on either a Bruker 400 MHz Avance III HD spectrometer or a 500 MHz DCH Cryoprobe spectrometer, operating at 400 and 500 MHz for ^{1}H NMR, and at 100 and 125 MHz for ^{13}C NMR, respectively. Spectra were processed using MestReNova (v12.0.1). Chemical shifts (δ) are reported in parts per million (ppm) relative to residual solvent signals. Multiplicities are denoted as follows: s = singlet, d = doublet, t = triplet, q = quartet, m = multiplet, br = broad, and coupling constants (J) are reported in Hz. LC-MS analyses were performed using an Amazon ESI-MS (Bruker) connected to a Dionex UltiMate 3000 UHPLC system (Thermo Fisher Scientific). High-resolution mass spectrometry (HRMS) data were acquired on a Waters LCT Premier ESI spectrometer. Flash column chromatography was performed on a CombiFlash NEXTGEN 300+ system (Teledyne ISCO) using RediSep normal-phase silica columns. Reversed-phase flash chromatography was carried out on an Interchim puriFlash 4250 system equipped with a C18 column (catalog no. PF-15C18HP-F0025; 15 μm particle size). The synthesis of di-Boc protected PDS (**S1**) was performed as previously[1].

Experimental methods

Oligonucleotide annealing. HPLC purified oligonucleotides were purchased from Sigma-Aldrich unless otherwise stated. For G4s or the single-strand mutants, the indicated concentration of oligonucleotides in 10 mM Tris HCl, pH 7.4, 100 mM KCl were annealed at 95 °C for 5 min followed by gradually cooling to 20 °C. For double-stranded DNA, forward and reverse strand oligonucleotides were mixed by 1:1 and annealed in 10 mM Tris HCl, pH 7.4, 100 mM NaCl in the same way.

FRET melting assay. Oligonucleotides (see Supplementary Table 1) were purchased from Biomers. 400 nM oligonucleotides were annealed in FRET buffer (60 mM potassium cacodylate, pH = 7.4) at 95 °C for 5 min followed by gradually cooling to 20 °C. A series of probe concentrations were prepared in a 96-well plate: 150 μL of 6 μM ligand in FRET buffer

was prepared as the initial concentration. Subsequent serial dilutions were made by adding 100 µL of probe solutions to 50 µL of FRET buffer, resulting in 12 concentrations including a no-probe control. 25 µL per solutions were transferred to another 96-well plate, followed by adding 25 µL of annealed oligonucleotide solutions to each well. The plate was then sealed with an adhesive transparent cover and shaken gently for 10 min. Measurements of restoring FAM signal were recorded on a Bio-Rad CFX96 Touch Real-Time PCR Detection System by ramping from 25°C to 95°C at 0.5°C/min. Melting temperatures were determined by the first-derivative maxima of relative fluorescence unit (RFU) value against time, and ΔT_m was calculated by baseline correction of melting temperatures subtracting no-probe control. A one-site binding model in GraphPad Prism 7 was utilised to fit FRET curves. Mean was calculated from two replicates.

Fluorescence quench binding assay. The protocol was adapted from that described previously[3]. Oligonucleotides (Supplementary Table 2) were purchased from Biomers. 10 nM oligonucleotides in assay buffer (50 mM Tris HCl, pH 7.2, 150 mM KCl, 0.5 w/v % CHAPS, 0.05 v/v % Triton X-100) were annealed. A series of probe dilutions were prepared in a 96-well plate: 100 µL of 50 µM probe in 0.1 v/v % DMSO in water was prepared as the initial concentration. Subsequent serial dilutions were made by adding 50 µL of ligand solutions to 50 µL of 0.1 v/v % DMSO in water, resulting in a total of 12 concentrations including a no-probe control. 10 µL per solution of the series of dilutions were transferred to another 96-well plate, followed by adding 90 µL of the annealed oligonucleotide solution to each well to afford the final concentration of 9 nM. The plate was sealed with an adhesive foil cover and gently shaken for 2 h. End-point fluorescence was then measured on a fluorescence plate reader (BMG PHERAstar Plus). Differences in RFU were converted to absolute values relative to the no-probe control. The range of observed absolute RFU values were normalized as fraction bound. Dissociation constants (K_d) were calculated assuming a one-site binding model using GraphPad Prism 7. Standard deviations (S.D.) were calculated from four replicates.

Growth inhibition assay. U2OS, A549 and HeLa cells were seeded in 96-well white-wall plates (Corning) at a density of 2,000–3,000 cells per well in 100 µl complete growth medium and grown at 37 °C for 20–24 h. Prior to compound treatment, baseline luminescence (T_0) was measured in a separate assay plate using the CellTiter-Glo 2.0 cell viability assay (Promega, catalogue no. G9242). CellTiter-Glo reagent was added 1:1 to the medium (100 µl reagent per

well), mixed for 2 min on a shaker, incubated for 20 min at room temperature, and end-point luminescence was recorded on a PHERAstar FS plate reader. Cells were treated with 0.006–50 µM of PDS, G4L-PROTACs (14-point, 2-fold serial dilution), or with DMSO as vehicle control (final DMSO ≤0.2%). Cells were incubated with compounds for 96 h at 37 °C in a humidified atmosphere containing 5% CO_2. Cell viability was determined using the CellTiter-Glo 2.0 cell viability assay (Promega, catalogue no. G9242) according to the manufacturer's instructions. Briefly, 100 µl CellTiter-Glo reagent was added per well, plates were shaken for 2 min to induce lysis and incubated for 10 min at room temperature before measuring luminescence on a PHERAstar FS. Luminescence values were background-subtracted (media-only wells) and normalized to DMSO-treated control wells. Dose–response curves were fitted using the log(inhibitor) vs. response – variable slope model in GraphPad Prism 10, and GI_{50} values were calculated from the fitted curves. Results are reported as the mean from three biological replicates (n = 3).

Protein extraction and Western blot analysis. U2OS cells were seeded at 50,000 cells/well in 6-well plates using full growth DMEM media and cultured for 24 h. Cells were then treated with G4L-PROTACs compound or vehicle DMSO in 1.5 mL of full growth media for 72 h. Endo-Proter was pre-mixed with media at a ratio of 6 µl/1 ml. After treatment, cells were washed with PBS, detached with Accutase (Gibco, catalogue no. A1110501), and cell pellets were collected. Following another cold PBS wash, cells were lysed in RIPA buffer (Thermo Scientific, catalogue no. 89900) with protease inhibitor cocktail (ThermoFisher, catalogue no. 78438) and phosphatase inhibitor cocktail 3 (Sigma Aldrich, catalogue no. P0044). Lysates were sonicated using a Diagenode Bioruptor Plus (5 cycles: 30 s ON, 30 s OFF, high setting, 4°C), then centrifuged at 14,000 g for 15 min at 4°C to collect the supernatant as protein extracts. Protein concentrations were measured using the BCA assay, and samples were analysed by capillary electrophoresis on a Jess automated Western blot system (ProteinSimple) following the manufacturer's protocol (https://www.bio-techne.com/instruments/simple-western).

Protein-protein interaction networks. Proteins exhibiting a log2 fold change less than -0.585 (indicating degradation with a log2 fold greater than 0.585) were chosen for protein-protein interaction analysis for each PROTAC. These selected proteins were then submitted to the STRING webserver (https://string-db.org/) to identify interaction networks. Subsequently, the identified networks were imported into Cytoscape[2] for detailed analysis and visualization.

During this process, singleton nodes were excluded, and a minimum degree (in + out) of 5 was established to refine the network.

Colocalization analysis. Colocalization between EGFP and RFP signals was quantified using Manders' overlap coefficients (M1 and M2). For each cell, background-subtracted fluorescence intensity values were extracted from confocal images separately for nuclear and cytoplasmic regions. Manders' coefficients were then calculated as the fraction of the EGFP signal that overlaps with RFP (M1) and the fraction of the RFP signal that overlaps with EGFP (M2), using the standard definitions: $M1 = \Sigma(EGFP\_coloc) / \Sigma(EGFP\_total)$ and $M2 = \Sigma(RFP\_coloc) / \Sigma(RFP\_total)$, where coloc denotes pixels in which the intensity of the other channel is >0. Coefficients were computed for the entire dataset across all cells, yielding M1 and M2 values for both nuclear and cytoplasmic compartments.

Supplementary Table 1. DNA oligonucleotides for FRET melting assay

Oligomer	Sequence (5' to 3')	Source	Ref
G4 Kit1	FAM-**GGG**A**GGG**CGCT**GGG**AGGA**GGG**-TAMRA	Biomers	(3)
G4 Myc	FAM-TGA**GGG**T**GGG**TA**GGG**T**GGG**TAA-TAMRA	Biomers	(3)
G4 Telo	FAM-**GGG**TTA**GGG**TTA**GGG**TTA**GGG**-TAMRA	Biomers	(3)
dsDNA	FAM-TATAGCTATA-HEG-TATAGCTATA-TAMRA	Biomers	(3)

FAM = fluorescein; TAMRA = tetramethylrhodamine; HEG = [(-CH_2CH_2O-)$_6$].

Supplementary Table 2. DNA oligonucleotides for fluorescence quench binding assay

Oligomer	Sequence (from 5' to 3')	Source	Ref
G4 Telo	**Cy5**-A**GGG**TTA**GGG**TTA**GGG**TTA**GGG**T	Biomers	(4)
G4 Kit1	**Cy5**-A**GGG**A**GGG**CGCT**GGG**AGGA**GGG**	Biomers	(4)
G4 Myc	T**GGGG**A**GGG**T**GGGG**A**GGG**T**GGGG**AAGG-**Cy5**	Biomers	(4)
dsDNA	**Cy5**-CAATCGGATCGAATTCGATCCGATTG	Biomers	(4)

Cy5 = cyanine 5.

Supplementary Table 3. DNA oligonucleotides for G4 pull-down

Oligomer	Sequence (5' to 3')	Source	Ref
G4 Myc	TGA**GGG**T**GGG**TA**GGG**T**GGG**TAATTTTT[TgBtn]	Sigma-Aldrich	(*5*)
G4 Kit1	A**GGG**A**GGG**CGCT**GGG**AGGA**GGG**TTTTT[TgBtn]	Sigma-Aldrich	(*6*)
ss mutMyc	TGA**GTG**T**GTG**TA**GTG**T**GTG**TAATTTTT[TgBtn]	Sigma-Aldrich	-
ds Myc	TGA**GGG**T**GGG**TA**GGG**T**GGG**TAATTTTT[TgBtn]<br>TTACCCACCCTACCCACCCTCA	Sigma-Aldrich	-
ss mutKit1	A**GTG**A**GTG**CGCT**GTG**AGGA**GTG**TTTTT[TgBtn]	Sigma-Aldrich	-
ds Kit1	A**GGG**A**GGG**CGCT**GGG**AGGA**GGG**TTTTT[TgBtn]<br>CCCTCCTCCCAGCGCCCTCCCT	Sigma-Aldrich	-

Btn = biotin; Tg = tetraethylene glycol.

Supplementary Table 4. Antibodies for western blot and CUT&Tag analysis

**Antibodies**	**Species**	**Source**	**Cat#**
FUS	mouse	Santa Cruz	sc-47711
GAPDH	rabbit	Cell Signaling	D16H11
β-Actin	rabbit	Cell Signaling	4970
SMARCA4	rabbit	abcam	ab110641
PARP1	rabbit	Cell Signaling	9532
γH2AX	rabbit	Cell Signaling	2577
Ubiquitin	rabbit	abcam	ab70462
SOX2	rabbit	Merck	AB5603
SNRNP70	rabbit	abcam	ab83306

Supplementary Table 5. Oligonucleotides for ELISA

Oligomer	Sequence (5' to 3')	Source	Ref
G4 Myc	[Btn]TGA**GGG**T**GGG**TA**GGG**T**GGG**TAA	Sigma-Aldrich	(7)
G4 Kit1	[Btn]A**GGG**A**GGG**CGCT**GGG**AGGA**GGG**	Sigma-Aldrich	(7)
ss mutMyc	[Btn]TGA**GTG**T**GTG**TA**GTG**T**GTG**TAA	Sigma-Aldrich	-
ds Myc	[Btn]TGA**GGG**T**GGG**TA**GGG**T**GGG**TAA	Sigma-Aldrich	-
	TTACCCACCCTACCCACCCTCA		
ss mutKit1	A**GTG**A**GTG**CGCT**GTG**AGGA**GTG**	Sigma-Aldrich	-
ds Kit1	[Btn]A**GGG**A**GGG**CGCT**GGG**AGGA**GGG**	Sigma-Aldrich	-
	CCCTCCTCCCAGCGCCCTCCCT		

Btn = biotin.

Supplementary Table 6. Linker structure, length and E3 ligand of G4L-PROTACs.

#	Linker structure	Linker length	E3 ligand
1	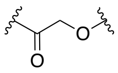	3	Thalidomide
2	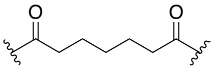	7	VH032
3	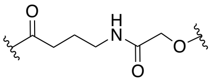	8	Thalidomide
4	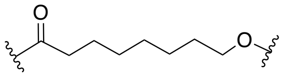	9	Thalidomide
5	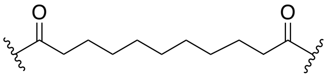	11	VH032
6	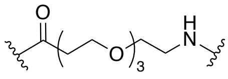	12	Pomalidomide
7	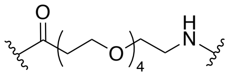	15	Pomalidomide
8	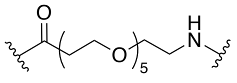	18	Pomalidomide
9	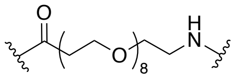	27	Pomalidomide
10	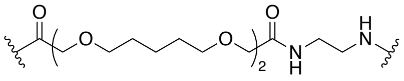	23	Pomalidomide
11	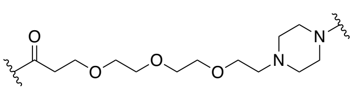	15	Thalidomide
12	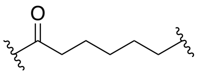	6	Phthalimide
13*	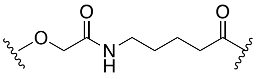	9	Thalidomide
14*	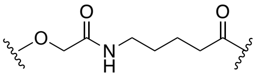	9	VH032

*The G4 ligand for G4L-PROTACs (**13-14**) are pyPDS.

Supplementary Table 7. Telomere-associated proteins in the proteomic dataset and their fold-change upon G4L-PROTAC treatment.

Gene Symbol	G4L-PROTAC 2	G4L-PROTAC 3	G4L-PROTAC 5	G4L-PROTAC 6	G4L-PROTAC 7	G4L-PROTAC 9	G4L-PROTAC10	G4L-PROTAC 11	Function/Association
TERF1	0.86	1.3	0.82	1.37	1.39	1.42	1.37	0.94	Shelterin component, binds telomeric DNA
TERF2	0.97	1.05	0.96	1.04	1.04	1.06	1.07	0.94	Shelterin component, protects telomere ends
POT1	0.86	0.97	0.97	0.99	0.93	0.99	1.01	0.98	Shelterin component, binds telomeric DNA to regulate telomere length
TERF2IP	1.05	1.2	1.04	1.15	1.16	1.21	1.19	1.01	Shelterin-associated, interacts with TERF2
TINF2	0.9	1.18	0.98	1.24	1.16	1.31	1.39	0.89	Shelterin component, links TRF1/TRF2 with POT1
ACD	0.91	0.89	0.95	0.97	1.05	1.04	1.20	0.97	Shelterin component, recruits telomerase
CTC1	0.95	0.97	1.08	1.16	0.91	1.01	1.13	1.00	CST complex, telomere replication
STN1	1.13	0.91	1.22	1.16	1.09	1.11	1.02	0.93	CST complex, telomere replication
TELO2	1.13	0.94	1.10	1.05	1.04	0.96	0.84	1.09	PIKK stabilizer, involved in telomere maintenance
HMBOX1	0.96	0.77	1.09	0.74	0.91	0.86	1.21	0.93	Binds telomeric repeats, regulates transcription
RPA2	1.00	0.99	1.04	1.06	1.03	1.05	1.06	1.07	Lagging strand synthesis at telomeres
ZBTB10	0.97	1.12	1.00	0.91	1.08	1.01	1.31	0.97	TERRA repressor, telomeric chromatin regulator

Supplementary Table 8. GFP and RFP signal quantification.

Object No	Average Nucleus EGFP	Average Cytoplasm EGFP	Nuclei percentage
1	5495.3	3155.6	64%
2	5204.3	2809.3	65%
3	6142.3	3347.1	65%
4	5535.0	3233.9	63%
5	5890.4	3550.1	62%
6	5422.9	3741.9	59%
7	5666.0	3975.7	59%
8	5681.2	4126.2	58%
9	5715.6	4337.7	57%
10	5268.4	4173.2	56%
11	5132.5	4116.2	55%
12	4558.5	4058.3	53%
13	5323.4	4418.8	55%
14	5375.0	4373.2	55%
15	5129.6	4317.8	54%
16	5414.8	4393.6	55%
17	5409.0	4198.4	56%
18	5517.3	4473.6	55%
19	5051.5	3916.2	56%
20	5082.5	3994.3	56%
21	5419.6	4154.0	57%
22	4196.8	3807.5	52%
23	4340.6	3605.9	55%
24	3886.9	3472.1	53%
25	5325.5	4253.2	56%
26	4727.1	3843.6	55%
27	5061.4	3649.7	58%
28	4296.7	3462.4	55%
29	2319.1	2735.7	46%
30	2785.3	2857.6	49%
31	3115.8	2881.2	52%
32	3462.1	3549.8	49%
33	2687.3	2571.2	51%
34	2531.4	3059.0	45%
35	3494.6	3202.7	52%
36	2185.0	2144.6	50%
37	2406.7	2494.3	49%
38	2828.0	2706.6	51%
39	2555.7	2424.9	51%
40	1283.0	1309.8	49%
41	1827.0	1700.7	52%
42	1518.2	1511.1	50%
43	2100.8	2299.8	48%
44	3536.0	2661.7	57%
45	1511.6	1558.8	49%
46	2755.9	2752.0	50%
47	3369.3	2920.9	54%
48	2305.3	2861.5	45%

Supplementary Table 9. Summary of rG4BPs degraded by each G4L-PROTAC.

G4L-PROTAC	Representative degraded proteins
**2**	RABGAP1L, NDUFS8, HNRNPUL1, AMOT, POLE, PTPN14
**3**	FUS, RABGAP1L, PTGFRN, MAP7, GPNMB, EDIL3, HNRNPUL1, AMOT, VPS13C, OSBPL3, PTPN14, EXOC4
**5**	RABGAP1L, SUN2, QKI, ASF1A, CHD2, EDIL3, HNRNPUL1, AMOT, LRBA, GPI, CHD3, PTPN14
**6**	DLG1, CADM1, GPNMB, TRAM2, CTNNB1, RABGAP1L, SRPK2, PTGFRN, AMOT, VPS13C, EDIL3, MACF1, OSBPL3, MYO1D
**7**	DLG1, CADM1, GPNMB, MSI2, CTNNB1, PTGFRN, AMOT, VPS13C, EDIL3, MACF1, OSBPL3, MYO1D, PTPN14, DST
**9**	DLG1, MYO1D, RABGAP1L, PTGFRN, MAP7, GPNMB, EDIL3, PBXIP1, AMOT, VPS13C, MACF1, OSBPL3
**10**	SMARCA4, PBRM1, HNRNPUL1, GPNMB, CTNNB1, RABGAP1L, AMOT, VPS13C, OSBPL3, MAP7, PBXIP1, PTPN14
**11**	SMARCA4, PBRM1, HNRNPUL1, EDIL3, JUP, LGALS3BP

Synthetic procedures

Synthesis of G4L-PROTACs

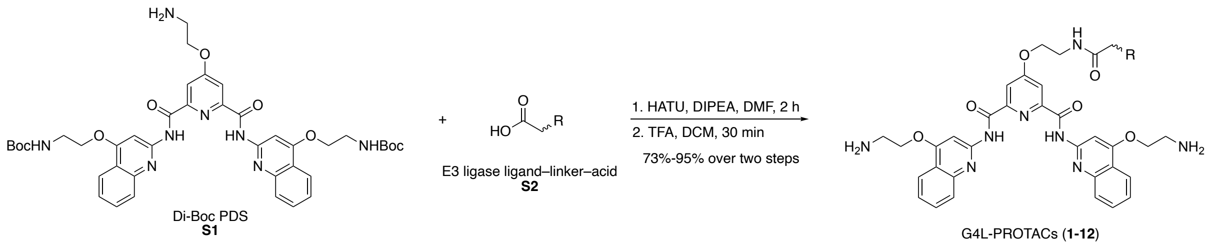

General procedure: The synthesis of di-Boc protected PDS (**S1**) was performed as previously[1]. E3 ligase ligand–linker–acid (**S2**, 30 µmol) and HATU (11.4 mg, 30 µmol) were dissolved in 0.5 mL anhydrous DMF. Di-Boc protected PDS (**S1**, 20 mg, 25 µmol) was added to the mixture followed by the addition of DIPEA (23 µL, 125 µmol), and the reaction was stirred for 2 h in room temperature under argon. After completion, the solvent was removed under reduced pressure. The resulting crude material was dissolved in 1 mL of 30% TFA in DCM (v/v) and stirred at room temperature for 30 minutes. The solvent was then evaporated to dryness under reduced pressure. The crude product was purified by reversed-phase flash column chromatography using a C18 column, with a gradient elution from water (containing 0.1% TFA, v/v) to acetonitrile (containing 0.1% TFA, v/v) over 30 minutes at a flow rate of 18 mL/min. Solvents were removed by lyophilization to yield the desired G4L-PROTACs (1–12) as off-white to yellow powders (yield: 73–95%).

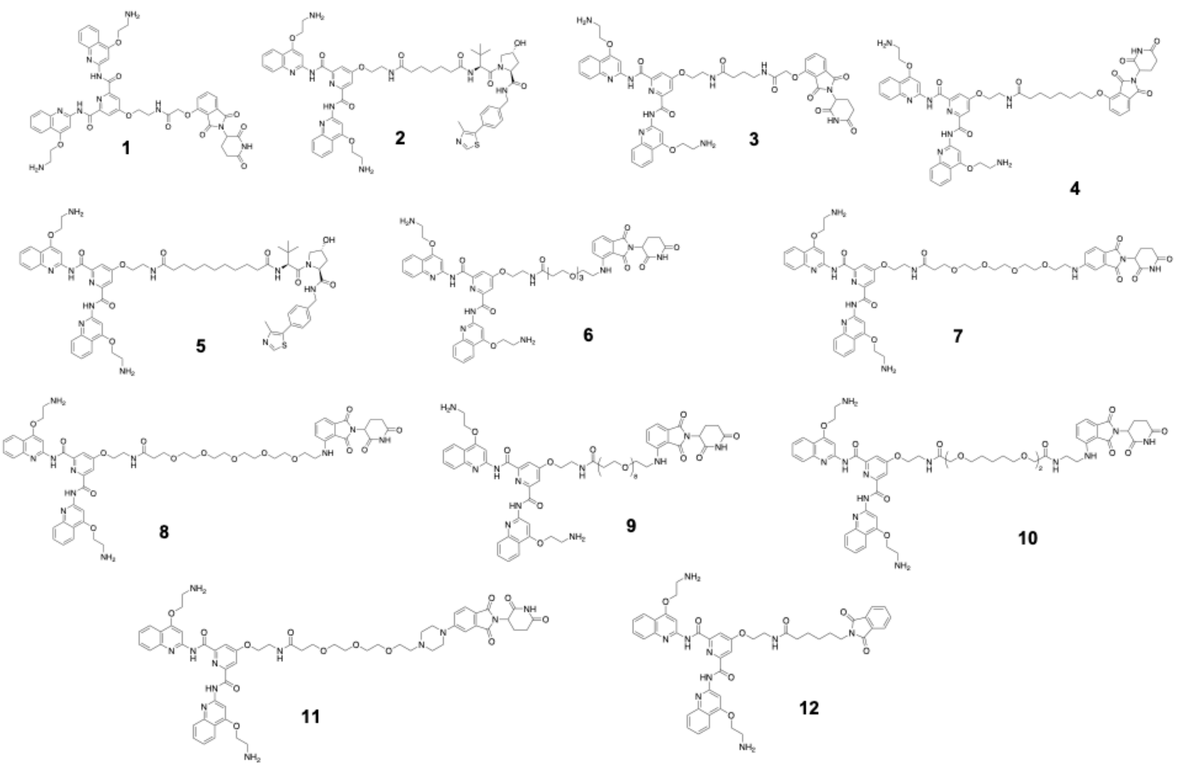

G4L-PROTAC1

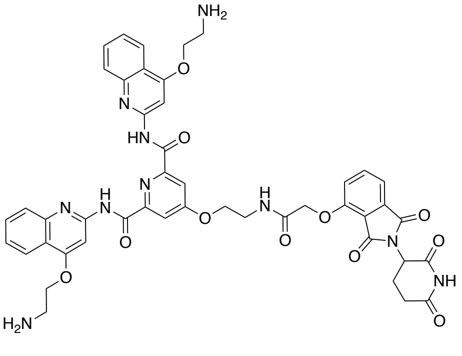

(N^2,N^6-bis(4-(2-aminoethoxy)quinolin-2-yl)-4-(2-(2-((2-(2,6-dioxopiperidin-3-yl)-1,3-dioxoisoindolin-4-yl)oxy)acetamido)ethoxy)pyridine-2,6-dicarboxamide): Thalidomide-alkyl3-COOH (10.5 mg, 30 µmol) and HATU (11.4 mg, 30 µmol) were dissolved in 0.5 mL anhydrous DMF. Di-Boc protected PDS (**S1**, 14 mg, 25 µmol) was added to the mixture followed by the addition of DIEA (23 µL, 125 µmol), and the reaction was stirred for 2 h in room temperature under argon. After completion, the solvent was removed under reduced pressure. The resulting crude material was dissolved in 1 mL of 30% TFA in DCM (v/v) and stirred at room temperature for 30 minutes. The solvent was then evaporated to dryness under reduced pressure. The crude product was purified by reversed-phase flash column chromatography using a C18 column, with a gradient elution from water (containing 0.1% TFA, v/v) to acetonitrile (containing 0.1% TFA, v/v) over 30 minutes at a flow rate of 18 mL/min.

Solvents were removed through freeze-drying to obtain G4L-PROTAC1 as a white powder (18.2 mg, 80% yield). ^{1}H NMR (400 MHz, DMSO-d_6) δ 12.09 (s, 2H), 11.11 (s, 1H), 8.46 (d, J = 8.0 Hz, 2H), 8.29 (s, 1H), 8.19 (s, 6H), 8.10 (s, 2H), 7.96 (d, J = 7.7 Hz, 4H), 7.86 – 7.74 (m, 3H), 7.57 (t, J = 7.9 Hz, 2H), 7.45 (dd, J = 20.4, 7.9 Hz, 2H), 5.13 (d, J = 18.2 Hz, 2H), 4.86 (s, 3H), 4.52 (s, 4H), 4.41 (s, 2H), 3.68 (s, 2H), 3.48 (s, 4H), 2.90 (s, 1H), 2.05 (s, 1H), 1.76 (s, 2H), 1.24 (s, 1H); HRMS (ESI-TOF): $[M+H]^+$ calculated for $C_{46}H_{43}N_{10}O_{11}$: 911.3107, found: 911.3128.

G4L-PROTAC2

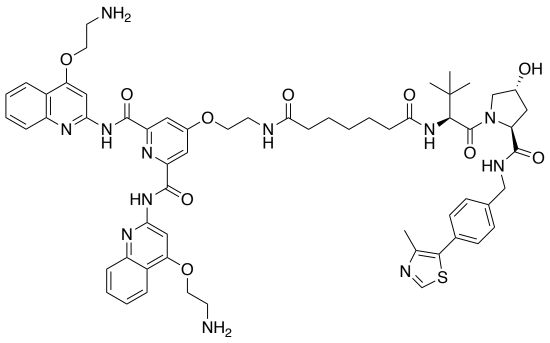

***N*^{2},*N*^{6}-bis(4-(2-aminoethoxy)quinolin-2-yl)-4-(2-(7-(((*S*)-1-((2*S*,4*R*)-4-hydroxy-2-((4-(4-methylthiazol-5-yl)benzyl)carbamoyl)pyrrolidin-1-yl)-3,3-dimethyl-1-oxobutan-2-yl)amino)-7-oxoheptanamido)ethoxy)pyridine-2,6-dicarboxamide:** VHL-alkyl7-COOH (15.4 mg, 30 µmol) and HATU (11.4 mg, 30 µmol) were dissolved in 0.5 mL anhydrous DMF. Di-Boc protected PDS (**S1**, 20 mg, 25 µmol) was added to the mixture followed by the addition of DIPEA (23 µL, 125 µmol), and the reaction was stirred for 2 h in room temperature under argon. After completion, the solvent was removed under reduced pressure. The resulting crude material was dissolved in 1 mL of 30% TFA in DCM (v/v) and stirred at room temperature for 30 minutes. The solvent was then evaporated to dryness under reduced pressure. The crude product was purified by reversed-phase flash column chromatography using a C18 column, with a gradient elution from water (containing 0.1% TFA, v/v) to acetonitrile (containing 0.1% TFA, v/v) over 30 minutes at a flow rate of 18 mL/min. Solvents were removed by lyophilization to yield the desired G4L-PROTAC2 as white powder (23.6 mg, yield: 82%). ^{1}H NMR (500 MHz, Methanol-d_4) δ 8.90 (s, 1H), 8.52 (d, J = 8.8 Hz, 2H), 8.24 (s, 2H), 8.16 (s, 2H), 8.09 (d, J = 8.5 Hz, 2H), 7.97 (t, J = 7.7 Hz, 2H), 7.74 – 7.69 (m, 3H), 7.46 (d, J = 8.1 Hz, 2H), 7.40 (d, J = 8.3 Hz, 3H), 4.78 (t, J = 4.9 Hz, 5H), 4.62 (s, 1H), 4.58 – 4.53 (m, 2H), 4.50 (d, J = 12.0 Hz, 2H), 4.44 (t, J = 5.4 Hz, 2H), 4.36 (d, J = 15.4 Hz, 2H), 2.46 (s, 4H), 2.34 – 2.19 (m, 7H), 2.08 (ddd, J = 13.3, 9.1, 4.5 Hz, 2H), 1.65 (dp, J = 14.5, 7.3 Hz, 6H), 1.37 (p, J

= 7.7 Hz, 3H), 1.02 (s, 11H); HRMS (ESI-TOF): $[M+H]^+$ calculated for $C_{60}H_{71}N_{12}O_{10}S$: 1151.5131, found: 1151.5122.

G4L-PROTAC3

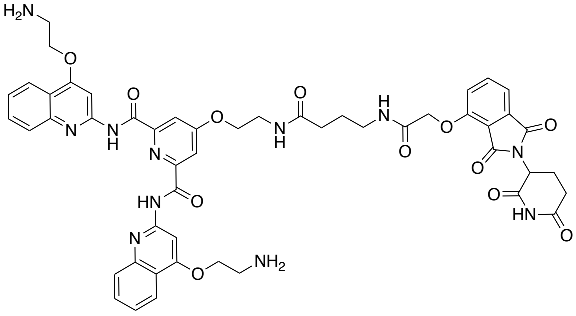

***N*²,*N*⁶-bis(4-(2-aminoethoxy)quinolin-2-yl)-4-(2-(4-(2-((2-(2,6-dioxopiperidin-3-yl)-1,3-dioxoisoindolin-4-yl)oxy)acetamido)butanamido)ethoxy)pyridine-2,6-dicarboxamide:** Thalidomide-C7-COOH (12.8 mg, 30 μmol) and HATU (11.4 mg, 30 μmol) were dissolved in 0.5 mL anhydrous DMF. Di-Boc protected PDS (**S1**, 20 mg, 25 μmol) was added to the mixture followed by the addition of DIPEA (23 μL, 125 μmol), and the reaction was stirred for 2 h in room temperature under argon. After completion, the solvent was removed under reduced pressure. The resulting crude material was dissolved in 1 mL of 30% TFA in DCM (v/v) and stirred at room temperature for 30 minutes. The solvent was then evaporated to dryness under reduced pressure. The crude product was purified by reversed-phase flash column chromatography using a C18 column, with a gradient elution from water (containing 0.1% TFA, v/v) to acetonitrile (containing 0.1% TFA, v/v) over 30 minutes at a flow rate of 18 mL/min. Solvents were removed by lyophilization to yield the desired G4L-PROTAC3 as white powder (yield: 78%). ^{1}H NMR (400 MHz MeOH-d_4): δ 8.51 (d, *J* = 8.0 Hz, 2H), 8.18 (s, 2H), 8.13 (s, 2H), 8.08 (d, *J* = 8.4, 2H), 7.96 (t, *J* = 7.8 Hz, 2H), 7.73 – 7.66 (m, 3H), 7.44 (d, *J* = 7.2 Hz, 1H), 7.35 (d, *J* = 8.4 Hz, 1H), 5.13 (dd, *J* = 12.4, 5.6 Hz, 1H), 4.78 (t, *J* = 4.8 Hz, 4H), 4.72 (s, 2H), 4.45 (t, *J* = 5.2 Hz, 2H), 3.73 – 3.67 (m, 6H), 2.89 – 2.82 (m, 1H), 2.80 – 2.71 (m, 2H), 2.32 (t, *J* = 7.2 Hz, 2H), 1.89 (t, *J* = 7.0 Hz, 2H), 1.33 – 1.26 (m, 2H); HRMS (ESI-TOF): $[M+H]^+$ calculated for $C_{50}H_{50}N_{11}O_{12}$: 996.3640, found: 996.3623.

G4L-PROTAC4

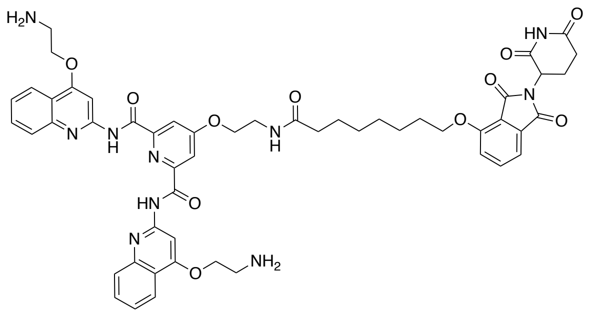

N^2,N^6-bis(4-(2-aminoethoxy)quinolin-2-yl)-4-(2-(8-((2-(2,6-dioxopiperidin-3-yl)-1,3-dioxoisoindolin-4-yl)oxy)octanamido)ethoxy)pyridine-2,6-dicarboxamide: Thalidomide-C8-COOH (13.0 mg, 30 μmol) and HATU (11.4 mg, 30 μmol) were dissolved in 0.5 mL anhydrous DMF. Di-Boc protected PDS (**S1**, 20 mg, 25 μmol) was added to the mixture followed by the addition of DIPEA (23 μL, 125 μmol), and the reaction was stirred for 2 h in room temperature under argon. After completion, the solvent was removed under reduced pressure. The resulting crude material was dissolved in 1 mL of 30% TFA in DCM (v/v) and stirred at room temperature for 30 minutes. The solvent was then evaporated to dryness under reduced pressure. The crude product was purified by reversed-phase flash column chromatography using a C18 column, with a gradient elution from water (containing 0.1% TFA, v/v) to acetonitrile (containing 0.1% TFA, v/v) over 30 minutes at a flow rate of 18 mL/min. Solvents were removed by lyophilization to yield the desired G4L-PROTAC4 as white powder (yield: 94%); ^{1}H NMR (400 MHz, DMSO) δ 12.09 (s, 2H), 11.09 (s, 1H), 8.44 (d, J = 8.4 Hz, 2H), 8.18 (s, 6H), 8.11 (s, 3H), 7.97 – 7.89 (m, 4H), 7.85 – 7.77 (m, 2H), 7.75 – 7.69 (m, 1H), 7.59 – 7.49 (m, 3H), 7.42 (d, J = 8.6 Hz, 1H), 7.36 (d, J = 7.2 Hz, 1H), 5.06 (dd, J = 12.8, 5.4 Hz, 1H), 4.51 (t, J = 5.0 Hz, 4H), 4.34 (t, J = 5.3 Hz, 2H), 4.13 (t, J = 6.5 Hz, 2H), 3.17 (s, 7H), 3.03 (s, 1H), 2.90 (s, 1H), 2.11 (t, J = 7.3 Hz, 2H), 1.71 (s, 2H), 1.51 (s, 3H), 1.26 (dd, J = 11.8, 5.7 Hz, 11H), 0.86 (s, 1H); HRMS (ESI-TOF): [M+H]$^+$ calculated for $C_{52}H_{55}N_{10}O_{11}$: 995.4046, found: 995.4033.

G4L-PROTAC5

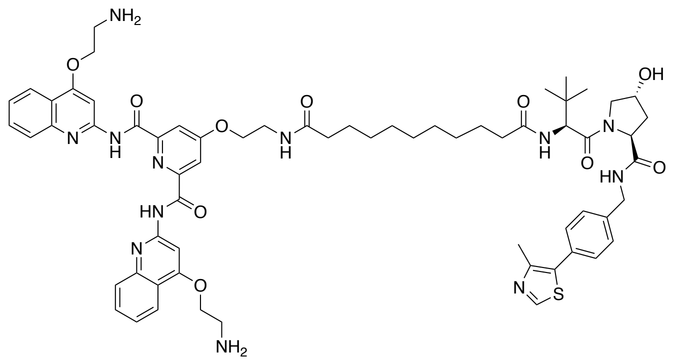

N^2,N^6-bis(4-(2-aminoethoxy)quinolin-2-yl)-4-(2-(11-(((*S*)-1-((2*S*,4*R*)-4-hydroxy-2-((4-(4-methylthiazol-5-yl)benzyl)carbamoyl)pyrrolidin-1-yl)-3,3-dimethyl-1-oxobutan-2-yl)amino)-11-oxoundecanamido)ethoxy)pyridine-2,6-dicarboxamide: VH032-alkylC11-COOH (14.8 mg, 30 µmol) and HATU (11.4 mg, 30 µmol) were dissolved in 0.5 mL anhydrous DMF. Di-Boc protected PDS (**S1**, 20 mg, 25 µmol) was added to the mixture followed by the addition of DIPEA (23 µL, 125 µmol), and the reaction was stirred for 2 h in room temperature under argon. After completion, the solvent was removed under reduced pressure. The resulting crude material was dissolved in 1 mL of 30% TFA in DCM (v/v) and stirred at room temperature for 30 minutes. The solvent was then evaporated to dryness under reduced pressure. The crude product was purified by reversed-phase flash column chromatography using a C18 column, with a gradient elution from water (containing 0.1% TFA, v/v) to acetonitrile (containing 0.1% TFA, v/v) over 30 minutes at a flow rate of 18 mL/min. Solvents were removed by lyophilization to yield the desired G4L-PROTAC5 as white powder (yield: 89%); ^{1}H NMR (400 MHz, DMSO) δ 12.10 (s, 2H), 8.98 (s, 1H), 8.56 (t, *J* = 6.0 Hz, 1H), 8.45 (d, *J* = 9.8 Hz, 2H), 8.14 (d, *J* = 18.9 Hz, 10H), 7.98 – 7.90 (m, 5H), 7.85 – 7.77 (m, 3H), 7.57 (t, *J* = 7.6 Hz, 3H), 7.38 (dd, *J* = 15.5, 7.0 Hz, 5H), 4.59 – 4.29 (m, 12H), 3.49 (s, 12H), 2.44 (s, 4H), 2.10 (t, *J* = 7.6 Hz, 5H), 1.47 (s, 7H), 1.22 (s, 11H), 0.92 (s, 11H); ^{13}C NMR (101 MHz, DMSO) δ 182.2, 163.8, 162.2, 151.5, 147.5, 140.0, 133.3, 130.1, 129.1, 127.9, 119.5, 110.2, 95.5, 76.3, 69.3, 65.6, 58.7, 51.7, 47.0, 40.6, 40.4, 40.3, 40.2, 40.0, 39.8, 39.6, 39.4, 38.7, 35.7, 29.1, 26.8, 16.4; HRMS (ESI-TOF): $[M+H]^+$ calculated for $C_{64}H_{79}N_{12}O_{10}S$: 1207.5757, found: 1207.5746.

G4L-PROTAC6

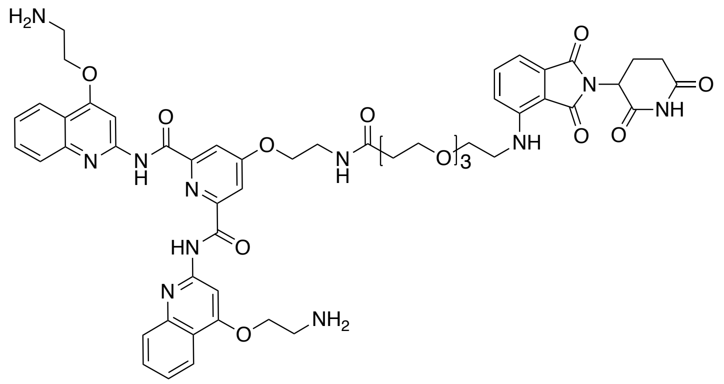

***N*²,*N*⁶-bis(4-(2-aminoethoxy)quinolin-2-yl)-4-(2-(3-(2-((2-(2,6-dioxopiperidin-3-yl)-1,3-dioxoisoindolin-4-yl)amino)ethoxy)propanamido)ethoxy)pyridine-2,6-dicarboxamide:** Pomalidomide-PEG3-COOH (14.3 mg, 30 μmol) and HATU (11.4 mg, 30 μmol) were dissolved in 0.5 mL anhydrous DMF. Di-Boc protected PDS (**S1**, 20 mg, 25 μmol) was added to the mixture followed by the addition of DIPEA (23 μL, 125 μmol), and the reaction was stirred for 2 h in room temperature under argon. After completion, the solvent was removed under reduced pressure. The resulting crude material was dissolved in 1 mL of 30% TFA in DCM (v/v) and stirred at room temperature for 30 minutes. The solvent was then evaporated to dryness under reduced pressure. The crude product was purified by reversed-phase flash column chromatography using a C18 column, with a gradient elution from water (containing 0.1% TFA, v/v) to acetonitrile (containing 0.1% TFA, v/v) over 30 minutes at a flow rate of 18 mL/min. Solvents were removed by lyophilization to yield the desired G4L-PROTAC6 as yellow powder (yield: 95%); ^{1}H NMR (400 MHz, DMSO) δ 12.10 (s, 2H), 11.09 (s, 1H), 8.45 (d, J = 8.3 Hz, 2H), 8.24 (s, 7H), 8.11 (s, 2H), 7.99 – 7.90 (m, 4H), 7.81 (t, J = 8.4 Hz, 2H), 7.60 – 7.51 (m, 3H), 7.09 (d, J = 8.6 Hz, 1H), 7.01 (d, J = 7.0 Hz, 1H), 6.57 (s, 1H), 5.05 (dd, J = 12.8, 5.4 Hz, 1H), 4.52 (t, J = 5.1 Hz, 4H), 4.34 (t, J = 5.5 Hz, 2H), 3.60 (q, J = 5.7 Hz, 5H), 3.52 (d, J = 5.6 Hz, 8H), 3.44 (s, 2H), 2.94 – 2.82 (m, 1H), 2.58 (d, J = 13.6 Hz, 2H), 2.36 (t, J = 6.4 Hz, 2H), 2.11 – 1.98 (m, 1H); ^{13}C NMR (101 MHz, DMSO) δ 173.3, 171.1, 170.5, 169.4, 167.7, 167.6, 163.7, 162.2, 159.3, 159.0, 158.6, 152.7, 151.5, 147.4, 146.8, 136.6, 132.5, 131.3, 127.1, 125.0, 123.2, 120.8, 119.5, 117.9, 114.9, 112.6, 111.1, 109.7, 95.5, 70.2, 70.2, 70.0, 69.3, 68.0, 67.2, 65.7, 49.0, 42.1, 40.6, 40.4, 40.2, 40.0, 39.8, 39.6, 39.3, 38.7, 38.2, 36.5, 31.4, 22.6. HRMS (ESI-TOF): $[M+H]^+$ calculated for $C_{53}H_{58}N_{11}O_{13}$: 1056.4210, found: 1056.4205.

G4L-PROTAC7

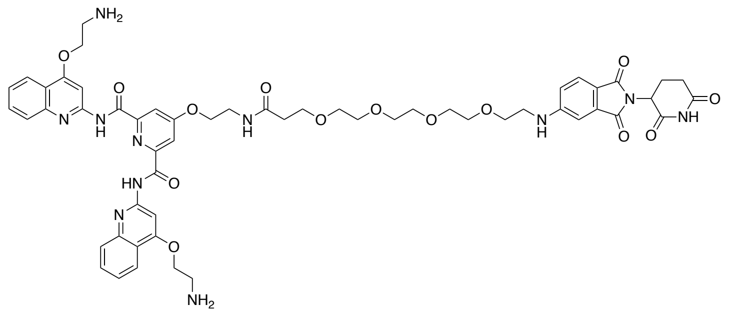

***N*²,*N*⁶-bis(4-(2-aminoethoxy)quinolin-2-yl)-4-((1-((2-(2,6-dioxopiperidin-3-yl)-1,3-dioxoisoindolin-5-yl)amino)-15-oxo-3,6,9,12-tetraoxa-16-azaoctadecan-18-yl)oxy)pyridine-2,6-dicarboxamide**

Pomalidomide-PEG4-COOH (15.6 mg, 30 μmol) and HATU (11.4 mg, 30 μmol) were dissolved in 0.5 mL anhydrous DMF. Di-Boc protected PDS (**S1**, 20 mg, 25 μmol) was added to the mixture followed by the addition of DIPEA (23 μL, 125 μmol), and the reaction was stirred for 2 h in room temperature under argon. After completion, the solvent was removed under reduced pressure. The resulting crude material was dissolved in 1 mL of 30% TFA in DCM (v/v) and stirred at room temperature for 30 minutes. The solvent was then evaporated to dryness under reduced pressure. The crude product was purified by reversed-phase flash column chromatography using a C18 column, with a gradient elution from water (containing 0.1% TFA, v/v) to acetonitrile (containing 0.1% TFA, v/v) over 30 minutes at a flow rate of 18 mL/min. Solvents were removed by lyophilization to yield the desired G4L-PROTAC7 as yellow powder (yield: 92%); ^{1}H NMR (400 MHz, DMSO) δ 12.10 (s, 2H), 11.09 (s, 1H), 8.46 (d, *J* = 10.0 Hz, 2H), 8.25 (s, 8H), 8.11 (s, 2H), 8.00 – 7.90 (m, 5H), 7.86 – 7.76 (m, 3H), 7.61 – 7.50 (m, 4H), 7.11 (d, *J* = 8.6 Hz, 1H), 7.02 (d, *J* = 7.0 Hz, 1H), 6.58 (s, 1H), 5.05 (dd, *J* = 12.9, 5.4 Hz, 1H), 4.53 (s, 4H), 4.34 (s, 2H), 3.60 (q, *J* = 6.1 Hz, 5H), 2.95 – 2.82 (m, 1H), 2.59 (d, *J* = 16.4 Hz, 2H), 2.37 (t, *J* = 6.4 Hz, 2H), 2.10 – 1.98 (m, 1H); ^{13}C NMR (101 MHz, DMSO) δ 173.3, 171.1, 170.5, 169.4, 167.7, 167.6, 163.7, 162.3, 159.4, 159.0, 158.7, 158.3, 152.7, 151.5, 147.3, 146.8, 136.6, 132.5, 131.3, 127.1, 125.0, 123.2, 120.6, 119.5, 117.8, 117.7, 114.8, 112.6, 111.9, 111.1, 109.7, 95.5, 70.3, 70.2, 70.1, 70.0, 69.3, 68.0, 67.2, 65.7, 49.0, 42.1, 40.6, 40.4, 40.2, 40.0, 39.8, 39.5, 39.3, 38.7, 38.2, 36.5, 31.4, 22.6; HRMS (ESI-TOF): $[M+H]^+$ calculated for $C_{55}H_{62}N_{11}O_{14}$: 1100.4472, found: 1100.4467.

G4L-PROTAC8

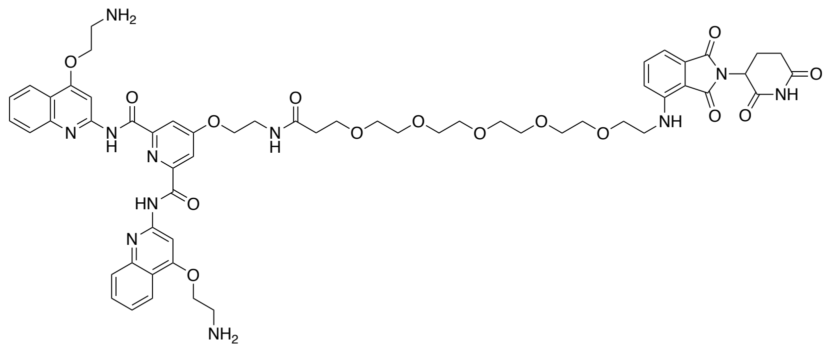

N^2,N^6-bis(4-(2-aminoethoxy)quinolin-2-yl)-4-((1-((2-(2,6-dioxopiperidin-3-yl)-1,3-dioxoisoindolin-4-yl)amino)-18-oxo-3,6,9,12,15-pentaoxa-19-azahenicosan-21-yl)oxy)pyridine-2,6-dicarboxamide

Pomalidomide-PEG5-COOH (16.9 mg, 30 μmol) and HATU (11.4 mg, 30 μmol) were dissolved in 0.5 mL anhydrous DMF. Di-Boc protected PDS (**S1**, 20 mg, 25 μmol) was added to the mixture followed by the addition of DIPEA (23 μL, 125 μmol), and the reaction was stirred for 2 h in room temperature under argon. After completion, the solvent was removed under reduced pressure. The resulting crude material was dissolved in 1 mL of 30% TFA in DCM (v/v) and stirred at room temperature for 30 minutes. The solvent was then evaporated to dryness under reduced pressure. The crude product was purified by reversed-phase flash column chromatography using a C18 column, with a gradient elution from water (containing 0.1% TFA, v/v) to acetonitrile (containing 0.1% TFA, v/v) over 30 minutes at a flow rate of 18 mL/min. Solvents were removed by lyophilization to yield the desired G4L-PROTAC8 as yellow powder (yield: 81%); ^{1}H NMR (400 MHz, DMSO) δ 12.10 (s, 2H), 11.09 (s, 1H), 8.45 (d, *J* = 8.4 Hz, 2H), 8.24 (s, 7H), 8.11 (s, 2H), 8.00 – 7.90 (m, 4H), 7.81 (t, *J* = 7.7 Hz, 2H), 7.61 – 7.50 (m, 3H), 7.12 (d, *J* = 8.6 Hz, 1H), 7.03 (d, *J* = 7.0 Hz, 1H), 6.58 (s, 1H), 5.05 (dd, *J* = 12.9, 5.4 Hz, 1H), 4.52 (t, *J* = 4.9 Hz, 4H), 4.34 (t, *J* = 5.5 Hz, 2H), 3.61 (q, *J* = 5.8 Hz, 5H), 3.53 (s, 4H), 2.93 – 2.82 (m, 1H), 2.59 (d, *J* = 16.6 Hz, 1H), 2.37 (t, *J* = 6.4 Hz, 2H), 2.04 (d, *J* = 7.7 Hz, 1H); ^{13}C NMR (101 MHz, DMSO) δ 173.3, 171.1, 170.5, 169.4, 167.7, 167.6, 163.8, 162.2, 158.9, 158.6, 152.8, 151.5, 147.4, 146.8, 136.7, 132.5, 131.3, 127.1, 125.0, 123.2, 119.5, 117.9, 115.0, 112.6, 111.1, 109.7, 95.5, 70.3, 70.2, 70.1, 70.0, 69.3, 67.2, 65.7, 49.0, 42.1, 40.6, 40.4, 40.2, 40.1, 40.0, 39.8, 39.6, 39.3, 38.7, 36.5, 31.4, 22.6; HRMS (ESI-TOF): $[M+H]^+$ calculated for $C_{57}H_{66}N_{11}O_{15}$: 1144.4734, found: 1144.4727.

G4L-PROTAC9

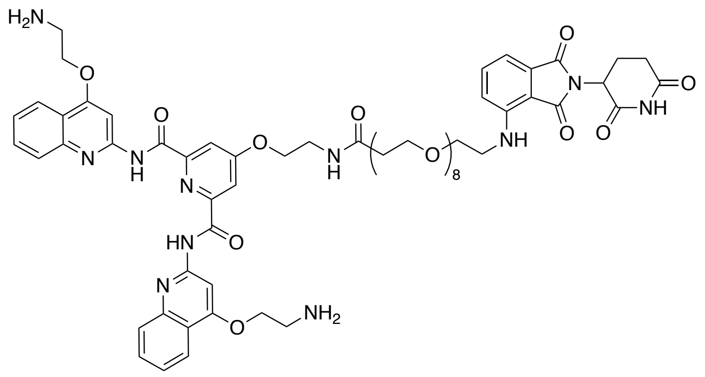

N^2,N^6-bis(4-(2-aminoethoxy)quinolin-2-yl)-4-((1-((2-(2,6-dioxopiperidin-3-yl)-1,3-dioxoisoindolin-4-yl)amino)-27-oxo-3,6,9,12,15,18,21,24-octaoxa-28-azatriacontan-30-yl)oxy)pyridine-2,6-dicarboxamide

Pomalidomide-5'-PEG8-C2-COOH (20.9 mg, 30 μmol) and HATU (11.4 mg, 30 μmol) were dissolved in 0.5 mL anhydrous DMF. Di-Boc protected PDS (**S1**, 20 mg, 25 μmol) was added to the mixture followed by the addition of DIPEA (23 μL, 125 μmol), and the reaction was stirred for 2 h in room temperature under argon. After completion, the solvent was removed under reduced pressure. The resulting crude material was dissolved in 1 mL of 30% TFA in DCM (v/v) and stirred at room temperature for 30 minutes. The solvent was then evaporated to dryness under reduced pressure. The crude product was purified by reversed-phase flash column chromatography using a C18 column, with a gradient elution from water (containing 0.1% TFA, v/v) to acetonitrile (containing 0.1% TFA, v/v) over 30 minutes at a flow rate of 18 mL/min. Solvents were removed by lyophilization to yield the desired G4L-PROTAC9 as yellow powder (yield: 89%); ^{1}H NMR (400 MHz, DMSO) δ 12.10 (s, 2H), 11.09 (s, 1H), 8.45 (d, *J* = 8.4 Hz, 2H), 8.21 (s, 8H), 8.11 (s, 2H), 7.99 – 7.89 (m, 4H), 7.81 (t, *J* = 8.3 Hz, 3H), 7.57 (t, *J* = 5.7 Hz, 3H), 7.13 (d, *J* = 8.7 Hz, 1H), 7.07 – 6.97 (m, 1H), 5.05 (dd, *J* = 12.9, 5.3 Hz, 1H), 4.52 (t, *J* = 5.1 Hz, 12H), 4.34 (s, 7H), 3.62 (t, *J* = 6.3 Hz, 6H), 2.85 (s, 2H), 2.61 (s, 2H), 2.38 (d, *J* = 6.4 Hz, 2H), 2.01 (s, 1H); HRMS (ESI-TOF): $[M+H]^+$ calculated for $C_{63}H_{78}N_{11}O_{18}$: 1276.5521, found: 1276.5510.

G4L-PROTAC10

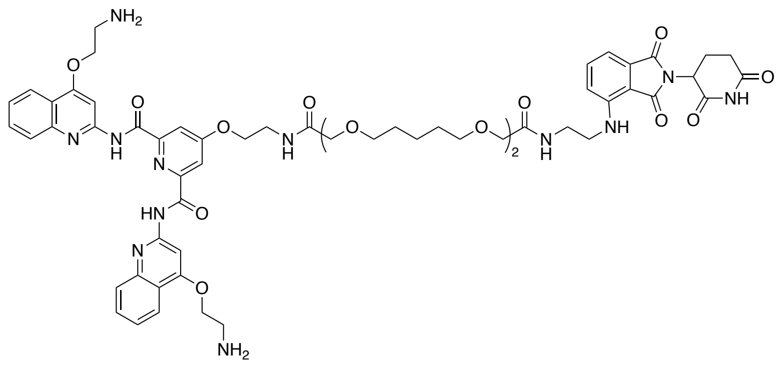

***N*²,*N*⁶-bis(4-(2-aminoethoxy)quinolin-2-yl)-4-((26-((2-(2,6-dioxopiperidin-3-yl)-1,3-dioxoisoindolin-4-yl)amino)-4,23-dioxo-6,12,15,21-tetraoxa-3,24-diazahexacosyl)oxy)pyridine-2,6-dicarboxamide**

Pomalidomide-C2-amido-(C1-O-C5-O-C1)2-COOH (19.5 mg, 30 μmol) and HATU (11.4 mg, 30 μmol) were dissolved in 0.5 mL anhydrous DMF. Di-Boc protected PDS (**S1**, 20 mg, 25 μmol) was added to the mixture followed by the addition of DIPEA (23 μL, 125 μmol), and the reaction was stirred for 2 h in room temperature under argon. After completion, the solvent was removed under reduced pressure. The resulting crude material was dissolved in 1 mL of 30% TFA in DCM (v/v) and stirred at room temperature for 30 minutes. The solvent was then evaporated to dryness under reduced pressure. The crude product was purified by reversed-phase flash column chromatography using a C18 column, with a gradient elution from water (containing 0.1% TFA, v/v) to acetonitrile (containing 0.1% TFA, v/v) over 30 minutes at a flow rate of 18 mL/min. Solvents were removed by lyophilization to yield the desired G4L-PROTAC10 as yellow powder (yield: 92%); ^{1}H NMR (400 MHz, DMSO) δ 12.09 (s, 2H), 11.09 (s, 1H), 8.45 (d, J = 8.3 Hz, 2H), 8.23 (s, 7H), 8.12 (s, 3H), 7.99 – 7.86 (m, 7H), 7.81 (t, J = 7.7 Hz, 3H), 7.55 (d, J = 7.7 Hz, 3H), 7.35 (s, 1H), 7.19 (d, J = 8.6 Hz, 1H), 7.02 (d, J = 6.6 Hz, 1H), 6.73 (s, 1H), 5.06 (s, 1H), 4.52 (s, 5H), 4.40 (s, 3H), 3.83 (d, J = 24.9 Hz, 20H), 3.35 (d, J = 34.6 Hz, 18H), 2.87 (s, 2H), 2.01 (s, 1H), 1.38 (d, J = 76.5 Hz, 14H); ^{13}C NMR (101 MHz, DMSO) δ 173.3, 170.3, 163.7, 162.2, 152.8, 147.5, 140.1, 131.3, 125.0, 119.5, 95.5, 71.3, 70.6, 70.2, 69.9, 65.6, 40.6, 40.4, 40.2, 40.0, 39.8, 39.6, 39.3, 38.7, 29.4, 22.6; HRMS (ESI-TOF): $[M+H]^+$ calculated for $C_{62}H_{75}N_{12}O_{15}$: 1227.5469, found: 1227.5478.

G4L-PROTAC11

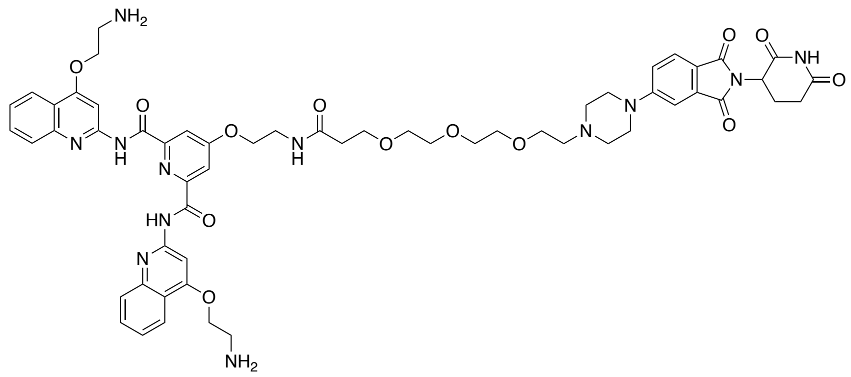

***N*²,*N*⁶-bis(4-(2-aminoethoxy)quinolin-2-yl)-4-((1-(4-(2-(2,6-dioxopiperidin-3-yl)-1,3-dioxoisoindolin-5-yl)piperazin-1-yl)-12-oxo-3,6,9-trioxa-13-azapentadecan-15-yl)oxy)pyridine-2,6-dicarboxamide**

Thalidomide-PEG3-piperazine-COOH (16.3 mg, 30 μmol) and HATU (11.4 mg, 30 μmol) were dissolved in 0.5 mL anhydrous DMF. Di-Boc protected PDS (**S1**, 20 mg, 25 μmol) was added to the mixture followed by the addition of DIPEA (23 μL, 125 μmol), and the reaction was stirred for 2 h in room temperature under argon. After completion, the solvent was removed under reduced pressure. The resulting crude material was dissolved in 1 mL of 30% TFA in DCM (v/v) and stirred at room temperature for 30 minutes. The solvent was then evaporated to dryness under reduced pressure. The crude product was purified by reversed-phase flash column chromatography using a C18 column, with a gradient elution from water (containing 0.1% TFA, v/v) to acetonitrile (containing 0.1% TFA, v/v) over 30 minutes at a flow rate of 18 mL/min. Solvents were removed by lyophilization to yield the desired G4L-PROTAC11 as white powder (yield: 93%); ^{1}H NMR (400 MHz, DMSO) δ 12.10 (s, 1H), 11.09 (s, 1H), 8.46 (d, J = 10.0 Hz, 1H), 8.29 (s, 4H), 8.10 (s, 1H), 7.99 – 7.90 (m, 2H), 7.85 – 7.71 (m, 2H), 7.56 (t, J = 8.3 Hz, 1H), 7.48 (d, J = 8.7 Hz, 1H), 7.39 – 7.31 (m, 1H), 6.26 (s, 9H), 5.17 – 4.98 (m, 1H), 4.52 (s, 2H), 4.34 (s, 1H), 4.21 (s, 2H), 3.79 (s, 2H), 3.69 – 3.43 (m, 17H), 3.38 (s, 3H), 2.89 (s, 1H), 2.60 (d, J = 14.3 Hz, 2H), 2.46 (t, J = 6.3 Hz, 1H), 2.39 (t, J = 6.4 Hz, 1H), 2.11 – 1.98 (m, 1H); ^{13}C NMR (101 MHz, DMSO) δ 173.3, 173.1, 171.0, 170.5, 167.8, 167.6, 167.3, 163.7, 162.3, 159.4, 159.0, 158.7, 158.3, 154.5, 152.7, 151.5, 147.3, 134.3, 131.3, 127.1, 125.4, 123.2, 120.4, 119.5, 119.2, 117.8, 114.9, 95.5, 70.1, 70.0, 70.0, 69.9, 67.2, 66.7, 65.7, 64.7, 55.3, 51.1, 49.3, 44.6, 40.6, 40.4, 40.3, 40.2, 40.0, 39.7, 39.5, 39.3, 38.7, 36.5, 35.2, 31.4, 22.6; HRMS (ESI-TOF): $[M+H]^+$ calculated for $C_{57}H_{65}N_{12}O_{13}$: 1125.4789, found: 1125.4777.

G4L-PROTAC12

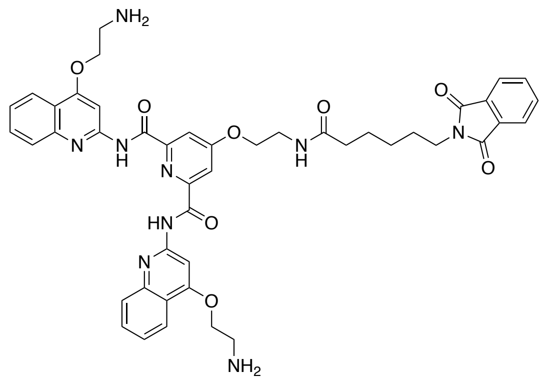

N^2,N^6-bis(4-(2-aminoethoxy)quinolin-2-yl)-4-(2-(6-(1,3-dioxoisoindolin-2-yl)hexanamido)ethoxy)pyridine-2,6-dicarboxamide

O-Phthalimide-C5-acid (7.8 mg, 30 μmol) and HATU (11.4 mg, 30 μmol) were dissolved in 0.5 mL anhydrous DMF. Di-Boc protected PDS (**S1**, 20 mg, 25 μmol) was added to the mixture followed by the addition of DIPEA (23 μL, 125 μmol), and the reaction was stirred for 2 h in room temperature under argon. After completion, the solvent was removed under reduced pressure. The resulting crude material was dissolved in 1 mL of 30% TFA in DCM (v/v) and stirred at room temperature for 30 minutes. The solvent was then evaporated to dryness under reduced pressure. The crude product was purified by reversed-phase flash column chromatography using a C18 column, with a gradient elution from water (containing 0.1% TFA, v/v) to acetonitrile (containing 0.1% TFA, v/v) over 30 minutes at a flow rate of 18 mL/min. Solvents were removed by lyophilization to yield the desired G4L-PROTAC12 as white powder (yield: 85%);^{1}H NMR (400 MHz, DMSO) δ 12.08 (s, 2H), 8.44 (d, *J* = 8.3 Hz, 2H), 8.26 (s, 6H), 8.11 (s, 3H), 7.99 – 7.89 (m, 4H), 7.81 (t, *J* = 6.6 Hz, 6H), 7.56 (t, *J* = 7.6 Hz, 2H), 4.52 (t, *J* = 5.0 Hz, 5H), 4.33 (t, *J* = 5.5 Hz, 3H), 3.51 (dt, *J* = 10.9, 6.4 Hz, 9H), 2.10 (t, *J* = 7.4 Hz, 2H), 1.55 (dp, *J* = 15.4, 7.4 Hz, 4H), 1.25 (qd, *J* = 9.0, 6.0 Hz, 2H); ^{13}C NMR (101 MHz, DMSO) δ 173.0, 168.4, 167.6, 163.7, 162.2, 159.0, 158.6, 152.7, 151.5, 147.3, 134.8, 132.0, 131.3, 127.1, 125.0, 123.4, 123.2, 119.5, 117.9, 112.6, 95.5, 67.9, 65.7, 40.6, 40.4, 40.3, 40.2, 40.0, 39.8, 39.5, 39.3, 38.7, 38.1, 37.7, 35.6, 28.2, 26.4, 25.2; HRMS (ESI-TOF): $[M+H]^+$ calculated for $C_{45}H_{46}N_9O_8$: 840.3464, found: 840.3452.

G4L-PROTAC13

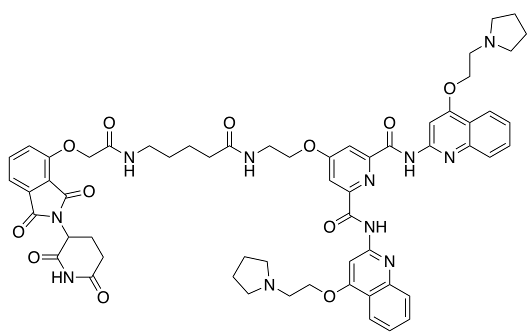

4-(2-(5-(2-((2-(2,6-dioxopiperidin-3-yl)-1,3-dioxoisoindolin-4-yl)oxy)acetamido)pentanamido)ethoxy)-*N*²,*N*⁶-bis(4-(2-(pyrrolidin-1-yl)ethoxy)quinolin-2-yl)pyridine-2,6-dicarboxamide: ^{1}H NMR (500 MHz MeOH-d$_4$): δ 8.31 (d, *J* = 8.5 Hz, 2H), 8.03 (s, 2H), 7.97 (s, 2H), 7.89 (d, *J* = 8.5 Hz, 2H), 7.81 – 7.75 (m, 2H), 7.60 – 7.54 (m, 2H), 7.44 (t, *J* = 7.8 Hz, 1H), 7.21 (d, *J* = 8.5 Hz, 1H), 7.17 (d, *J* = 7.5 Hz, 1H), 5.13 (dd, *J* = 13.0, 5.5 Hz, 1H), 4.77 (t, *J* = 4.2 Hz, 4H), 4.57 (s, 2H), 4.39 (t, *J* = 5.2 Hz, 2H), 4.04 – 3.79 (m, 8H), 3.72 (t, *J* = 5.0 Hz, 2H), 3.40 (br, 4H), 3.26 (t, *J* = 7.2 Hz, 2H), 2.93 – 2.83 (m, 1H), 2.81 – 2.67 (m, 2H), 2.32 (t, *J* = 7.0 Hz, 2H), 2.27 – 2.12 (m, 8H), 1.76 – 1.68 (m, 2H), 1.63 – 1.55 (m, 2H); ^{13}C NMR (125 MHz, MeOH-*d*$_4$) δ ^{13}C NMR ^{13}C NMR (125 MHz, MeOH-*d*$_4$) δ 176.3, 174.6, 171.5, 169.6, 169.5, 168.4, 167.6, 164.1, 163.8, 155.8, 152.8, 151.6, 147.4, 138.0, 134.6, 132.5, 127.4, 126.6, 123.3, 121.4, 120.4, 119.0, 117.6, 113.7, 95.9, 69.2, 69.0, 65.7, 56.2, 54.8, 50.6, 40.0, 39.6, 36.6, 32.2, 29.4, 24.3, 24.0, 23.7; HRMS (ESI): [M+H]$^+$ calculated for $C_{59}H_{64}N_{11}O_{12}$: 1118.4736, found: 1118.4718.

G4L-PROTAC14

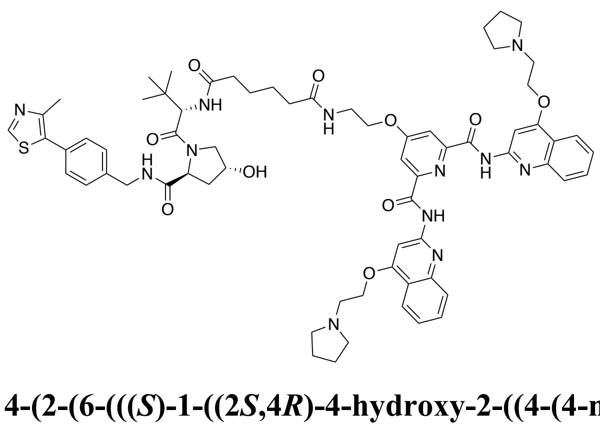

4-(2-(6-(((*S*)-1-((2*S*,4*R*)-4-hydroxy-2-((4-(4-methylthiazol-5-yl)benzyl)carbamoyl)pyrrolidin-1-yl)-3,3-dimethyl-1-oxobutan-2-yl)amino)-6-oxohexanamido)ethoxy)-*N*²,*N*⁶-bis(4-(2-(pyrrolidin-1-yl)ethoxy)quinolin-2-yl)pyridine-2,6-dicarboxamide: ^{1}H NMR (500 MHz, MeOH-*d*$_4$) δ 8.84 (s, 1H), 8.24 (d, *J* = 8.0 Hz, 2H), 7.99 (s, 2H), 7.90 (s, 2H), 7.79 (d, *J* = 8.5 Hz, 2H), 7.67 (t, *J* = 7.5 Hz, 2H), 7.51 (t, *J* = 7.5 Hz,

2H), 7.42 (d, *J* = 8.5 Hz, 2H), 7.36 (d, *J* = 8.0 Hz, 2H), 4.67 (t, *J* = 4.8 Hz, 4H), 4.59 (s, 1H), 4.58 – 4.48 (m, 2H), 4.48 – 4.45 (m, 1H), 4.31 (t, *J* = 5.5 Hz, 2H), 3.93 (t, *J* = 4.5 Hz, 4H), 3.88 (d, *J* = 11.0 Hz, 1H), 3.77 (dd, *J* = 11.0, 4.0 Hz, 1H), 3.68 (t, *J* = 5.2 Hz, 2H), 3.63 (br, 8H), 2.43 (s, 3H), 2.37 – 2.25 (m, 4H), 2.24 – 2.15 (m, 9H), 2.09 – 2.01 (m, 1H), 1.71 – 1.61 (m, 4H), 1.00 (s, 9H); ^{13}C NMR (125 MHz MeOH-d$_4$): δ ^{13}C NMR (125 MHz, MeOH-*d*$_4$) δ 176.4, 175.6, 174.5, 172.3, 169.4, 163.8, 163.5, 152.9, 152.8, 151.8, 149.0, 148.3, 140.2, 133.3, 132.0, 131.5, 130.3, 128.9, 128.0, 126.3, 123.1, 120.5, 113.3, 95.8, 71.1, 68.9, 65.6, 60.8, 59.1, 58.0, 56.3, 54.9, 43.7, 39.6, 38.9, 36.7, 36.5, 36.3, 27.0, 26.5, 24.0, 15.8; HRMS (ESI): [M+H]$^+$ calculated for $C_{67}H_{81}N_{12}O_{10}S$: 1245.5919, found: 1245.5890.

NMR spectra

Di-Boc PDS (di-tert-butyl (((((4-(2-aminoethoxy)pyridine-2,6-dicarbonyl)bis(azanediyl))bis(quinoline-2,4-diyl))bis(oxy))bis(ethane-2,1-diyl))dicarbamate)

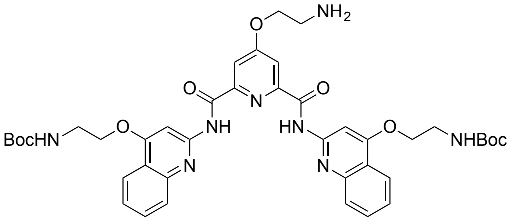

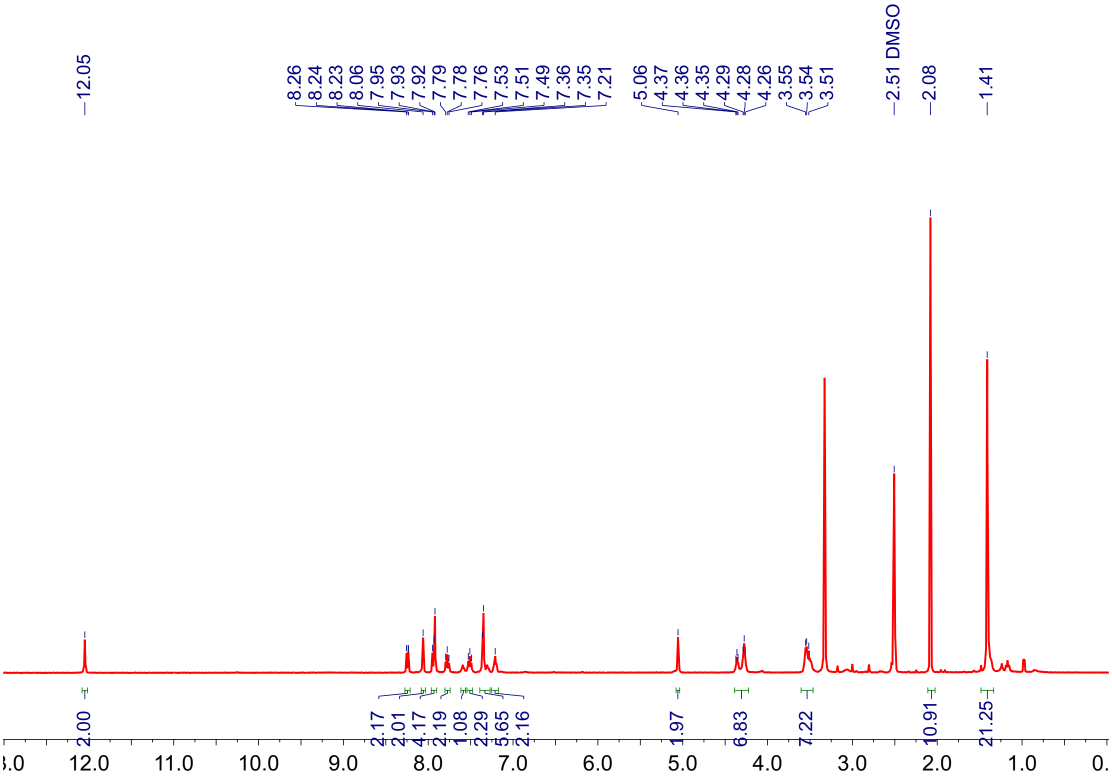

G4L-PROTAC1

(N^2,N^6-bis(4-(2-aminoethoxy)quinolin-2-yl)-4-(2-(2-((2-(2,6-dioxopiperidin-3-yl)-1,3-dioxoisoindolin-4-yl)oxy)acetamido)ethoxy)pyridine-2,6-dicarboxamide)

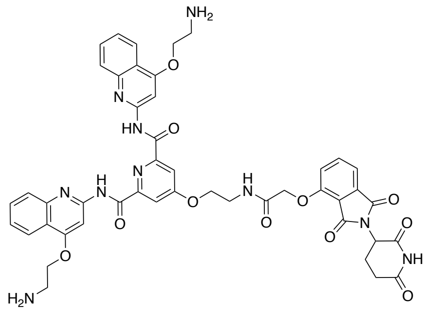

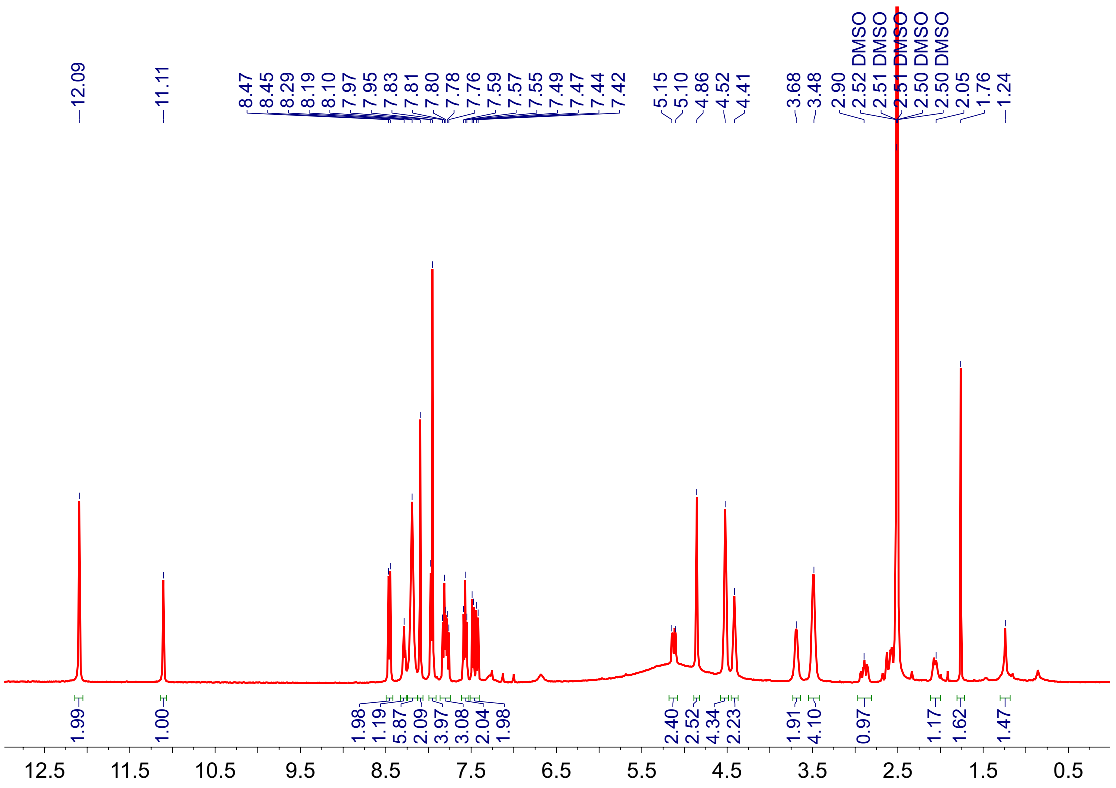

G4L-PROTAC2

***N*²,*N*⁶-bis(4-(2-aminoethoxy)quinolin-2-yl)-4-(2-(7-(((*S*)-1-((2*S*,4*R*)-4-hydroxy-2-((4-(4-methylthiazol-5-yl)benzyl)carbamoyl)pyrrolidin-1-yl)-3,3-dimethyl-1-oxobutan-2-yl)amino)-7-oxoheptanamido)ethoxy)pyridine-2,6-dicarboxamide**

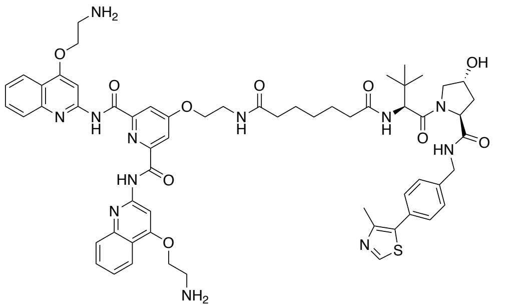

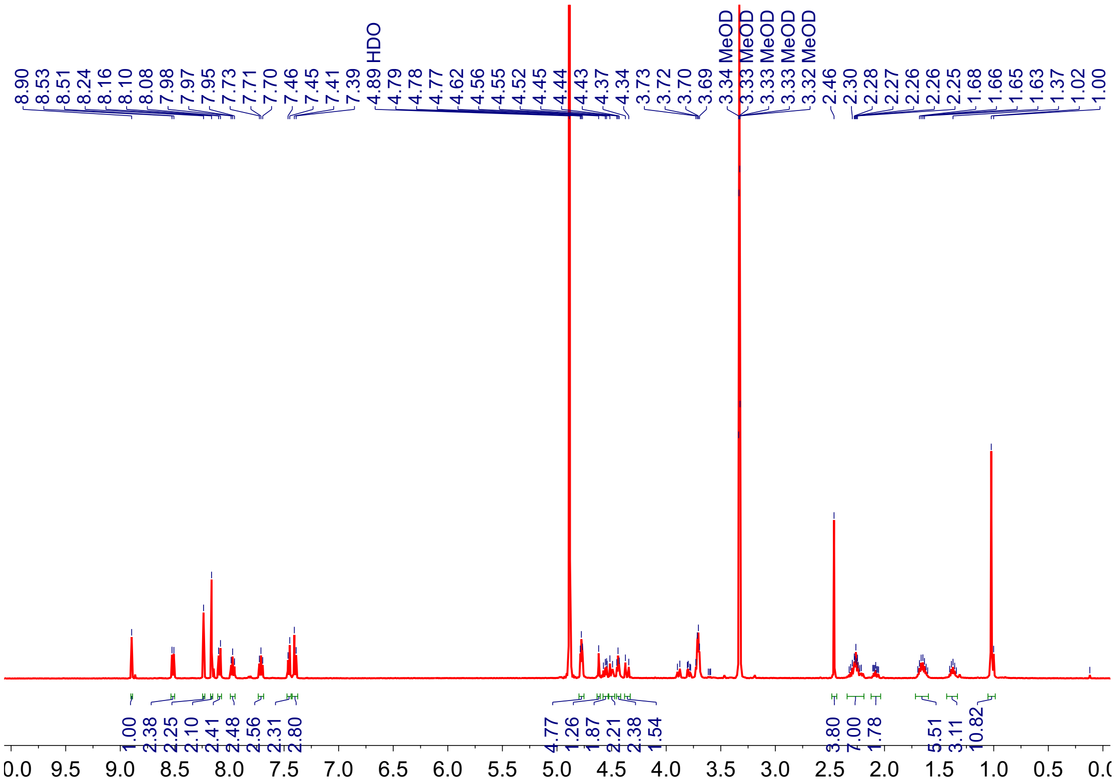

G4L-PROTAC3

***N*²,*N*⁶-bis(4-(2-aminoethoxy)quinolin-2-yl)-4-(2-(4-(2-((2-(2,6-dioxopiperidin-3-yl)-1,3-dioxoisoindolin-4-yl)oxy)acetamido)butanamido)ethoxy)pyridine-2,6-dicarboxamide**

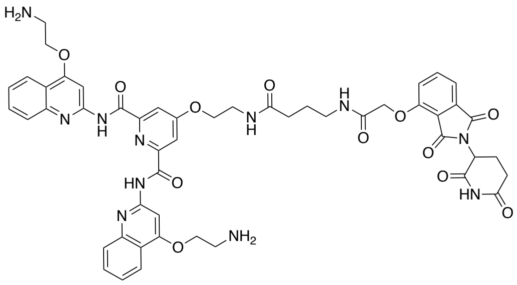

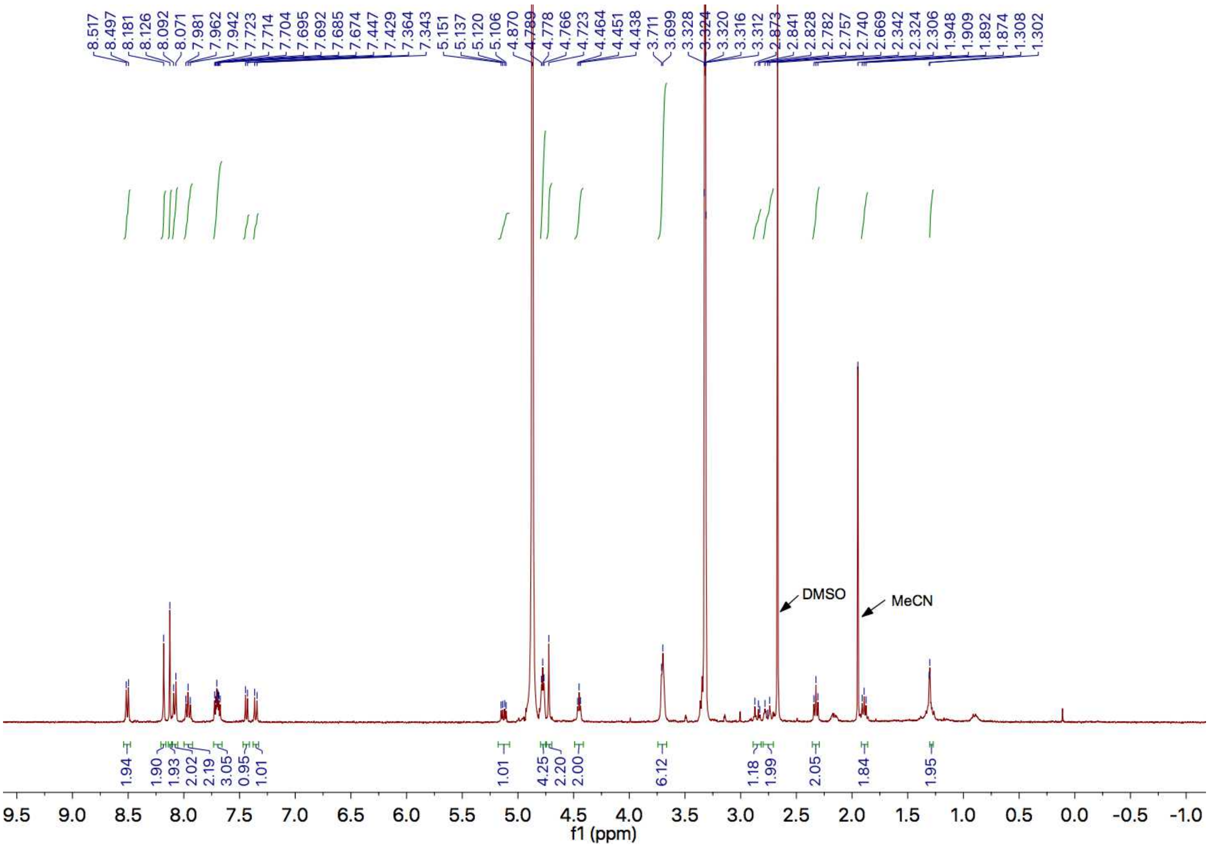

G4L-PROTAC4

***N*[2],*N*[6]-bis(4-(2-aminoethoxy)quinolin-2-yl)-4-(2-(8-((2-(2,6-dioxopiperidin-3-yl)-1,3-dioxoisoindolin-4-yl)oxy)octanamido)ethoxy)pyridine-2,6-dicarboxamide**

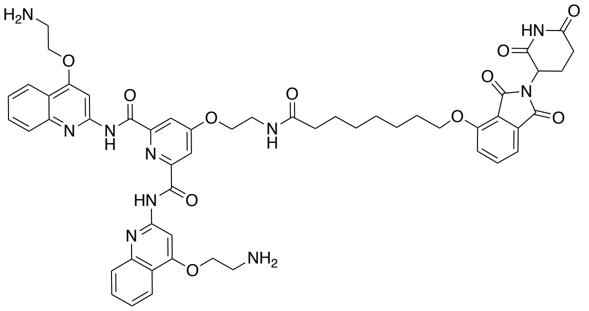

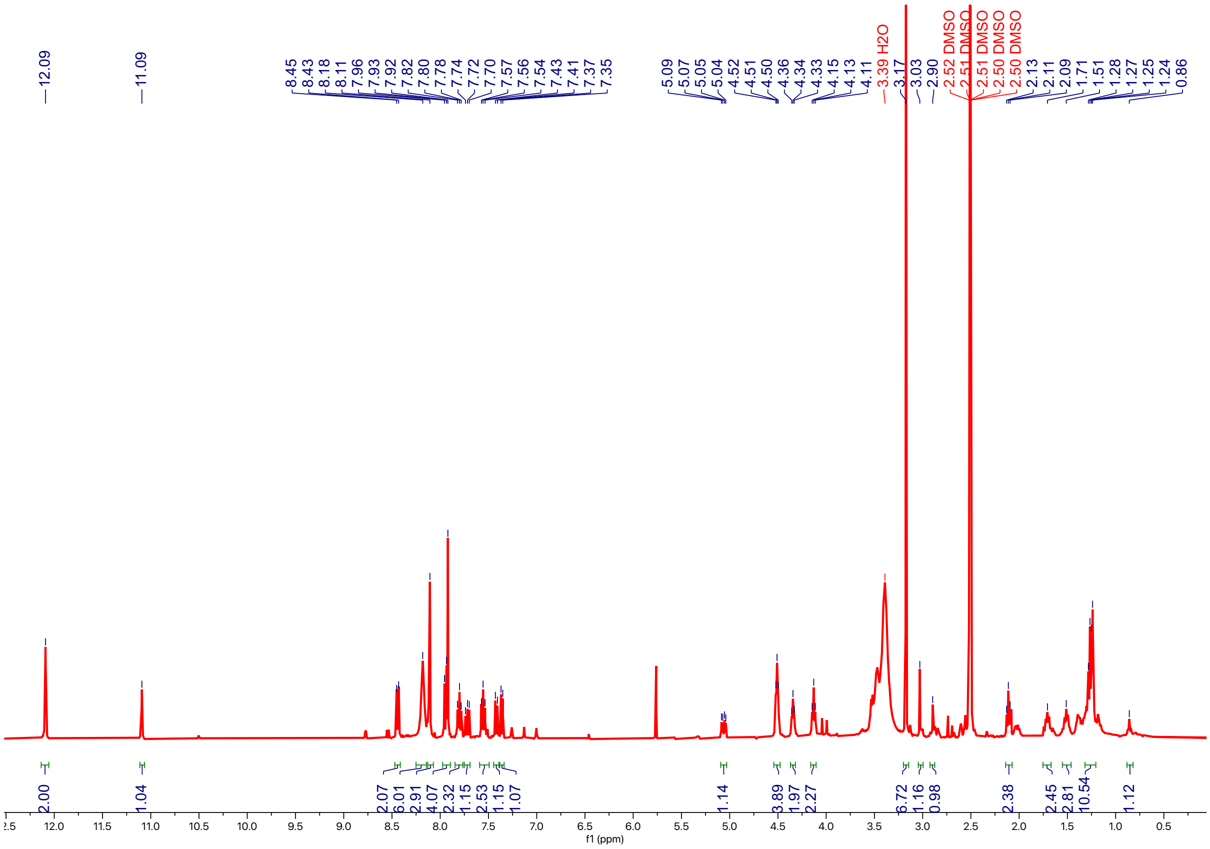

G4L-PROTAC5

***N*²,*N*⁶-bis(4-(2-aminoethoxy)quinolin-2-yl)-4-(2-(11-(((*S*)-1-((2*S*,4*R*)-4-hydroxy-2-((4-(4-methylthiazol-5-yl)benzyl)carbamoyl)pyrrolidin-1-yl)-3,3-dimethyl-1-oxobutan-2-yl)amino)-11-oxoundecanamido)ethoxy)pyridine-2,6-dicarboxamide:**

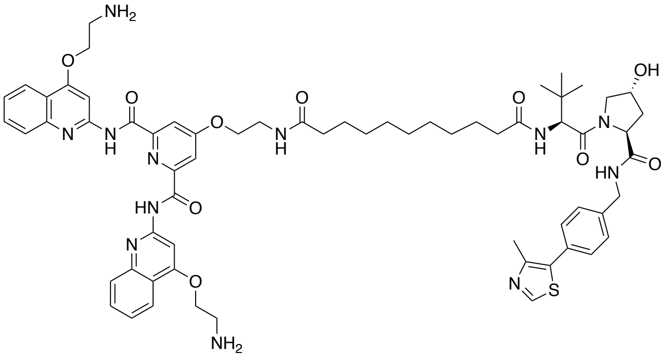

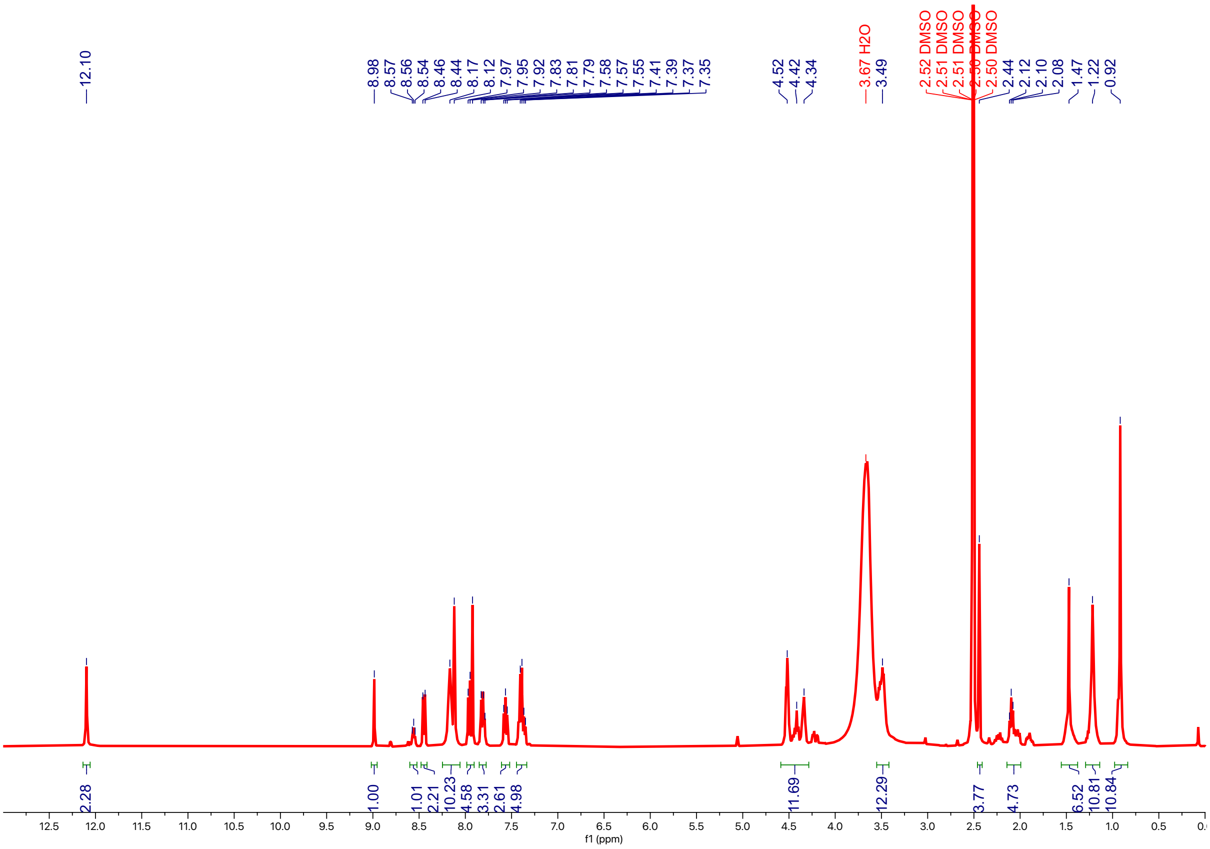

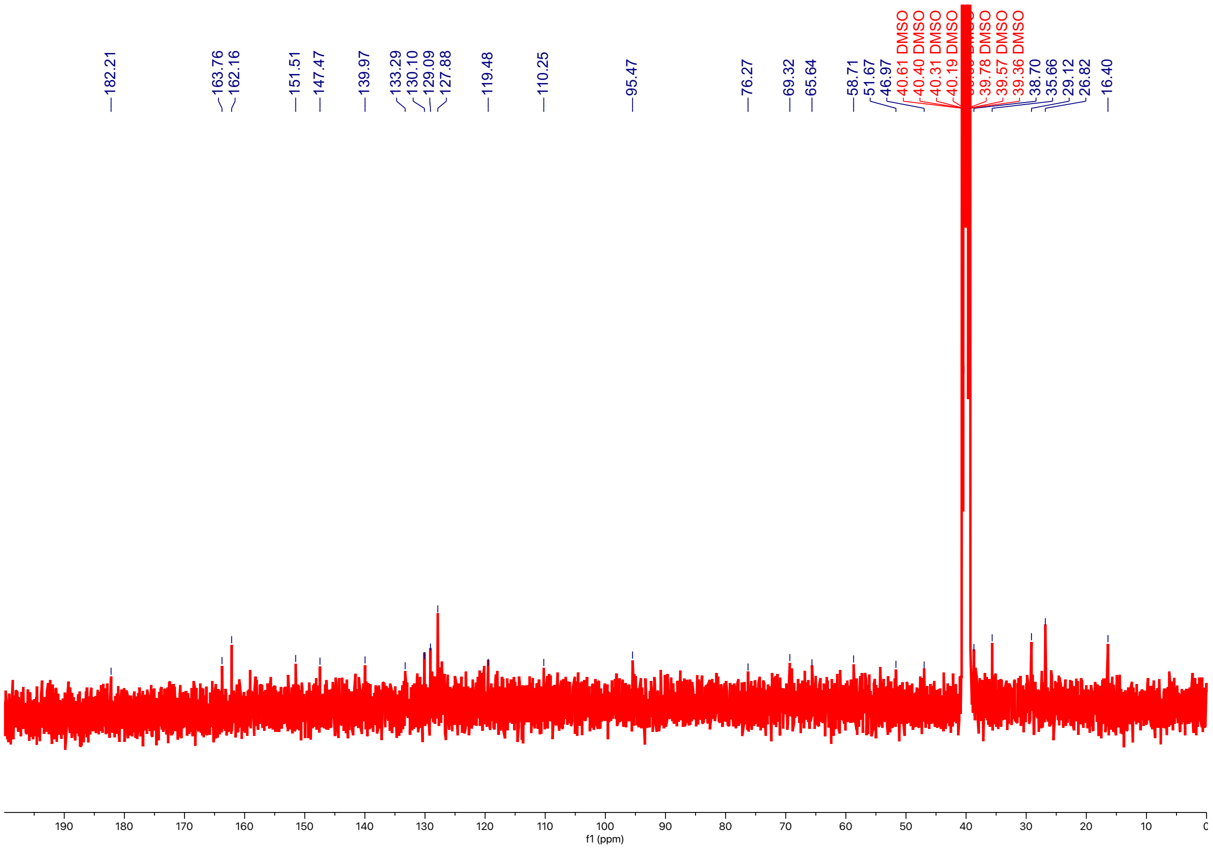
182.21
163.76
162.16
151.51
147.47
139.97
133.29
130.10
129.09
127.88
119.48
110.25
95.47
76.27
69.32
65.64
58.71
51.67
46.97
40.61 DMSO
40.40 DMSO
40.31 DMSO
40.19 DMSO
39.78 DMSO
39.57 DMSO
39.36 DMSO
38.70
35.66
29.12
26.82
16.40
190 180 170 160 150 140 130 120 110 100 90 80 70 60 50 40 30 20 10 0
f1 (ppm)

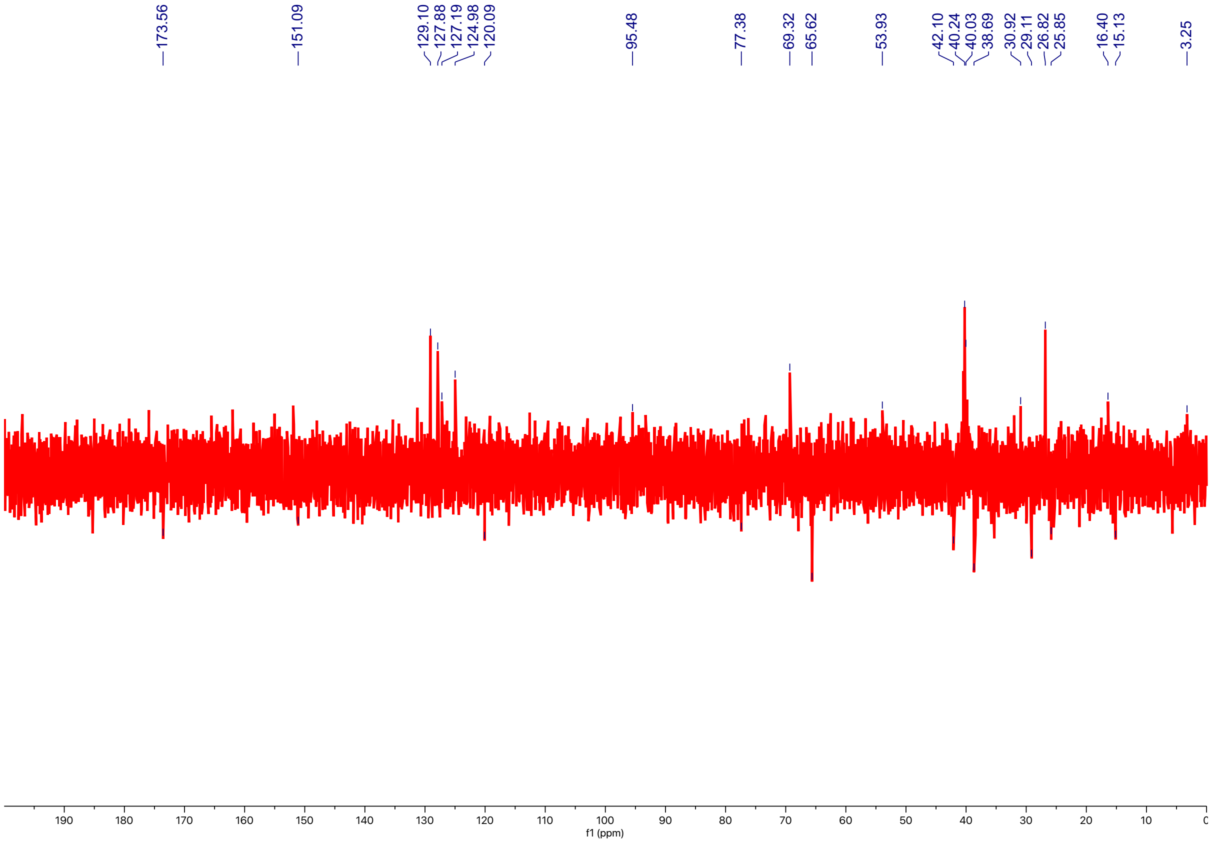
173.56
151.09
129.10
127.88
127.19
124.98
120.09
95.48
77.38
69.32
65.62
53.93
42.10
40.24
40.03
38.69
30.92
29.11
26.82
25.85
16.40
15.13
3.25
190 180 170 160 150 140 130 120 110 100 90 80 70 60 50 40 30 20 10 0
f1 (ppm)

G4L-PROTAC6

N[2],*N*[6]-bis(4-(2-aminoethoxy)quinolin-2-yl)-4-(2-(3-(2-((2-(2,6-dioxopiperidin-3-yl)-1,3-dioxoisoindolin-4-yl)amino)ethoxy)propanamido)ethoxy)pyridine-2,6-dicarboxamide

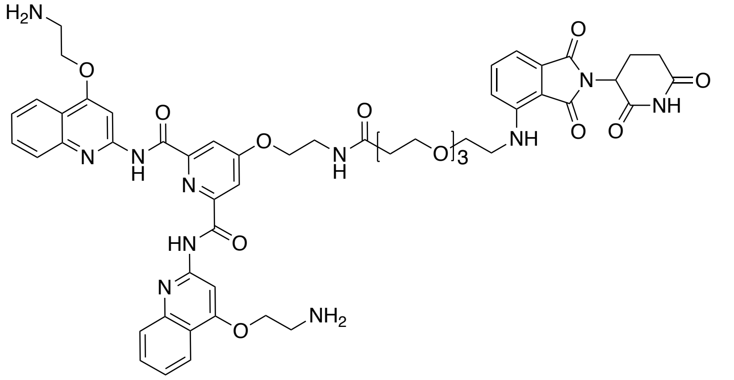

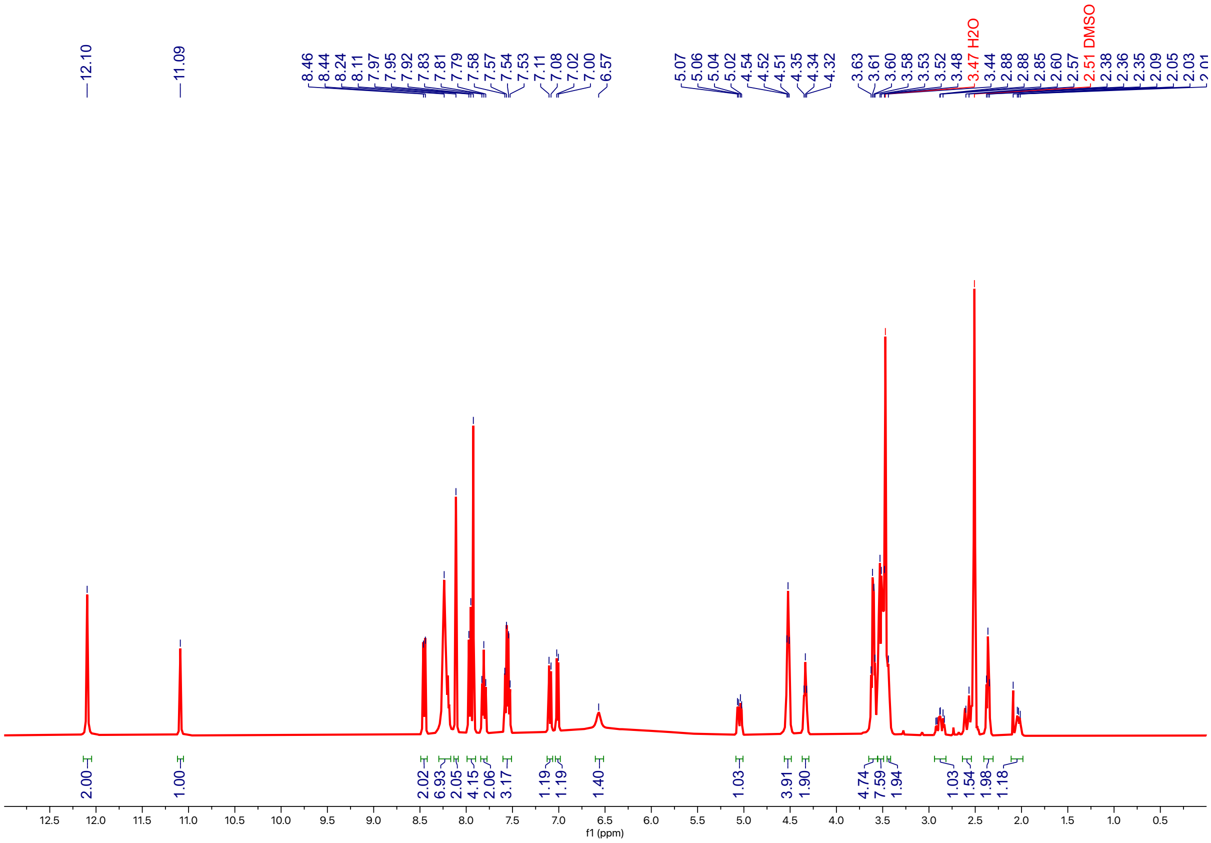

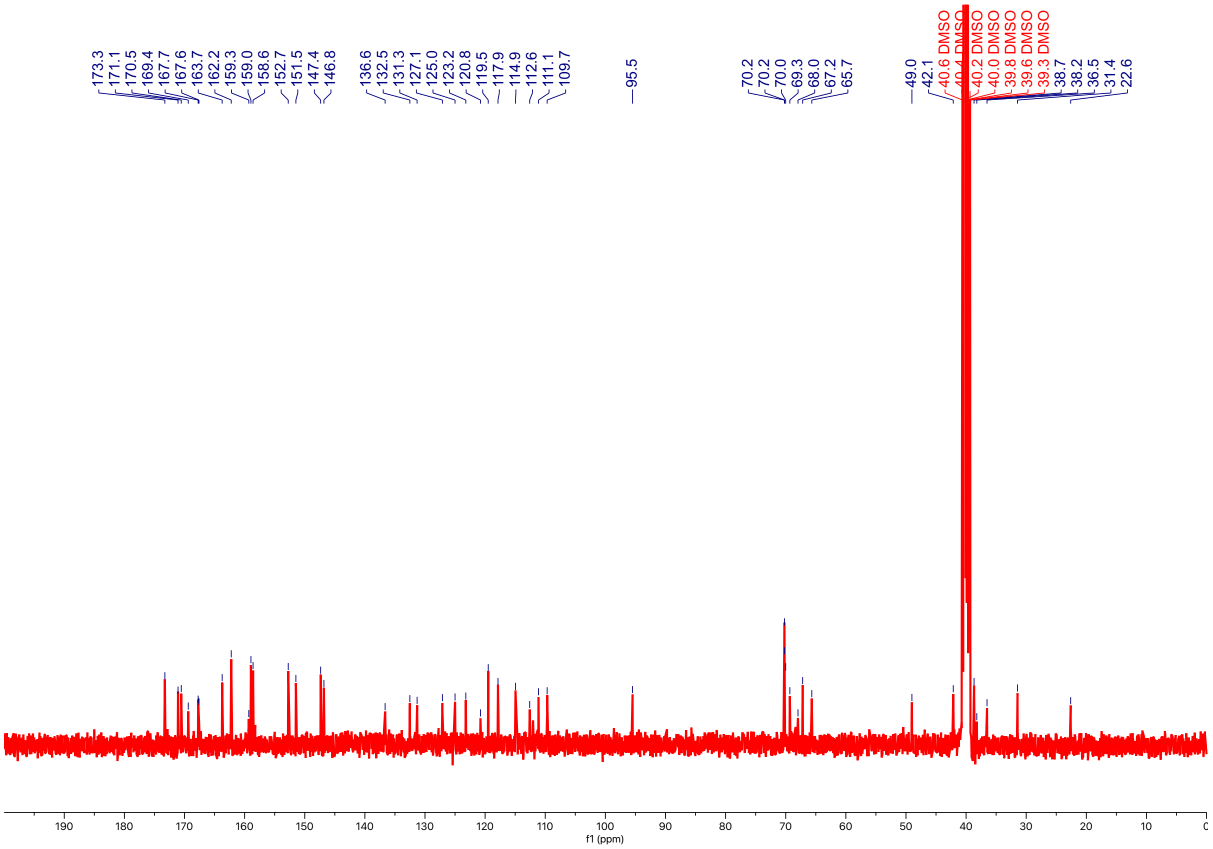

173.3
171.1
170.5
169.4
167.7
167.6
163.7
162.2
159.3
159.0
158.6
152.7
151.5
147.4
146.8
136.6
132.5
131.3
127.1
125.0
123.2
120.8
119.5
117.9
114.9
112.6
111.1
109.7
95.5
70.2
70.2
70.0
69.3
68.0
67.2
65.7
49.0
42.1
40.6 DMSO
40.2 DMSO
40.0 DMSO
39.8 DMSO
39.6 DMSO
39.3 DMSO
38.7
38.2
36.5
31.4
22.6
190 180 170 160 150 140 130 120 110 100 90 80 70 60 50 40 30 20 10
f1 (ppm)

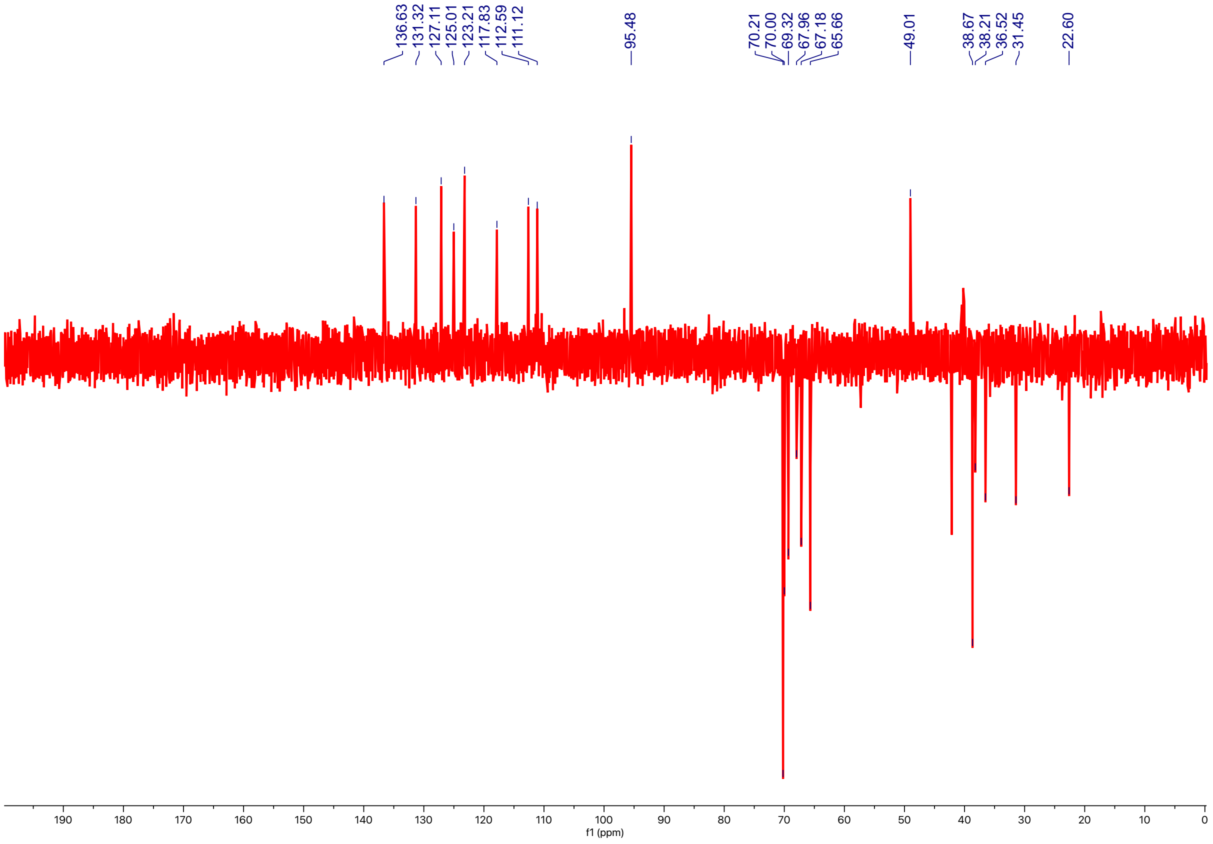

136.63
131.32
127.11
125.01
123.21
117.83
112.59
111.12
95.48
70.21
70.00
69.32
67.96
67.18
65.66
49.01
38.67
38.21
36.52
31.45
22.60
190 180 170 160 150 140 130 120 110 100 90 80 70 60 50 40 30 20 10 0
f1 (ppm)

G4L-PROTAC7

***N*^{2},*N*^{6}-bis(4-(2-aminoethoxy)quinolin-2-yl)-4-((1-((2-(2,6-dioxopiperidin-3-yl)-1,3-dioxoisoindolin-5-yl)amino)-15-oxo-3,6,9,12-tetraoxa-16-azaoctadecan-18-yl)oxy)pyridine-2,6-dicarboxamide**

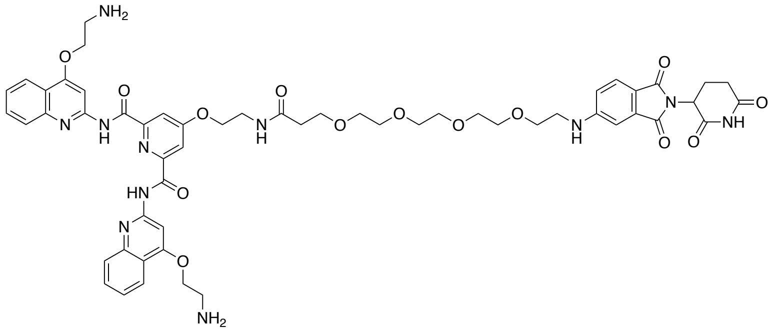

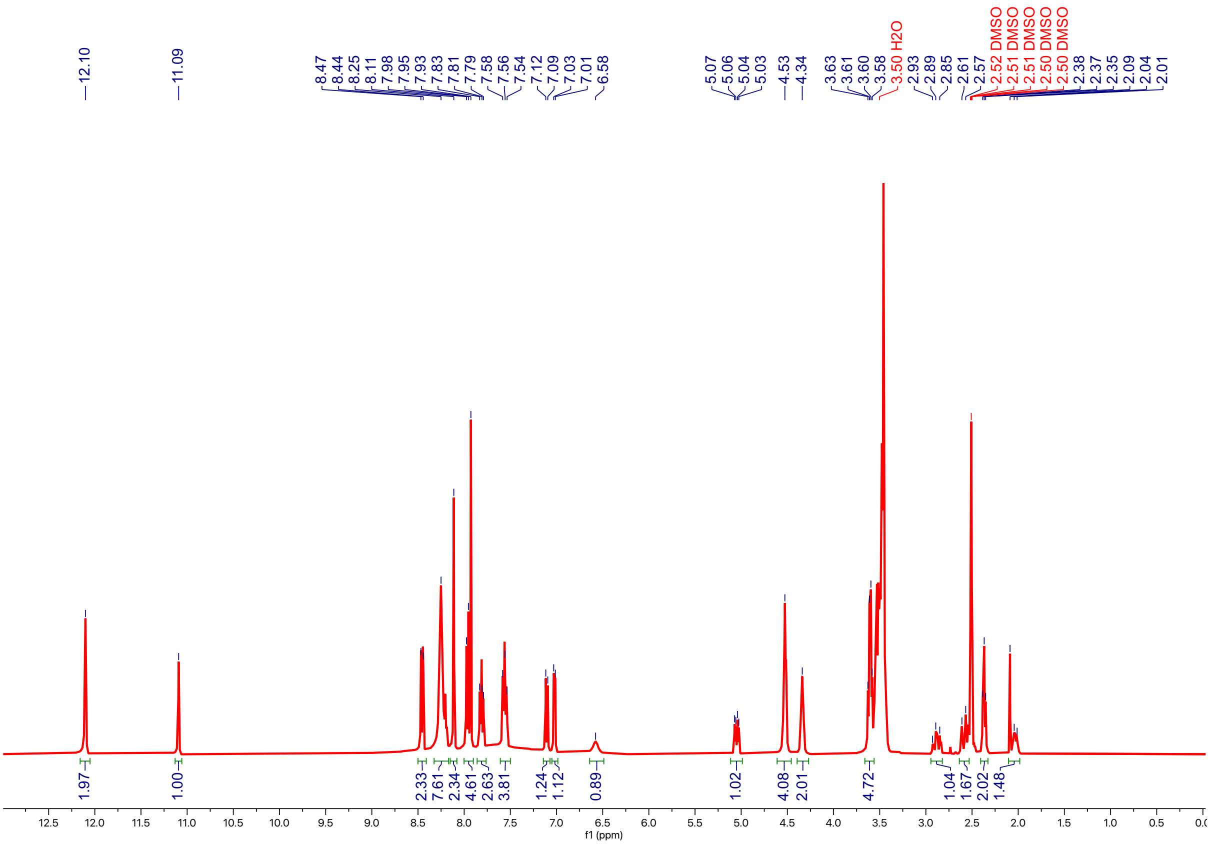

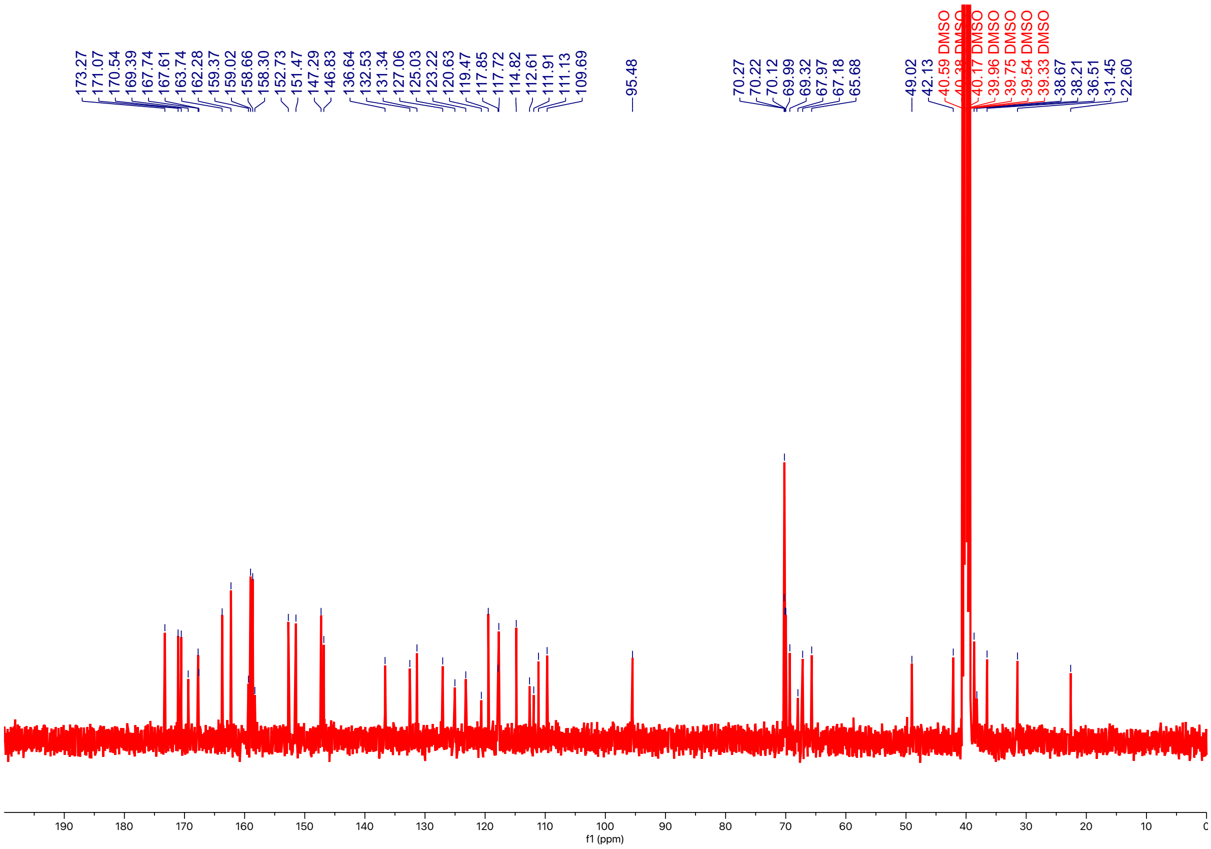
173.27
171.07
170.54
169.39
167.74
167.61
163.74
162.28
159.37
159.02
158.66
158.30
152.73
151.47
147.29
146.83
136.64
132.53
131.34
127.06
125.03
123.22
120.63
119.47
117.85
117.72
114.82
112.61
111.91
111.13
109.69
95.48
70.27
70.22
70.12
69.99
69.32
67.97
67.18
65.68
49.02
42.13
40.59 DMSO
40.38 DMSO
40.17 DMSO
39.96 DMSO
39.75 DMSO
39.54 DMSO
39.33 DMSO
38.67
38.21
36.51
31.45
22.60
f1 (ppm)

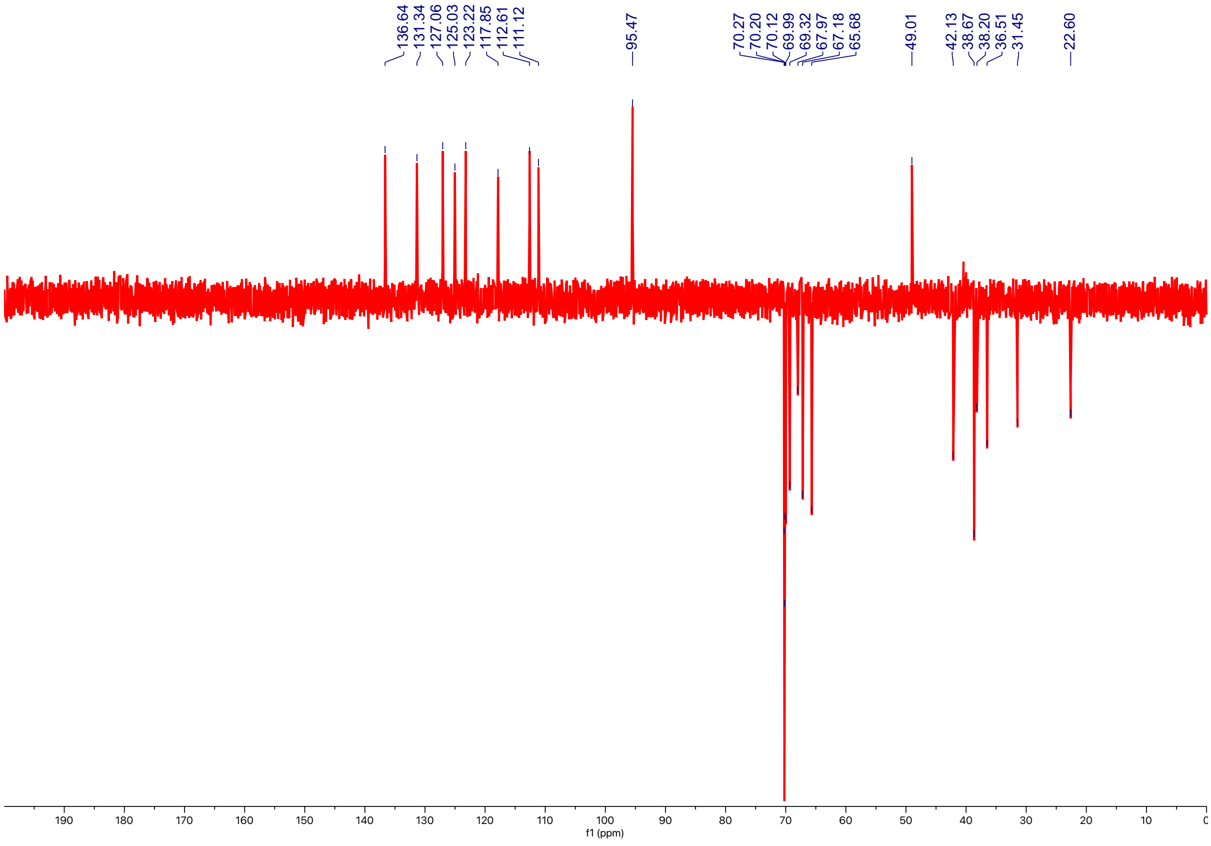
136.64
131.34
127.06
125.03
123.22
117.85
112.61
111.12
95.47
70.27
70.20
70.12
69.99
69.32
67.97
67.18
65.68
49.01
42.13
38.67
38.20
36.51
31.45
22.60
f1 (ppm)

G4L-PROTAC8

***N*[2],*N*[6]-bis(4-(2-aminoethoxy)quinolin-2-yl)-4-((1-((2-(2,6-dioxopiperidin-3-yl)-1,3-dioxoisoindolin-4-yl)amino)-18-oxo-3,6,9,12,15-pentaoxa-19-azahenicosan-21-yl)oxy)pyridine-2,6-dicarboxamide**

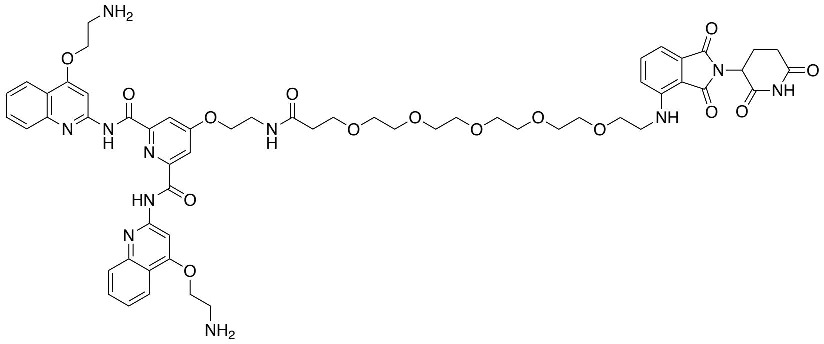

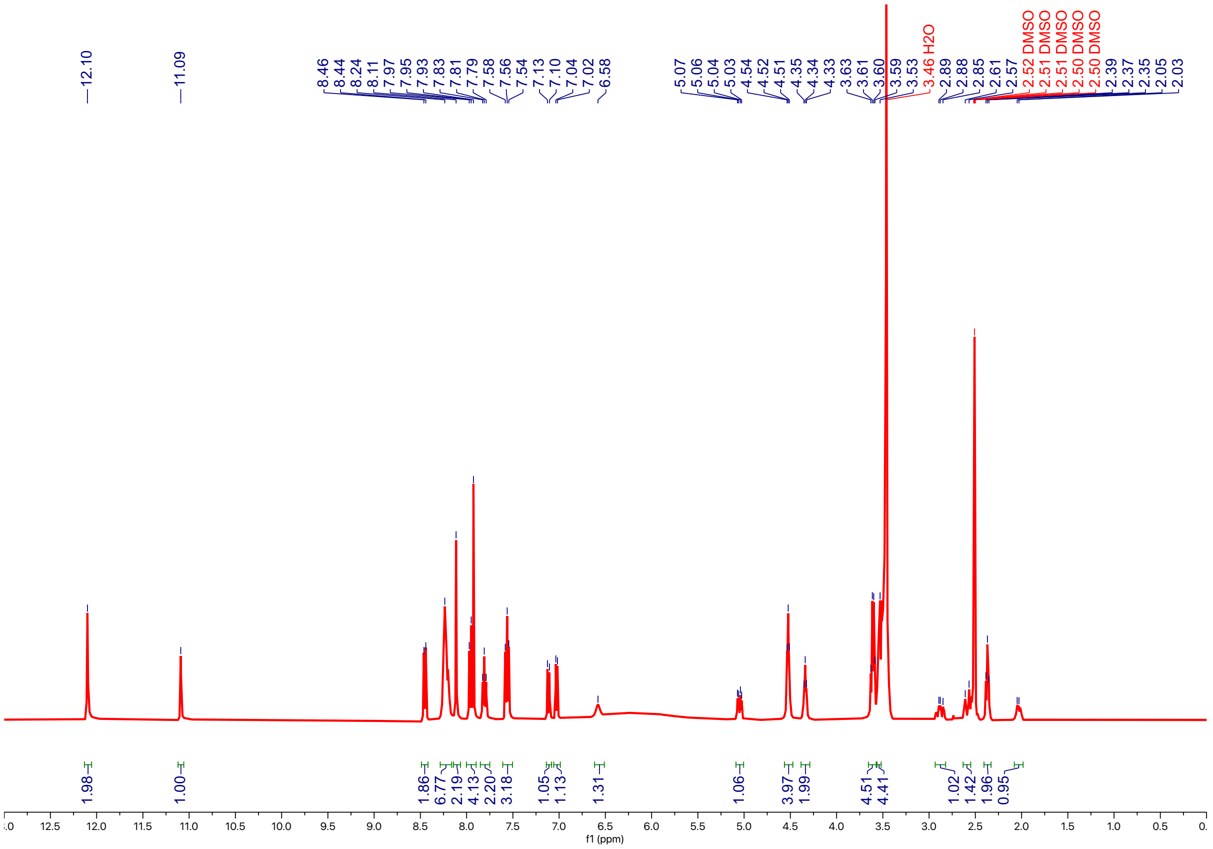

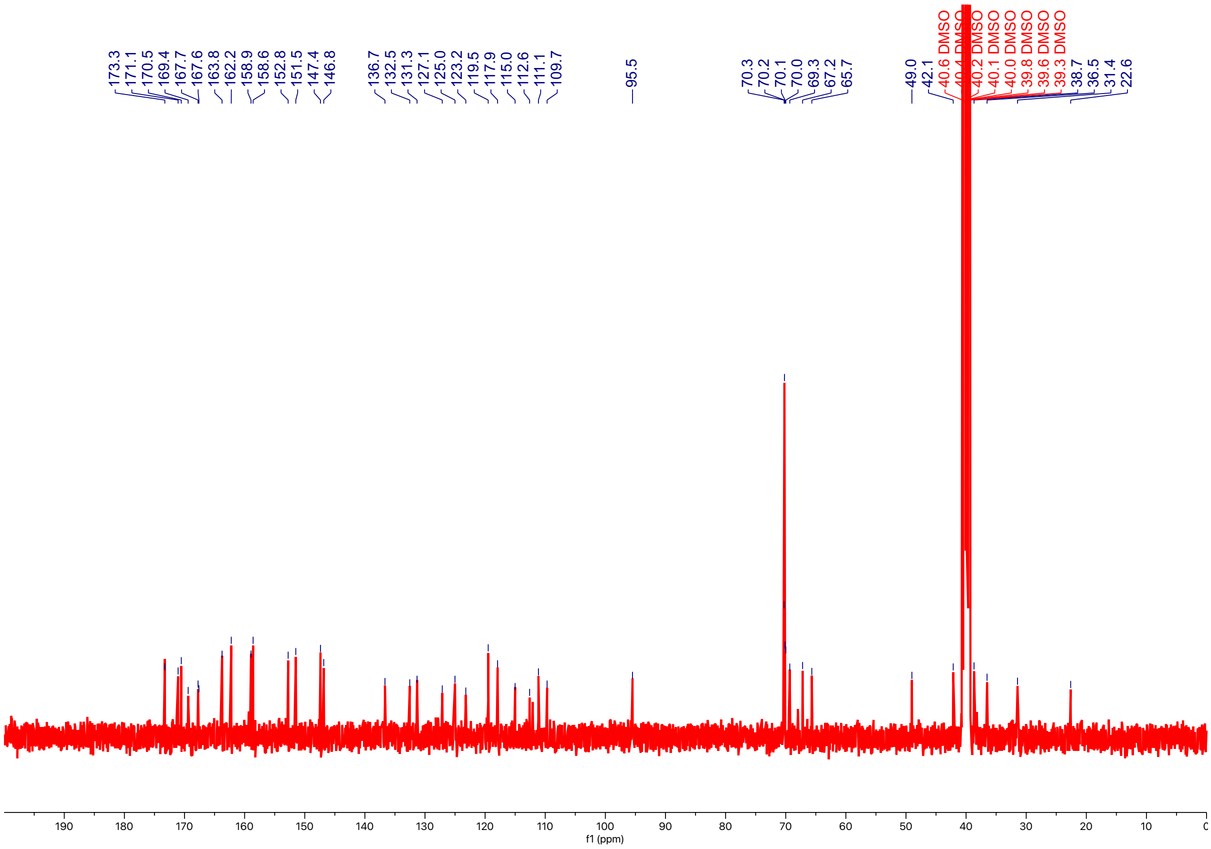
173.3
171.1
170.5
169.4
167.7
167.6
163.8
162.2
158.9
158.6
152.8
151.5
147.4
146.8
136.7
132.5
131.3
127.1
125.0
123.2
119.5
117.9
115.0
112.6
111.1
109.7
95.5
70.3
70.2
70.1
70.0
69.3
67.2
65.7
49.0
42.1
40.6 DMSO
40.2 DMSO
40.1 DMSO
40.0 DMSO
39.8 DMSO
39.6 DMSO
39.3 DMSO
38.7
36.5
31.4
22.6
190 180 170 160 150 140 130 120 110 100 90 80 70 60 50 40 30 20 10 0
f1 (ppm)

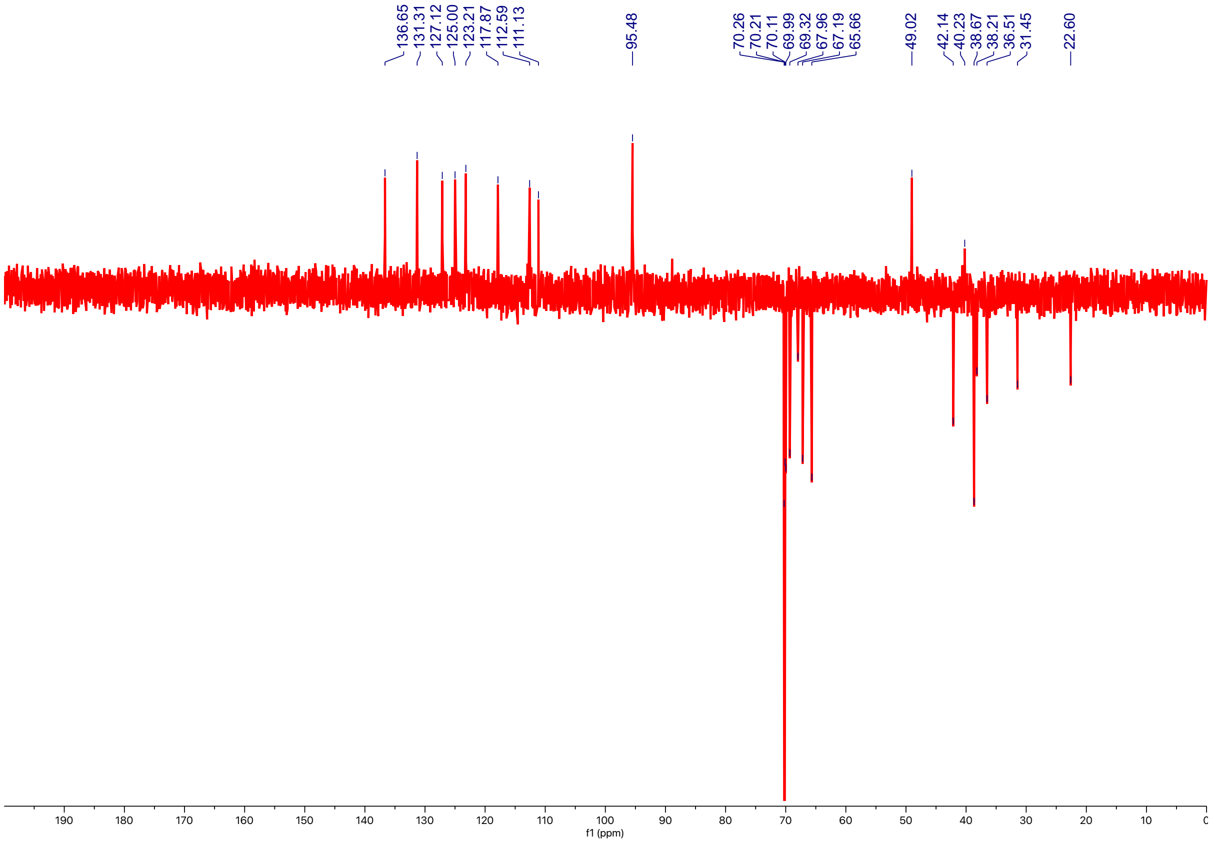
136.65
131.31
127.12
125.00
123.21
117.87
112.59
111.13
95.48
70.26
70.21
70.11
69.99
69.32
67.96
67.19
65.66
49.02
42.14
40.23
38.67
38.21
36.51
31.45
22.60
190 180 170 160 150 140 130 120 110 100 90 80 70 60 50 40 30 20 10 0
f1 (ppm)

G4L-PROTAC9

***N*[2],*N*[6]-bis(4-(2-aminoethoxy)quinolin-2-yl)-4-((1-((2-(2,6-dioxopiperidin-3-yl)-1,3-dioxoisoindolin-4-yl)amino)-27-oxo-3,6,9,12,15,18,21,24-octaoxa-28-azatriacontan-30-yl)oxy)pyridine-2,6-dicarboxamide**

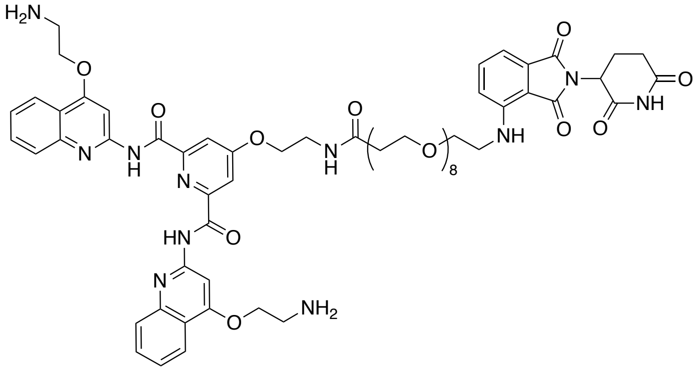

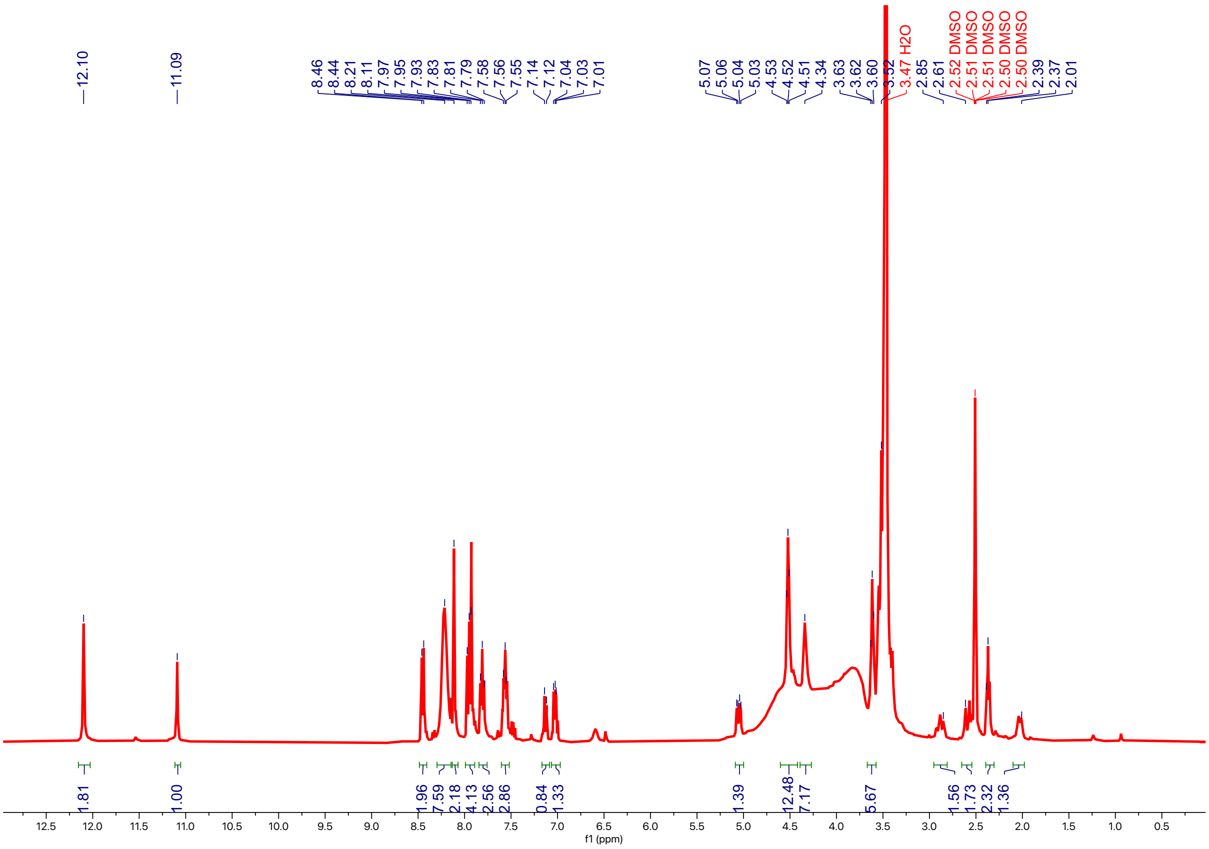

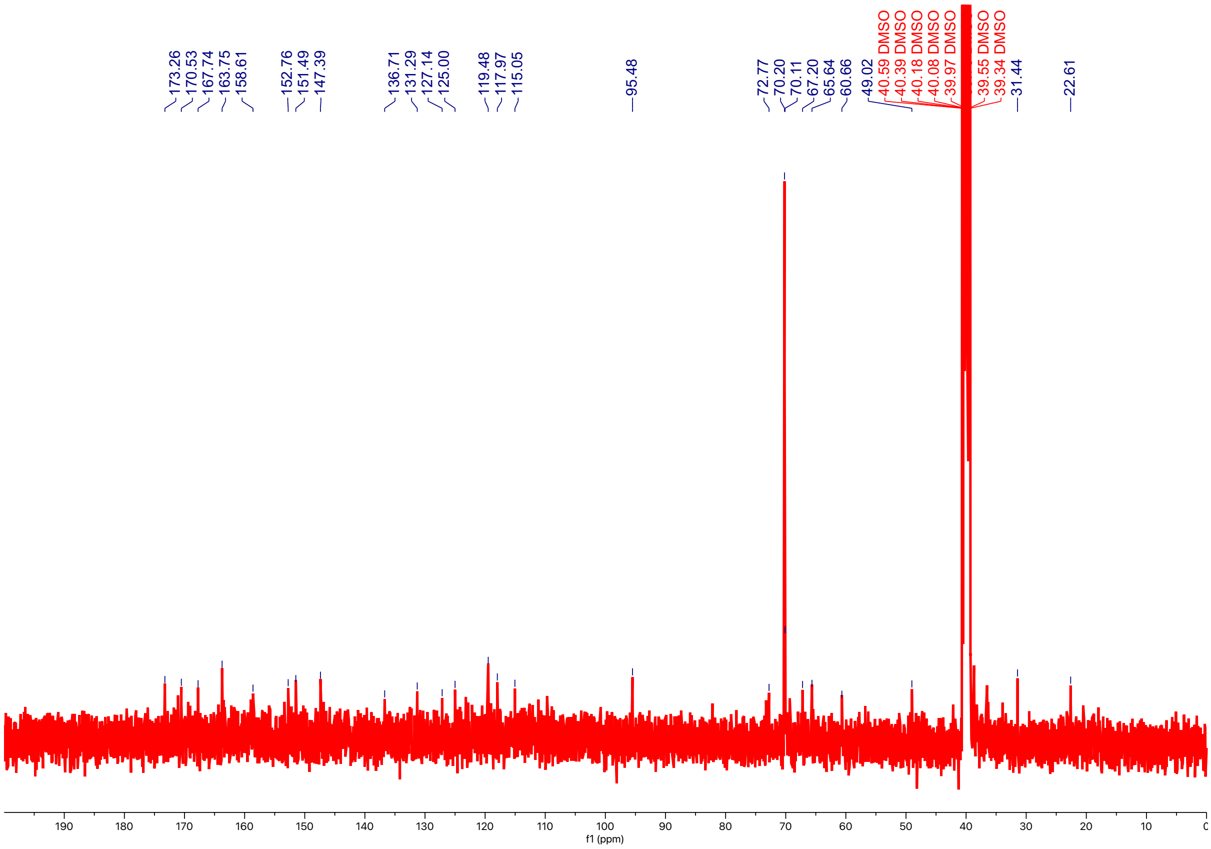
173.26
170.53
167.74
163.75
158.61
152.76
151.49
147.39
136.71
131.29
127.14
125.00
119.48
117.97
115.05
95.48
72.77
70.20
70.11
67.20
65.64
60.66
49.02
40.59 DMSO
40.39 DMSO
40.18 DMSO
40.08 DMSO
39.97 DMSO
39.55 DMSO
39.34 DMSO
31.44
22.61
f1 (ppm)

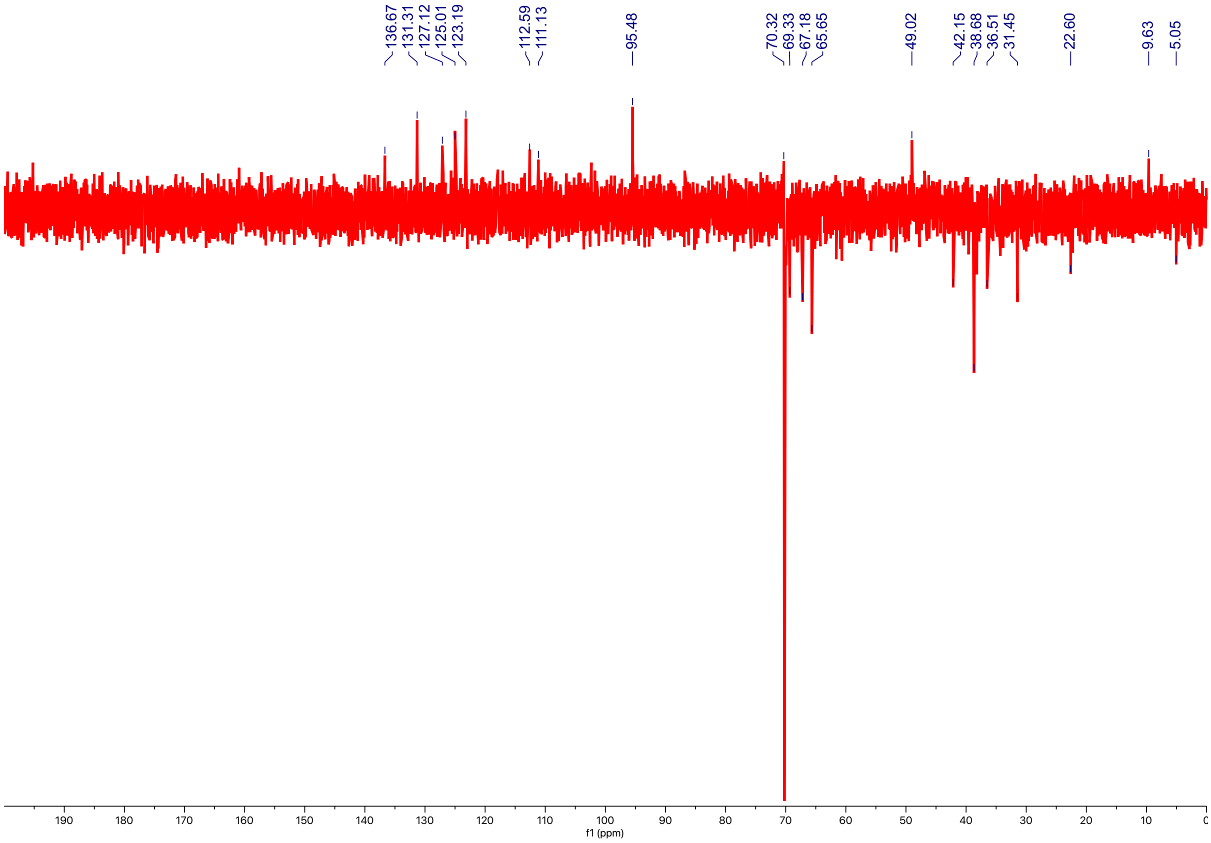
136.67
131.31
127.12
125.01
123.19
112.59
111.13
95.48
70.32
69.33
67.18
65.65
49.02
42.15
38.68
36.51
31.45
22.60
9.63
5.05
f1 (ppm)

G4L-PROTAC10

***N*²,*N*⁶-bis(4-(2-aminoethoxy)quinolin-2-yl)-4-((26-((2-(2,6-dioxopiperidin-3-yl)-1,3-dioxoisoindolin-4-yl)amino)-4,23-dioxo-6,12,15,21-tetraoxa-3,24-diazahexacosyl)oxy)pyridine-2,6-dicarboxamide**

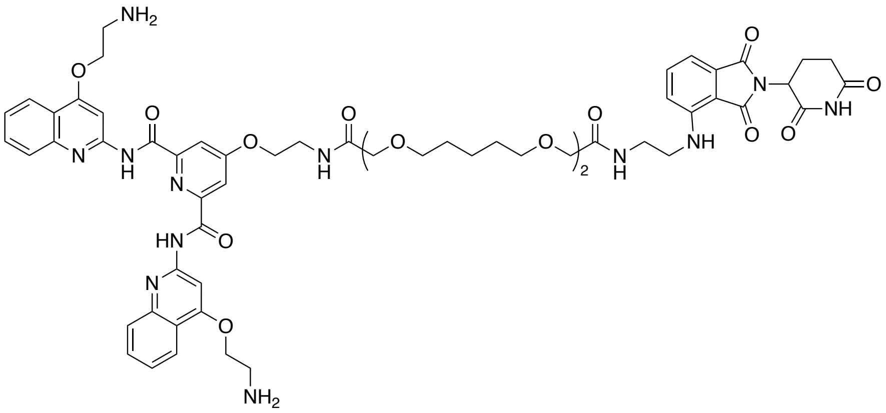

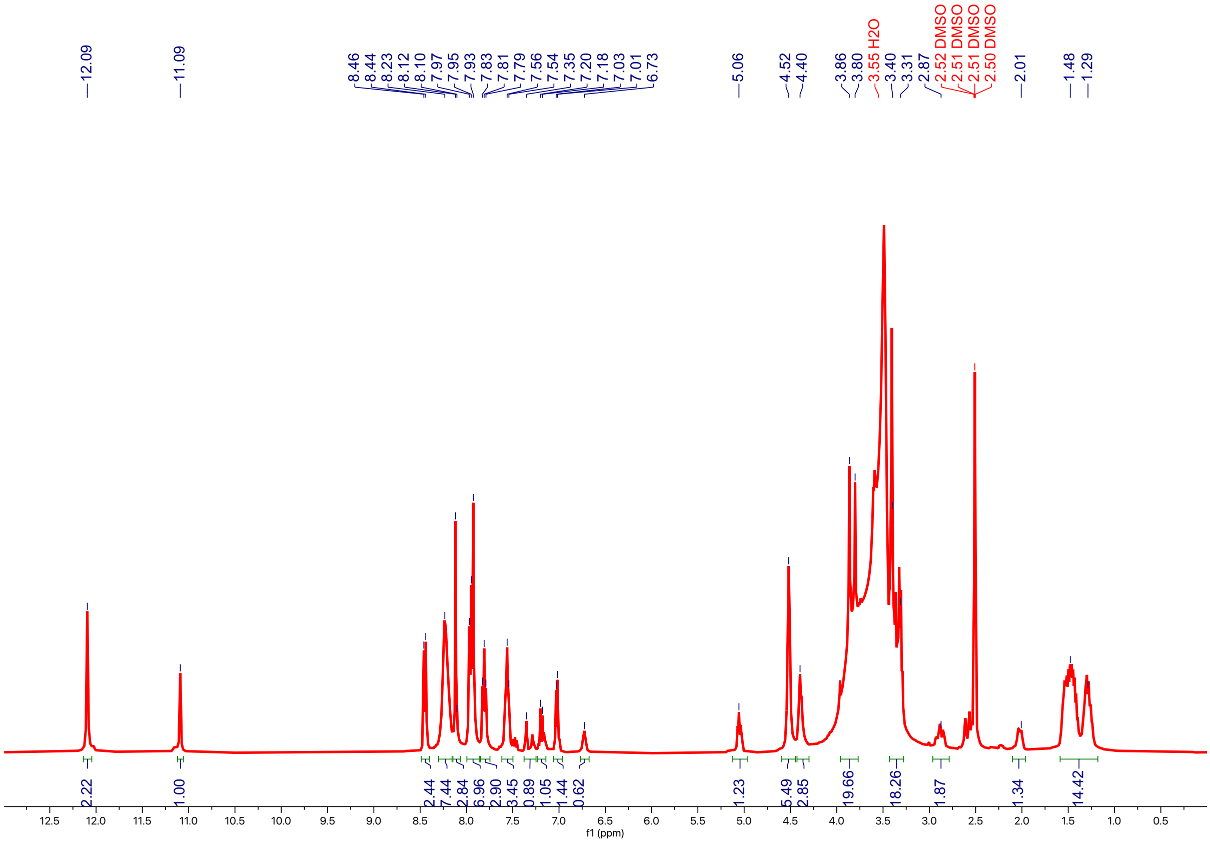

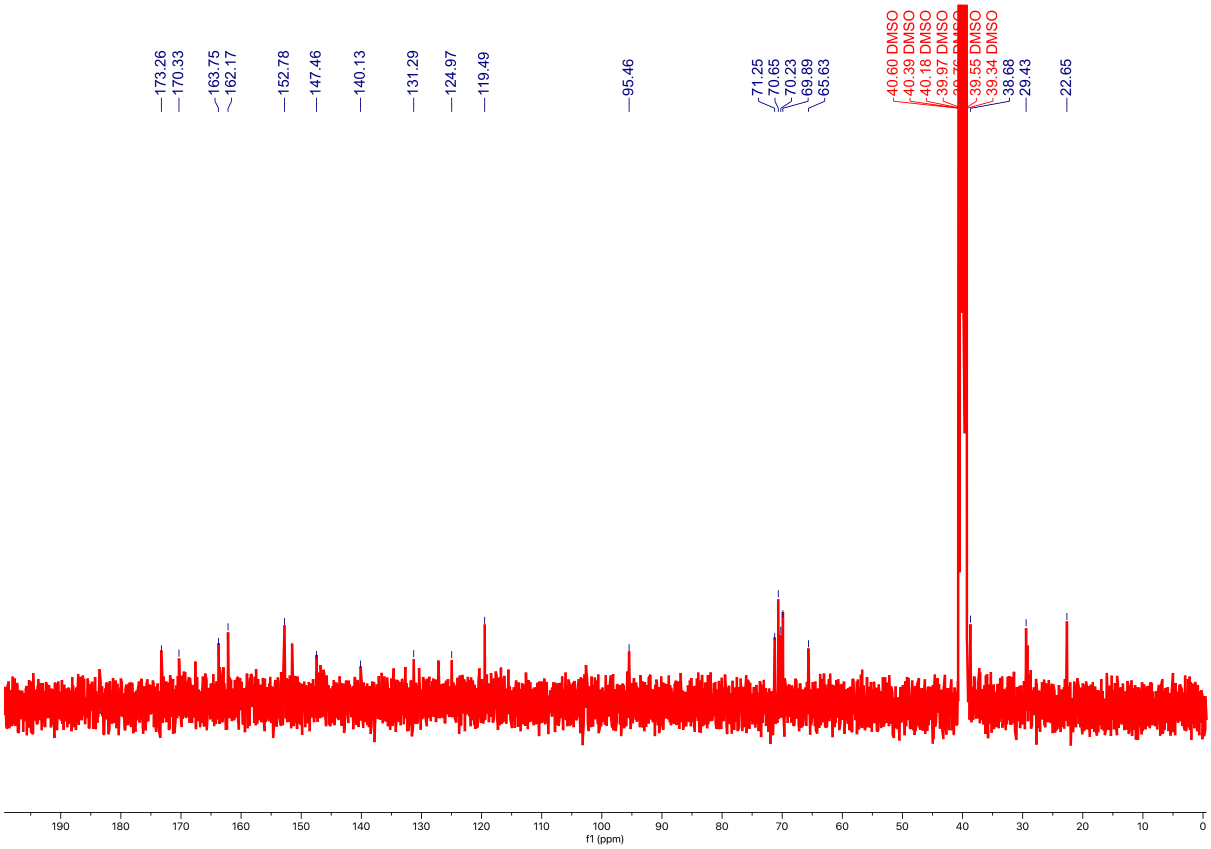
173.26
170.33
163.75
162.17
152.78
147.46
140.13
131.29
124.97
119.49
95.46
71.25
70.65
70.23
69.89
65.63
40.60 DMSO
40.39 DMSO
40.18 DMSO
39.97 DMSO
39.55 DMSO
39.34 DMSO
38.68
29.43
22.65
190 180 170 160 150 140 130 120 110 100 90 80 70 60 50 40 30 20 10 0
f1 (ppm)

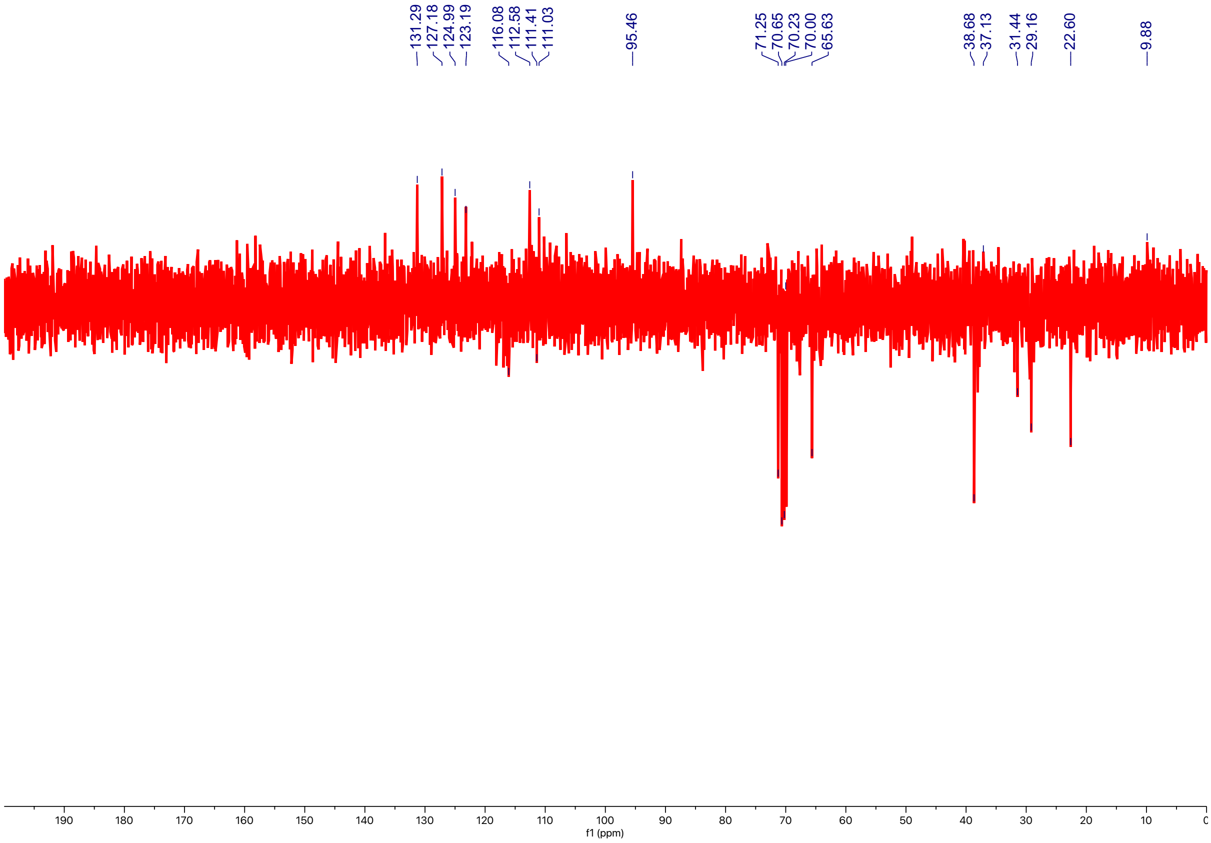
131.29
127.18
124.99
123.19
116.08
112.58
111.41
111.03
95.46
71.25
70.65
70.23
70.00
65.63
38.68
37.13
31.44
29.16
22.60
9.88
190 180 170 160 150 140 130 120 110 100 90 80 70 60 50 40 30 20 10
f1 (ppm)

G4L-PROTAC11

N^2,N^6-bis(4-(2-aminoethoxy)quinolin-2-yl)-4-((1-(4-(2-(2,6-dioxopiperidin-3-yl)-1,3-dioxoisoindolin-5-yl)piperazin-1-yl)-12-oxo-3,6,9-trioxa-13-azapentadecan-15-yl)oxy)pyridine-2,6-dicarboxamide

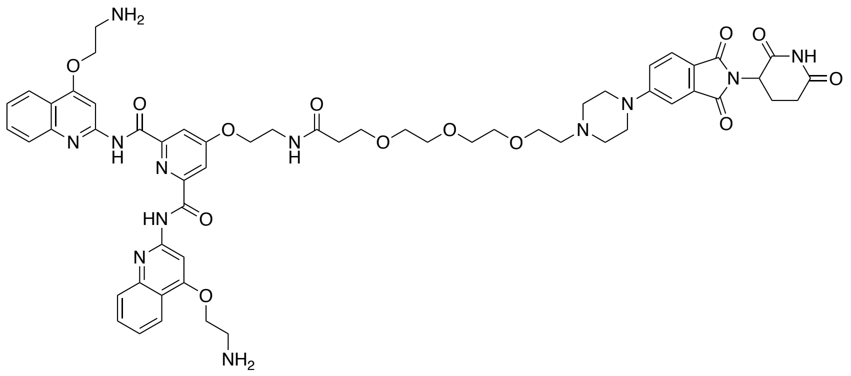

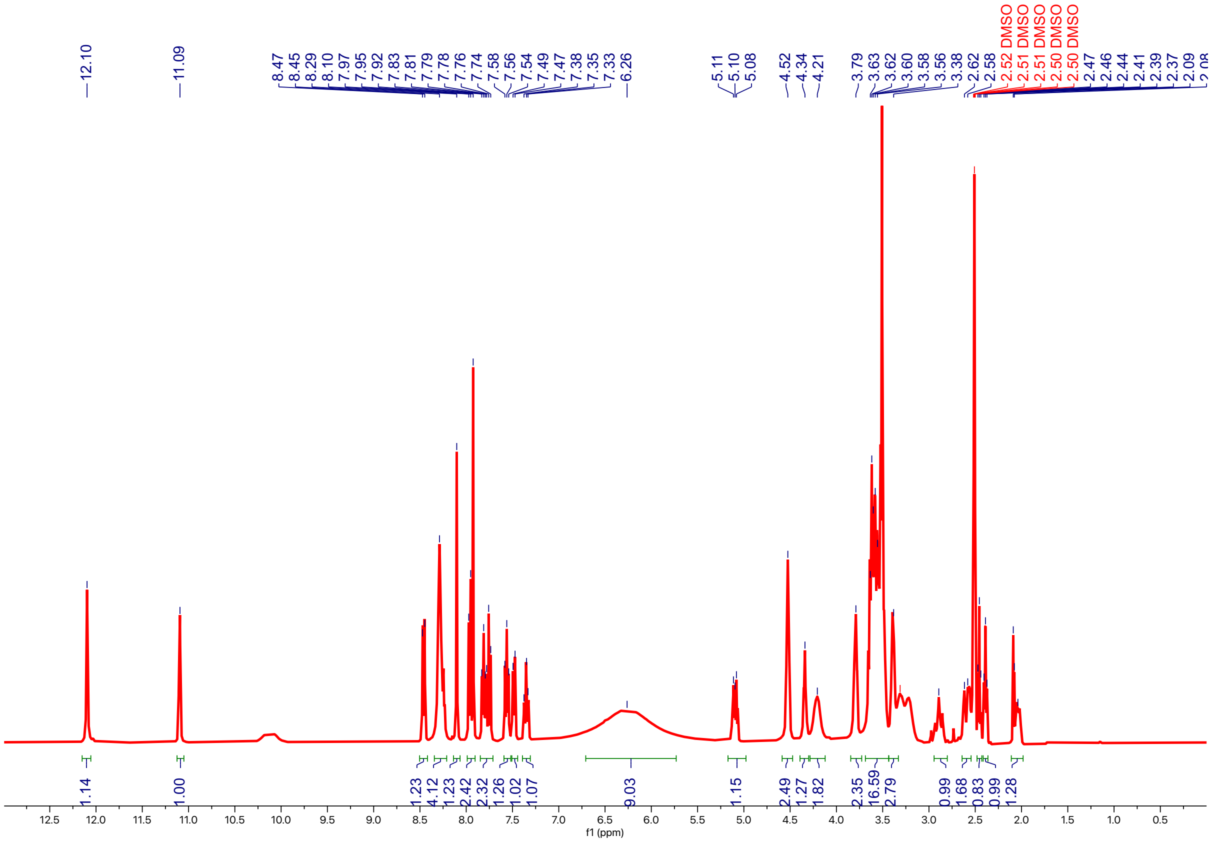

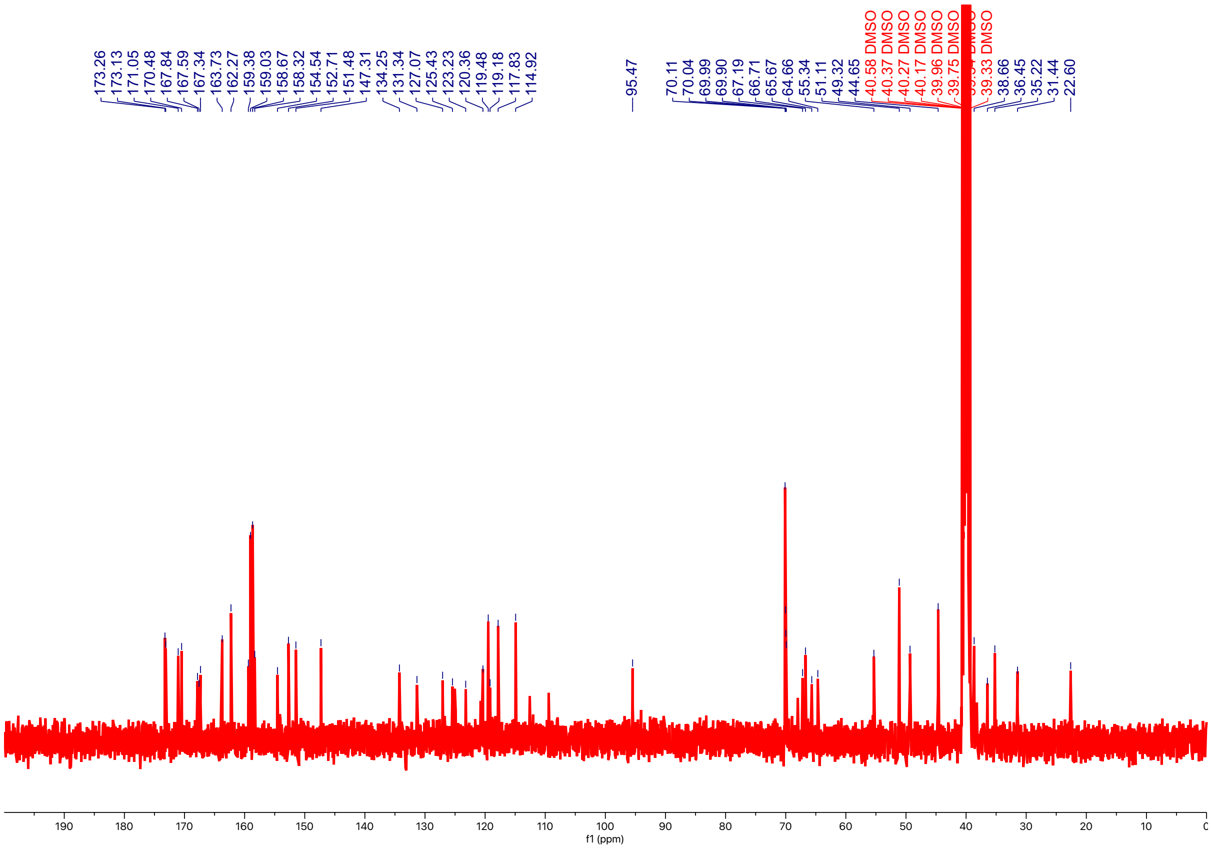
173.26
173.13
171.05
170.48
167.84
167.59
167.34
163.73
162.27
159.38
159.03
158.67
158.32
154.54
152.71
151.48
147.31
134.25
131.34
127.07
125.43
123.23
120.36
119.48
119.18
117.83
114.92
95.47
70.11
70.04
69.99
69.90
67.19
66.71
65.67
64.66
55.34
51.11
49.32
44.65
40.58 DMSO
40.37 DMSO
40.27 DMSO
40.17 DMSO
39.96 DMSO
39.75 DMSO
39.33 DMSO
38.66
36.45
35.22
31.44
22.60
190
180
170
160
150
140
130
120
110
100
90
80
70
60
50
40
30
20
10
f1 (ppm)

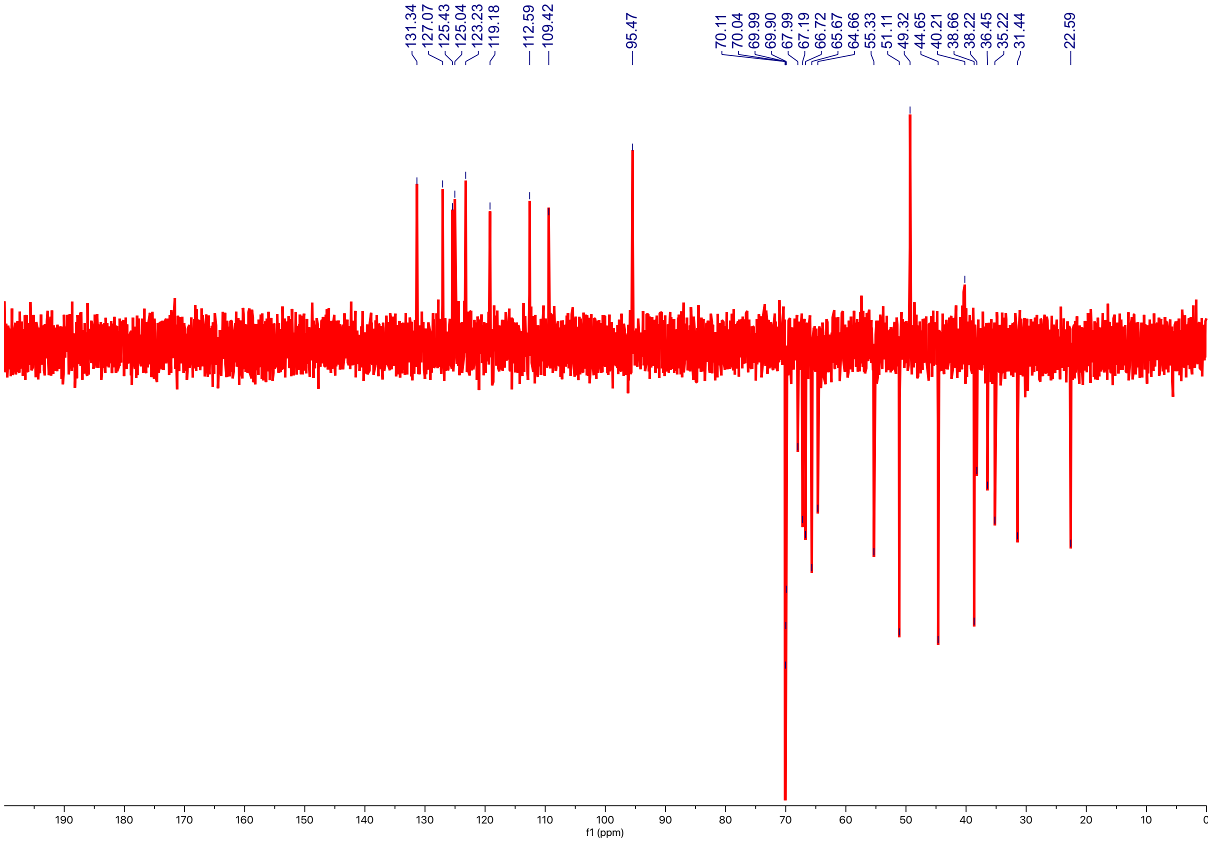
131.34
127.07
125.43
125.04
123.23
119.18
112.59
109.42
95.47
70.11
70.04
69.99
69.90
67.99
67.19
66.72
65.67
64.66
55.33
51.11
49.32
44.65
40.21
38.66
38.22
36.45
35.22
31.44
22.59
190
180
170
160
150
140
130
120
110
100
90
80
70
60
50
40
30
20
10
f1 (ppm)

G4L-PROTAC12

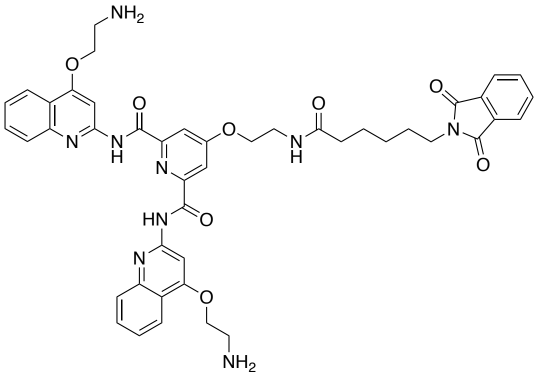

***N*²,*N*⁶-bis(4-(2-aminoethoxy)quinolin-2-yl)-4-(2-(6-(1,3-dioxoisoindolin-2-yl)hexanamido)ethoxy)pyridine-2,6-dicarboxamide**

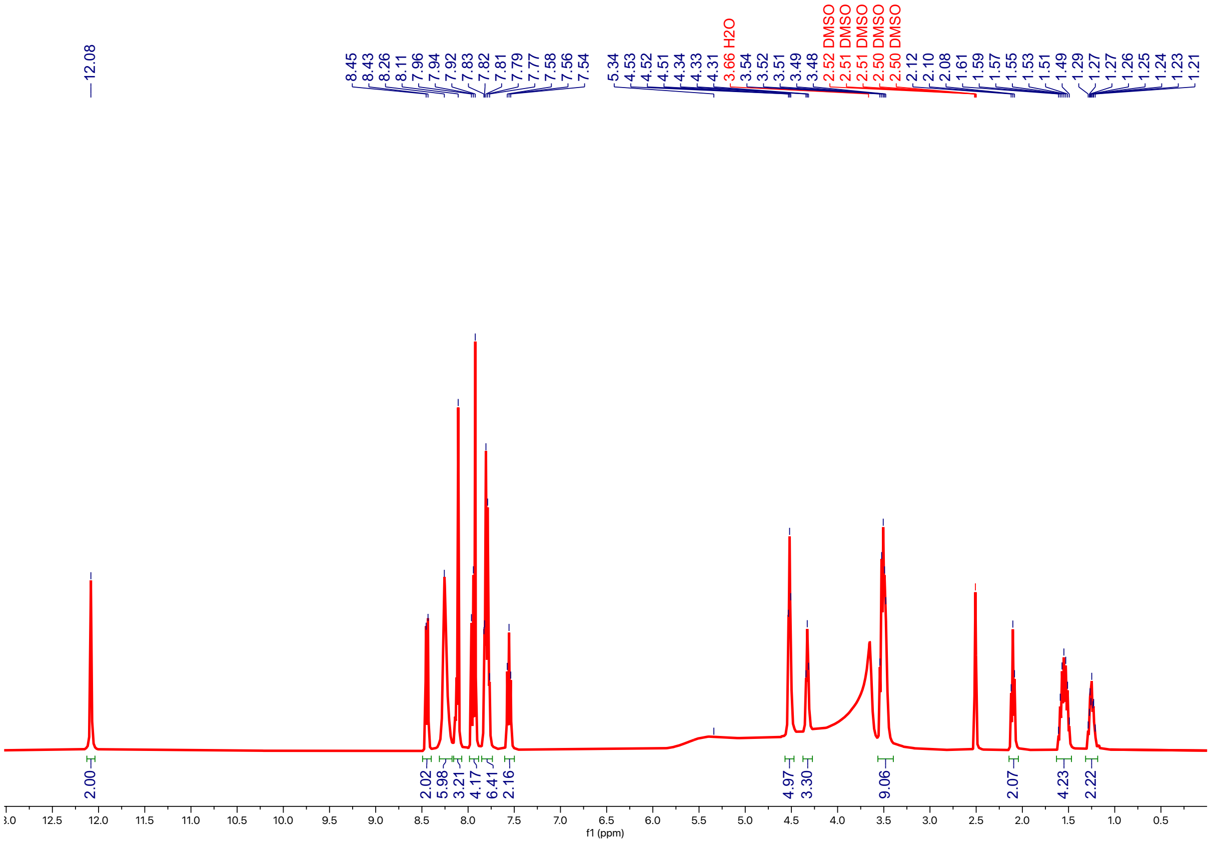

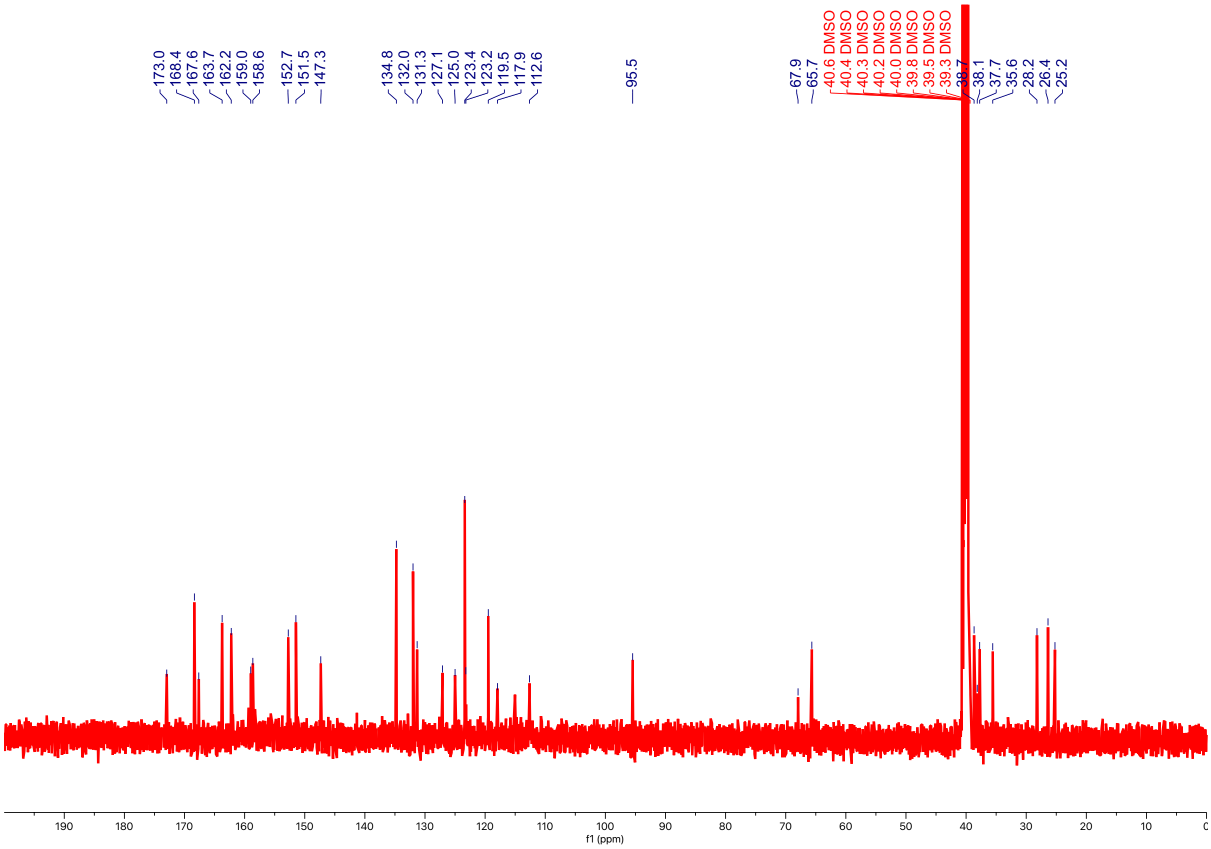
173.0
168.4
167.6
163.7
162.2
159.0
158.6
152.7
151.5
147.3
134.8
132.0
131.3
127.1
125.0
123.4
123.2
119.5
117.9
112.6
95.5
67.9
65.7
40.6 DMSO
40.4 DMSO
40.3 DMSO
40.2 DMSO
40.0 DMSO
39.8 DMSO
39.5 DMSO
39.3 DMSO
38.7
38.1
37.7
35.6
28.2
26.4
25.2
f1 (ppm)

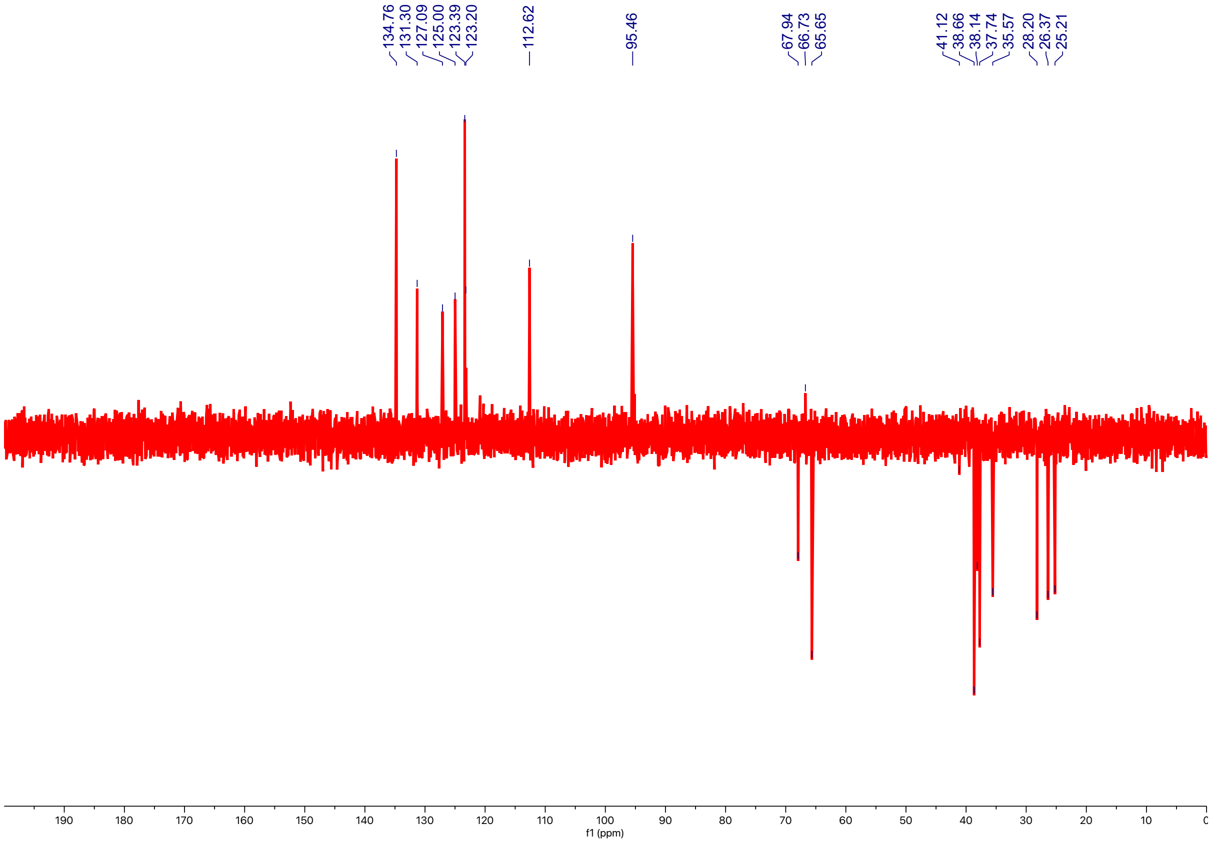
134.76
131.30
127.09
125.00
123.39
123.20
112.62
95.46
67.94
66.73
65.65
41.12
38.66
38.14
37.74
35.57
28.20
26.37
25.21
f1 (ppm)

G4L-PROTAC13

4-(2-(5-(2-((2-(2,6-dioxopiperidin-3-yl)-1,3-dioxoisoindolin-4-yl)oxy)acetamido)pentanamido)ethoxy)-*N*2,*N*6-bis(4-(2-(pyrrolidin-1-yl)ethoxy)quinolin-2-yl)pyridine-2,6-dicarboxamide

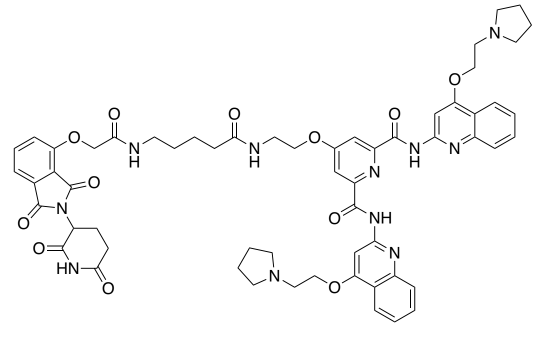

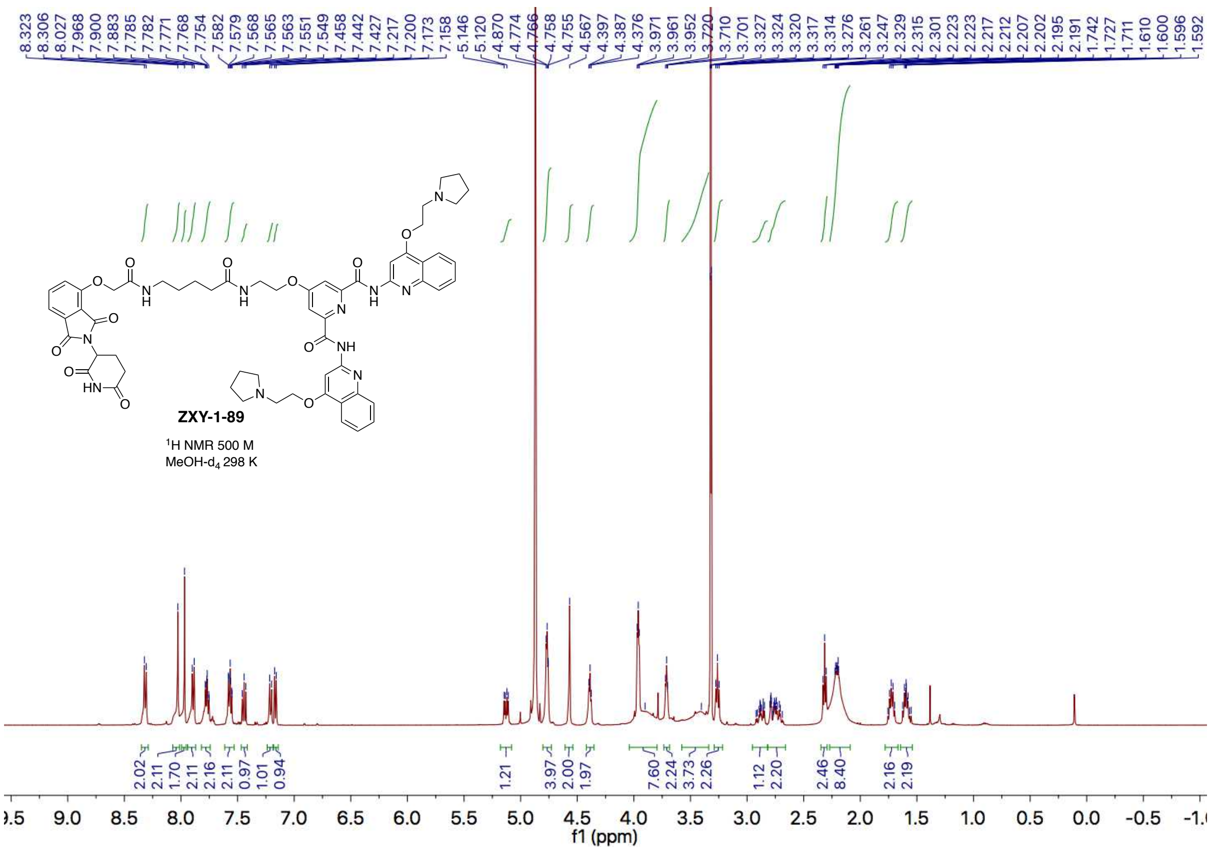

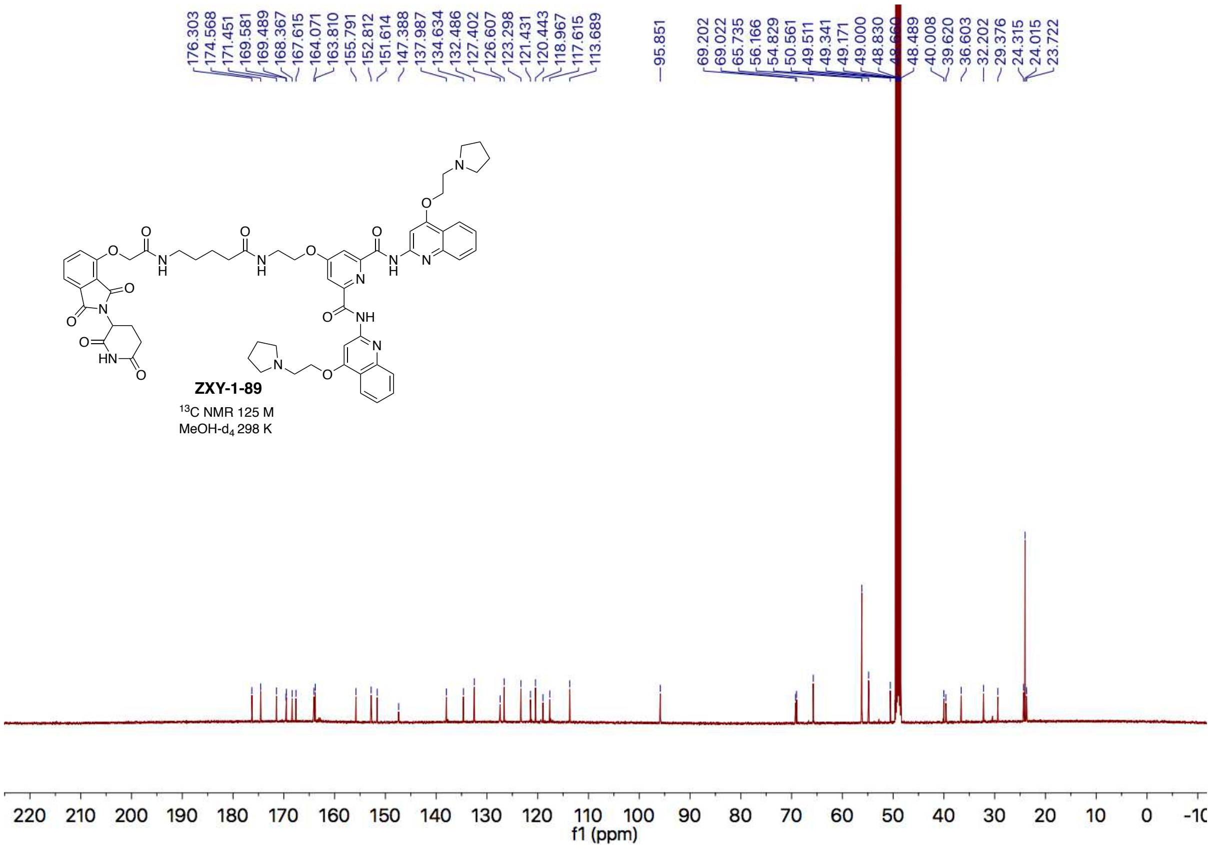
176.303
174.568
171.451
169.581
169.489
168.367
167.615
164.071
163.810
155.791
152.812
151.614
147.388
137.987
134.634
132.486
127.402
126.607
123.298
121.431
120.443
118.967
117.615
113.689
95.851
69.202
69.022
65.735
56.166
54.829
50.561
49.511
49.341
49.171
49.000
48.830
48.489
40.008
39.620
36.603
32.202
29.376
24.315
24.015
23.722
ZXY-1-89
13C NMR 125 M
MeOH-d4 298 K
220 210 200 190 180 170 160 150 140 130 120 110 100 90 80 70 60 50 40 30 20 10 0 -10
f1 (ppm)

G4L-PROTAC14

4-(2-(6-(((*S*)-1-((2*S*,4*R*)-4-hydroxy-2-((4-(4-methylthiazol-5-yl)benzyl)carbamoyl)pyrrolidin-1-yl)-3,3-dimethyl-1-oxobutan-2-yl)amino)-6-oxohexanamido)ethoxy)-*N*2,*N*6-bis(4-(2-(pyrrolidin-1-yl)ethoxy)quinolin-2-yl)pyridine-2,6-dicarboxamide

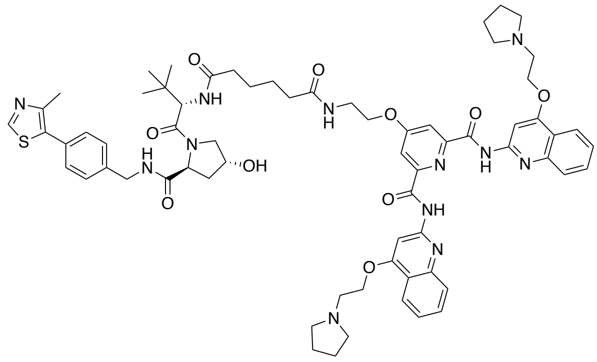

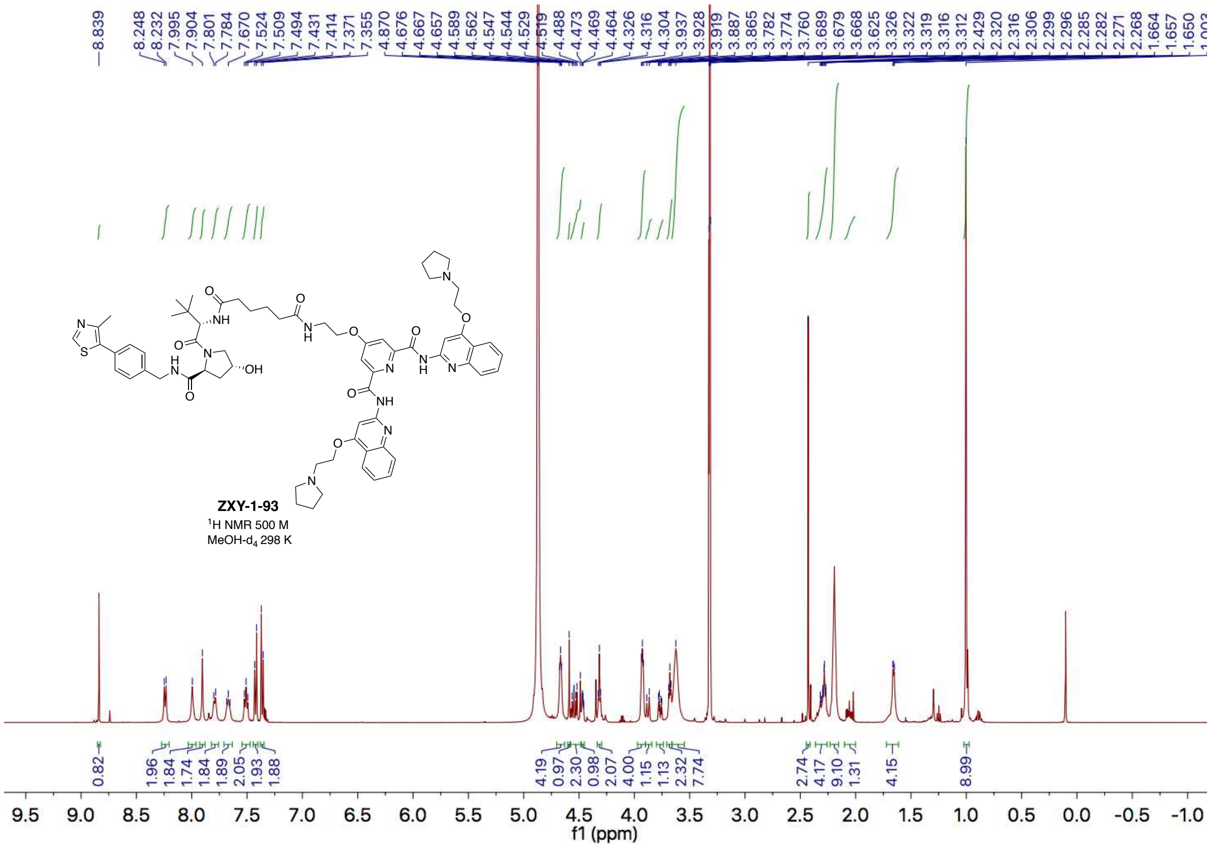

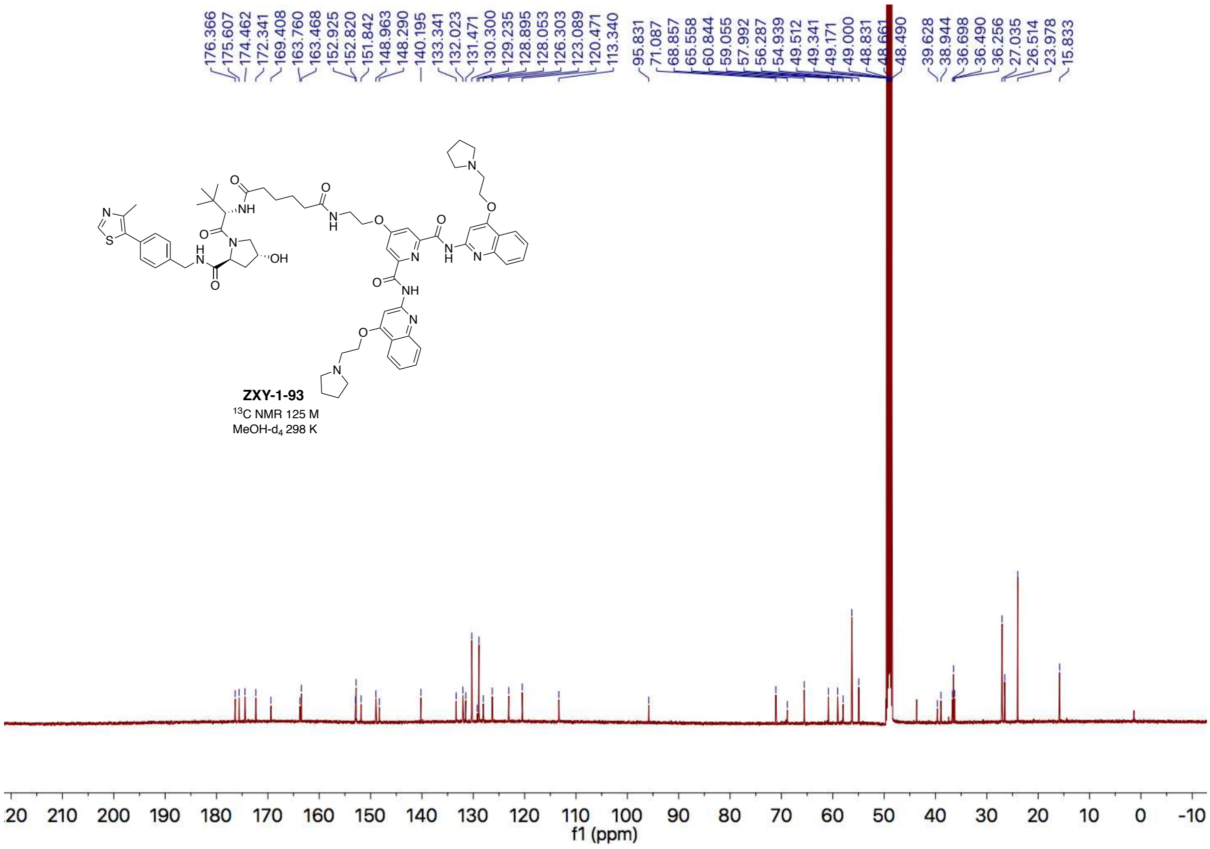
176.366
175.607
174.462
172.341
169.408
163.760
163.468
152.925
152.820
151.842
148.963
148.290
140.195
133.341
132.023
131.471
130.300
129.235
128.895
128.053
126.303
123.089
120.471
113.340
95.831
71.087
68.857
65.558
60.844
59.055
57.992
56.287
54.939
49.512
49.341
49.171
49.000
48.831
48.661
48.490
39.628
38.944
36.698
36.490
36.256
27.035
26.514
23.978
15.833
OH
NH
N
S
O
ZXY-1-93
13C NMR 125 M
MeOH-d4 298 K
20 210 200 190 180 170 160 150 140 130 120 110 100 90 80 70 60 50 40 30 20 10 0 -10
f1 (ppm)

Supplementary Figure 1. Chemical structures of G4L-PROTACs used for targeted degradation of FUS and SMARCA4 for CUT&Tag experiments.

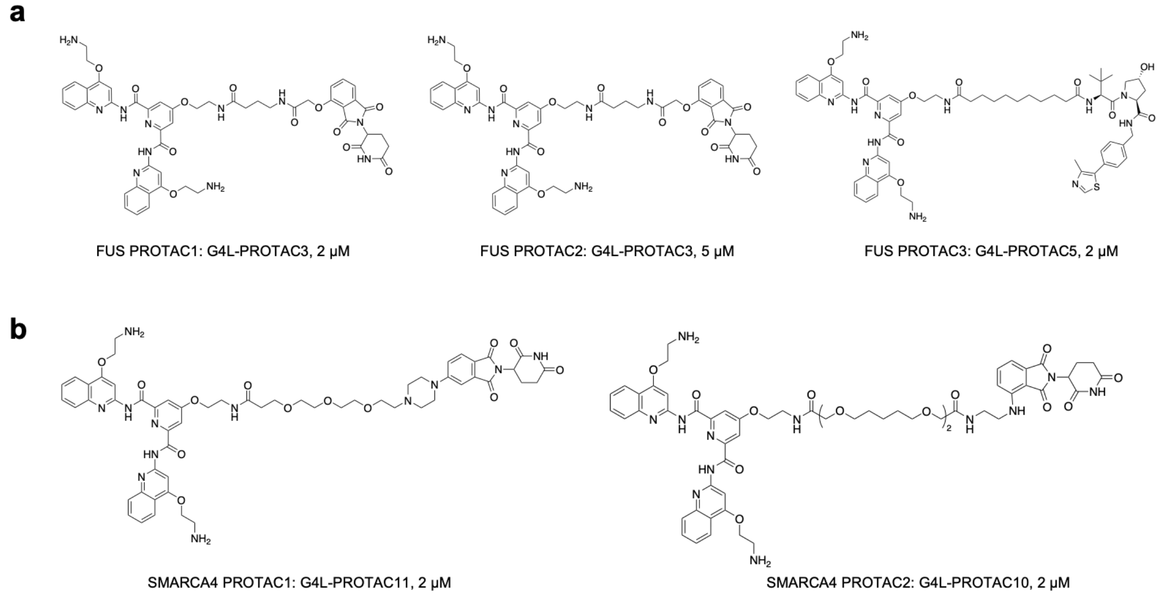

a, Structures of three G4L-PROTAC compounds used to target FUS for degradation:

G4L-PROTAC3 (FUS PROTAC1) at 2 μM,

G4L-PROTAC3 (FUS PROTAC2) at 5 μM,

G4L-PROTAC5 (FUS PROTAC3) at 2 μM.

b, Structures of two G4L-PROTACs used for SMARCA4 degradation:

G4L-PROTAC11 (SMARCA4 PROTAC1) at 2 μM,

G4L-PROTAC10 (SMARCA4 PROTAC2) at 2 μM.

Supplementary Figure 2. Sample-wise Pearson correlation of SMARCA4 and FUS CUT&Tag datasets.

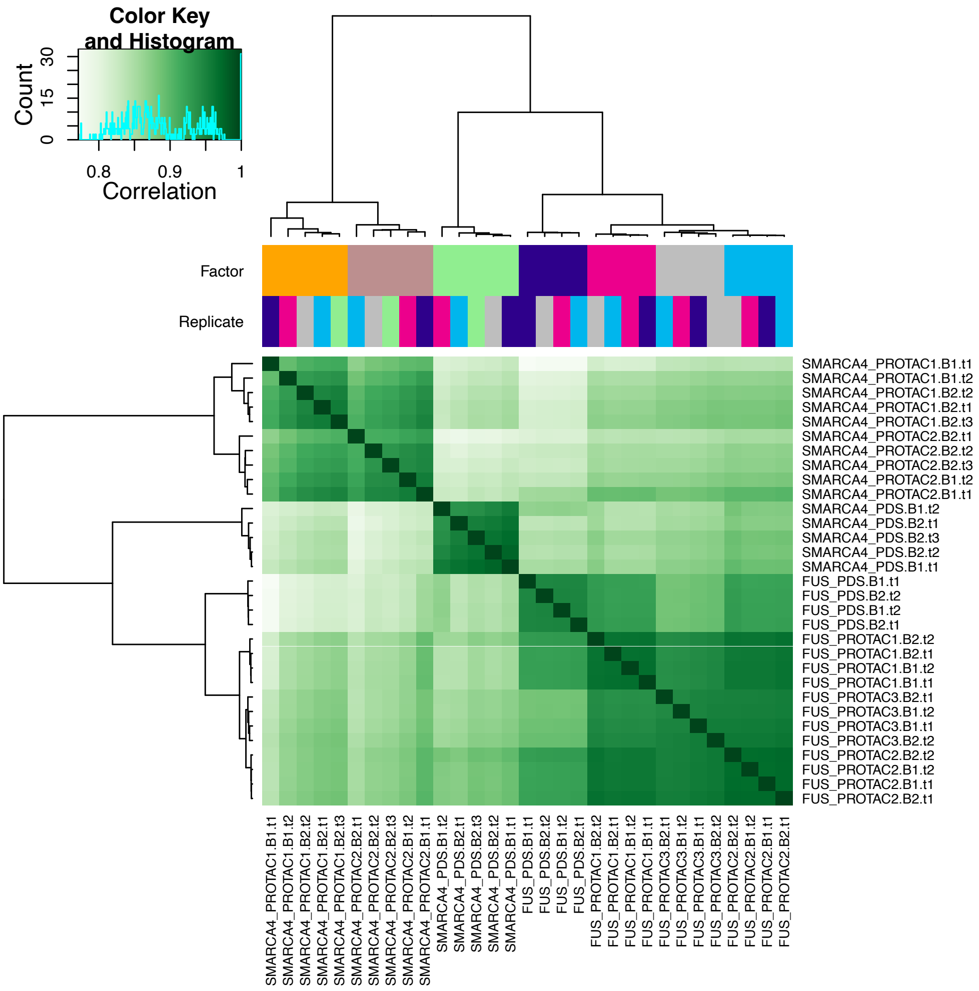

Hierarchical clustering heatmap showing Pearson correlation coefficients across all SMARCA4 and FUS CUT&Tag samples from U2OS cells treated with PDS (control) or G4L-PROTACs (PROTAC1–3 for FUS; PROTAC1–2 for SMARCA4). Columns and rows represent individual replicate datasets, color-coded by protein target (factor) and replicate batch. Samples cluster first by protein factor (SMARCA4 vs FUS) and then by treatment condition, with high within-group correlation among biological replicates.

Histogram (top left) displays the distribution of pairwise correlation values, with most correlations exceeding 0.8, indicating strong signal consistency and high data quality across conditions.

Supplementary Figure 3. Principal component analysis of SMARCA4 and FUS CUT&Tag datasets.

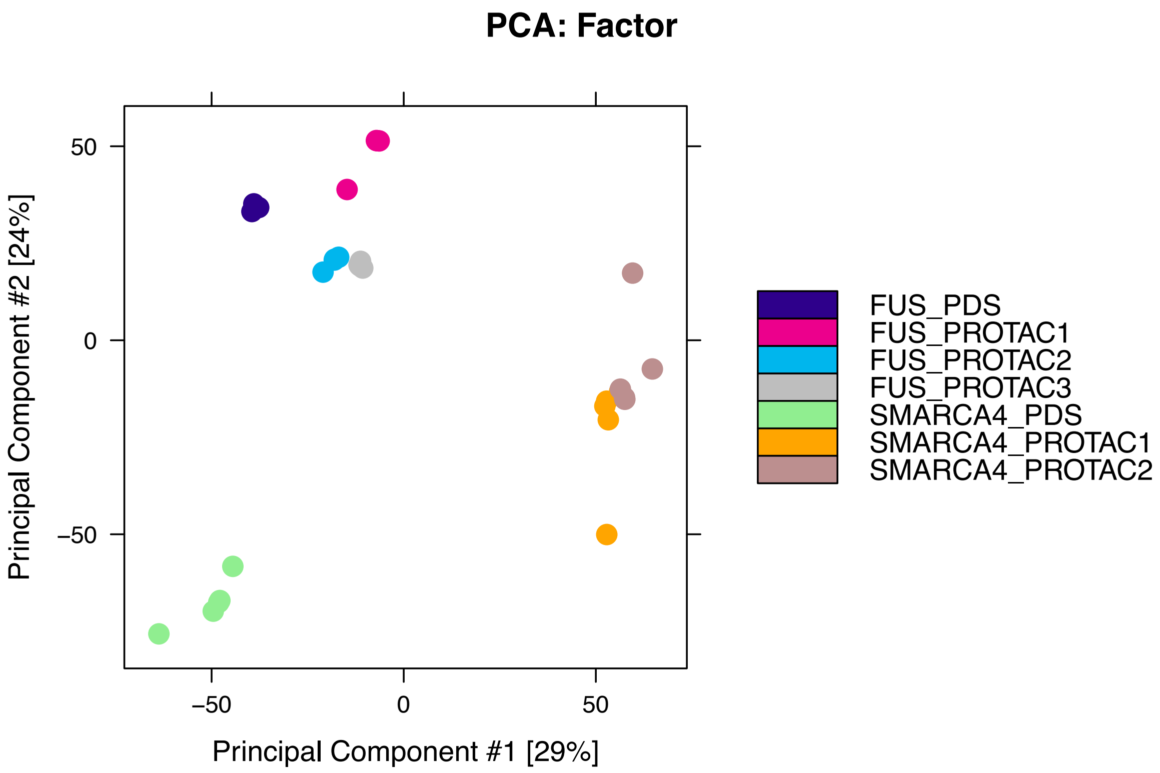

Principal component analysis (PCA) of genome-wide CUT&Tag signal for SMARCA4 and FUS across U2OS cells treated with PDS or G4L-PROTACs. Each point represents one replicate. Samples are color-coded by protein target and treatment group as indicated in the legend. Principal Component 1 (PC1) explains 29% of the variance, while PC2 explains 24%. Samples cluster distinctly by factor (FUS vs. SMARCA4) and show further separation by treatment condition, with clear divergence between control (PDS) and respective PROTAC treatments. This PCA confirms the reproducibility of CUT&Tag data and the consistent, treatment-specific effects of G4L-PROTACs on chromatin binding profiles.

Supplementary Figure 4. G4-specific depletion of SMARCA4 and FUS binding following G4L-PROTAC treatment.

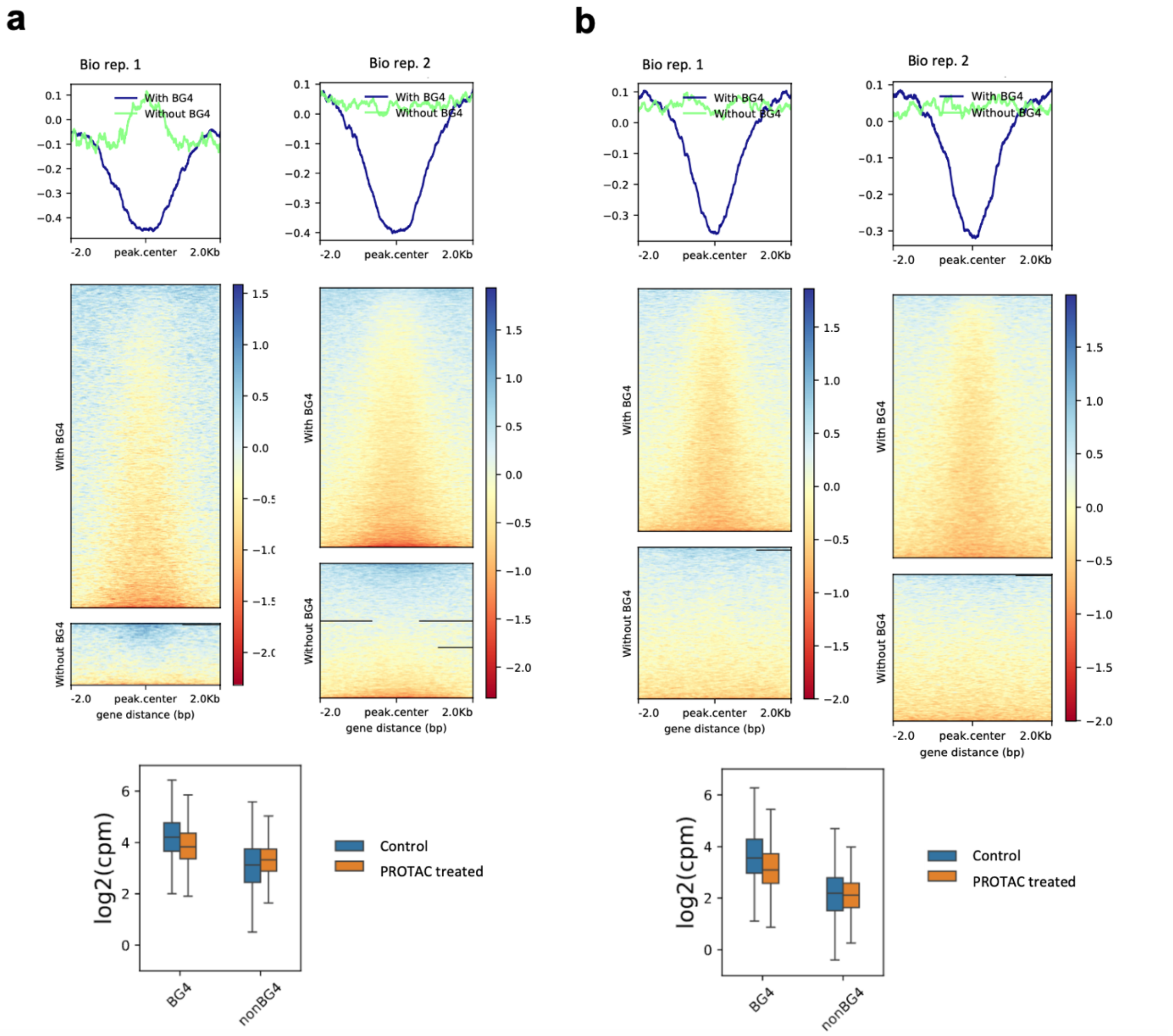

a, CUT&Tag analysis of SMARCA4 chromatin occupancy in U2OS cells treated with SMARCA4-targeting G4L-PROTAC1 compared to control. **Top:** Average signal profiles (two biological replicates) centered on peak regions overlapping BG4-defined G4 sites (blue) versus non-G4 sites (green). **Middle:** Heatmaps showing SMARCA4 signal intensities within ±2 kb of peak centers, stratified by G4 overlap. Reduced signal is observed specifically at G4 sites in the PROTAC-treated condition. **Bottom:** Box plots showing $\log_2$ counts per million (CPM) of SMARCA4 signal at G4 versus non-G4 sites in control and PROTAC-treated cells. G4-specific loss is evident with minimal change at non-G4 sites.

b, Same as (a) for FUS CUT&Tag, following treatment with FUS-targeting G4L-PROTAC3. G4 regions show strong depletion in FUS signal upon PROTAC treatment, with limited change at non-G4 peaks, across both replicates.

Supplementary Figure 5. Representative genome browser tracks of SMARCA4 CUT&Tag signal at G4 and non-G4 chromatin loci.

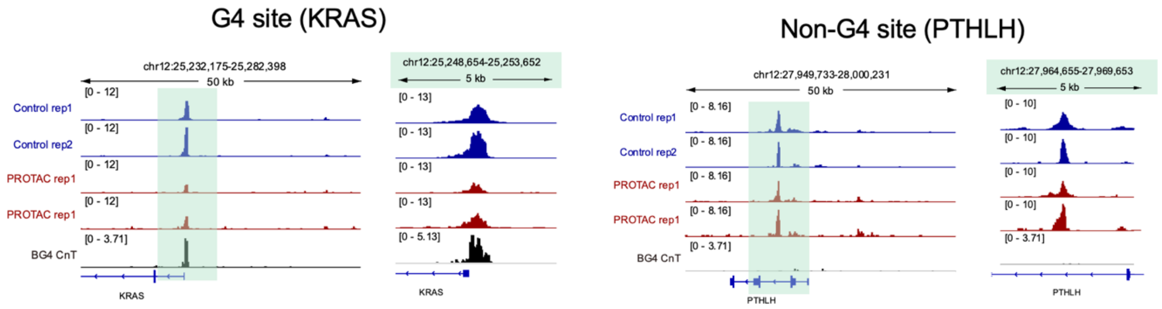

Genome browser views of SMARCA4 CUT&Tag signal at a G4-positive locus (KRAS; left) and a non-G4 locus (PTHLH; right) in U2OS cells treated with control (PDS; blue) or SMARCA4-targeting G4L-PROTAC (brown). Tracks from two biological replicates are shown for each condition. BG4 CUT&Tag (black) confirms G4 structure at the KRAS locus but not at PTHLH. SMARCA4 signal is markedly reduced at the G4 locus upon PROTAC treatment, while the non-G4 site shows relatively preserved binding, consistent with selective degradation of G4-associated SMARCA4. Shaded areas indicate regions of peak signal.

Supplementary Figure 6. G4 enrichment among SMARCA4 differential CUT&Tag peaks following PROTAC treatment.

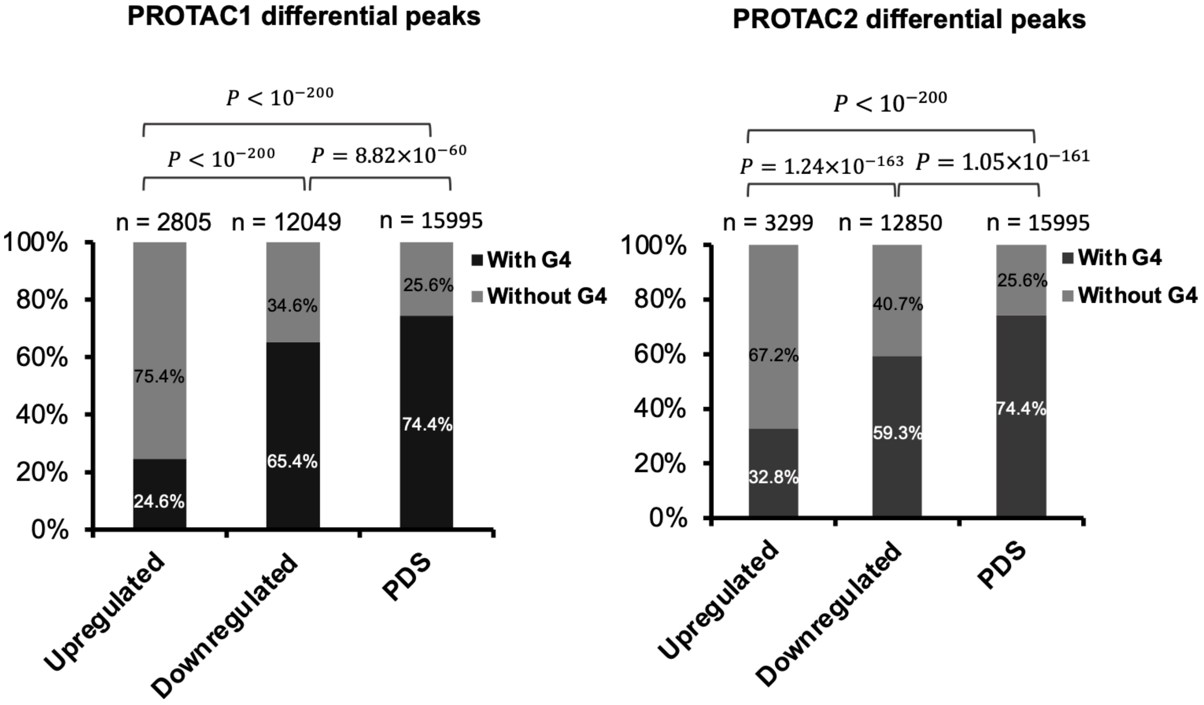

Stacked bar plots showing the proportion of SMARCA4 peaks that overlap with G4-forming regions (black) versus non-G4 regions (gray) in U2OS cells treated with PROTAC1 (left) or PROTAC2 (right), compared with PDS-treated controls. Bars represent peaks that are significantly upregulated, downregulated, or total peaks in the PDS reference group. Chi-square tests with Yates' correction reveal statistically significant enrichment of G4-overlapping peaks among downregulated SMARCA4 binding sites following treatment with either PROTAC, and depletion of G4-associated peaks among upregulated sites (all comparisons, $P < 10^{-60}$).

Supplementary Figure 7. Proportion of G4-overlapping peaks among SMARCA4 differential binding sites following PROTAC treatment.

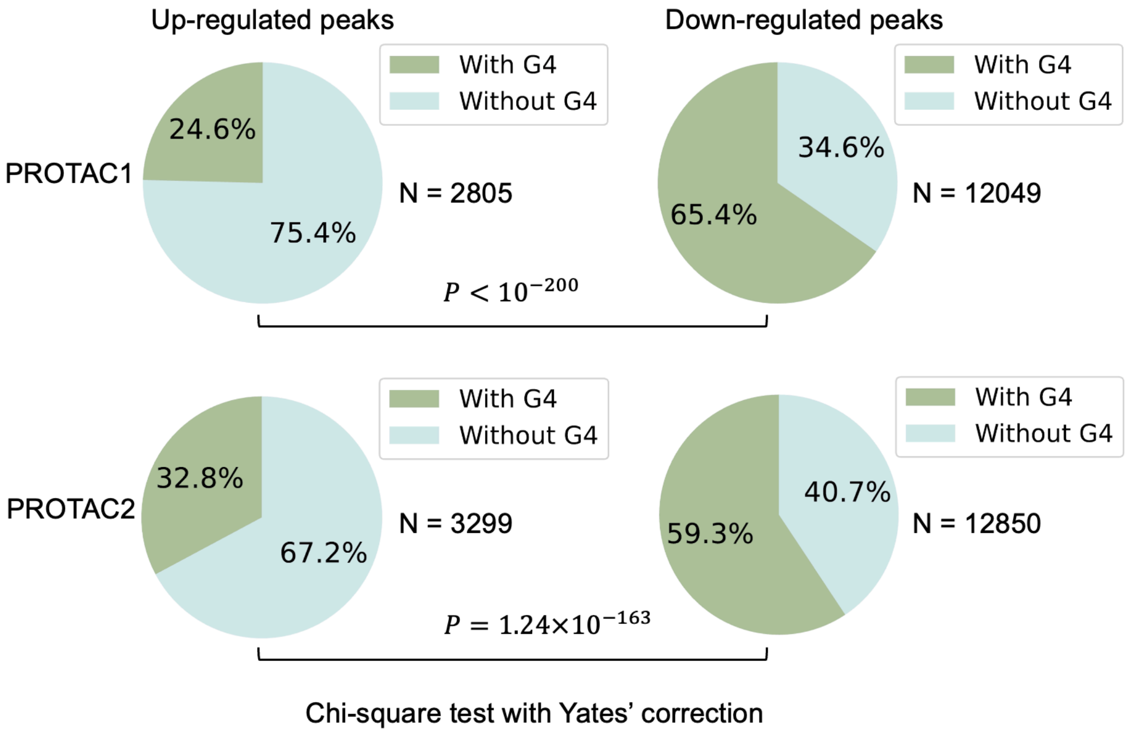

Pie charts show the fraction of SMARCA4 CUT&Tag peaks that overlap with G4-forming regions (green) or occur outside G4 loci (light blue) among upregulated and downregulated peaks in U2OS cells treated with PROTAC1 (top row) or PROTAC2 (bottom row). Peak counts and G4 proportions are indicated for each group. Chi-square tests with Yates' correction show that downregulated peaks are significantly enriched for G4-overlapping loci, while upregulated peaks are depleted for G4 association ($P < 10^{-163}$ for PROTAC2, $P < 10^{-200}$ for PROTAC1).

Supplementary Figure 8. G4 enrichment among FUS CUT&Tag peaks altered by G4L-PROTAC treatment.

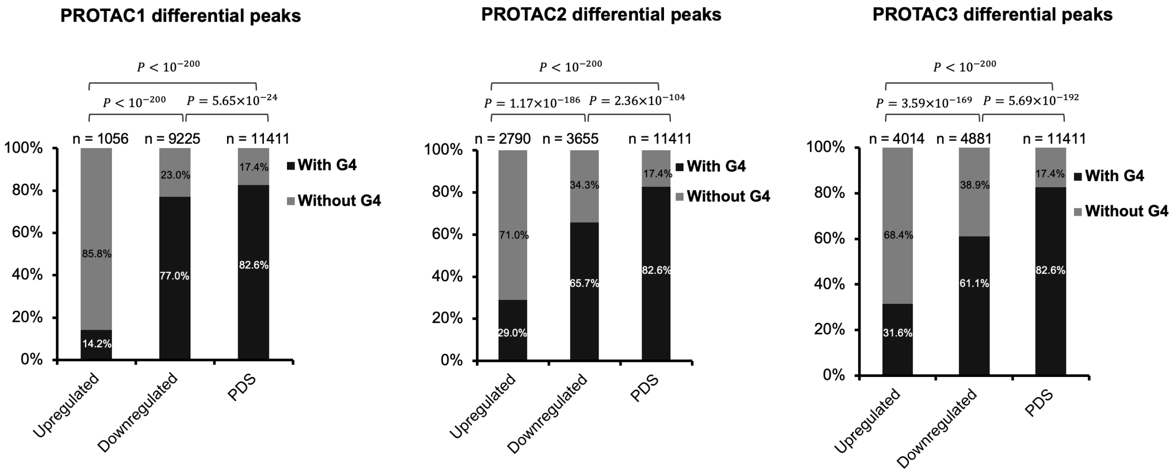

Stacked bar plots showing the proportion of FUS CUT&Tag peaks overlapping G4 regions (black) or non-G4 regions (gray) among peaks significantly upregulated, downregulated, or unchanged (PDS reference) following treatment with PROTAC1 (left), PROTAC2 (middle), or PROTAC3 (right). Chi-square tests with Yates' correction show highly significant differences in G4 enrichment, with G4-containing peaks disproportionately represented among downregulated regions and underrepresented among upregulated sites ($P < 10^{-104}$ to $P < 10^{-200}$ across comparisons).

Supplementary Figure 9. Proportion of G4-overlapping peaks among FUS differential binding sites following G4L-PROTAC treatment.

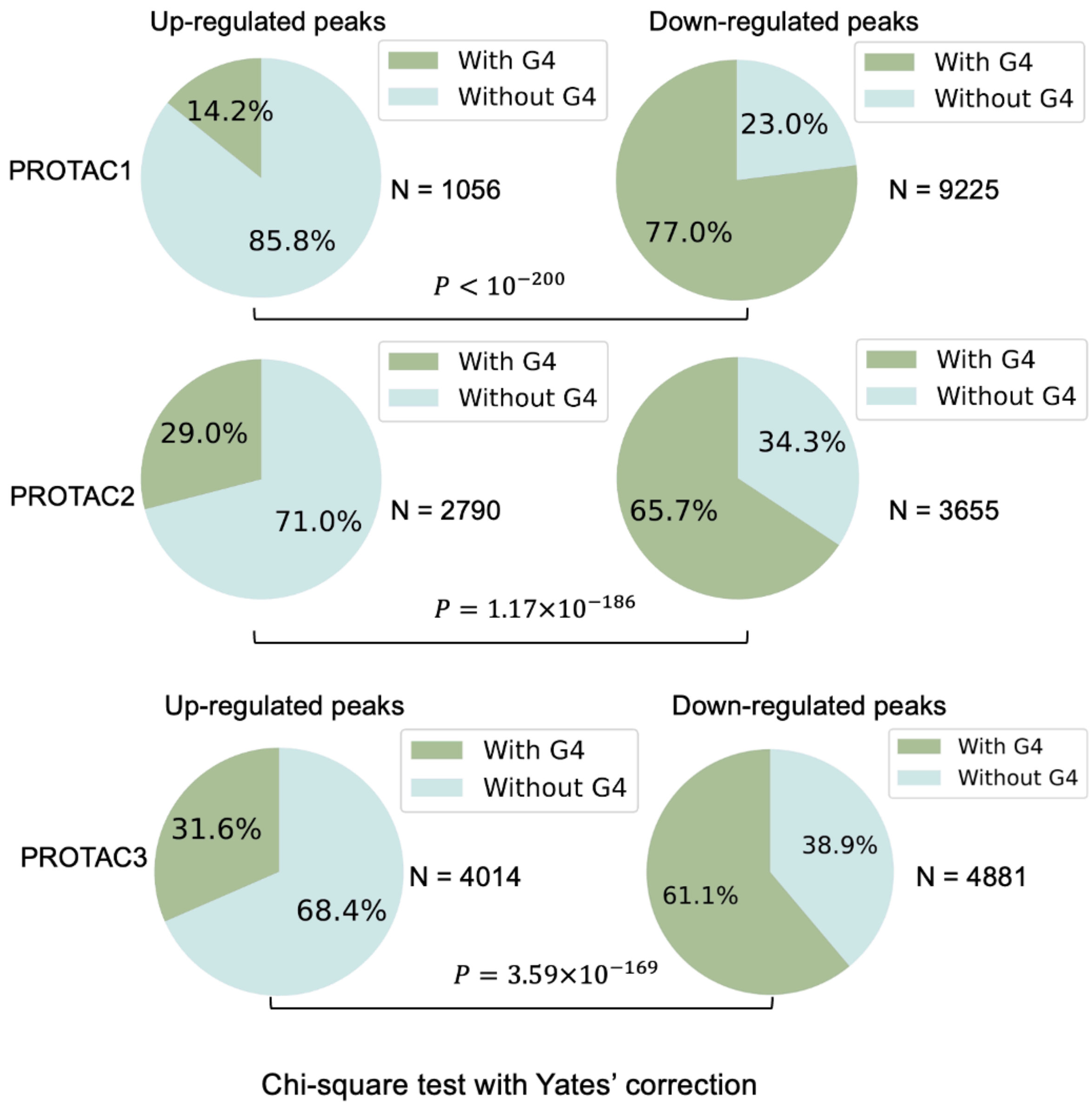

Pie charts showing the percentage of FUS CUT&Tag peaks that overlap G4-forming regions (green) or do not (light blue), stratified by significantly upregulated (left) or downregulated (right) peaks in U2OS cells treated with PROTAC1 (top row), PROTAC2 (middle row), or PROTAC3 (bottom row).

Chi-square tests with Yates' correction reveal strong statistical enrichment of G4-containing peaks among downregulated regions and significant depletion among upregulated peaks for each PROTAC ($P < 10^{-169}$ to $P < 10^{-200}$).

Supplementary Figure 10. Reproducibility of CUT&Tag replicates for SMARCA4 and FUS across G4L-PROTAC treatments.

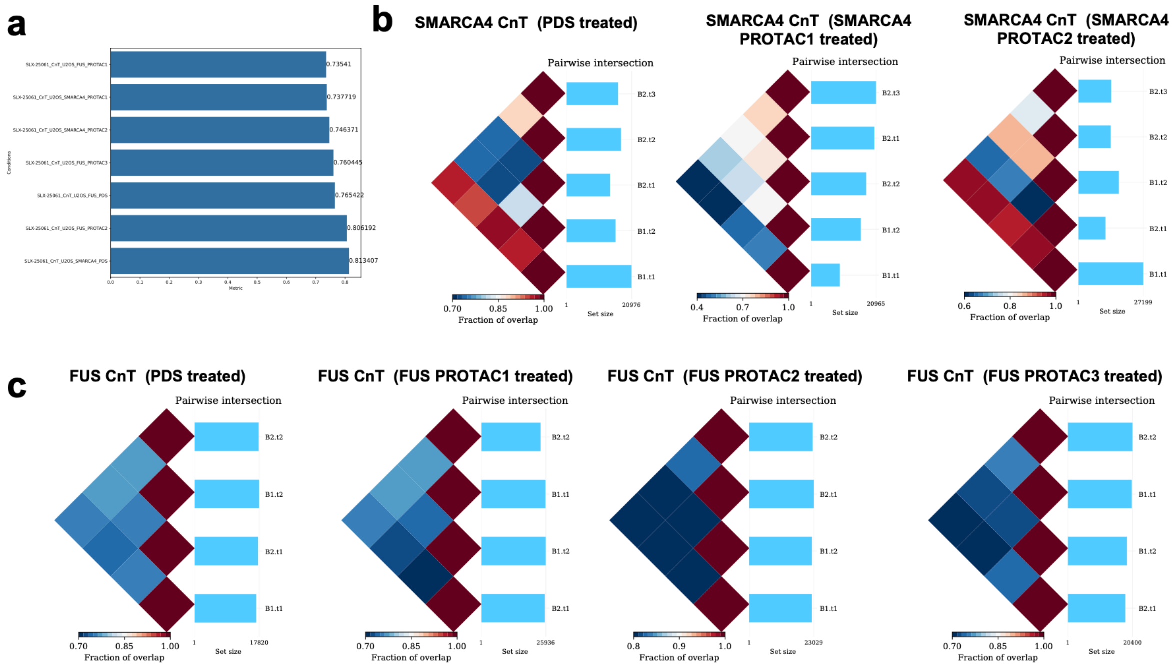

a, Bar plot summarizing pairwise similarity metrics (Jaccard index) across CUT&Tag replicate datasets for SMARCA4 and FUS under control (PDS-treated) and various G4L-PROTAC treatments. High correlation values across conditions indicate consistent profiling.

b, Pairwise intersection matrices showing the fraction of overlapping peaks among technical and biological replicates for SMARCA4 CUT&Tag under PDS treatment (left), PROTAC1 (middle), and PROTAC2 (right). Set sizes (right-hand bar plots) indicate the total number of peaks in each replicate.

c, Corresponding pairwise intersection matrices for FUS CUT&Tag replicates under PDS (left), PROTAC1 (middle left), PROTAC2 (middle right), and PROTAC3 (right) treatments. Consistently high overlap between replicates confirms robust reproducibility and peak detection.

Supplementary Figure 11. Intersections of CUT&Tag peak sets for SMARCA4 and FUS under G4L-PROTAC treatments.

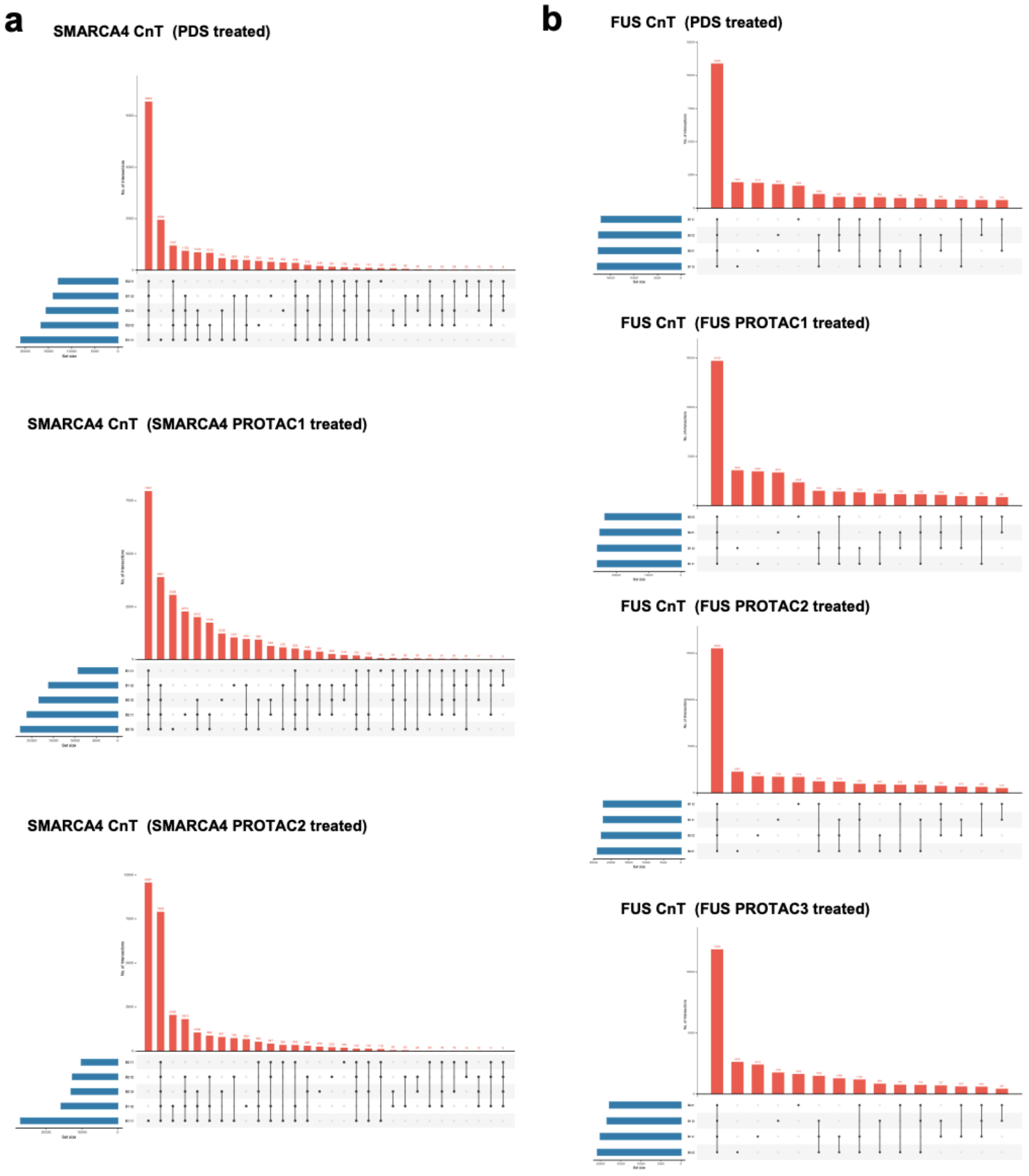

a, UpSet plots showing the distribution and intersection of SMARCA4 CUT&Tag peaks across replicate samples in cells treated with PDS (top), SMARCA4 PROTAC1 (middle), or SMARCA4 PROTAC2 (bottom). Bars (top) represent the number of peaks unique or shared across combinations of replicates (bottom matrix), while side bars indicate total peaks per sample.

b, UpSet plots for FUS CUT&Tag peak sets in cells treated with PDS (top), FUS PROTAC1 (middle top), FUS PROTAC2 (middle bottom), or FUS PROTAC3 (bottom). Shared and unique peak distributions are displayed as in (a), highlighting reproducible peak sets and treatment-specific differences in chromatin occupancy.

Supplementary Figure 12. Differential analysis of FUS CUT&Tag binding across G4L-PROTAC treatments.

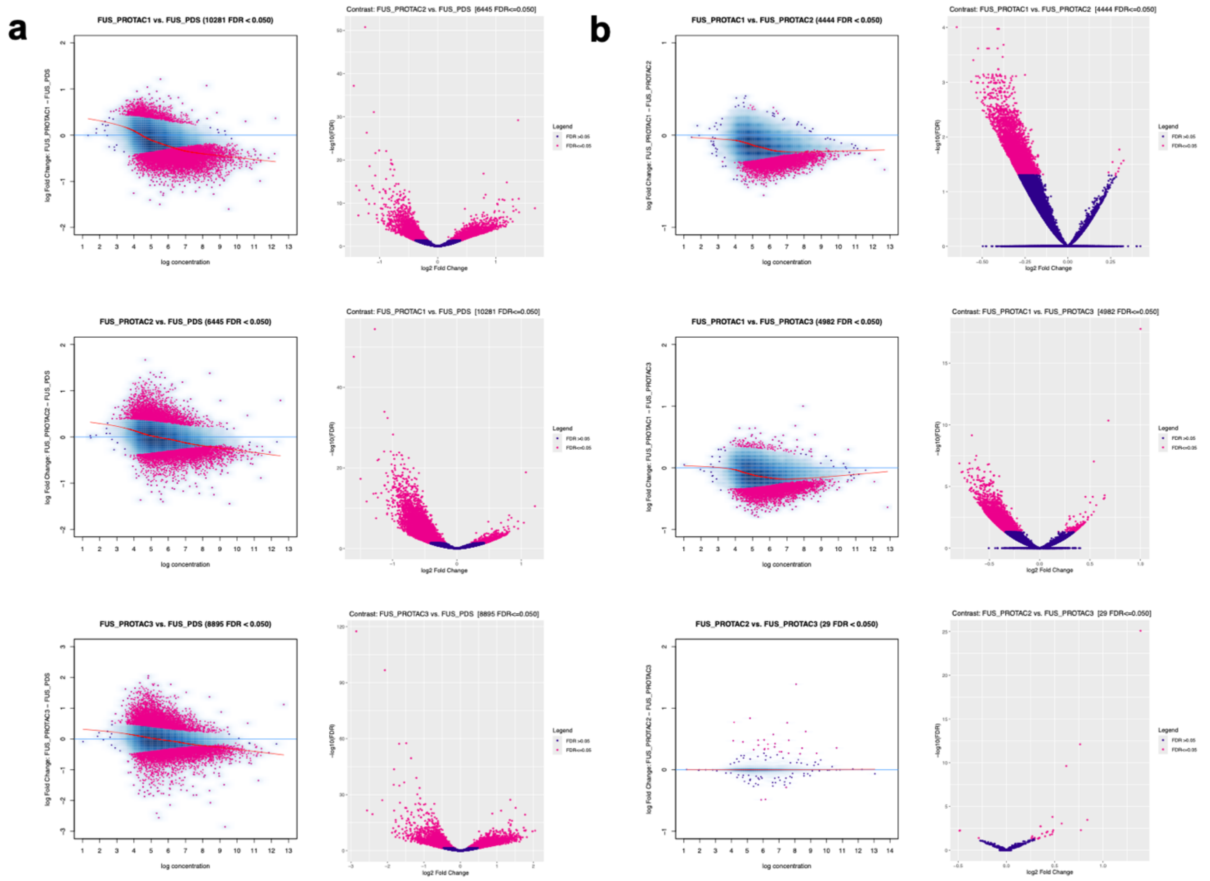

a, Scatter plots (left) and volcano plots (right) comparing FUS chromatin occupancy in U2OS cells treated with FUS-targeting G4L-PROTACs versus PDS. Differential enrichment is shown for FUS PROTAC1, PROTAC2, and PROTAC3 relative to PDS-treated controls. Each dot represents a CUT&Tag peak, with significantly downregulated peaks (FDR < 0.05) highlighted in pink.

b, Pairwise comparisons of FUS CUT&Tag signal across different PROTAC treatments (e.g., PROTAC1 vs. PROTAC2, PROTAC1 vs. PROTAC3, PROTAC2 vs. PROTAC3). Scatter plots (left) and corresponding volcano plots (right) indicate treatment-specific differences in peak signal intensity and statistical significance.

Supplementary Figure 13. Differential analysis of SMARCA4 CUT&Tag binding following G4L-PROTAC treatment.

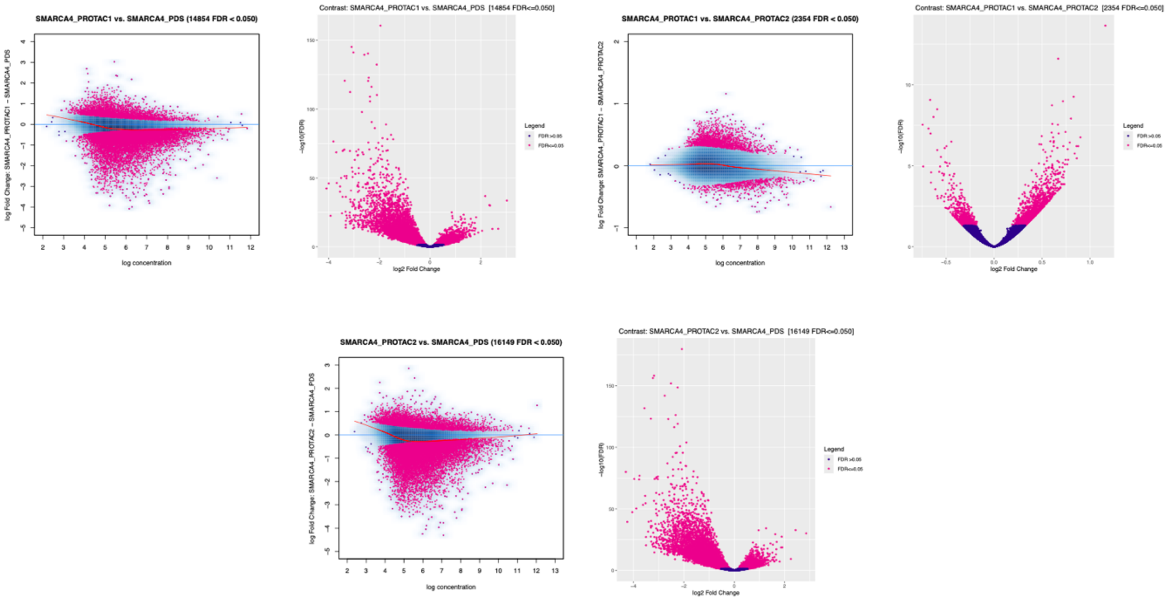

Scatter plots (left) and volcano plots (right) display differential chromatin occupancy of SMARCA4 in U2OS cells treated with G4L-PROTAC1 or G4L-PROTAC2, compared with PDS-treated controls or each other.

Top row: SMARCA4 PROTAC1 versus PDS (left two panels), and SMARCA4 PROTAC1 versus PROTAC2 (right two panels).

Bottom row: SMARCA4 PROTAC2 versus PDS. Each dot represents a SMARCA4 CUT&Tag peak; significantly differentially enriched sites (FDR < 0.05) are highlighted in pink.

Supplementary Figure 14. Functional and genomic annotation of regions with reduced SMARCA4 occupancy following PROTAC1 treatment.

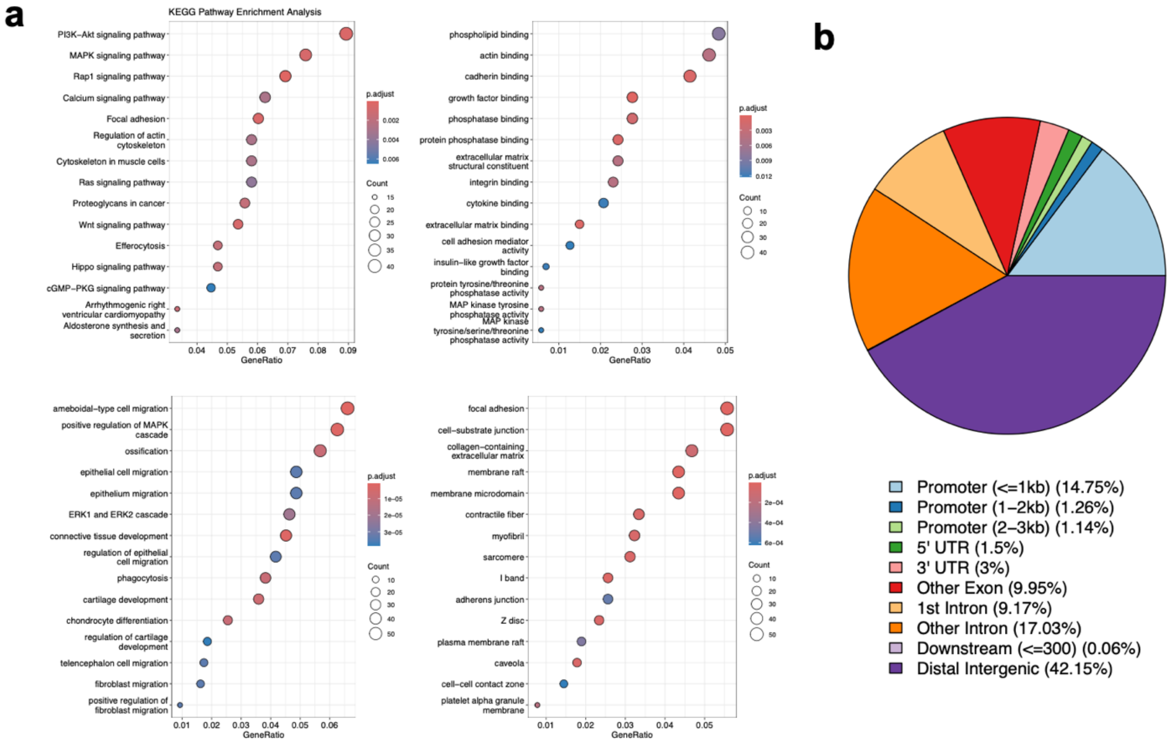

a, KEGG and GO enrichment analysis of genes associated with significantly downregulated SMARCA4 CUT&Tag peaks in U2OS cells treated with SMARCA4-targeting PROTAC1 compared to PDS control. Dot plots represent enriched terms from (top left) KEGG pathways, (top right) molecular function (MF), (bottom left) biological process (BP), and (bottom right) cellular component (CC) categories. Dot size indicates the number of associated genes, and color reflects adjusted p-value. Enriched pathways include PI3K-Akt signaling, MAPK signaling, and focal adhesion, suggesting functional consequences for signal transduction and cytoskeletal organization.

b, Genomic distribution of significantly downregulated SMARCA4 binding sites. Peaks are most frequently localized to distal intergenic regions (42.15%) and intronic regions (26.2%), with a smaller fraction at promoter-proximal regions (≤3 kb).

Supplementary Figure 15. Functional and genomic annotation of regions with increased SMARCA4 occupancy following PROTAC1 treatment.

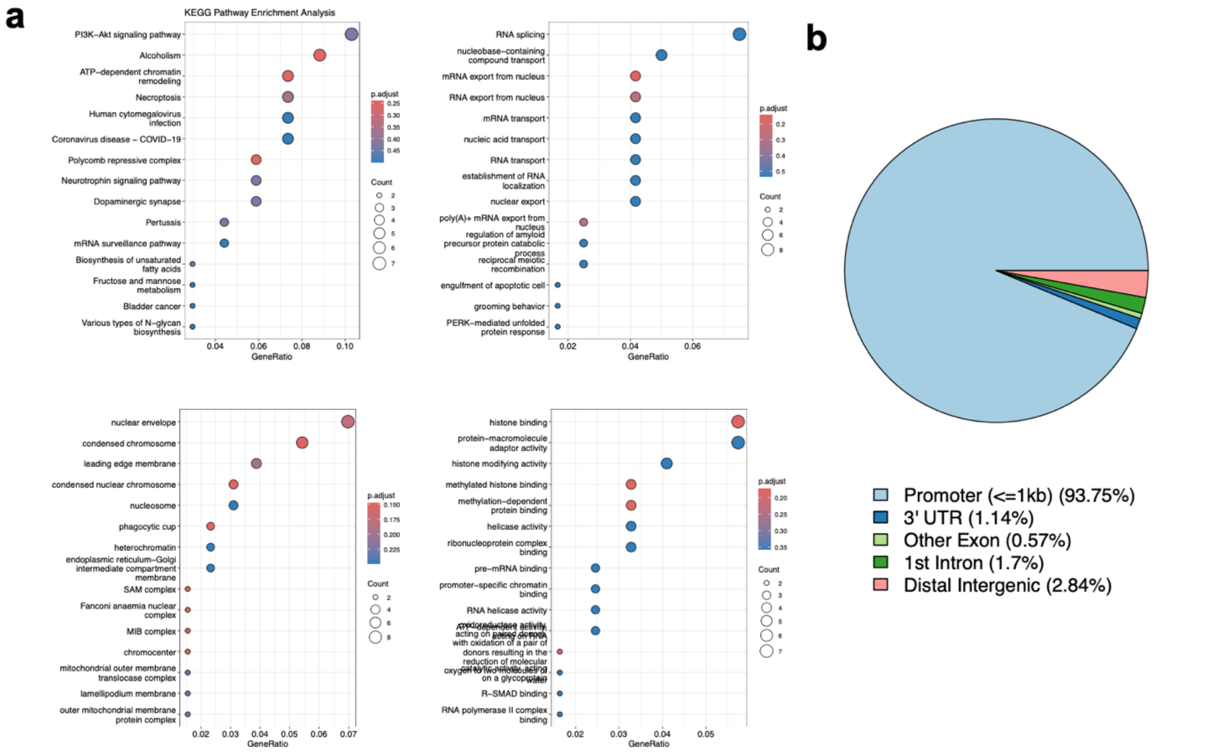

a, Enrichment analysis of genes associated with significantly upregulated SMARCA4 CUT&Tag peaks in U2OS cells treated with SMARCA4-targeting PROTAC1 versus PDS control. Dot plots display enriched categories from KEGG pathways (top left), biological processes (top right), cellular components (bottom left), and molecular functions (bottom right). GeneRatio represents the proportion of input genes mapped to a given term; dot size reflects gene count, and color indicates adjusted p-value. Enriched terms include mRNA processing, RNA export, chromatin binding, and histone modification-related activities.

b, Genomic distribution of SMARCA4 peaks significantly increased upon PROTAC1 treatment. The majority of upregulated peaks (93.75%) are located within promoter-proximal regions (≤1 kb), with a small fraction mapping to distal intergenic and genic regulatory regions.

Supplementary Figure 16. Functional and genomic annotation of regions with reduced SMARCA4 occupancy following PROTAC2 treatment.

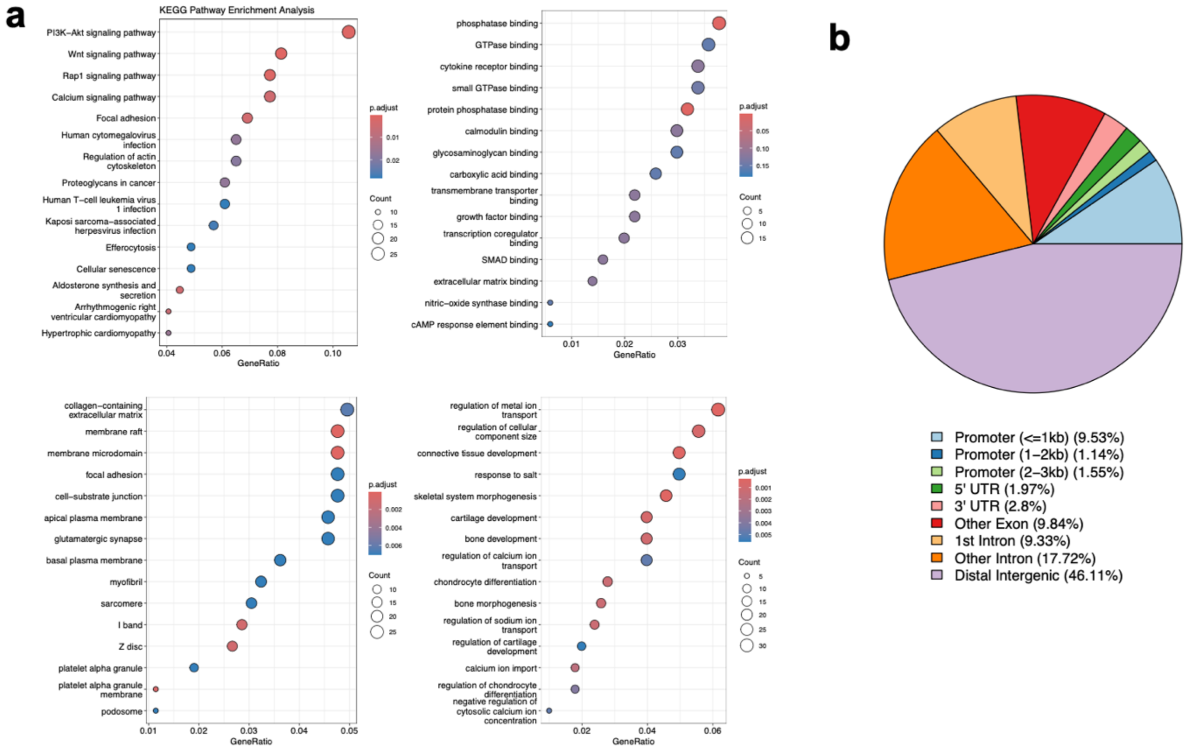

a, KEGG and GO enrichment analysis of genes associated with significantly downregulated SMARCA4 CUT&Tag peaks in U2OS cells treated with SMARCA4-targeting PROTAC2 compared to PDS control. Dot plots show enriched terms from KEGG pathways (top left), molecular function (top right), cellular component (bottom left), and biological process (bottom right). Dot size reflects the number of associated genes; color indicates adjusted p-value. Enriched pathways include PI3K-Akt signaling, Wnt signaling, and focal adhesion, suggesting altered cell signaling and structural organization upon SMARCA4 depletion.

b, Genomic annotation of differentially downregulated SMARCA4 binding peaks, indicating substantial enrichment at distal intergenic (46.11%) and intronic regions (combined 27.05%), with lower representation at promoter-proximal loci (12.22%).

Supplementary Figure 17. Functional and genomic annotation of regions with increased SMARCA4 occupancy following PROTAC2 treatment.

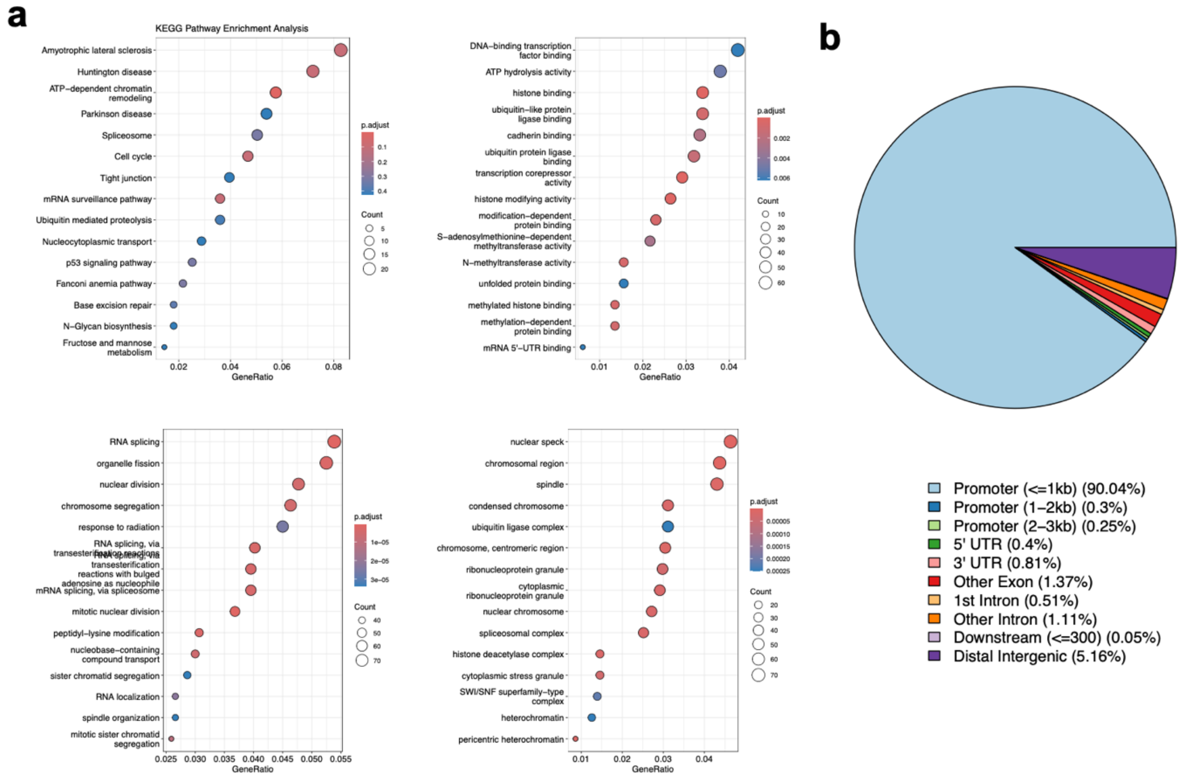

a, KEGG and GO enrichment analysis of genes associated with significantly upregulated SMARCA4 CUT&Tag peaks in U2OS cells treated with SMARCA4-targeting PROTAC2 versus PDS control. Dot plots display significantly enriched terms from KEGG pathways (top left), molecular function (top right), biological process (bottom left), and cellular component (bottom right). GeneRatio represents the proportion of genes associated with each term; dot size indicates gene count, and color reflects adjusted p-value. Upregulated peaks are associated with pathways involved in chromatin remodeling, RNA splicing, mitotic progression, and ubiquitin-mediated proteolysis.

b, Genomic distribution of SMARCA4 peaks significantly increased by PROTAC2 treatment. The majority are enriched at promoter regions ≤1 kb (90.04%), with relatively few sites in distal intergenic and other genic regions.

Supplementary Figure 18. Volcano plots showing proteome-wide depletion of G4-binding proteins across G4L-PROTAC variants.

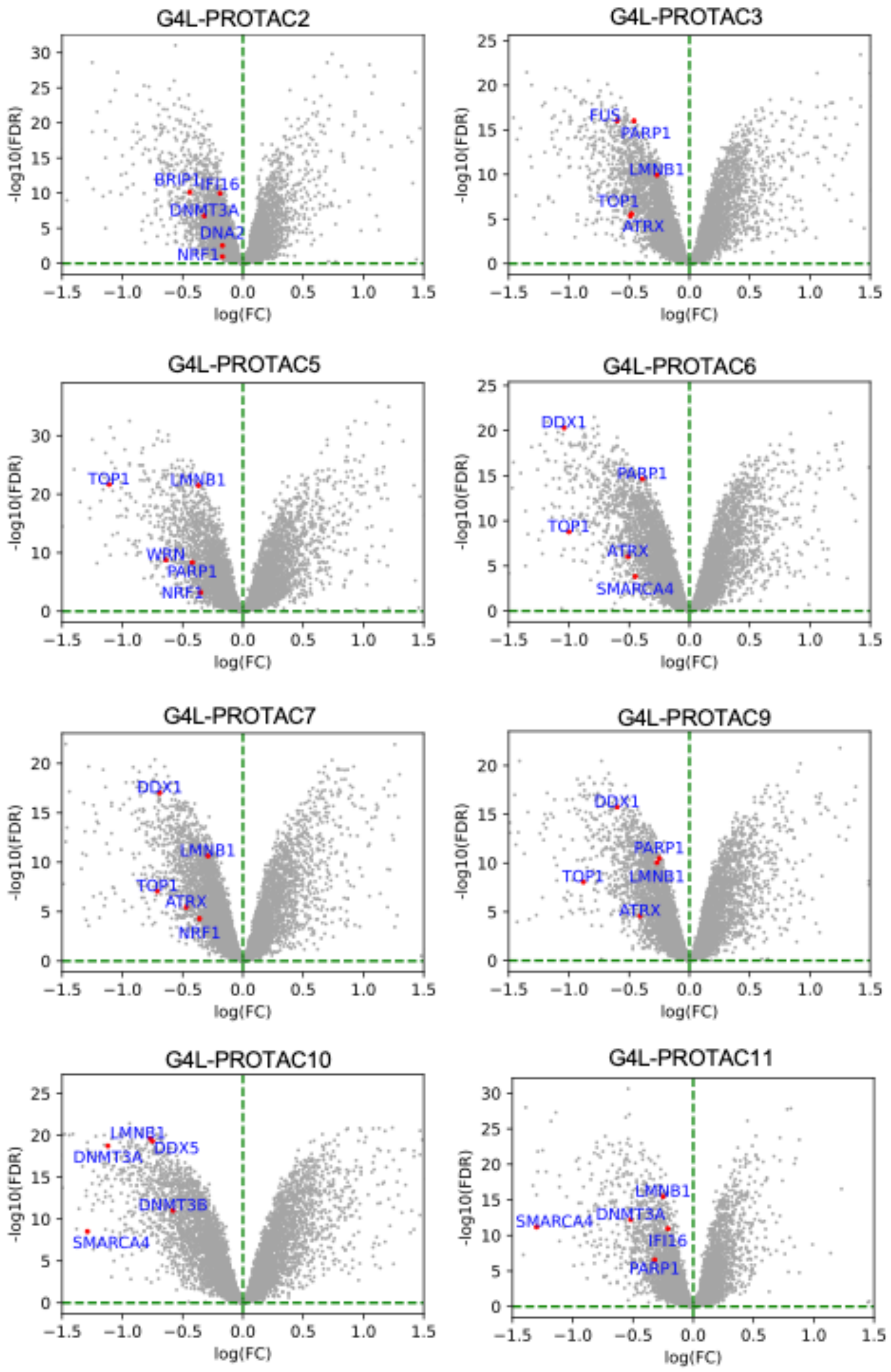

Volcano plots display $\log_2$ fold change (x-axis) versus $-\log_{10}$(FDR) (y-axis) for protein abundance in U2OS cells treated with individual G4L-PROTAC compounds (G4L-PROTAC2 through G4L-PROTAC11) compared to a negative control PROTAC. Each dot represents a

protein identified by quantitative mass spectrometry. Significantly downregulated proteins (left of vertical green line) include known G-quadruplex binding proteins (G4BPs), highlighted and labeled in blue. Prominent G4BPs such as TOP1, PARP1, LMNB1, ATRX, SMARCA4, and DDX1 are consistently depleted across multiple compounds, indicating conserved G4-targeting activity. G4L-PROTAC5 and G4L-PROTAC6 display particularly broad depletion signatures, suggesting enhanced degradation efficiency. The green dashed lines indicate FDR and fold-change thresholds used to define significant changes.

Supplementary Figure 19. Volcano plots showing depletion of RNA G4-binding proteins across G4L-PROTAC variants.

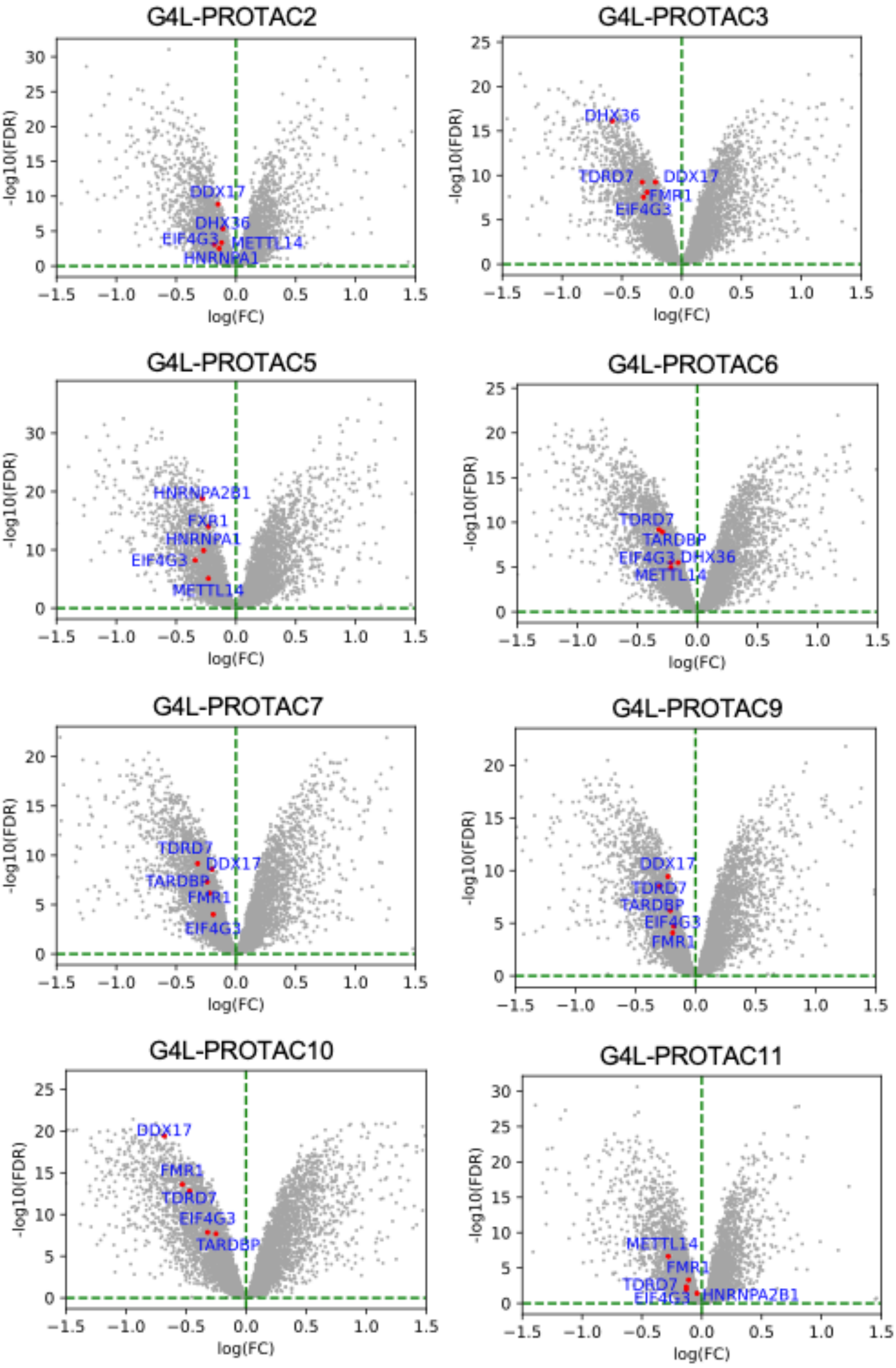

Volcano plots display proteomic fold changes for RNA G4-binding proteins (G4RBPs) in U2OS cells treated with G4L-PROTAC2 through G4L-PROTAC11, relative to a negative control PROTAC. The x-axis represents $\log_2$ fold change (logFC), and the y-axis shows statistical significance as $-\log_{10}$(FDR). Each dot corresponds to an individual protein identified

by mass spectrometry; significantly downregulated RNA G4RBPs are labeled in blue. Key G4RBPs such as **DDX17**, **DHX36**, **EIF4G3**, **FMR1**, **TDRD7**, **TARDBP**, **HNRNPA2B1**, and **METTL14** are consistently depleted across multiple G4L-PROTACs, indicating a shared degradation signature for RNA G4-targeting activity. Green dashed lines represent the FDR and fold-change thresholds used to identify statistically significant changes.

Supplementary Figure 20. Heatmap of differentially expressed proteins across G4L-PROTAC treatments.

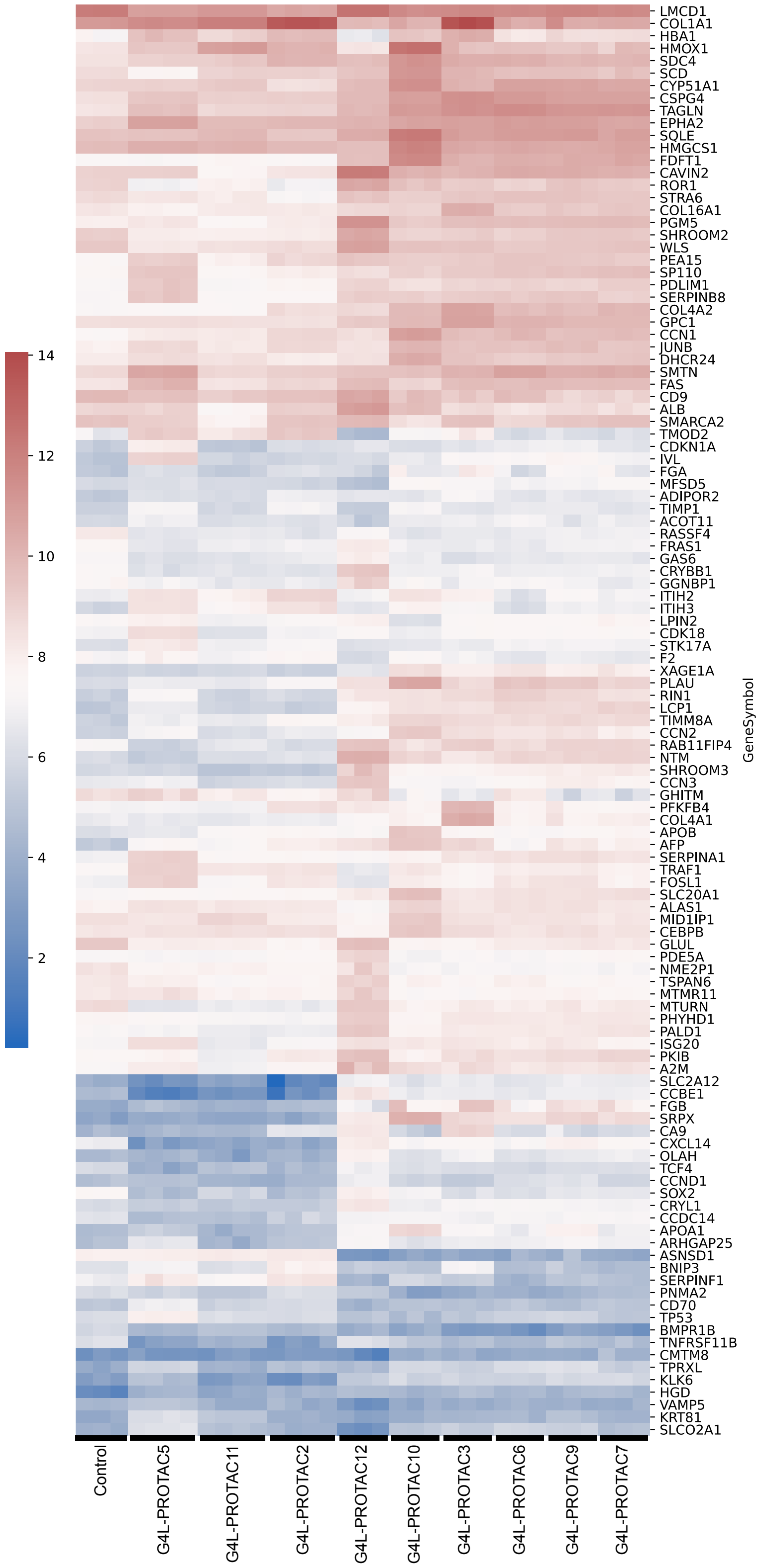

Unsupervised hierarchical clustering heatmap of $\log_2$ fold change values for proteins significantly altered by treatment with the G4L-PROTAC series in U2OS cells, relative to a negative control PROTAC. Each column represents a G4L-PROTAC-treated sample, and each

row corresponds to a protein. Color intensity reflects relative protein abundance, with red indicating upregulation and blue indicating downregulation. The heatmap reveals distinct clustering patterns among G4L-PROTACs and highlights both shared and compound-specific proteome remodeling effects.

Notably, subsets of proteins are consistently downregulated across multiple compounds, including known G4-binding proteins, while other proteins show treatment-specific modulation, reflecting differences in degradation specificity or downstream pathway engagement. These results illustrate the broad yet selective proteomic impact of G4L-PROTACs and support their ability to differentially remodel cellular protein networks based on compound structure.

Supplementary Figure 21. Gene set enrichment analysis (GSEA) of proteomic changes in G4L-PROTAC2-treated cells.

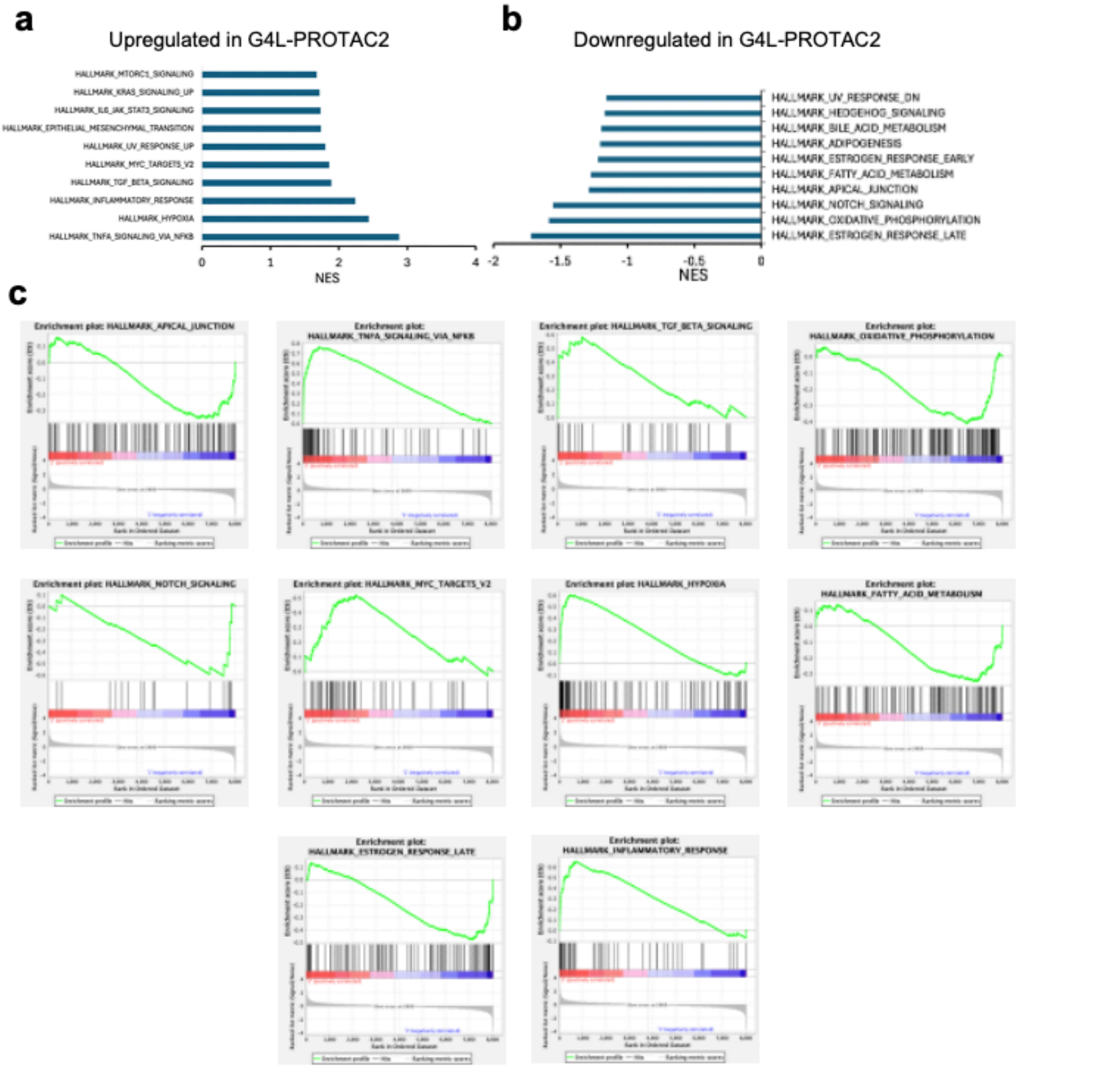

a, b, Bar plots showing normalized enrichment scores (NES) for hallmark gene sets significantly upregulated (a) or downregulated (b) in U2OS cells treated with G4L-PROTAC2 compared to a negative control PROTAC. Top enriched pathways in the upregulated group include mTORC1 signaling, TNFα via NF-κB, MYC targets, and epithelial–mesenchymal transition, while downregulated pathways include oxidative phosphorylation, estrogen response, fatty acid metabolism, and Notch signaling.

c, GSEA enrichment plots for a subset of hallmark gene sets from (a) and (b), illustrating enrichment distribution and leading-edge subsets. Each plot shows the running enrichment score (green curve), the position of gene set members in the ranked list (black ticks), and the enrichment profile.

Supplementary Figure 22. Gene set enrichment analysis of proteomic changes in G4L-PROTAC11-treated U2OS cells.

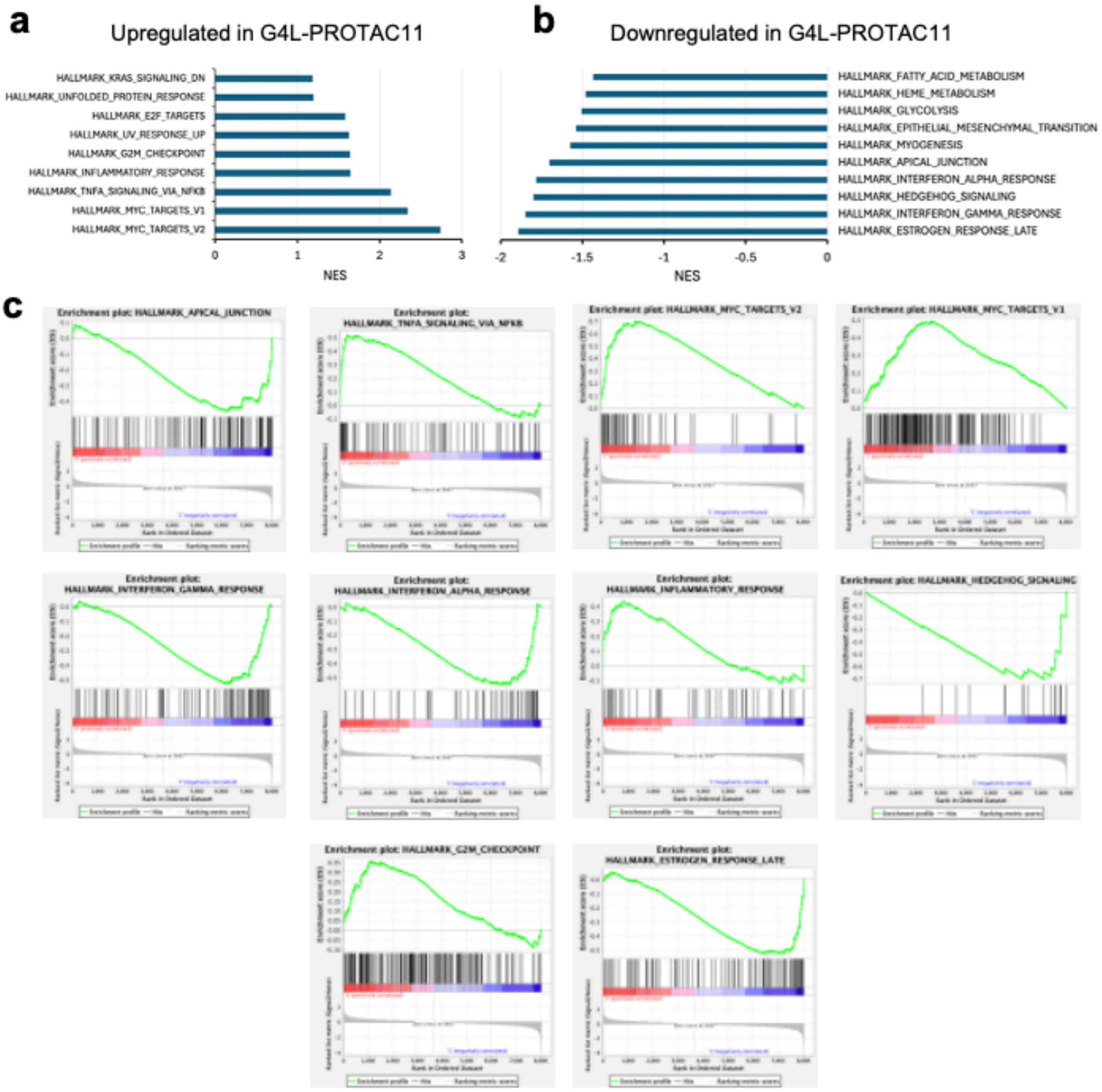

a, Normalized enrichment scores (NES) from hallmark gene set enrichment analysis comparing G4L-PROTAC11 treatment to a negative control PROTAC. Upregulated gene sets include MYC targets, TNFα via NF-κB, inflammatory response, G2/M checkpoint, and UV response, indicating activation of proliferative and stress-related transcriptional programs.

b, Downregulated gene sets include fatty acid metabolism, heme metabolism, glycolysis, myogenesis, and interferon signaling, pointing to suppression of metabolic and differentiation-related pathways.

c, Representative enrichment plots from selected hallmark gene sets in (a) and (b), showing the distribution of gene ranks and enrichment profiles. Each plot displays the running enrichment score (green line), rank positions (black ticks), and leading-edge subsets.

Supplementary Figure 23. Gene set enrichment analysis of proteomic changes in G4L-PROTAC5-treated U2OS cells.

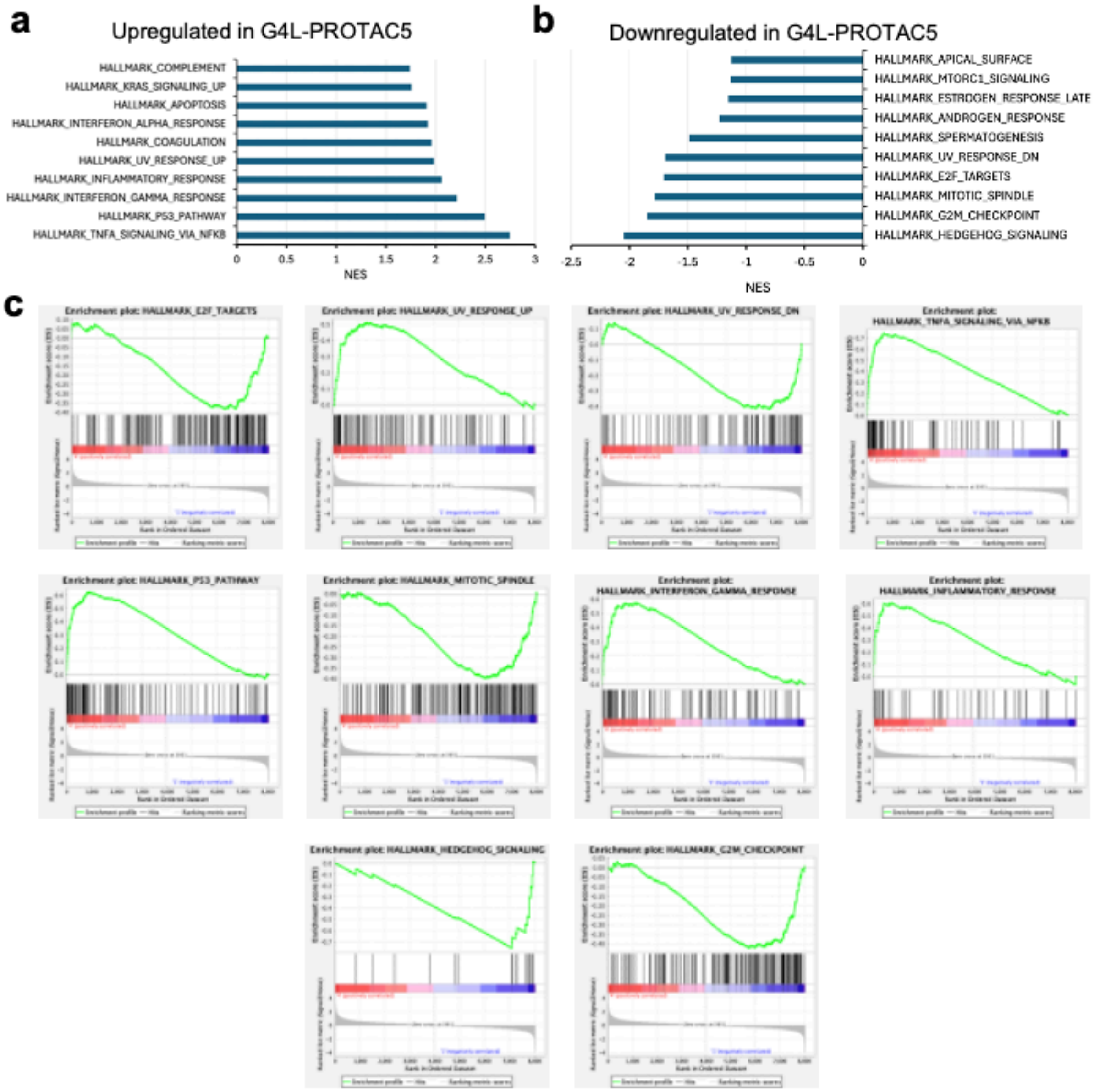

a, Normalized enrichment scores (NES) from hallmark gene set enrichment analysis comparing G4L-PROTAC5 treatment versus negative control PROTAC. Upregulated pathways include TNFα signaling via NF-κB, p53 pathway, interferon responses (α and γ), complement, KRAS signaling, and apoptosis, indicating enhanced inflammatory, stress, and immune-related responses.

b, Downregulated pathways include mTORC1 signaling, mitotic spindle, androgen response, and hedgehog signaling, reflecting suppression of cell cycle progression, metabolism, and developmental signaling.

c, Representative enrichment plots from selected gene sets showing the ranked position of signature genes and enrichment profile scores (green lines). Enrichment of inflammatory and apoptotic signatures is contrasted with depletion of proliferative and hormone-responsive gene programs.

Supplementary Figure 24. AlphaFold-predicted structural models and confidence scores for candidate G4-binding protein interactions.

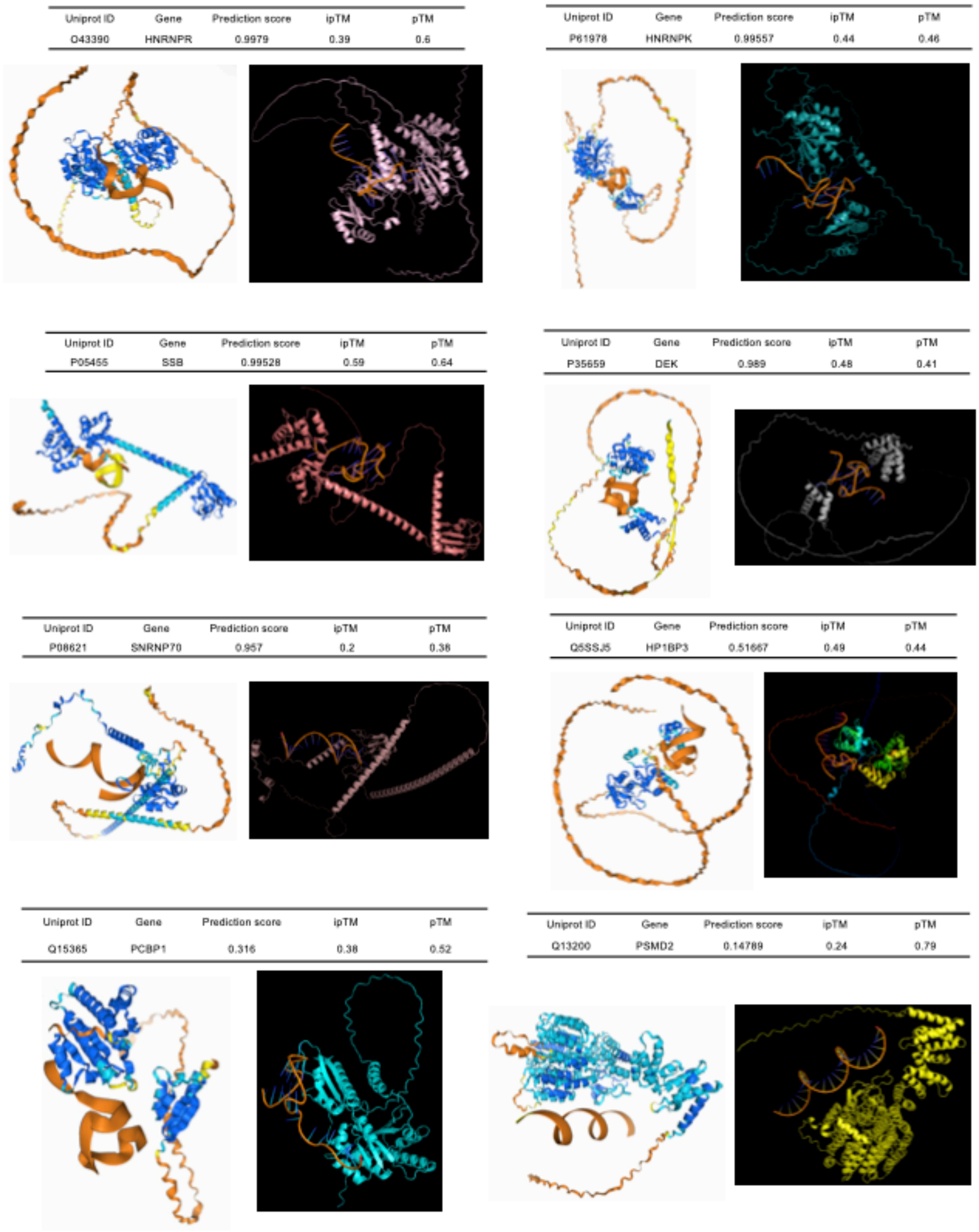

Predicted complex structures of selected G4-binding protein (G4BP) candidates generated using AlphaFold-Multimer. For each protein, two views are shown: the full predicted complex (left) and a ribbon-style or surface representation (right) highlighting protein-protein interfaces. UniProt ID, gene symbol, and model prediction scores are indicated above each pair of images. Confidence metrics include the ipTM (interface predicted TM-score) and pTM (predicted TM-score), reflecting the predicted accuracy of subunit interfaces and the overall complex fold,

respectively. High-confidence predictions (ipTM > 0.8) are observed for select complexes (e.g., HNRNPIR, SSB), while intermediate (0.6–0.8) and low-confidence (≤0.6) models are also represented. Notably, several G4BP candidates exhibit structural confidence in the interface regions, suggesting potential for direct or stable interaction in a G4-associated context.

Supplementary Figure 25. AlphaFold-Multimer structural predictions for candidate G4-interacting protein complexes with low to intermediate confidence scores.

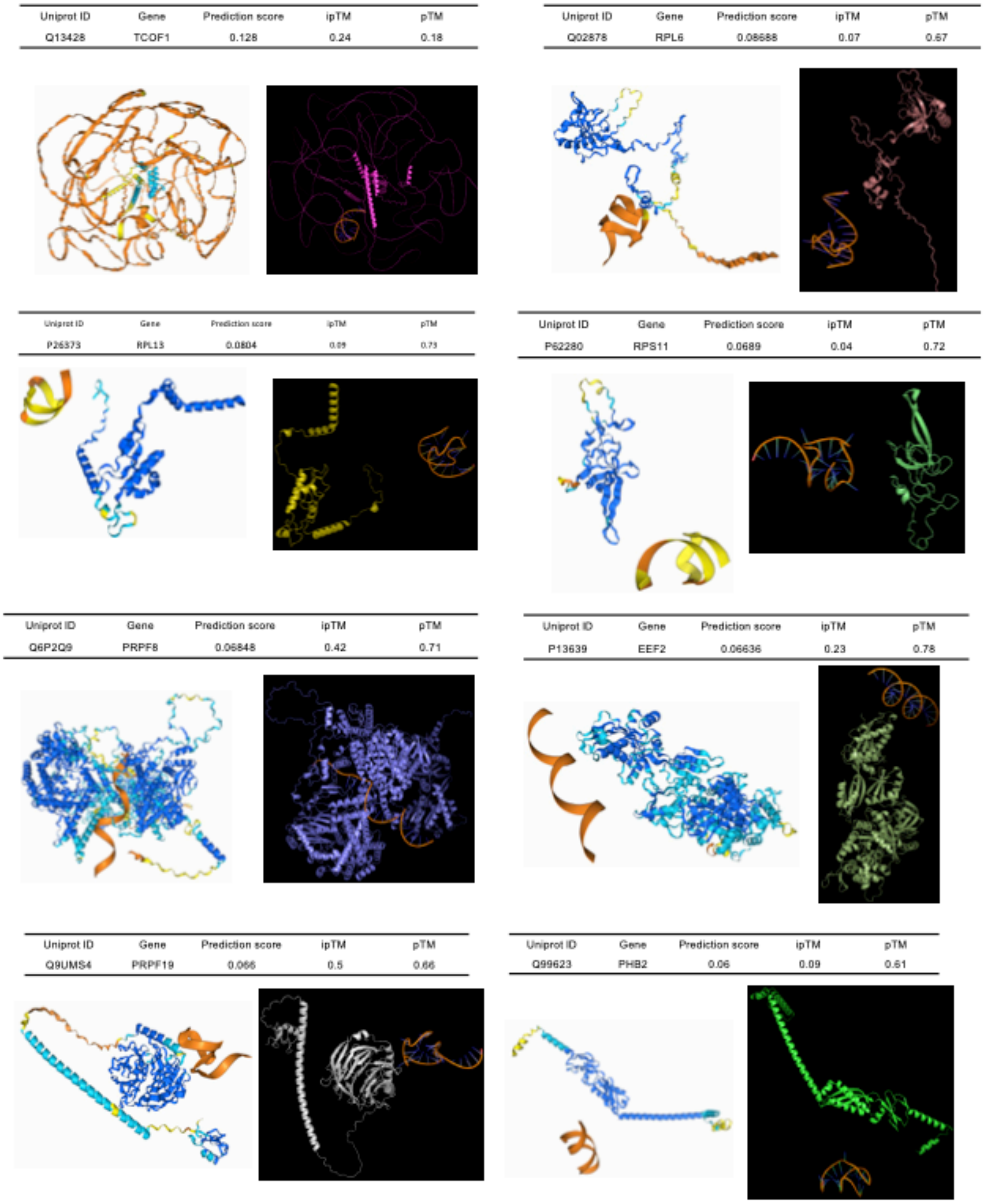

Predicted complex models for selected proteins with potential G4 interactions, generated using AlphaFold-Multimer. Each protein pair is represented by a full structure model (left) and a secondary or surface representation (right) to illustrate conformational detail and subunit interfaces. UniProt IDs, gene names, and prediction confidence scores are provided above each panel. ipTM scores (interface predicted TM) and pTM scores (predicted TM) reflect AlphaFold's confidence in interface accuracy and global fold, respectively. Most models

shown here fall within the intermediate (0.6–0.8) or low (<0.6) confidence ranges, indicating varying levels of prediction reliability. A few structures (e.g., EEF2, PRPF19, RPS11) approach interface prediction scores consistent with potentially meaningful contacts.

Supplementary Figure 26. Network diagram for G4L-PROTACs downregulated hubs.

G4L-PROTAC2

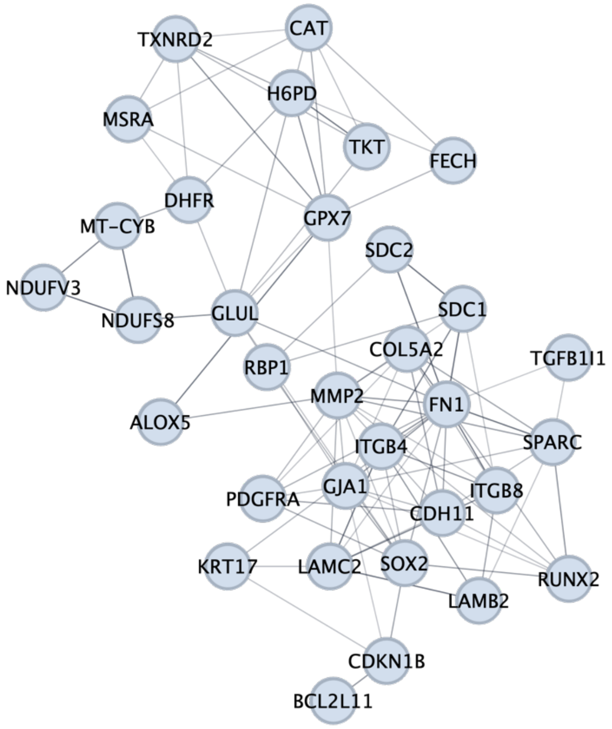

G4L-PROTAC11

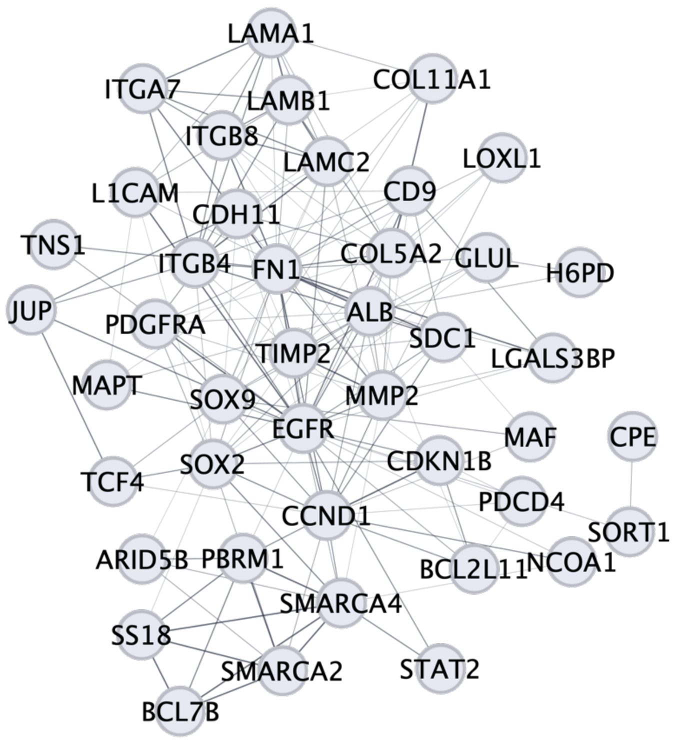

G4L-PROTAC5

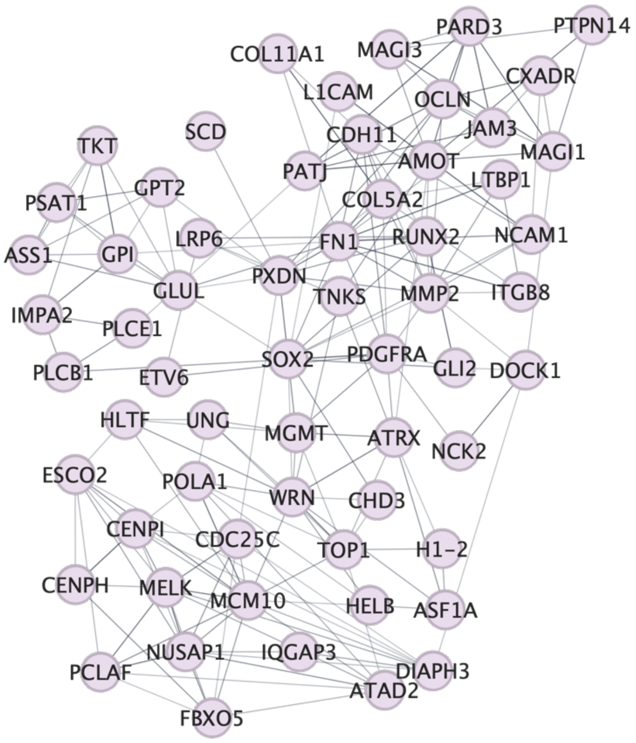

G4L-PROTAC3

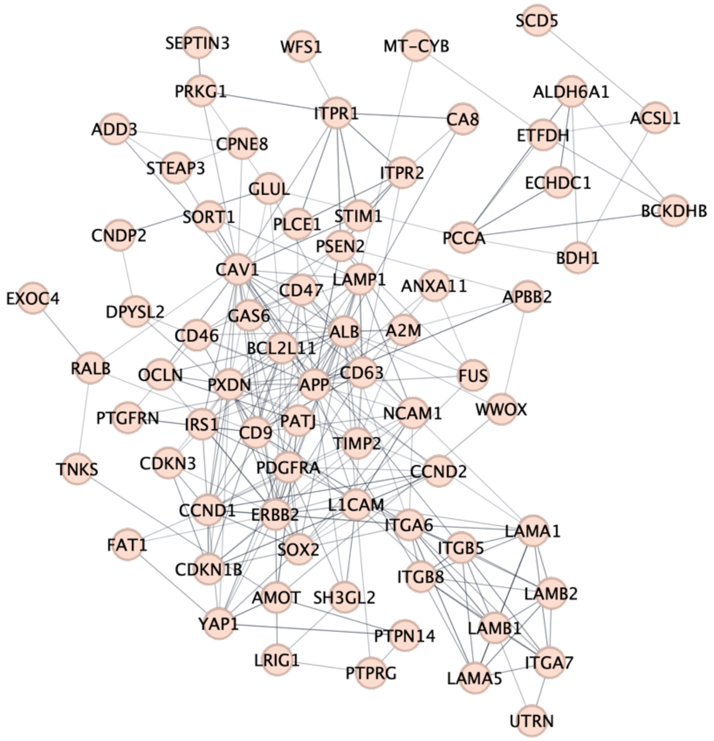

G4L-PROTAC10

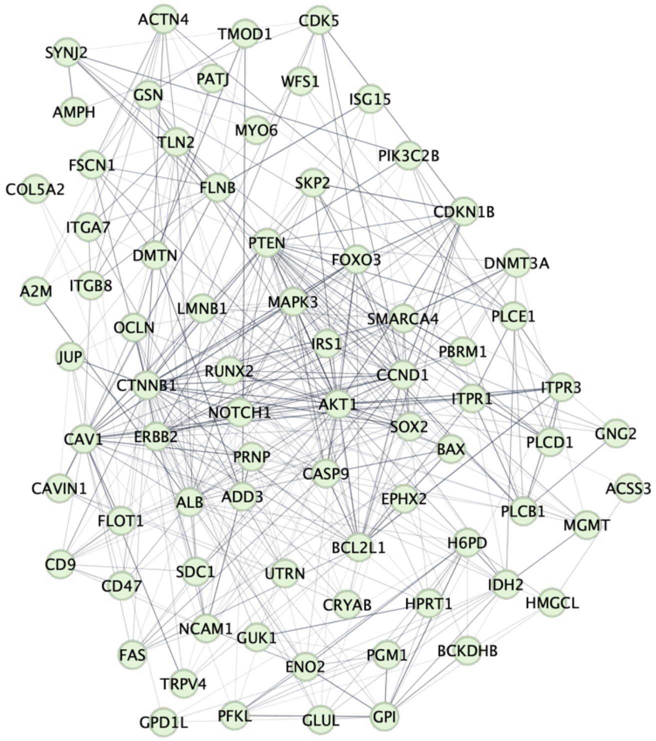

G4L-PROTAC9

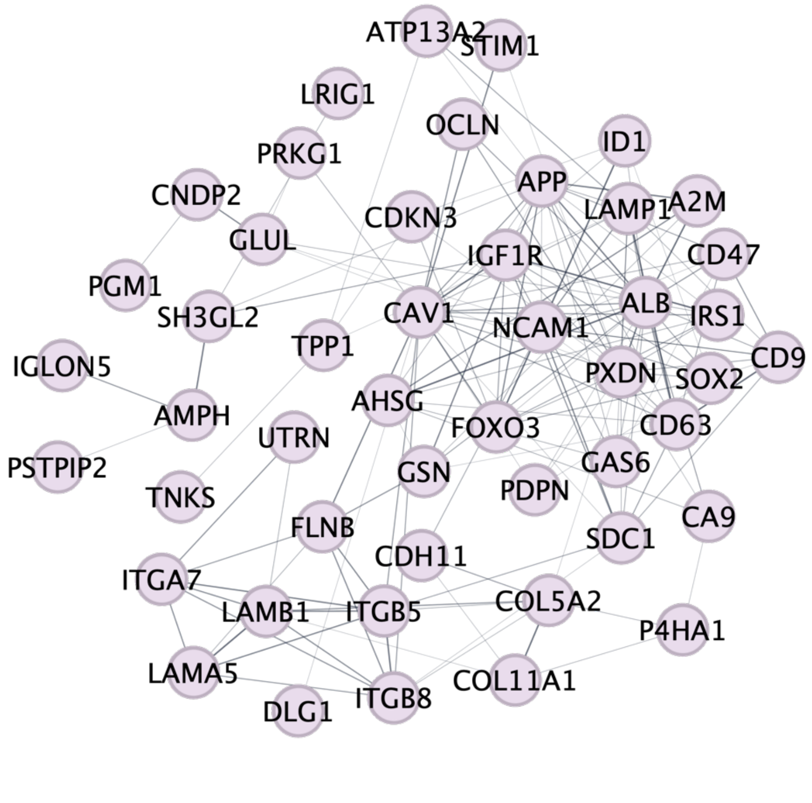

G4L-PROTAC6

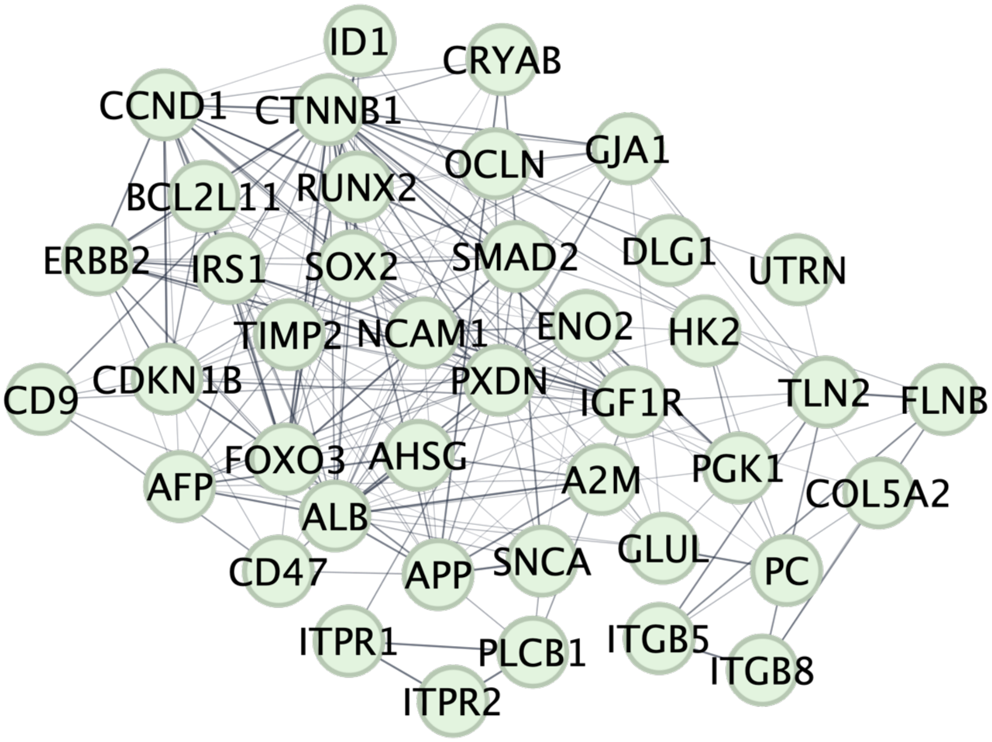

G4L-PROTAC7

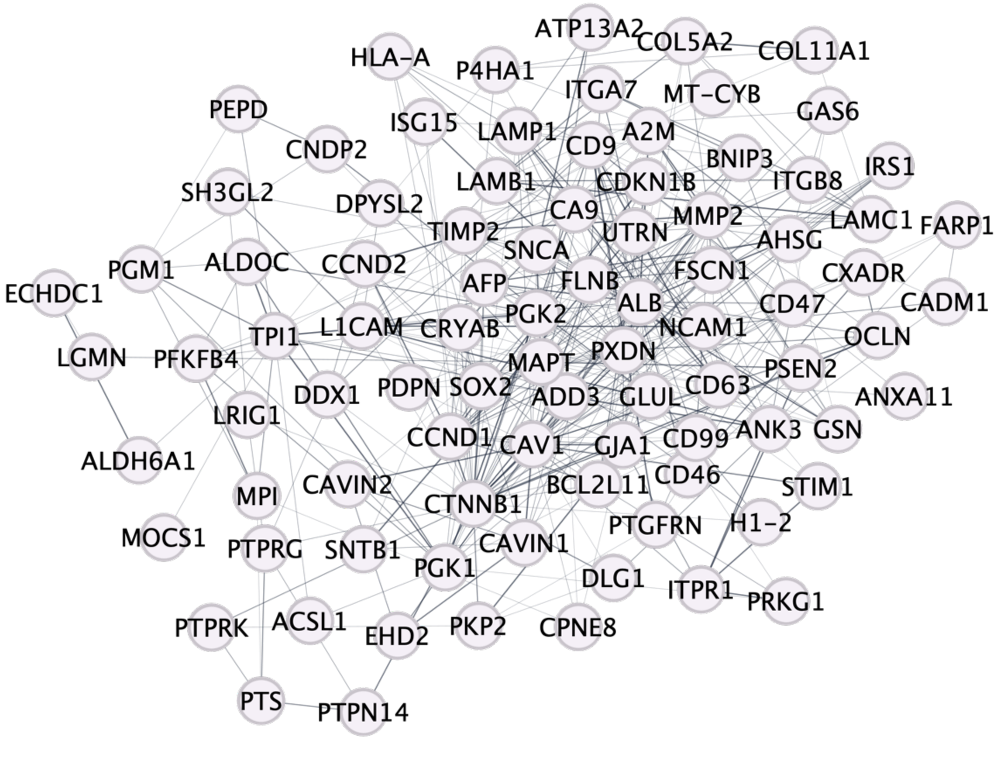

Supplementary Figure 27. G4L-PROTACs induce G2/M cell cycle arrest in multiple cancer cell lines.

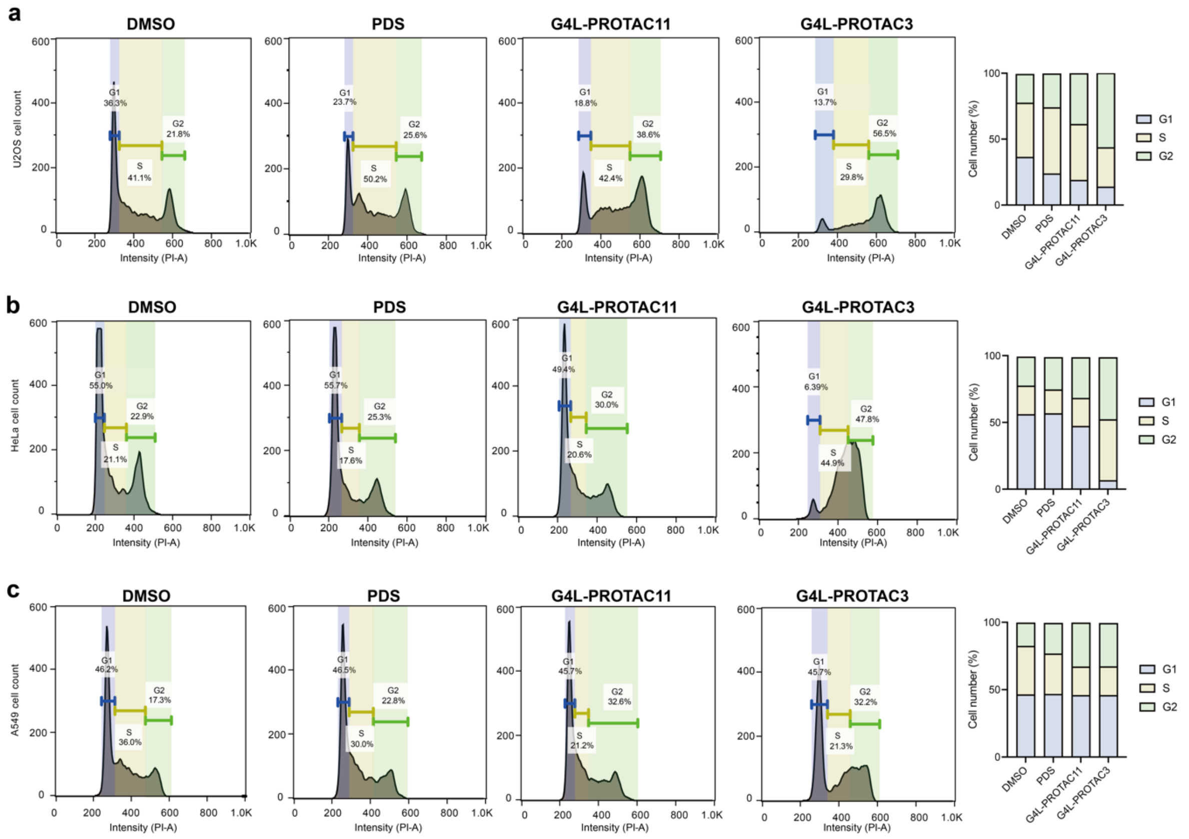

a–c, Flow cytometry analysis of cell cycle distribution in (**a**) U2OS, (**b**) HeLa, and (**c**) A549 cells following 72 h treatment with DMSO (vehicle), PDS (10 µM), G4L-PROTAC11 (10 µM), or G4L-PROTAC3 (10 µM). Cells were stained with propidium iodide using the Tali Cell Cycle Kit and analyzed on a MACSQuant VYB flow cytometer. Representative DNA content histograms show the percentage of cells in G1, S, and G2/M phases, with corresponding quantification bar plots. Both G4L-PROTAC3 and G4L-PROTAC11 induced an increase in G2/M-phase cell population compared to DMSO or PDS treatment, consistent with a G2/M checkpoint response. Endo-Porter (6 µL/mL) was included in all treatment conditions to facilitate compound uptake.

Supplementary Figure 28. Gating strategy for cell cycle analysis.

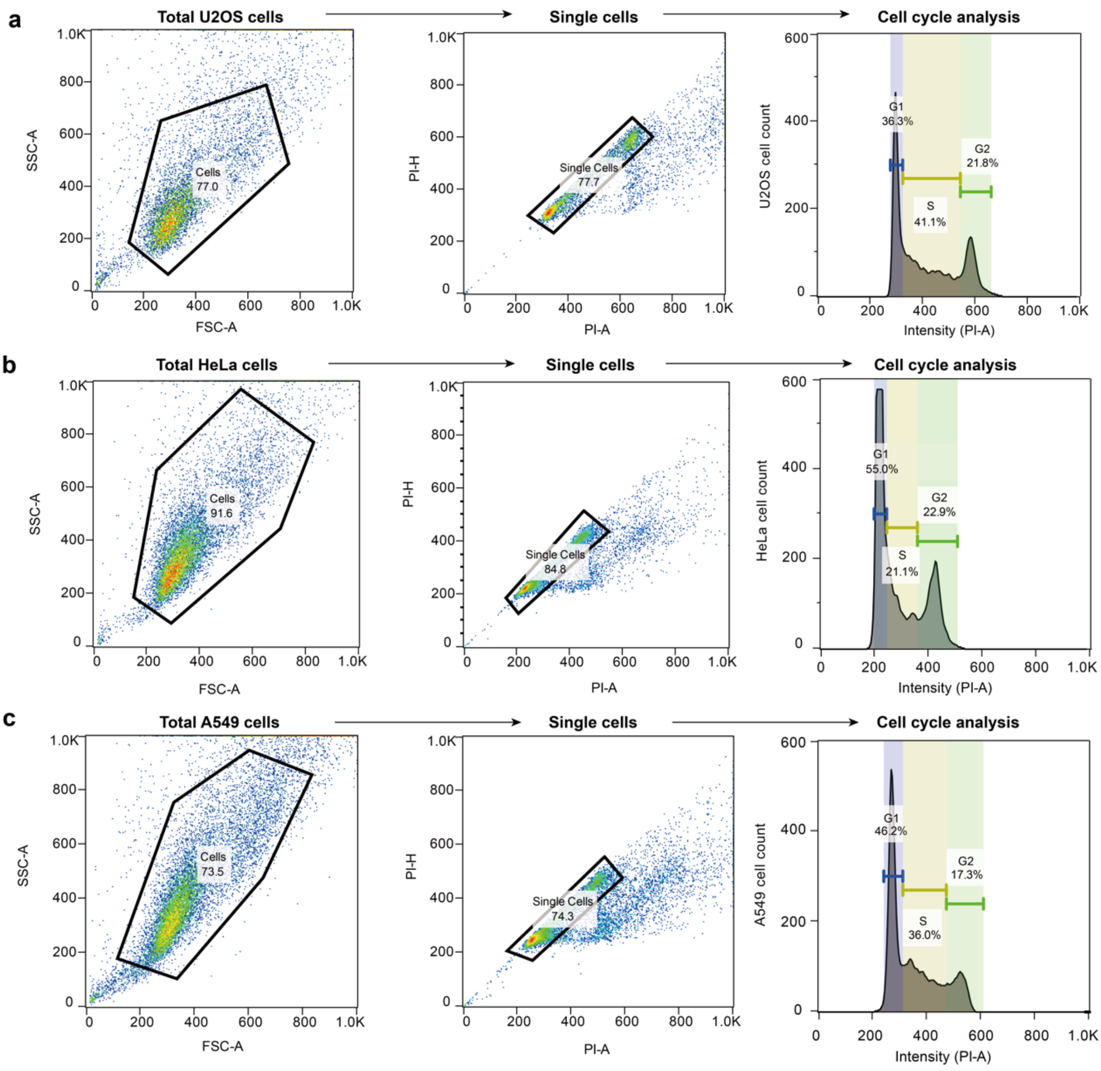

a–c, Representative flow cytometry plots illustrating the gating workflow for identifying cell cycle phases in (**a**) U2OS, (**b**) HeLa, and (**c**) A549 cells. From left to right: total cell populations were first gated based on forward scatter (FSC-A) and side scatter (SSC-A) to exclude debris. Single cells were then selected by gating PI-A versus PI-H to exclude doublets. The final panel shows DNA content histograms generated from propidium iodide staining, with quantification of G1, S, and G2/M phase populations under DMSO treatment as baseline. This gating strategy was consistently applied to all conditions in the cell cycle experiment.

Supplementary Figure 29. Carboxylate-modified G4L-PROTAC analogues exhibit improvements in cellular uptake but limited degradation activity without delivery aid.

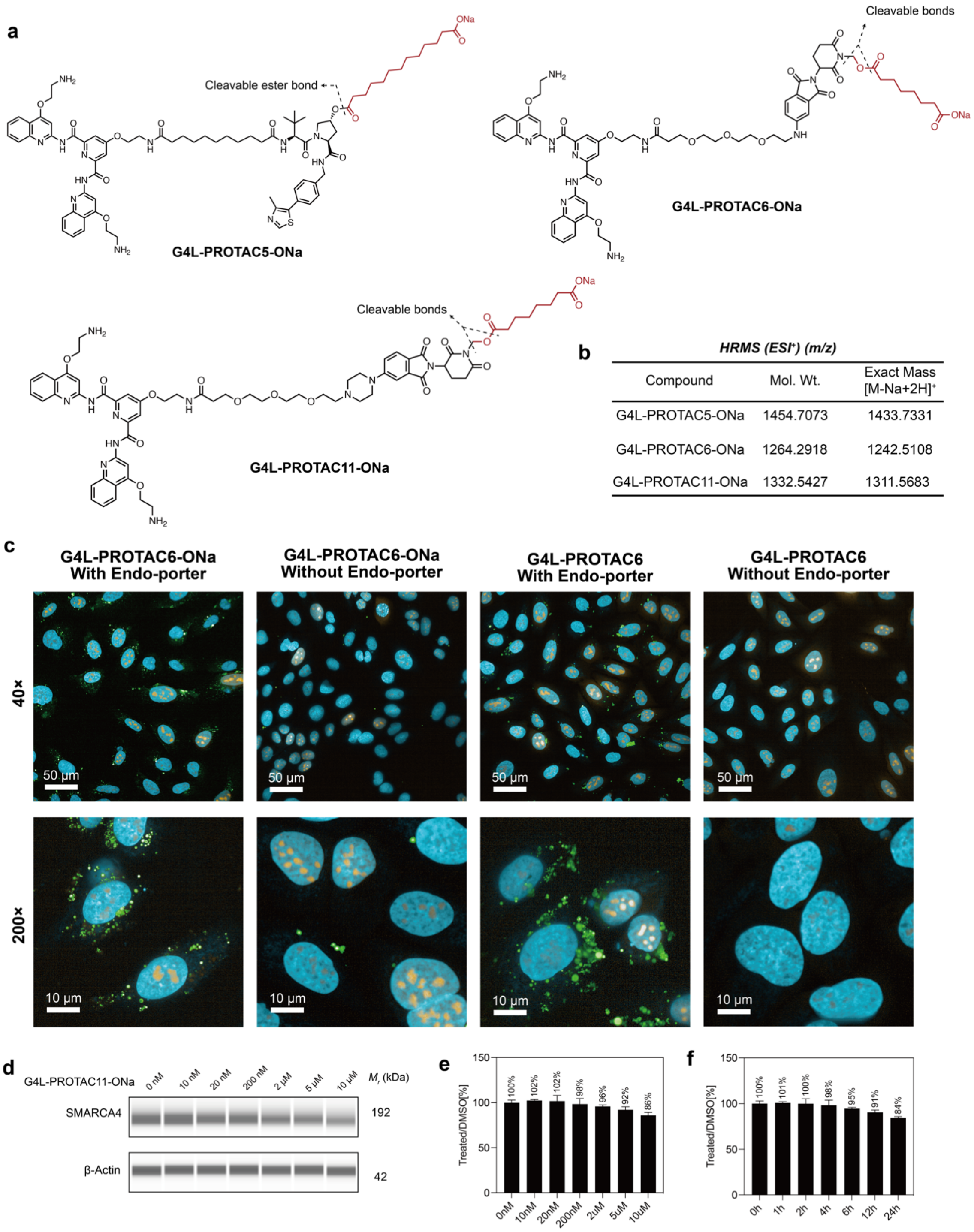

a, Chemical structures of carboxylate-modified analogues G4L-PROTAC5-CNa, G4L-PROTAC6-CNa, and G4L-PROTAC11-CNa bearing cleavable ester-linked –COONa moieties designed to improve solubility and permeability. **b**, High-resolution mass spectrometry (HRMS) analysis confirming the expected exact masses of the synthesized analogues. **c**, Confocal microscopy of U2OS cells treated with fluorescent G4L-PROTAC6-CNa (2 μM, 12 h), with or

without Endo-Porter. While treatment without Endo-Porter shows modest intracellular green fluorescence, uptake remains significantly lower than in Endo-Porter–treated cells. Blue: Hoechst nuclear stain; green: fluorescent G4L-PROTACs. **d**, Western blot analysis of SMARCA4 protein levels in U2OS cells treated with G4L-PROTAC11-CNa at indicated concentrations for 12 h without Endo-Porter. **e**, Quantification of SMARCA4 protein levels relative to β-actin loading control from the blot in d, showing only partial degradation at higher concentrations. **f,** Time-course of SMARCA4 levels in cells treated with 10 µM G4L-PROTAC11-CNa and no Endo-Porter.

Source Data: Supplementary Figure 29. Uncropped Western-blot image.

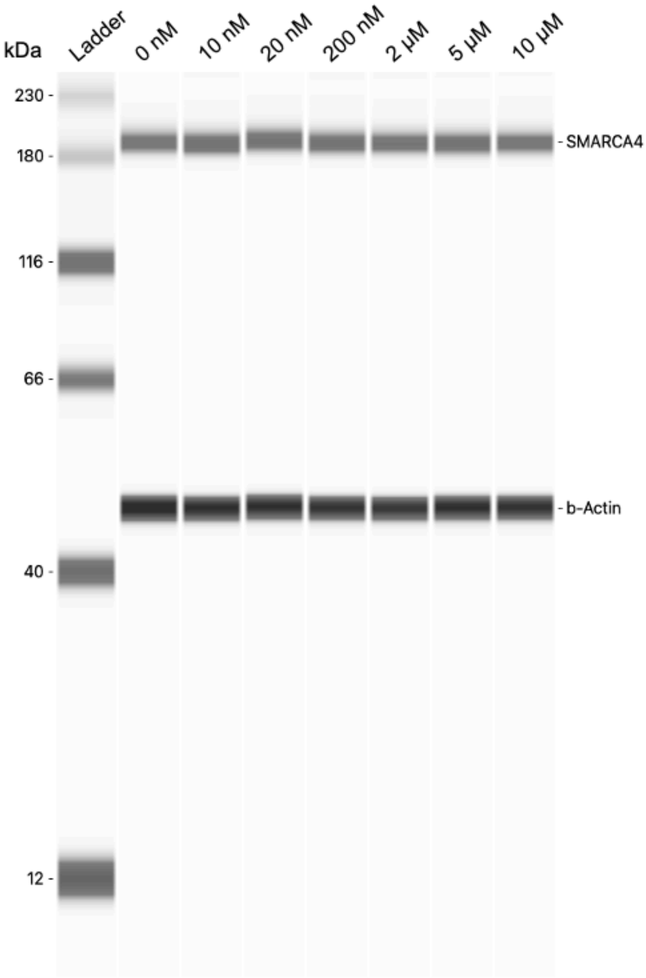

References

1. Zhang, X., Spiegel, J., Martínez Cuesta, S., Adhikari, S. & Balasubramanian, S. Chemical profiling of DNA G-quadruplex-interacting proteins in live cells. *Nat. Chem.* **13**, 626-633 (2021).

2. Shannon, Paul, et al. Cytoscape: a software environment for integrated models of biomolecular interaction networks. *Genome research* **13**, 2498-2504 (2003).

3. Müller, S., Kumari, S., Rodriguez, R. & Balasubramanian, S. Small-molecule-mediated G-quadruplex isolation from human cells. *Nat. Chem.* **2**, 1095–1098 (2010).

4. Le, D. D., Di Antonio, M., Chan, L. K. M. & Balasubramanian, S. G-quadruplex ligands exhibit differential G-tetrad selectivity. *Chem. Commun.* **51**, 8048–8050 (2015).

5. Ambrus, A., Chen, D., Dai, J., Jones, R. A. & Yang, D. Solution structure of the biologically relevant G-quadruplex element in the human c-MYC promoter. Implications for G-quadruplex stabilization. *Biochemistry* **44**, 2048–2058 (2005).

6. Wei, D., Parkinson, G. N., Reszka, A. P. & Neidle, S. Crystal structure of a c-kit promoter quadruplex reveals the structural role of metal ions and water molecules in maintaining loop conformation. *Nucleic Acids Res.* **40**, 4691–4700 (2012).

7. Biffi, G., Tannahill, D., McCafferty, J. & Balasubramanian, S. Quantitative visualization of DNA G-quadruplex structures in human cells. *Nat. Chem.* **5**, 182–186 (2013).
