# Supplementary material for: Degradation of G-quadruplex-binding proteins in chromatin using G4-ligand-based proteolysis-targeting chimeras
Source: Nat Chem. 2026 Mar 19;18(6):1092–101. doi: 10.1038/s41557-026-02111-y (PMC13236602; doi:10.1038/s41557-026-02111-y)
Supplement: Supplementary file 2 — Reporting Summary [file 41557_2026_2111_MOESM2_ESM.pdf]

nature portfolio

Corresponding author(s): Shankar Balasubramanian
Last updated by author(s): Feb 13, 2026

Reporting Summary

Nature Portfolio wishes to improve the reproducibility of the work that we publish. This form provides structure for consistency and transparency in reporting. For further information on Nature Portfolio policies, see our Editorial Policies and the Editorial Policy Checklist.

Statistics

For all statistical analyses, confirm that the following items are present in the figure legend, table legend, main text, or Methods section.

n/a	Confirmed	
☐	☒	The exact sample size (*n*) for each experimental group/condition, given as a discrete number and unit of measurement
☐	☒	A statement on whether measurements were taken from distinct samples or whether the same sample was measured repeatedly
☐	☒	The statistical test(s) used AND whether they are one- or two-sided<br>*Only common tests should be described solely by name; describe more complex techniques in the Methods section.*
☒	☐	A description of all covariates tested
☒	☐	A description of any assumptions or corrections, such as tests of normality and adjustment for multiple comparisons
☐	☒	A full description of the statistical parameters including central tendency (e.g. means) or other basic estimates (e.g. regression coefficient) AND variation (e.g. standard deviation) or associated estimates of uncertainty (e.g. confidence intervals)
☐	☒	For null hypothesis testing, the test statistic (e.g. *F*, *t*, *r*) with confidence intervals, effect sizes, degrees of freedom and *P* value noted<br>*Give P values as exact values whenever suitable.*
☒	☐	For Bayesian analysis, information on the choice of priors and Markov chain Monte Carlo settings
☒	☐	For hierarchical and complex designs, identification of the appropriate level for tests and full reporting of outcomes
☒	☐	Estimates of effect sizes (e.g. Cohen's *d*, Pearson's *r*), indicating how they were calculated

Our web collection on statistics for biologists contains articles on many of the points above.

Software and code

Policy information about availability of computer code

Data collection	NMR data were collected on a Bruker 400 MHz Avance III HD Spectrometer and a 500 MHz Avance III Smart Probe Spectrometer.<br>HRMS data were collected on a Waters LCT Premier (ESI) spectrometer.<br>Measurements of restoring FAM signal from FRET melting assay were recorded on a Bio-Rad CFX96 Touch Real-Time PCR Detection System.<br>Fluorescence quench binding assay End-point fluorescence was measured on a fluorescence plate reader (BMG PHERAstar Plus).<br>The cell staining images were collected on a Bio-Rad ChemiDoc MP system.<br>A Bio-Rad CFX96 Touch Real-Time PCR Detection System was used for concentration quantification of sequencing libraries.<br>Sequencing data were collected on a NextSeq 2000 sequencer (Illumina).
Data analysis	NMR data were processed in MestReNova (version 12.0.1).<br>FRET and Fluorescence quench binding assay data were processed and analysed in GraphPad Prism 10 (version 10.2.2).<br>Cell staining images were processed in software Fiji (version 2.14.0/1.54f).<br>Bioinformatics data analyses and processing were performed using Bash, R (version 4.1) and Python (version >=3.4) programming languages.<br>The following tools were also used: demuxIllumina (version 3.0.9), FastQC (version 0.11.8), MultiQC (version 1.11), cutadapt (version 1.18), BWA (version 0.7.17-r1188), Picard (version 2.20.3), Seacr (version 1.3), EnhancedVolcano (v.1.16.0), deepTools (version 2.0), DiffBind (version 3.10.1), Gencode v37.<br>The analysis scripts are available on the GitHub page dedicated to this study: https://github.com/sblab-informatics/G4L-PROTACs

For manuscripts utilizing custom algorithms or software that are central to the research but not yet described in published literature, software must be made available to editors and reviewers. We strongly encourage code deposition in a community repository (e.g. GitHub). See the Nature Portfolio guidelines for submitting code & software for further information.

Data

Policy information about availability of data

All manuscripts must include a data availability statement. This statement should provide the following information, where applicable:

- Accession codes, unique identifiers, or web links for publicly available datasets
- A description of any restrictions on data availability
- For clinical datasets or third party data, please ensure that the statement adheres to our policy

TMT-labelled quantitative proteomics data documented in the supplementary data file Supplementary_Data_G4L_PROTAC, which contains peptide intensities, metadata and enriched proteins from the G4L-PROTACs versus Negative Control statistical comparisons. SMARCA4 and FUS CUT&Tag data are available at NCBI GEO (accession number GSE296701). BG4 CUT&Tag data were generated previously and are available under accession number GSE181373. The mass spectrometry proteomics data have been deposited to the ProteomeXchange Consortium via the PRIDE partner repository with the dataset identifier PXD073248. Source data are provided with this paper.

Research involving human participants, their data, or biological material

Policy information about studies with human participants or human data. See also policy information about sex, gender (identity/presentation), and sexual orientation and race, ethnicity and racism.

Reporting on sex and gender	n/a
Reporting on race, ethnicity, or other socially relevant groupings	n/a
Population characteristics	n/a
Recruitment	n/a
Ethics oversight	n/a

Note that full information on the approval of the study protocol must also be provided in the manuscript.

Field-specific reporting

Please select the one below that is the best fit for your research. If you are not sure, read the appropriate sections before making your selection.

☒ Life sciences ☐ Behavioural & social sciences ☐ Ecological, evolutionary & environmental sciences

For a reference copy of the document with all sections, see nature.com/documents/nr-reporting-summary-flat.pdf

Life sciences study design

All studies must disclose on these points even when the disclosure is negative.

Sample size	According to a previous Chem-map study (Yu, Z. et al. Nat. Biotechnol. 41, 1265–1271 (2023)), 600,000 cells per sample were used for mapping SMARCA4 and FUS binding sites; the same number was used in this study. For crystal violet staining experiments, 1,000 cells per well in 6-well plates were seeded to ensure appropriate confluence for quantification. For Western blot experiments, 50,000 cells per well were seeded in 6-well plates to ensure adequate protein extraction for analysis using the Jess automated Western blot system (ProteinSimple) following the manufacturer's protocol (https://www.bio-techne.com/instruments/simple-western).
Data exclusions	No data were excluded from analyses in this study.
Replication	SMARCA4 and FUS CUT&Tag experiments were performed in two biological replicates with two technical replicates each, and showed consistent results. For Western blot experiments, similar results were obtained in three independent biological replicates. SOX2 and SNRNP70 ELISA experiments include three technical replicates each and yielded consistent Kd values. Proteomic analyses were performed on at least three independent biological replicates. All experiments were reliably reproducible.
Randomization	No randomization was performed, as control conditions were run in parallel to avoid systematic measurement bias.
Blinding	No blinding was performed, as control conditions were run in parallel to avoid systematic measurement bias.

Reporting for specific materials, systems and methods

We require information from authors about some types of materials, experimental systems and methods used in many studies. Here, indicate whether each material, system or method listed is relevant to your study. If you are not sure if a list item applies to your research, read the appropriate section before selecting a response.

Materials & experimental systems

n/a	Involved in the study
☐	☒ Antibodies
☐	☒ Eukaryotic cell lines
☒	☐ Palaeontology and archaeology
☒	☐ Animals and other organisms
☒	☐ Clinical data
☒	☐ Dual use research of concern
☒	☐ Plants

Methods

n/a	Involved in the study
☒	☐ ChIP-seq
☐	☒ Flow cytometry
☒	☐ MRI-based neuroimaging

Antibodies

Antibodies used

Guinea Pig anti-Rabbit IgG secondary antibody (antibodies-online, catalogue no. ABIN101961), BG4 (scFv) was expressed as previously described (Biffi, G. et al. Nat. Chem. 5, 182–186 (2013); Hänsel-Hertsch, R. et al. Nat. Protoc. 13, 551–564 (2018)), rabbit anti-FLAG antibody (Cell Signaling Technology, catalogue no. 2368S), rabbit anti-γH2AX (Cell Signaling Technology, catalogue no. 2577), rabbit anti-PARP1 (Cell Signaling Technology, catalogue no. 9532), rabbit anti-β-Actin (Cell Signaling Technology, catalogue no. 4970), rabbit anti-GAPDH (Cell Signaling Technology, catalogue no. D16H11), mouse anti-FUS (Santa Cruz, catalogue no. sc-47711), rabbit anti-SMARCA4 (Abcam, catalogue no. ab110641), rabbit anti-ubiquitin (Abcam, catalogue no. ab70462), rabbit anti-SOX2 (Merck, catalogue no. AB5603), and rabbit anti-SNRNP70 (Abcam, catalogue no. ab83306) were used for western blot and/or CUT&Tag analysis.

Validation

All commercial antibodies were validated by the manufacturers or ourselves according to a previous study. Guinea Pig anti-Rabbit IgG secondary antibody (antibodies-online, catalogue no. ABIN101961) was tested for ELISA, immunohistochemistry, Western blot, CUT&RUN, and CUT&Tag in rabbit samples and preadsorbed to minimize cross-reactivity with other species. Rabbit anti-FLAG antibody (Cell Signaling Technology, catalogue no. 2368S) was validated by the manufacturer for Western Blot, immunoprecipitation, and immunofluorescence in human samples. Rabbit anti-γH2AX (Cell Signaling Technology, catalogue no. 2577), PARP1 (catalogue no. 9532), β-Actin (catalogue no. 4970), and GAPDH (catalogue no. D16H11) antibodies were validated by the manufacturer for use in western blot and immunofluorescence across a range of mammalian species. Mouse anti-FUS (Santa Cruz, catalogue no. sc-47711) was validated for western blot in human and mouse samples. Rabbit anti-SMARCA4 (Abcam, catalogue no. ab110641), anti-ubiquitin (Abcam, catalogue no. ab70462), anti-SOX2 (Merck, catalogue no. AB5603), and anti-SNRNP70 (Abcam, catalogue no. ab83306) were validated for western blot or immunoprecipitation as per the manufacturer's protocols. We additionally validated the binding specificity and affinity of BG4 (scFv) to folded G4 DNA oligonucleotides by ELISA, as previously described (Biffi, G. et al., Nat. Chem. 5, 182–186, 2013).

Eukaryotic cell lines

Policy information about cell lines and Sex and Gender in Research

Cell line source(s)

Human osteosarcoma U2OS cells (HTB-96), human cervical cancer HeLa cells (CCL-2), and human lung carcinoma A549 cells (CCL-185) were purchased from ATCC.

Authentication

Short tandem repeat (STR) profiling was used to distinguish between individual human cell lines and rule out intra-species contamination. This was performed by the CRUK Cambridge Institute Biorepository Core Facility.

Mycoplasma contamination

Cells were confirmed mycoplasma-free by periodic tests based on RNA-capture ELISA performed by the CRUK Cambridge Institute Biorepository Core Facility.

Commonly misidentified lines
(See ICLAC register)

No commonly misidentified cell lines were used.

Plants

Seed stocks

Report on the source of all seed stocks or other plant material used. If applicable, state the seed stock centre and catalogue number. If plant specimens were collected from the field, describe the collection location, date and sampling procedures.

Novel plant genotypes

Describe the methods by which all novel plant genotypes were produced. This includes those generated by transgenic approaches, gene editing, chemical/radiation-based mutagenesis and hybridization. For transgenic lines, describe the transformation method, the number of independent lines analyzed and the generation upon which experiments were performed. For gene-edited lines, describe the editor used, the endogenous sequence targeted for editing, the targeting guide RNA sequence (if applicable) and how the editor was applied.

Authentication

Describe any authentication procedures for each seed stock used or novel genotype generated. Describe any experiments used to assess the effect of a mutation and, where applicable, how potential secondary effects (e.g. second site T-DNA insertions, mosiacism, off-target gene editing) were examined.

Flow Cytometry

Plots

Confirm that:

☒ The axis labels state the marker and fluorochrome used (e.g. CD4-FITC).

☒ The axis scales are clearly visible. Include numbers along axes only for bottom left plot of group (a 'group' is an analysis of identical markers).

☒ All plots are contour plots with outliers or pseudocolor plots.

☒ A numerical value for number of cells or percentage (with statistics) is provided.

Methodology

Sample preparation	U2OS cells were transfected with plasmids encoding SG4-GFP or G4P-RFP fusion constructs and cultured under standard conditions. After 24 hours, cells were treated with G4L-PROTAC2 at various concentrations (20nM, 200nM, 2µM, 5µM, 10µM) for 6 hours. Cells were then harvested, washed with PBS, and resuspended in FACS buffer (PBS + 2% FBS) for flow cytometric analysis.
Instrument	Flow cytometry was performed using a CytoFlex SRT cell sorter (Beckman Coulter).
Software	Data acquisition and analysis were conducted using CytExpert software (Beckman Coulter). Post-analysis plots and statistics were generated using FlowJo (v10.8).
Cell population abundance	Fluorescence intensity of GFP or RFP was used to quantify the abundance of SG4-GFP and G4P-RFP expressing cells, respectively. Percentages of GFP+ or RFP+ cells were determined from pseudocolor dot plots and used to assess the degradation efficiency of G4L-PROTAC2 at each dose.
Gating strategy	Forward and side scatter (FSC/SSC) parameters were used to identify the viable single-cell population. Gates for GFP+ or RFP+ cells were set based on untreated control samples. Dot plots display GFP (FL7-A) versus RFP (FL4-A) for dual-marker discrimination, with quadrant gates used to distinguish positive and negative populations.

☒ Tick this box to confirm that a figure exemplifying the gating strategy is provided in the Supplementary Information.
