# Supplementary material for: Degradation of G-quadruplex-binding proteins in chromatin using G4-ligand-based proteolysis-targeting chimeras
Source: Nat Chem. 2026 Mar 19;18(6):1092–101. doi: 10.1038/s41557-026-02111-y (PMC13236602; doi:10.1038/s41557-026-02111-y)

Fig. 3a

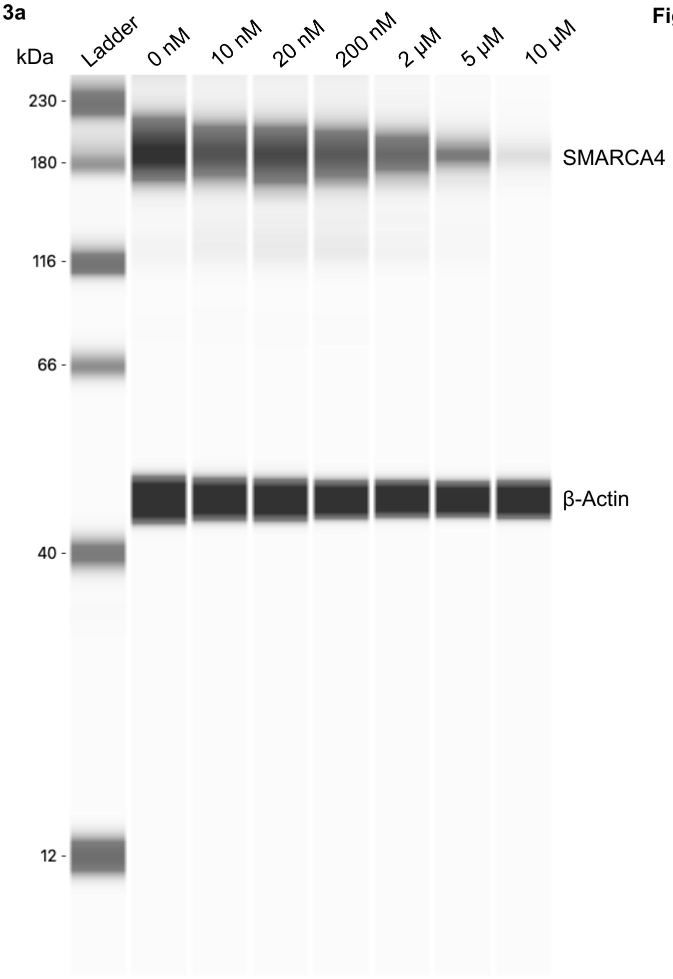

Fig. 3b

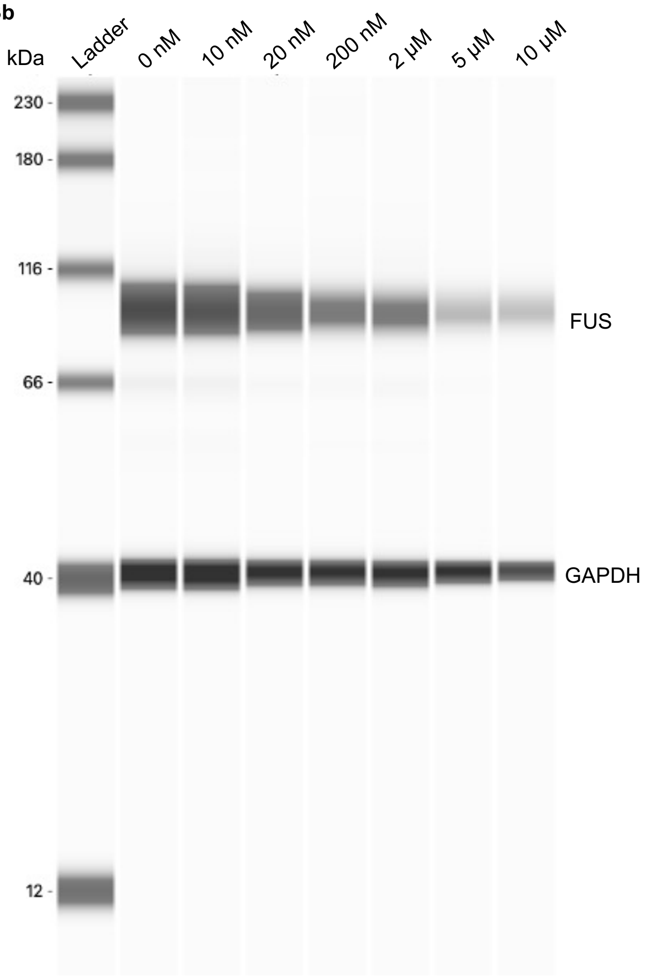

Fig. 3c

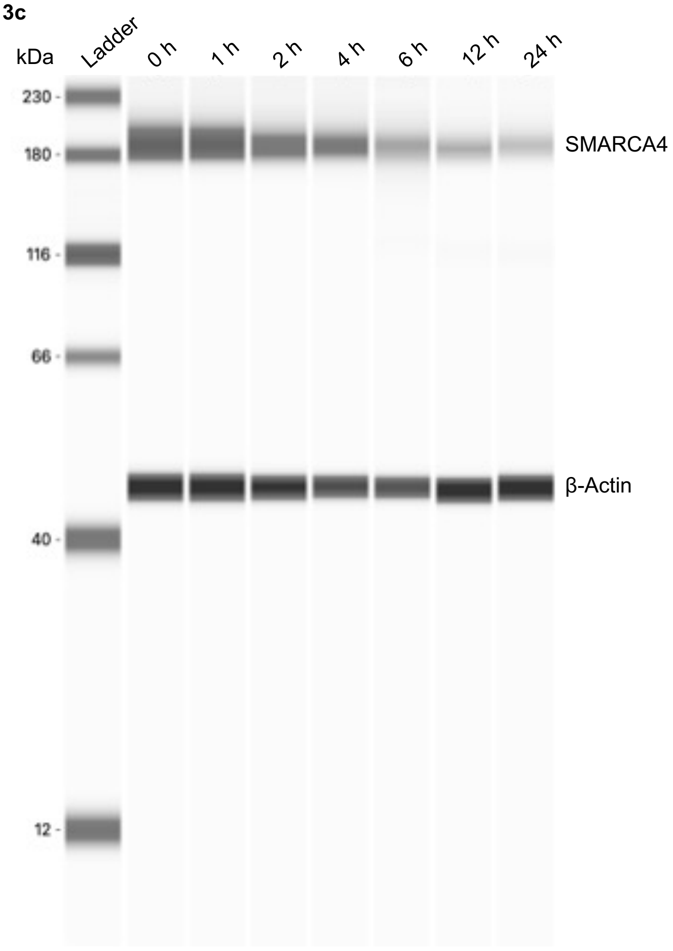

Fig. 3d

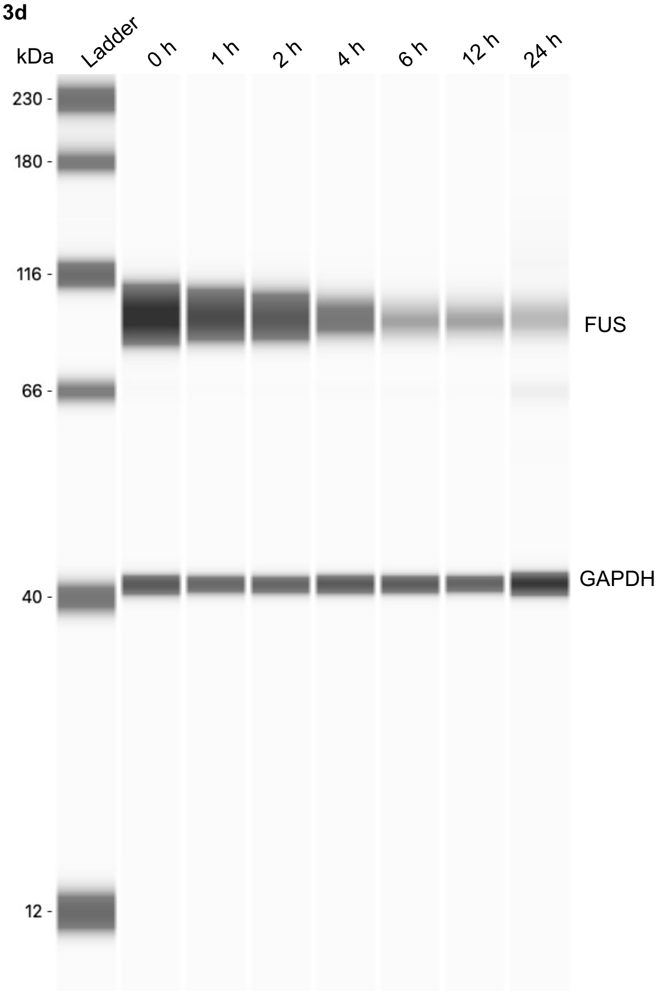

Fig. 3e

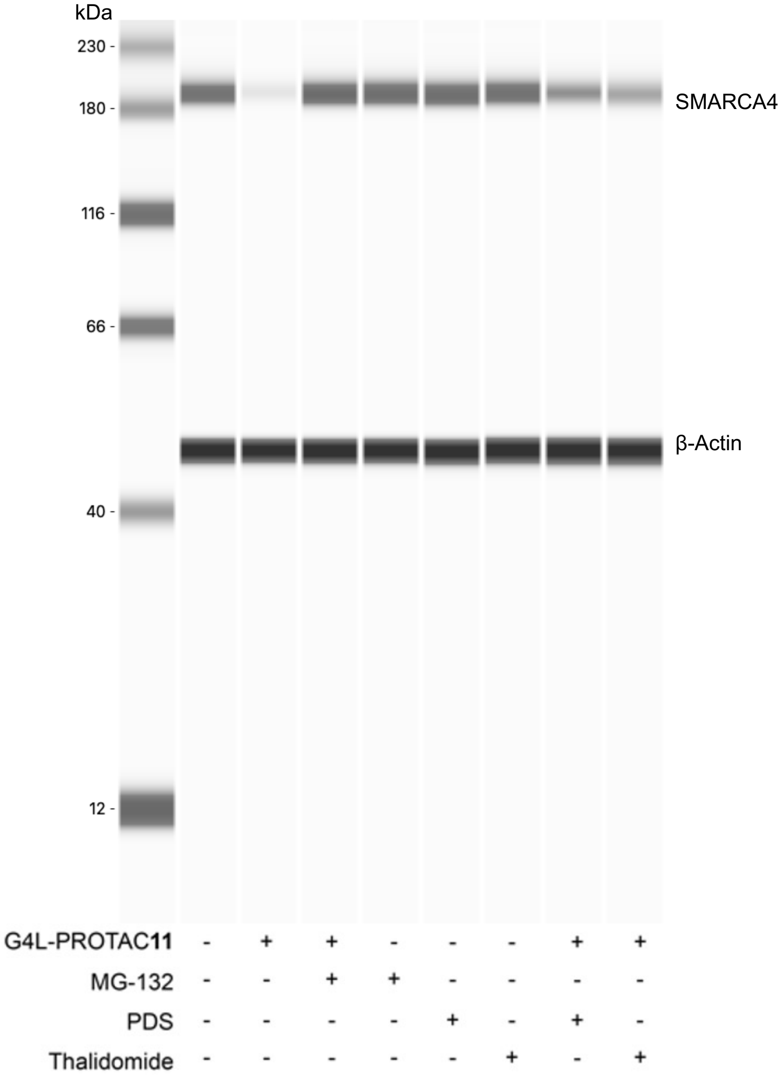
kDa
230
180
116
66
40
12
SMARCA4
β-Actin
G4L-PROTAC11 - + + - - - + +
MG-132 - - + + - - - -
PDS - - - - + - + -
Thalidomide - - - - - + - +

Fig. 3f

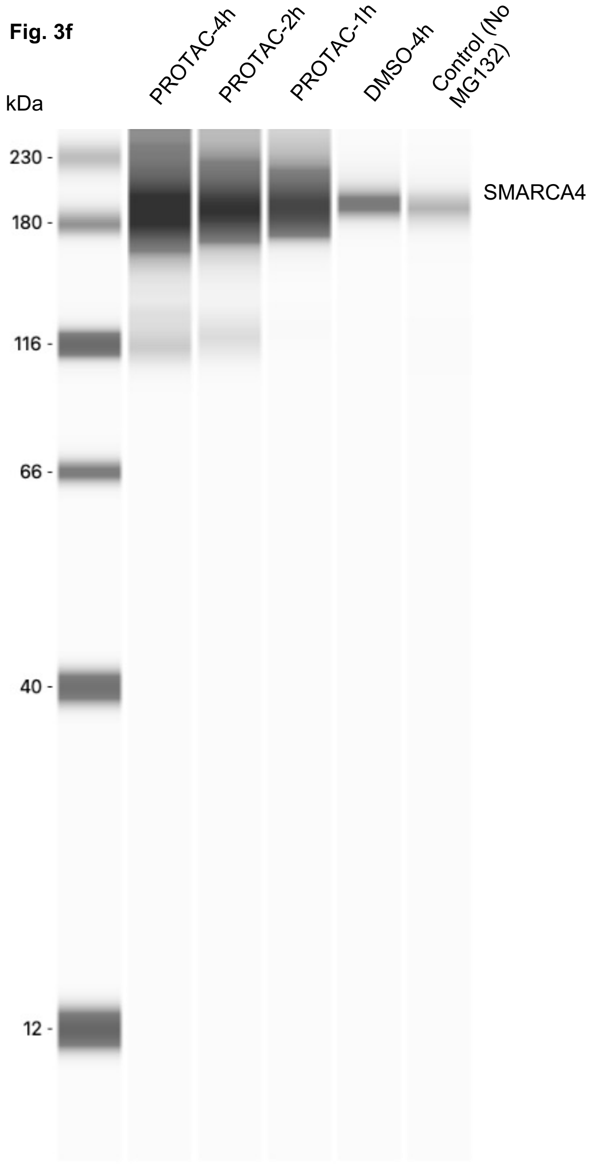
PROTAC-4h
PROTAC-2h
PROTAC-1h
DMSO-4h
Control (No MG132)
kDa
230
180
116
66
40
12
SMARCA4

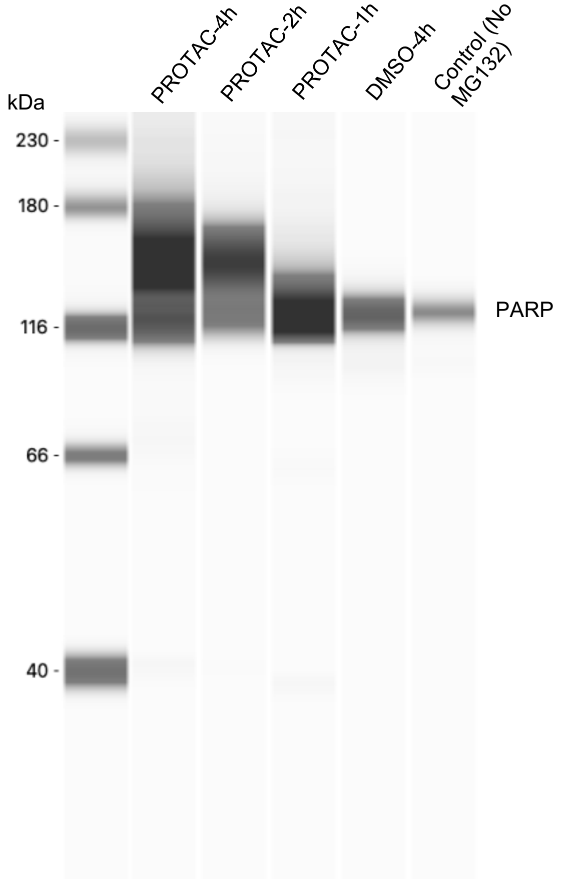
PROTAC-4h
PROTAC-2h
PROTAC-1h
DMSO-4h
Control (No MG132)
kDa
230
180
116
66
40
PARP

Fig. 3g

	G4L-PROTAC11 treated		Control (PDS)
**Differential**	**Peak gain**	**Peak loss**	**-**
**G4 sites**	690	7878	11894
**Non-G4 sites**	2115	4171	4101
**Total**	2805	12049	15995

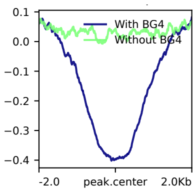

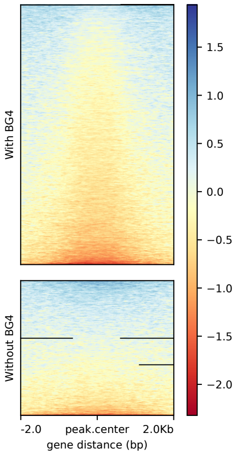

Step1: generate log2FC profile of SMARCA4 CnT between PROTAC and PDS control.

Step2: extract G4 and non-G4 peaks for PROTAC and PDS control, respectively.

Step3: visualize the log2FC profile around G4 and non-G4 peaks from PDS control.

Step4: visualize the log2FC profile around G4 and non-G4 peaks from G4L-PROTAC.

Fig. 3h

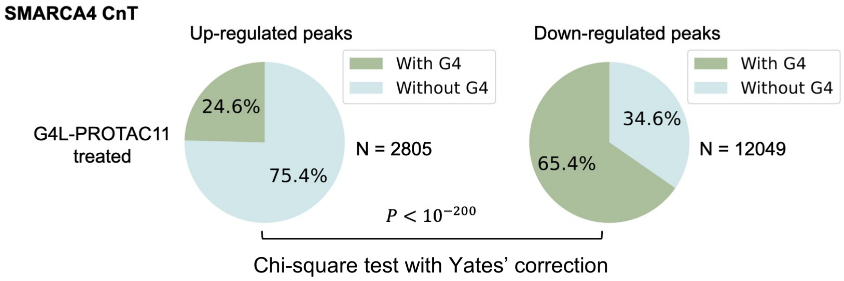

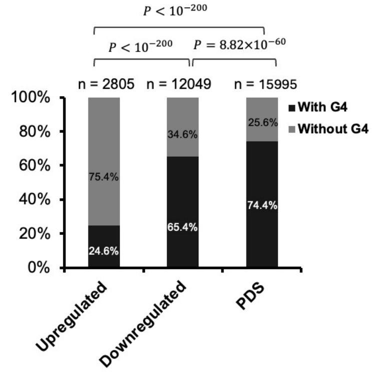

Fig. 3i

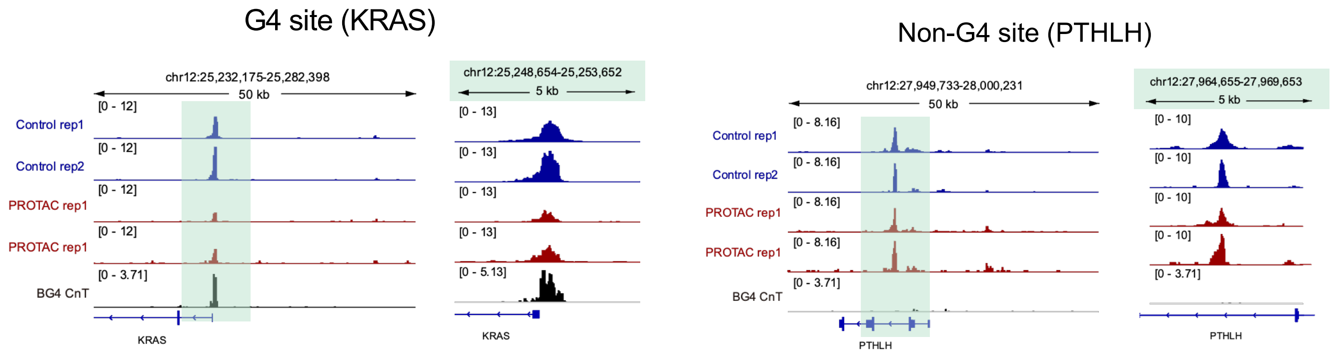

Supplement: Supplementary file 4 — Unprocessed western blots. [file 41557_2026_2111_MOESM4_ESM.pdf]
