# Supplementary material for: Degradation of G-quadruplex-binding proteins in chromatin using G4-ligand-based proteolysis-targeting chimeras
Source: Nat Chem. 2026 Mar 19;18(6):1092–101. doi: 10.1038/s41557-026-02111-y (PMC13236602; doi:10.1038/s41557-026-02111-y)
Supplement: Supplementary file 6 — Unprocessed image. [file 41557_2026_2111_MOESM6_ESM.pdf]

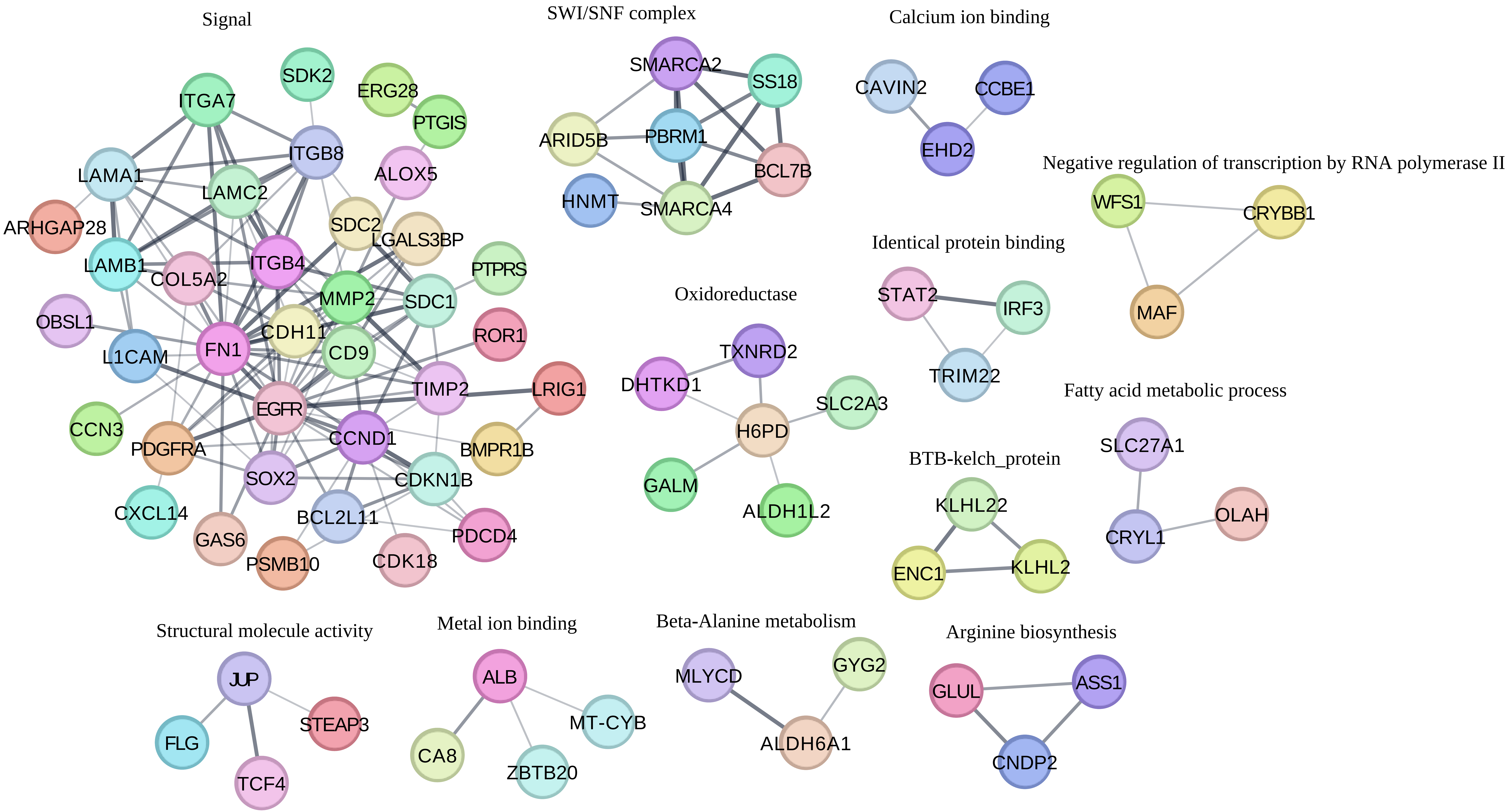

Signal
SDK2
ERG28
ITGA7
PTGIS
ITGB8
LAMA1
ALOX5
LAMC2
ARHGAP28
SDC2
LGALS3BP
ITGB4
LAMB1
PTPRS
COL5A2
MMP2
SDC1
OBSL1
CDH11
ROR1
FN1
CD9
L1CAM
TIMP2
LRIG1
EGFR
CCN3
CCND1
BMPR1B
PDGFRA
SOX2
CDKN1B
CXCL14
BCL2L11
PDCD4
GAS6
CDK18
PSMB10
SWI/SNF complex
SMARCA2
SS18
ARID5B
PBRM1
BCL7B
HNMT
SMARCA4
Calcium ion binding
CAVIN2
CCBE1
EHD2
Negative regulation of transcription by RNA polymerase II
WFS1
CRYBB1
MAF
Identical protein binding
STAT2
IRF3
TRIM22
Oxidoreductase
TXNRD2
DHTKD1
SLC2A3
H6PD
GALM
ALDH1L2
Fatty acid metabolic process
SLC27A1
BTB-kelch_protein
KLHL22
CRYL1
OLAH
ENC1
KLHL2
Structural molecule activity
JUP
STEAP3
FLG
TCF4
Metal ion binding
ALB
MT-CYB
CA8
ZBTB20
Beta-Alanine metabolism
MLYCD
GYG2
ALDH6A1
Arginine biosynthesis
GLUL
ASS1
CNDP2
