# Supplementary figures and images for: Degradation of G-quadruplex-binding proteins in chromatin using G4-ligand-based proteolysis-targeting chimeras
Source: Nat Chem. 2026 Mar 19;18(6):1092–101. doi: 10.1038/s41557-026-02111-y (PMC13236602; doi:10.1038/s41557-026-02111-y)

Fig. 6c

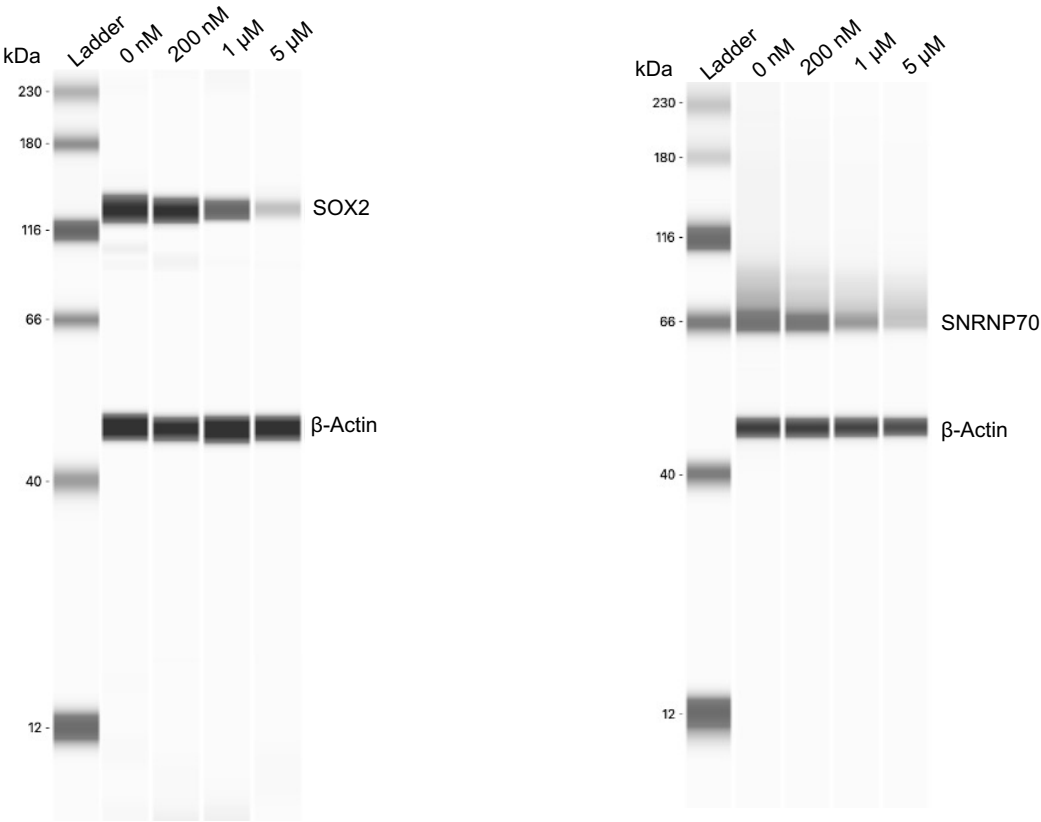

Fig. 6e

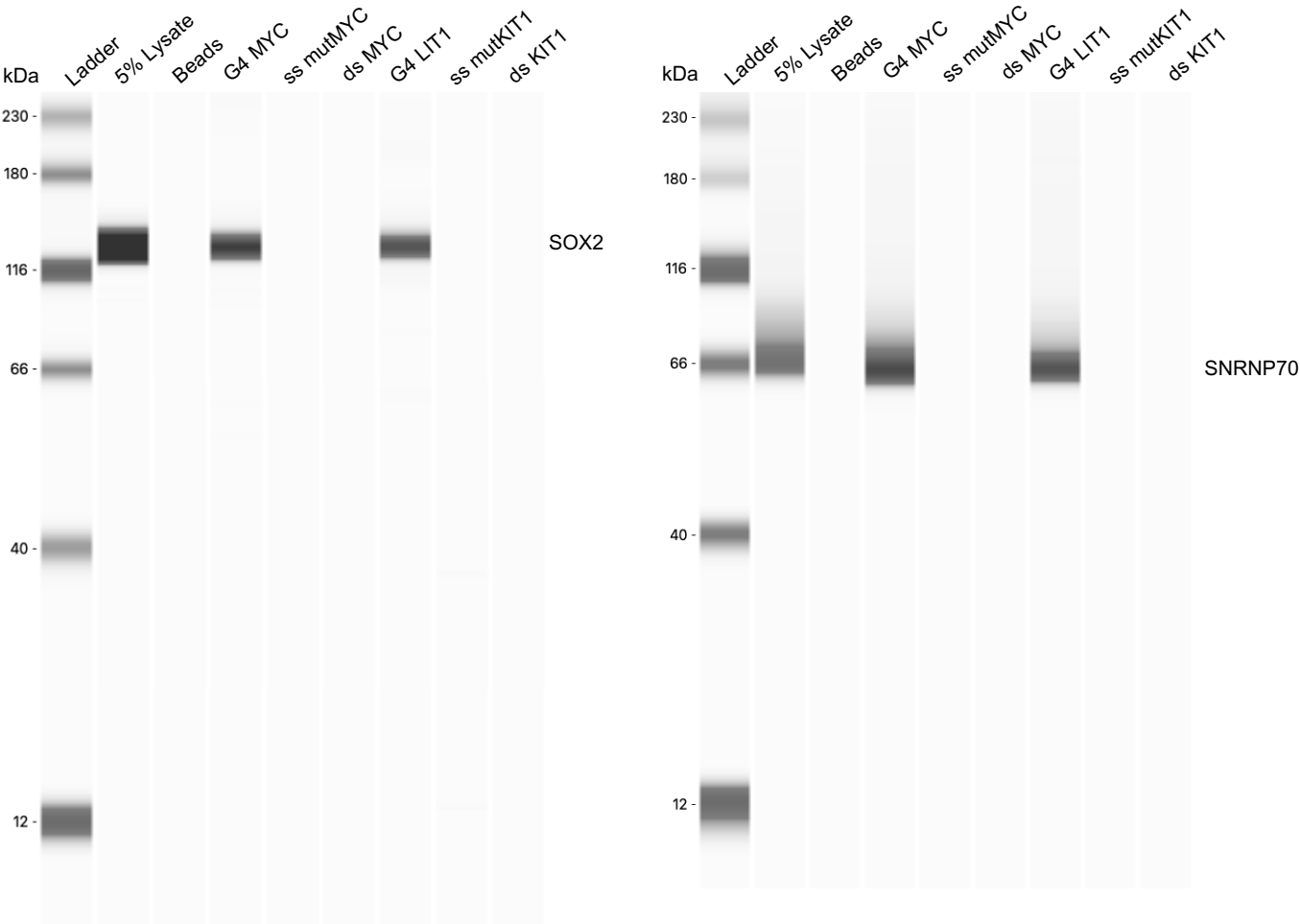

Supplement: Supplementary file 9 — Unprocessed western blots. [file 41557_2026_2111_MOESM9_ESM.pdf]

Extended Data Fig. 3a

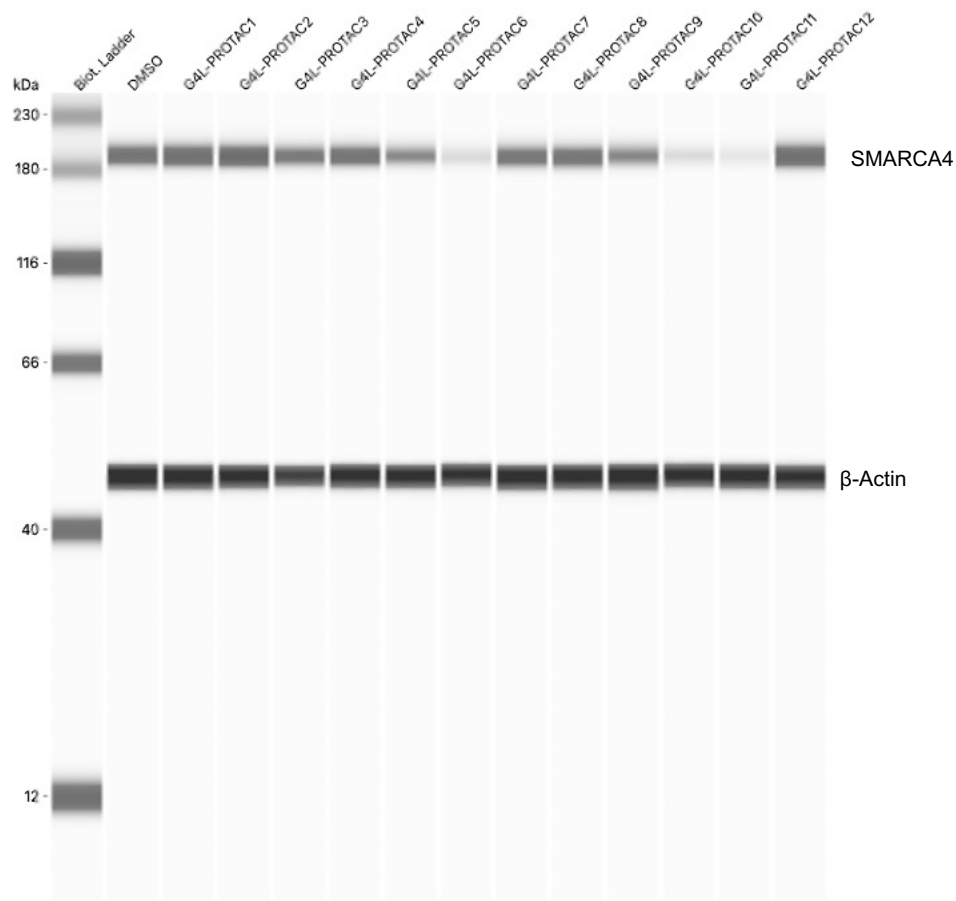

Extended Data Fig. 3c

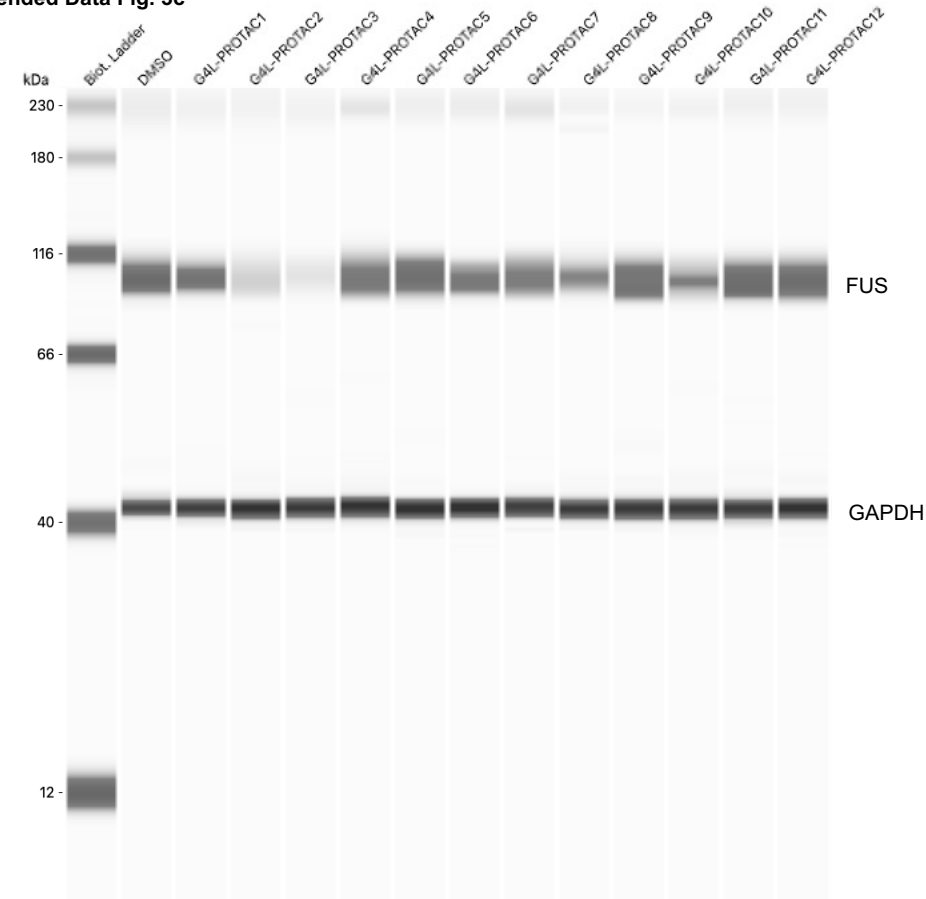

Extended Data Fig. 3h

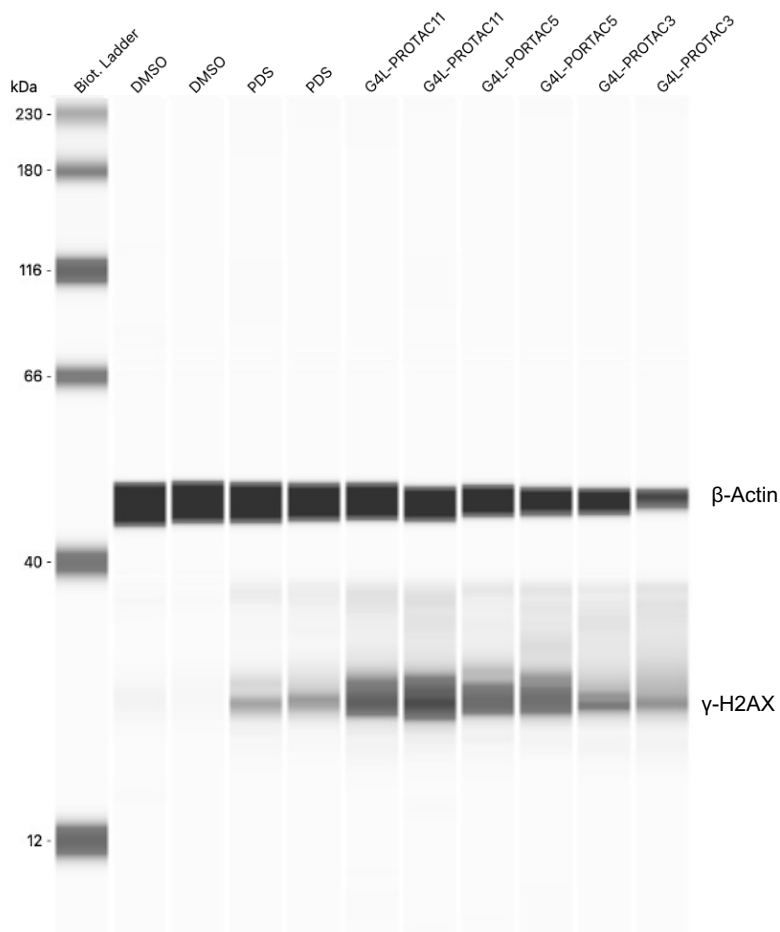

Extended Data Fig. 3i

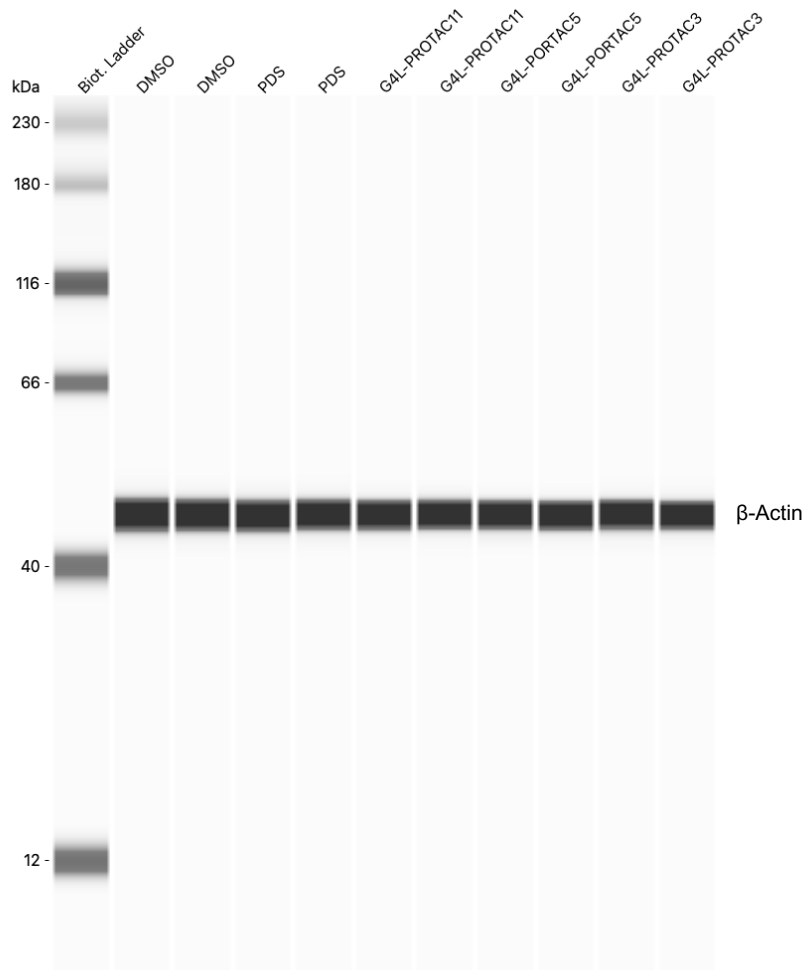

Supplement: Supplementary file 12 — Unprocessed western blots. [file 41557_2026_2111_MOESM12_ESM.pdf]
