# Supplementary material for: Degradation of G-quadruplex-binding proteins in chromatin using G4-ligand-based proteolysis-targeting chimeras
Source: Nat Chem. 2026 Mar 19;18(6):1092–101. doi: 10.1038/s41557-026-02111-y (PMC13236602; doi:10.1038/s41557-026-02111-y)

Extended Data Fig. 4a

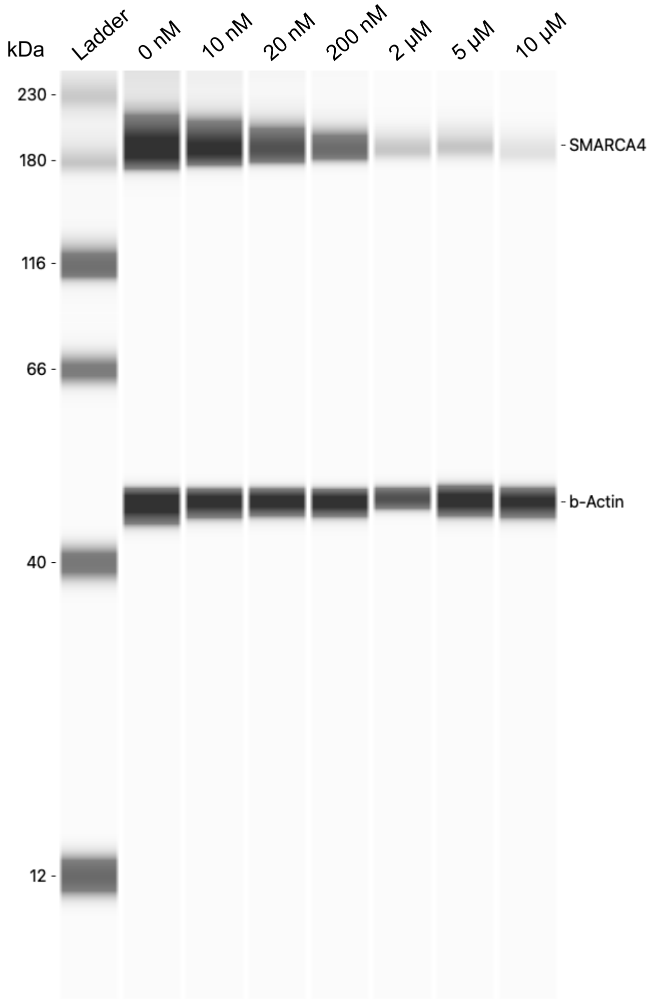

Extended Data Fig. 4b

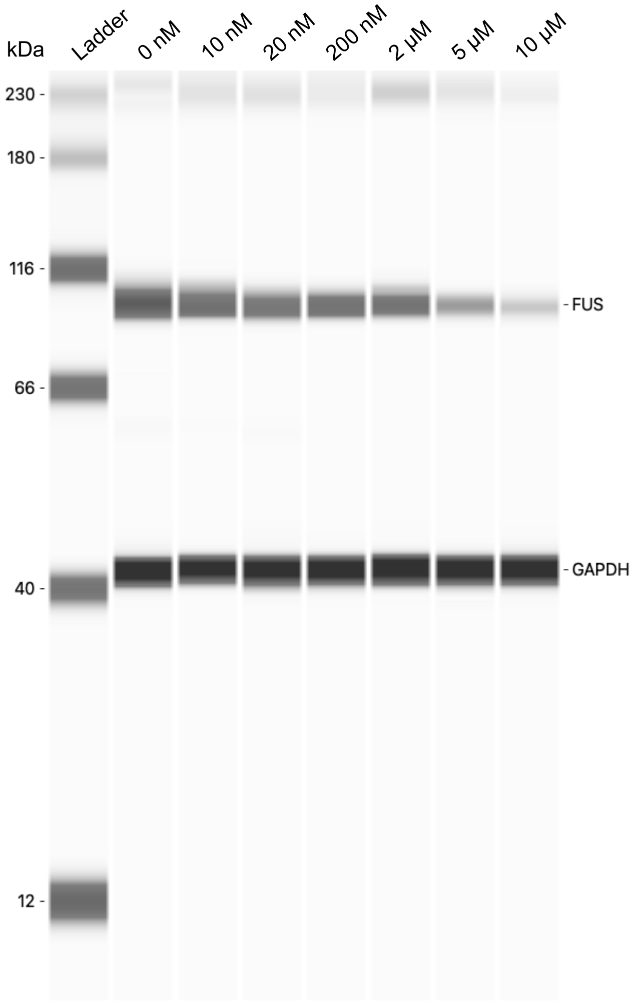

Extended Data Fig. 4c

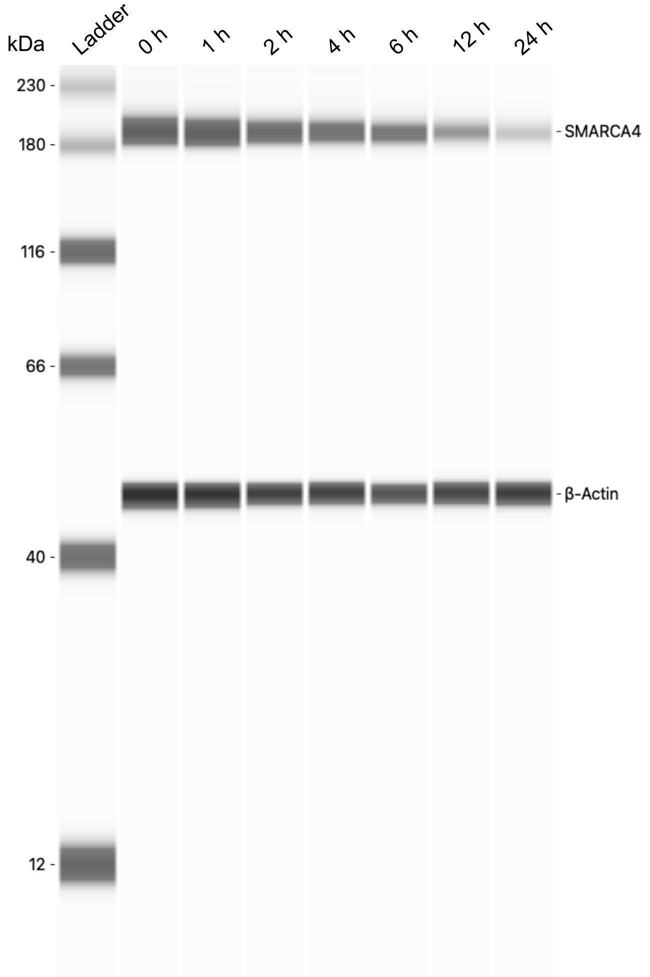

Extended Data Fig. 4d

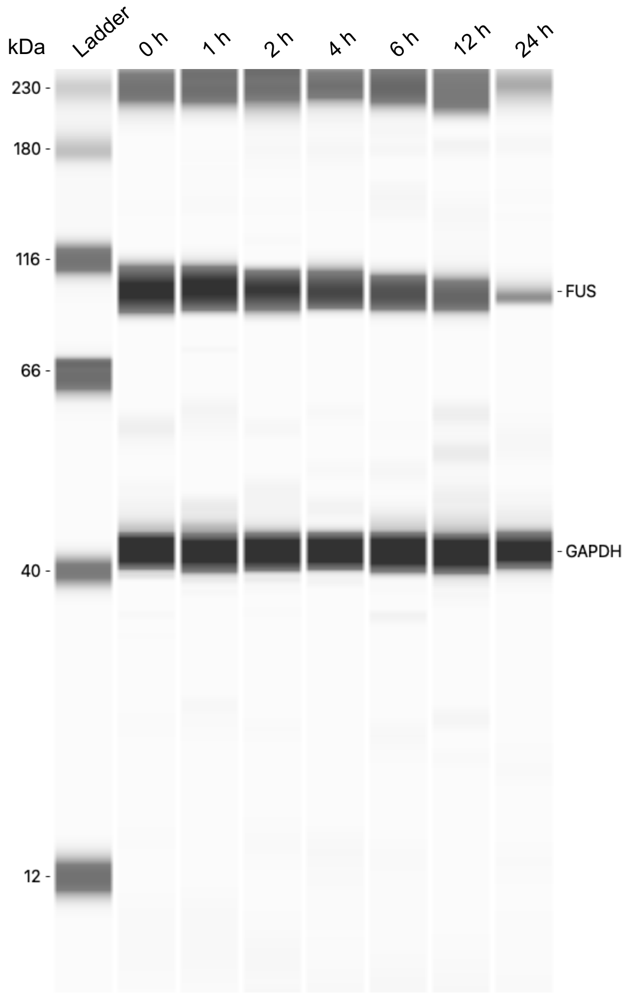

Extended Data Fig. 4e

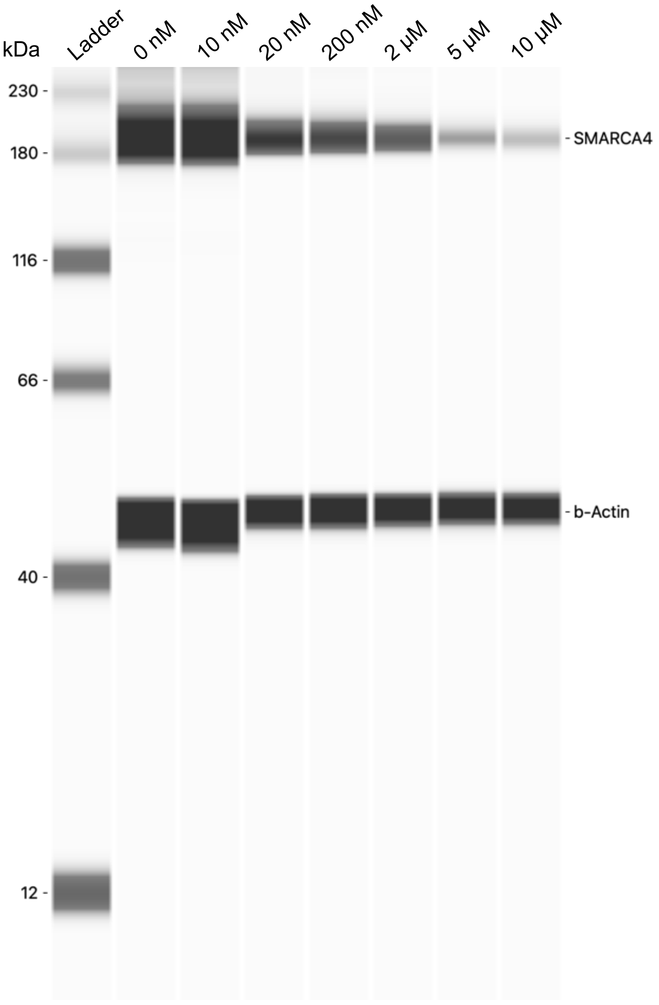

Extended Data Fig. 4f

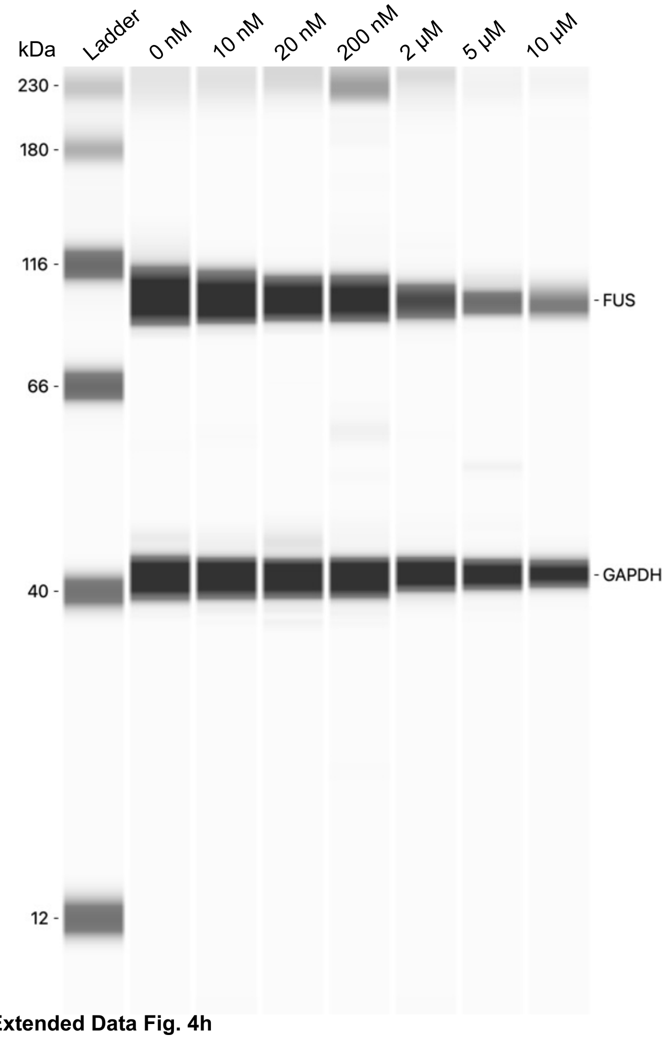

Extended Data Fig. 4g

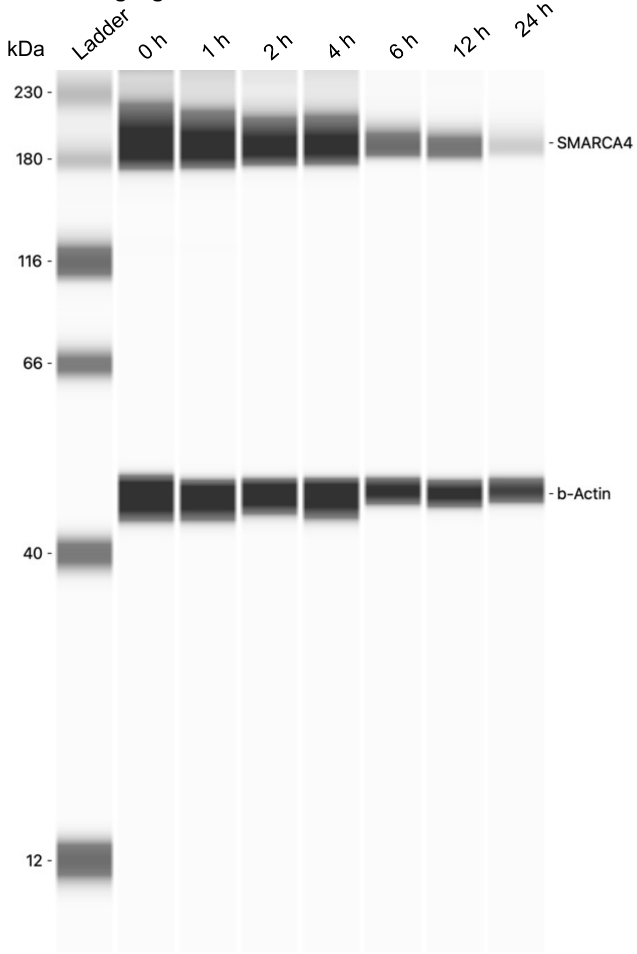

Extended Data Fig. 4h

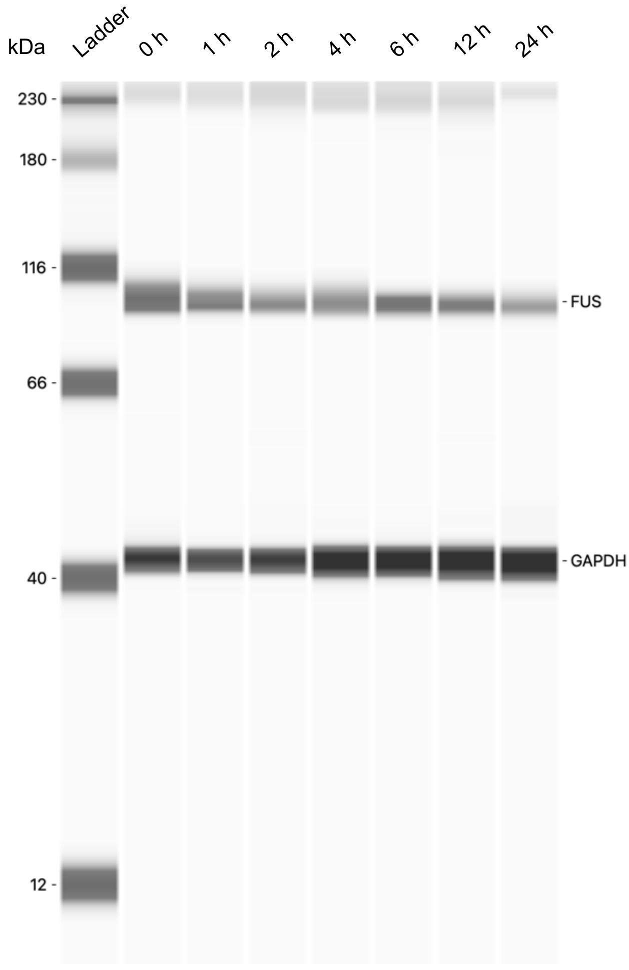

Extended Data Fig. 4i

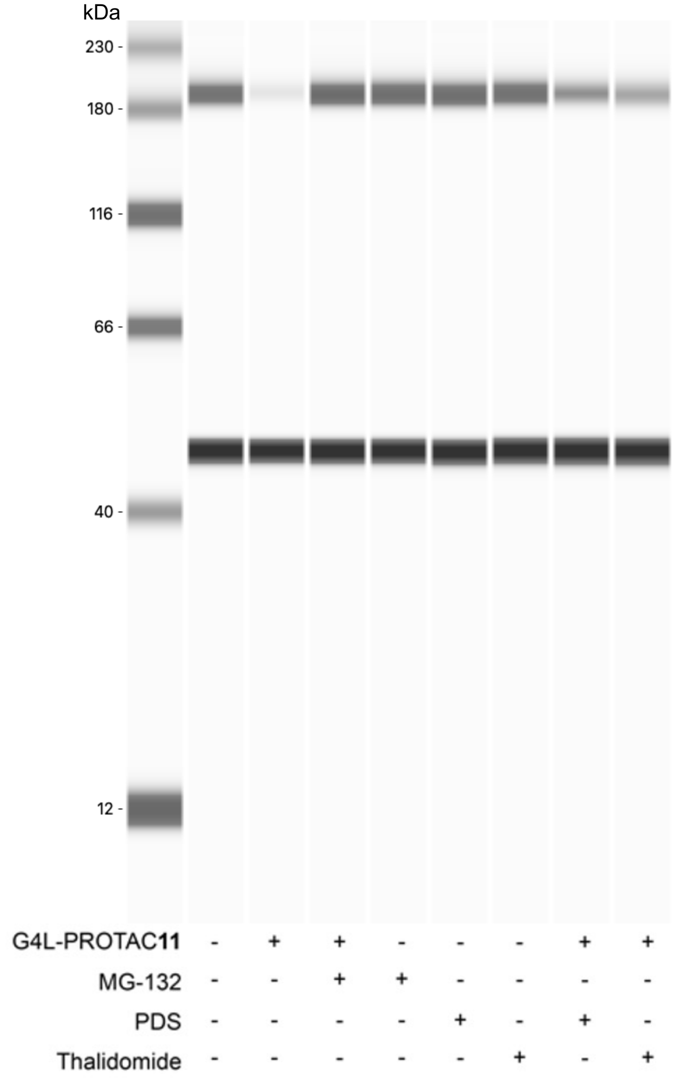

Extended Data Fig. 4j

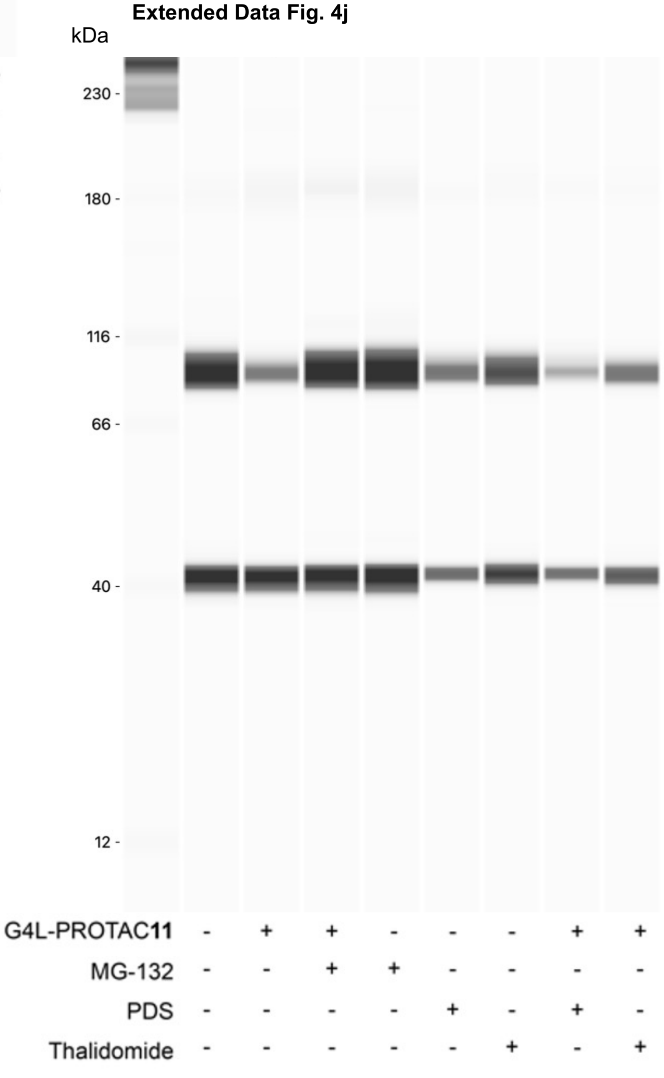

Supplement: Supplementary file 14 — Unprocessed western blots. [file 41557_2026_2111_MOESM14_ESM.pdf]
