# Supplementary material for: Degradation of G-quadruplex-binding proteins in chromatin using G4-ligand-based proteolysis-targeting chimeras
Source: Nat Chem. 2026 Mar 19;18(6):1092–101. doi: 10.1038/s41557-026-02111-y (PMC13236602; doi:10.1038/s41557-026-02111-y)
Supplement: Supplementary file 17 — Unprocessed image. [file 41557_2026_2111_MOESM17_ESM.pdf]

Extended Data Fig. 6f

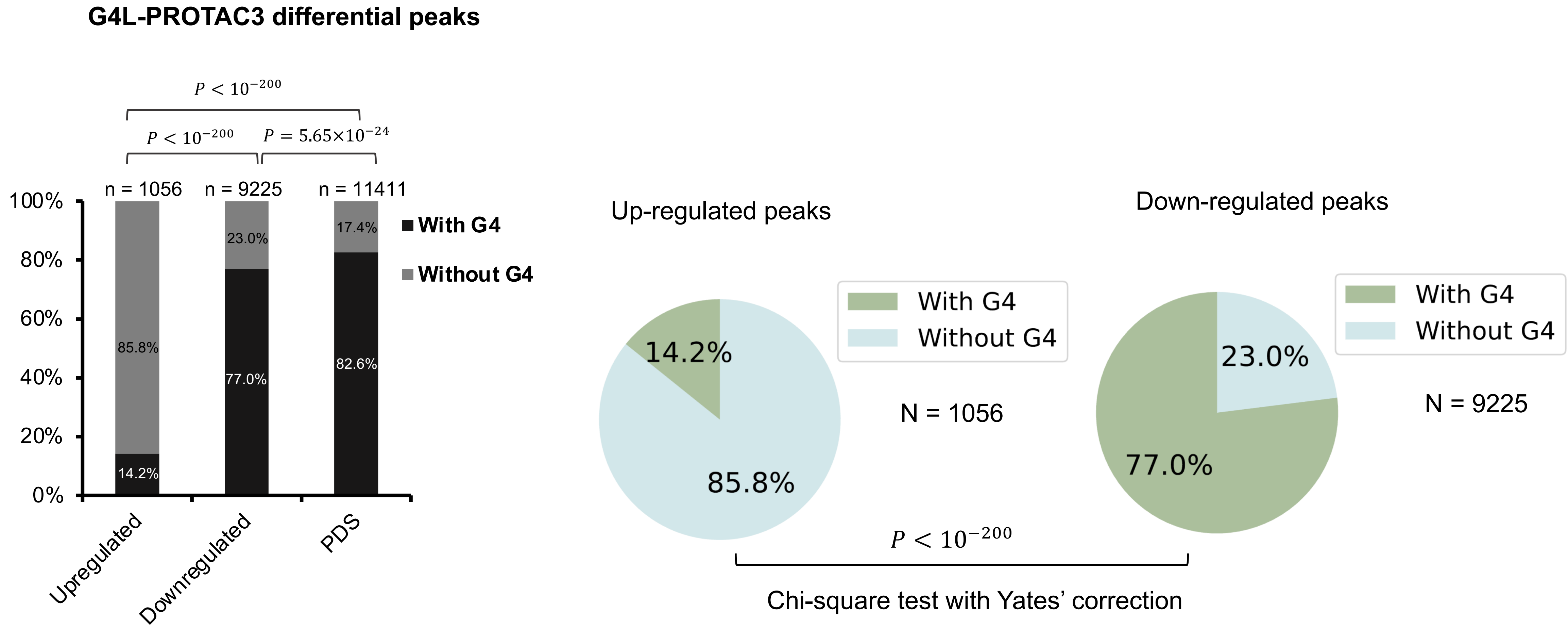

Extended Data Fig. 6e

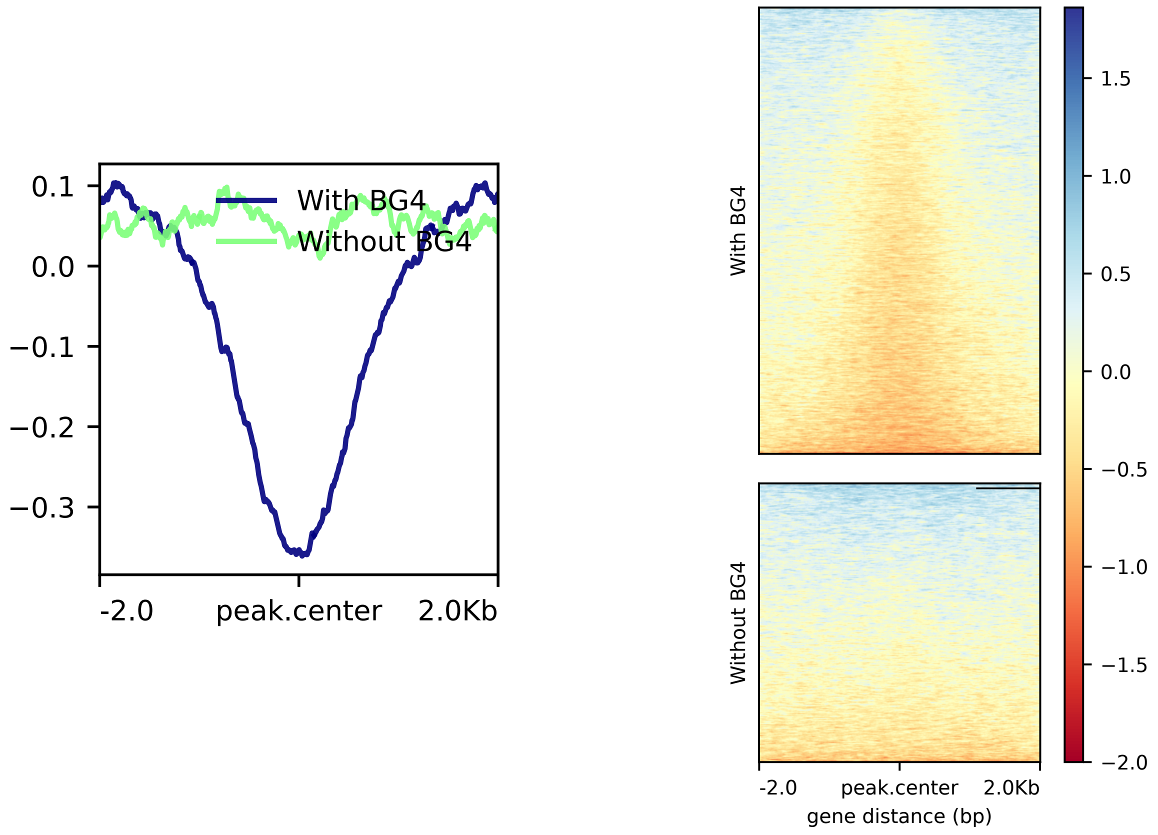

- Step1: generate log2FC profile of FUS CnT between G4L-PROTAC and PDS control.
- Step2: extract G4 and non-G4 peaks for PROTAC and PDS control, respectively.
- Step3: visualize the log2FC profile around G4 and non-G4 peaks from PDS control.
- Step4: visualize the log2FC profile around G4 and non-G4 peaks from PROTAC.
